# iOri-Human: identify human origin of replication by incorporating dinucleotide physicochemical properties into pseudo nucleotide composition

**DOI:** 10.18632/oncotarget.11975

**Published:** 2016-09-12

**Authors:** Chang-Jian Zhang, Hua Tang, Wen-Chao Li, Hao Lin, Wei Chen, Kuo-Chen Chou

**Affiliations:** ^1^ Key Laboratory for Neuro-Information of Ministry of Education, School of Life Science and Technology, Center for Informational Biology, University of Electronic Science and Technology of China, Chengdu, 610054, China; ^2^ Department of Pathophysiology, Southwest Medical University, Luzhou, 646000, China; ^3^ Department of Physics, School of Sciences, and Center for Genomics and Computational Biology, North China University of Science and Technology, Tangshan, 063000, China; ^4^ Gordon Life Science Institute, Boston, MA, 02478, USA

**Keywords:** human DNA, origin of replication, pseudo k-tuple nucleotide composition, physicochemical properties of dinucleotides

## Abstract

The initiation of replication is an extremely important process in DNA life cycle. Given an uncharacterized DNA sequence, can we identify where its origin of replication (ORI) is located? It is no doubt a fundamental problem in genome analysis. Particularly, with the rapid development of genome sequencing technology that results in a huge amount of sequence data, it is highly desired to develop computational methods for rapidly and effectively identifying the ORIs in these genomes. Unfortunately, by means of the existing computational methods, such as sequence alignment or kmer strategies, it could hardly achieve decent success rates. To address this problem, we developed a predictor called “iOri-Human”. Rigorous jackknife tests have shown that its overall accuracy and stability in identifying human ORIs are over 75% and 50%, respectively. In the predictor, it is through the pseudo nucleotide composition (an extension of pseudo amino acid composition) that 96 physicochemical properties for the 16 possible constituent dinucleotides have been incorporated to reflect the global sequence patterns in DNA as well as its local sequence patterns. Moreover, a user-friendly web-server for iOri-Human has been established at http://lin.uestc.edu.cn/server/iOri-Human.html, by which users can easily get their desired results without the need to through the complicated mathematics involved.

## INTRODUCTION

DNA replication is a basic biochemical process during cell growth and division [1]. The initiation of DNA replication in eukaryotes occurs at specific genomic loci called “ORI” (origin of replication) or “RO” (replication origin) [2]. Timely duplication of the genome is an essential step in the reproduction of any cell [3]. There is only one ORI for most of bacterial genomes [4]. In contrast to that, eukaryotic genomes contain much more ORI sites [5]. Although eukaryotic replication mechanism is quite conservative, DNA replication initiator lacks obvious consensus sequence or structure between the different species [6].

The ORI in *Saccharomyces cerevisiae* (*S. cerevisiae*) is formed by domain A, domain B and domain C [7]. Each of the three domains has its special motif and function as elaborated in [8–9]. Interestingly, in the *S. cerevisiae* genome there are over 12,000 conservative sequences, of which, however, only 400 are of ORI [10].

**Figure 1:**
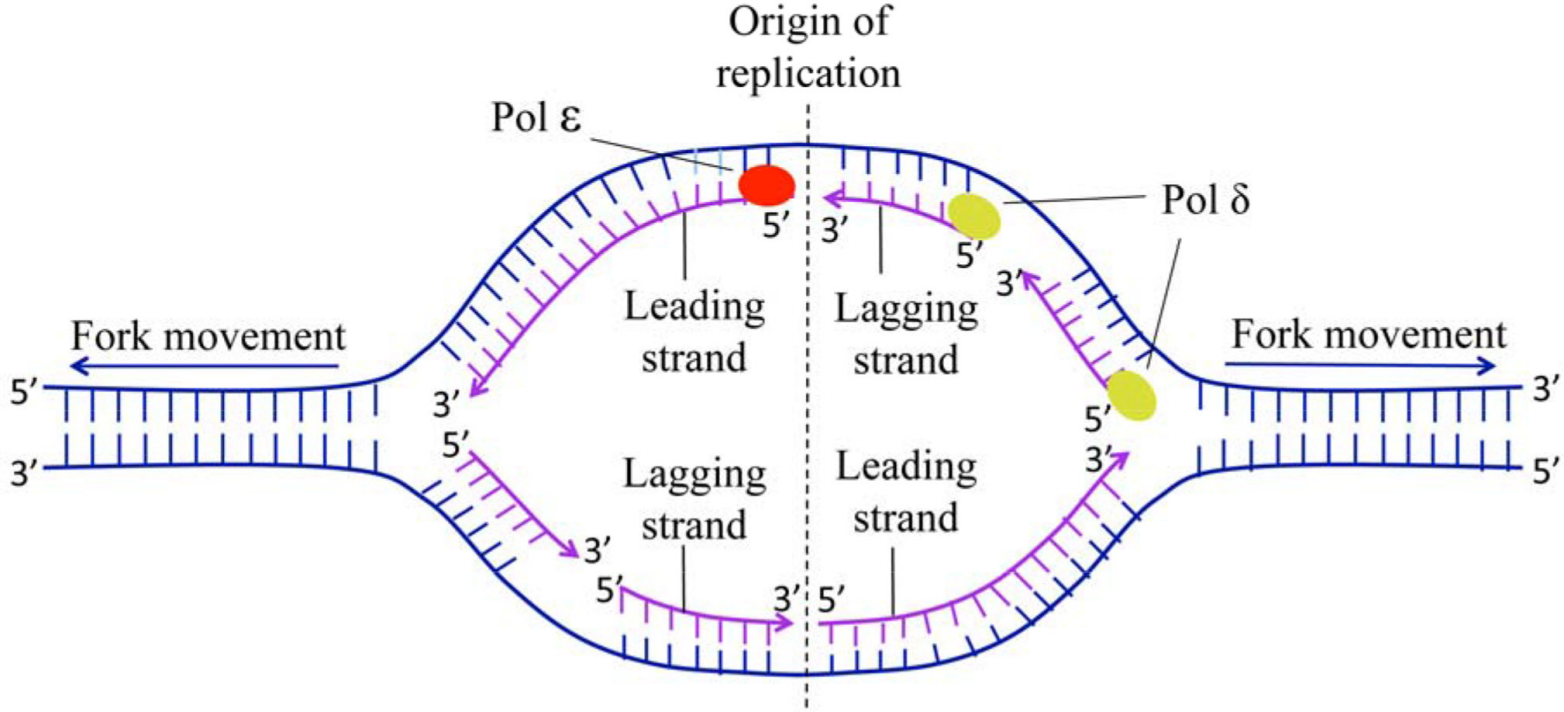
The schematic diagram of origin of replication of human The process of DNA replication requires two DNA polymerase complexes traveling in opposite direction (i.e. two bidirectional replication forks) from the origin.

For the detailed replication process in human DNA, see [11–13] as well as Figure 1.

Although Chip (chromatin immunoprecipitation) is a very powerful technique to determine the ORI [14], it is time-consuming and costly. Therefore, it would be very helpful to develop bioinformatics tools in this regard.

Actually, considerable efforts [15–20] have been made for this purpose. Although these methods achieved encouraging results, the outcomes were often inconsistent and with limited accuracy. Particularly, none of these methods had taken into account the physicochemical properties of the DNA sequence concerned, one of the vitally important factors for conducting genome analysis, as indicated by a serious of recent studies (see, e.g., [21–27]).

The current study was devoted to establish a new computational method for predicting human ORIs based on the DNA's physicochemical properties.

According to Chou's 5-step rule [28], in developing a new statistical predictor we should make the following five procedures very clear as done in a series of recent publications [29–39]: (1) benchmark dataset; (2) sample formulation; (3) operation engine or algorithm; (4) accuracy evaluation; and (5) web-server. In the rest of this paper, we are to address these five steps one-by-one. However, to match the style of the Oncotarget journal, their order may be somewhat different.

## RESULTS AND DISCUSSION

### A new predictor as well as its web-server and user guide

A new and much more accurate sequence-based method, called iOri-Human, was developed for predicting the ORI sites in human DNA. In addition to the predictor's high accuracy, it is also very important to make its web-server available to the public [40, 41]. Because only with this, can it be widely used by most experimental scientists. In view of this, the web-server for iOri-Human has also been established. Furthermore, a user's guide is provided as follows.

**Figure 2:**
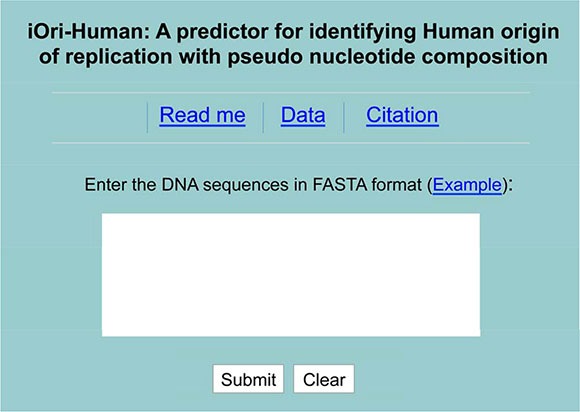
A semi-screenshot for the top-page of the iOri-Human web-server at http://lin.uestc.edu.cn/server/iOri-Human.html

1. Click the web server at http://lin.uestc.edu.cn/server/iOri-Human.html, the top page of iOri-Human will be shown on your computer screen, as shown in Figure 2.
2. In the input box at the center of Figure 2, type or copy/paste the query DNA sequences. The entered DNA sequences should be with the FASTA format. If not familiar with FASTA, click the button of Example.
3. See the predicted results by clicking on the Submit button. If using the three query sequences in the Example window, you will see the following outcomes on your computer's screen: the one for the first query sequence (with 300-bp long) is ‘Ori’; the one for the second query sequence (also with 300-bp long) is ‘non-Ori’; the one for the third query sequence (with 514-bp long) contains 514 – 300 + 1 = 215 sub-results, where the results for the segments from #1 to #134 are of ‘non-Ori’, those for the segments from #135 to #204 are of ‘Ori’, and those from #205 to #215 are of ‘non-Ori’, fully consistent with the experimental observations. The computational time is about a few seconds; of course, the more the number of query sequences, the longer the computational time will be.
4. To get the benchmark dataset, click on the Data button.
5. To find out the key relevant publications, click on the Citation button.

Caveats. The input query sequences should be 300 bp or longer, and expressed by DNA's single-letter codes: ‘A’, ‘C’, ‘G’, and ‘T’.

### The anticipated prediction accuracy

Listed in Table 1 are the success scores achieved by the predictor iOri-Human using the jackknife tests on the benchmark dataset (Supporting Information S1). Since it is the first predictor ever documented for identifying the ORI sites in human DNA sequences, it is not possible to demonstrate its power by a comparison with its published counterparts for exactly the same purpose. Nevertheless, it would be instructive to also list in Table 1 the corresponding optimal scores by the other machine-learning algorithms. As we can see from the table, the new iOri-Human achieved remarkably higher scores than its cohorts in almost all the four metrics, indicating clearly that the proposed new iOri-Human predictor is really very powerful. Note that, of the four metrics in Eq.8, the most important are the accuracy (Acc) and Mathew's correlation coefficient (MCC): the former reflects the overall accuracy of a predictor; while the latter, its stability in practical applications. The metrics sensitivity (Sn) and specificity (Sp) are used to measure a predictor from two different angles. When, and only when, both Sn and Sp of the predictor A are higher than those of the predictor B, can we say A is better than B. Actually, Sn and Sp are constrained with each other [42]. Therefore, it is meaningless to single out one from the two for making comparison. Accordingly, a really meaningful comparison in this regard should count the rates of both Sn and Sp, or even better the rate of their combination. That is exactly what MCC stand for.

**Table 1:** The success rates obtained by various machine-learning algorithms via jackknife tests on the benchmark dataset (Supporting Information S1)

Algorithm	Sn^a^	Sp^a^	Acc^a^	MCC^a^	AUC^b^
iOri-Human^c^	**0.762**	**0.739**	**0.75**	**0.501**	**0.835**
SVM^d^	0.688	0.544	0.616	0.400	0.651
Naive Bayes	0.379	0.746	0.563	0.286	0.614
KNN^e^	0.606	0.473	0.54	0.144	0.529
Decision Tree	0.078	0.936	0.508	0.028	0.511

a See Eq.8 for the definition of the metrics.

b AUC means the area under the ROC curves in Figure 3; the greater the AUC value is, the better the predictor will be [53, 54].

c The proposed predictor in which the number of trees used was 100 with seed equal to 1.

d The optimal parameters used for SVM were *C*= 0.5 and *γ* = 0.125.

e The optimal parameters used for KNN (K nearest neighbor) was *K* = 1.

In studying complicated biological systems, graphical analysis is a very useful approach [43–52] due to its intuitivity. Here, let us use the ROC (receiver operating characteristic) graph [53, 54] to show the advantage of iOri-Human over its cohorts. The red graphic line in Figure 3 is the ROC curve for the iOri-Human predictor, while those of its cohorts are with different colors as directly marked on the figure. The area under the ROC curve is called AUC (area under the curve). The larger the area, the better the corresponding predictor [53, 54]. It can be seen from Figure 3, the iOri-Human has the largest AUC in comparison with its cohorts, once again indicating its power.

**Figure 3:**
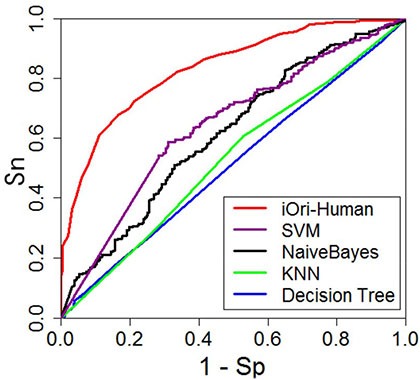
A graphical illustration to show the performances of iOri-Human and its cohorts via the ROC (receiver operating characteristic) curves [53, 54] The area under the ROC curve is called AUC (area under the curve). The greater the AUC value is, the better the performance will be. See the text for further explanation.

## MATERIALS AND METHODS

### Benchmark dataset

The human ORIs data were collected from OriDB [55] (http://tubic.tju.edu.cn/deori/). These sequences were derived from *Hela* cell line. To construct a reliable benchmark dataset, the following steps were followed. (1) Collected were only experiment-confirmed data; thus we obtained 283 human ORIs with 300 bp in length. (2) For each of the 283 ORIs, extract a 300 bp segment from its upstream region at [−600 bp, −300 bp] as the corresponding non-ORI; a total 283 non-ORI samples were obtained. (3) Use the CD-HIT software [56] and set 0.75 as the threshold to remove redundant samples. Note that using 0.75 for the cutoff threshold was a compromise between reducing redundancy bias and keeping enough number of samples for statistical analysis. If further imposing more stringent cutoff, the number of DNA samples left would be too few to have statistical significance. Finally, we obtained 283 human OTI samples and 282 non-ORI samples.

In literature, the benchmark dataset usually consists of a training dataset and a testing dataset: the former is constructed for the purpose of training a proposed model, while the latter for the purpose of testing it. As pointed out by a comprehensive review [57], however, there is no need to separate a benchmark dataset into a training dataset and a testing dataset for validating a prediction method if it is tested by the jackknife or subsampling (K-fold) cross-validation since the outcome thus obtained is actually from a combination of many different independent dataset tests. Therefore, the benchmark dataset S for the current study can be formulated as

(1) $$S=S^{+}\cup S^{-}$$

where the positive subset $S^{+}$ contains 283 human ORI samples, the negative subset $S^{-}$ contains 282 non-ORI samples, and the symbol ∪ represents the union in the set theory. The 283 + 282 = 565 DNA samples are each consist of 300 bp, as can be generally formulated by

(2) $$D=N_{1}N_{2}N_{3}\cdots N_{i}\cdots N_{300}$$

For readers' convenience, their detained sequences are given in Supporting Information S1.

### Pseudo *k*-tuple nucleotide composition

With the explosive growth of biological sequences generated in the post-genomic age, one of the most challenging problems in computational biology is how to formulate a biological sequence with a discrete model or vector, yet still considerably keep its sequence pattern or key feature. This is because almost all the existing machine-learning algorithms were developed to handle vector but not sequence samples, as elaborated in [40]. But a vector defined in a discrete model may completely lose this kind of sequence-pattern information. To overcome this problem, the “pseudo amino acid composition” [58, 59] or Chou's PseAAC [60–62] was developed to deal with protein/peptide sequences. Ever since PseAAC was proposed, it has penetrated into many biomedicine/drug development areas [63, 64] and nearly all the areas of computational proteomics (see, e.g., [65–70] as well as a long list of references cited in [71, 72]). Encouraged by its successes in computational proteomics, the idea of PseAAC was recently extended to dealing with DNA/RNA sequences in many important problems of genome analysis [23–27, 33, 35, 38, 73, 74] by introducing the pseudo nucleotide composition or PseKNC [75–79].

According to a recent review paper [41], the general form of PseKNC for a DNA sequence can be formulated as

(3) $$D={\left[\phi_{1}\;\phi_{2}\;\cdots \;\phi_{u}\;\cdots \;\phi_{z}\right]}^{T}$$

where **T** is the transpose operator, while *Z* an integer to reflect the vector's dimension. The value of *Z* as well as the components ϕ_*u*_ in Eq.3 will depend on how to extract the desired information from the DNA sequence. In the current study, we used the type-1 PseKNC [41], then the component in Eq.3 are given by

(4) $$\phi_{u}=\{\begin{array}{ll} \frac{f_{u}^{\text{kmer}}}{\sum_{i=1}^{4k}f_{i}^{\text{kmer}}+w\sum_{j=1}^{\lambda }\theta_{j}} & \left(1\leq u\leq 4^{k}\right)\\ \frac{w\theta_{u-4^{k}}}{\sum_{i=1}^{4k}f_{i}^{\text{kmer}}+w\sum_{j=1}^{\lambda }\theta_{j}} & \left(4^{k}+1\leq u\leq 4^{k}+\lambda \right) \end{array}$$

where $f_{i}^{kmer}$ is the normalized occurrence frequency of the *i*-th kmer in the DNA sequence of Eq.2, *λ* is the correlation tier used to reflect the long-range or global sequence pattern [41, 58], *w* is the factor used to adjust the weight between the local and global sequence coupling effects, and θ_*j*_ is the *j*-th structural correlation factor between all the *j*-th most contiguous dinucleotides as given by

(5) $$\theta_{j}=\frac{1}{L-j-1}\sum_{i=1}^{L-j-1}\Theta \left(N_{i}N_{i+1},N_{i+j},N_{i+j+1}\right)\left(j=1,2,\cdots ,\lambda \right)$$

where the correlation factor (Θ(N_*i*_ N_*i*+1_, N_*i*+*j*_ N_*i*+*j*+1_) is given by

(6) $$\Theta \left(N_{i}N_{i+1},N_{i+j}N_{i+j+1}\right)=\frac{1}{\Phi }{\sum_{\nu =1}^{\Phi }\left[P_{\nu }\left(N_{i}N_{i+1}\right)-P_{\nu }\left(N_{i+j}N_{i+j+1}\right)\right]}^{2}$$

where **Φ** is the number of local DNA structural properties considered that is equal to 6 in the current study as will be explained below; (*P*_ν_(N_*i*_N_*i*+1_)) is the numerical value of the *v*-th physicochemical property for the dinucleotide at position *i*.

The spatial arrangements of any two successive base pairs could be characterized by six types of local structural parameters, of which three are local translational parameters (shift, slide and rise) and the other three are local angular parameters (twist, tilt and roll) [25, 26]. In recent years, more and more researches have demonstrated that the six DNA structural properties play important roles in many biological processes [80, 81]. There are 4^2^ = 16 different dinucleotides, so the total number of local structural parameters is 6 × 16 = 96. Each of their parameter values can be found in Supplementary Table S1.

Before substituting these values into Eq.6, they were subjected to a standard conversion according to the following equation [82]

(7) $$P_{\nu }\left(N_{i}N_{i+1}\right)⇐\frac{P_{\nu }\left(N_{i}N_{i+1}\right)-<P_{\nu }>}{\text{SD}\left(P_{\nu }\right)}$$

where the *P*_ν_(N_*i*_N_*i*+1_) is the original value of the ν-th DNA physicochemical index for the dinucleotide N_*i*_N_*i*+1_ at position *i*; the symbol 〈□〉 means the average value of the quantity therein for 16 different indices of dinucleotides, and SD denotes the corresponding standard deviation. The advantage to carry out the standard conversion is that the converted values obtained by Eq.7 will have a zero mean value over the 16 different indices, and will remain unchanged if they go through the same conversion procedure again. See Supplementary Table S2 for the corresponding values converted via Eq.7 from Supplementary Table S1.

### Random forest

The random forests (RF) algorithm is a very powerful algorithm, widely used in many areas of computational biology (see, e.g. [2, 31, 32, 34, 36, 39, 83–89]). The idea of RF is based on the ensemble of a large number of decision trees, with each giving a classification to choose the final outcome via a vote over all the trees in the forest. In this study, the number of trees is 100 and the seed is 1. The detailed procedures of RF and its formulation have been very clearly elaborated in [90], and hence there is no need to repeat here.

The predictor obtained via the aforementioned procedures is called iOri-Human, where “i” stands for “identify”, and “Ori**-**Human” for “human origin of replication”.

As stated in Introduction, how to objectively evaluate its anticipated success rates is an indispensable procedure for developing a useful predictor [28]. To realize this, we need to consider two issues: one is what metrics should be defined to measure the predictor's quality; the other is what kind of test approach should be adopted to derive the metrics values. Below, let us address the two issues.

### A set of four intuitive metrics and their definitions

As stated in [91], to quantitatively evaluate the quality of a predictor in performing binary classification, four metrics are usually needed. They are: (1) Acc to measure the predictor's overall accuracy; (2) MCC, the stability; (3) Sn, the sensitivity; and (4) Sp, the specificity. Unfortunately, the conventional formulations for the four metrics are not quite intuitive and most experimental scientists feel difficult to understand them, particularly the stability of MCC. Fortunately, as elaborated in [22, 92], by using the Chou's symbols and derivation in studying signal peptides [93], the conventional metrics can be converted into a set of four intuitive equations, as given below:

(8) $$\{\begin{array}{ll} \text{Sn}=1-\frac{N_{-}^{+}}{N^{+}} & 0\leq \text{Sn}\leq 1\\ \text{Sp}=1-\frac{N_{+}^{-}}{N^{-}} & 0\leq \text{Sp}\leq 1\\ \text{Acc}=\Lambda =1-\frac{N_{-}^{+}+N_{+}^{-}}{N^{+}+N^{-}} & 0\leq \text{Acc}\leq 1\\ \text{MCC}=\frac{1-\left(\frac{N_{-}^{+}}{N^{+}}+\frac{N_{+}^{-}}{N^{-}}\right)}{\sqrt{\left(1+\frac{N_{+}^{-}-N_{-}^{+}}{N^{+}}\right)}\left(1+\frac{N_{-}^{+}-N_{+}^{-}}{N^{-}}\right)} & -1\leq \text{MCC}\leq 1 \end{array}$$

where *N*^+^ represents the total number of ORI samples investigated, while $N_{+}^{-}$ is the number of true ORIs incorrectly predicted to be of non-ORI; *N*^−^ the total number of the non-ORI samples investigated, while $N_{-}^{+}$ the number of the non-ORIs incorrectly predicted to be of ORI.

According to Eq.8, it is crystal clear to see the following. When $N_{-}^{+}=0$ meaning none of the true ORI sequences are incorrectly predicted to be of non-ORI, we have the sensitivity Sn = 1. When $N_{-}^{+}=N^{+}$ meaning that all the ORI samples are incorrectly predicted to be of non-ORI, we have the sensitivity Sn = 0. Likewise, when $N_{+}^{-}=0$ meaning none of the non-ORI samples are incorrectly predicted to be of ORI, we have the specificity Sp = 1; whereas $N_{+}^{-}=N^{-}$ meaning that all the non-ORI sequences are incorrectly predicted to be of ORI, we have the specificity Sp = 0. When $N_{-}^{+}=N_{+}^{-}=0$ meaning that none of ORI samples in the positive dataset and none of the non-ORI samples in the negative dataset are incorrectly predicted, we have the overall accuracy Acc = 1 and MCC = 1; when $N_{-}^{+}=N^{+}$ and $N_{+}^{-}=N^{-}$ meaning that all the ORI samples in the positive dataset and all the non-ORI samples in the negative dataset are incorrectly predicted, we have the overall accuracy Acc = 0 and MCC = −1; whereas when *N*^+^ = *N*^+^/2 and $N_{+}^{-}=N^{-}/2$ we have Acc = 0.5 and MCC = 0 meaning no better than random guess. Therefore, Eq.8 has made the meanings of sensitivity, specificity, overall accuracy, and stability much more intuitive and easier-to-understand, particularly for the meaning of MCC, as concurred recently by many investigators (see, e.g., [24, 25, 27, 33, 38, 73, 86, 87, 89, 94–101]).

Note that, however, the set of equations defined in Eq.8 is valid only for the single-label systems. For the multi-label systems whose emergence has become more frequent in system biology [102–104] and system medicine [105] or biomedicine [39], a completely different set of metrics are needed as elaborated in [106].

### Jackknife cross validation

With a set of intuitive metrics to measure the quality of a predictor, the next issue is what kind of validation method should be utilized to score these metrics. In statistics, the following three cross-validation methods are often used: (1) independent dataset test, (2) subsampling (or K-fold cross-validation) test, and (3) jackknife test [107]. Of these three, however, the jackknife test is deemed the least arbitrary that can always yield a unique outcome for a given benchmark dataset as elucidated in [28]. Accordingly, the jackknife test has been widely recognized and increasingly used by investigators to examine the quality of various predictors (see, e.g., [65–68, 70, 108–119]).

In view of this, here we also used the jackknife test to examine the quality of iOri-Human predictor. During the jackknifing process, both the training dataset and testing dataset are actually open, and each sample will be in turn moved between the two. The jackknife test can exclude the “memory” effect. Also, the arbitrariness problem as mentioned in [28] with the independent dataset and subsampling tests can be completely avoided because the outcome obtained by the jackknife cross-validation is always unique for a given benchmark dataset.

### Optimize parameters

As we can see from Eqs.4–5, the new predictor contains three parameters: one is *k*, the number of the nearest nucleotides considered to reflect the short-range or local pattern; one is λ, the number of the correlation tiers considered to reflect the long-range or global pattern; and one is *w*, the weight factor considered to adjust the effects between *k* and λ. Their values will be determined via an optimization procedure according to various concrete problems. For the current study, the grid search for the optimal values of the three parameters was conducted within the scope given below

(9) $$\{\begin{array}{ll} 1\leq k\leq 4 & \left(\text{with step }\Delta k=1\right)\\ 1\leq \lambda \leq 10 & \left(\text{with step }\Delta \lambda =1\right)\\ 0.1\leq w\leq 1.0 & \left(\text{with step }\Delta w=0.1\right) \end{array}$$

Where Δ*k*, Δ*λ* and Δ*w* represent the step gaps for *k*, *λ*, and *w*, respectively. The reason why the search scope for *k* is limited under 4 is because the possible number of *k*-mers (4^*k*^) would be too large to be covered by the current benchmark dataset. As for the parameter *λ*, generally speaking the greater it is, the more global sequence-order information the model will contain. However, if *λ* is too large, it would reduce the cluster-tolerant capacity [120] so as to lower down the cross-validation accuracy due to overfitting or “high dimension disaster” problem [121].

From Eq.9, a total of 4 × 10 × 10 = 400 individual combinations were investigated for finding the optimal parameter combination. To reduce the computational time, the 10-fold cross-validation approach was used to assess the performances of the 400 combinations. Once the optimal values of the three parameters were determined, the rigorous jackknife test was adopted to calculate the scores for the four metrics defined in Eq.8 as well as the AUC in Figure 3. The final values thus obtained are given below

10 $$\{\begin{array}{cc} \begin{array}{l} \text{Sn}=0.762\\ \text{Sp}=0.739\\ \text{Acc}=0.750\\ \text{MCC}=0.501\\ \text{AUC}=0.835 \end{array} & \left(k=4,\lambda =7,w=0.9\right) \end{array}$$

Also, see the results listed in Table 1, where the corresponding optimal parameters for various operation engines are also given.

## CONCLUSIONS

One of the most important and fundamental processes in human cells is of the DNA replication. Knowledge of ORIs is crucial for in-depth understanding such a biological process, and hence computational method is highly demanded in this area. Unfortunately, it was very difficult to achieve decent success rates by computational approach. In the current model, both the local and global sequence patterns of DNA can be reflected via its physicochemical properties. That is why the iOri-Human predictor can yield remarkably high success rates. We anticipate that it will become a very useful high throughput tool for genome analysis.

## SUPPLEMENTARY MATERIALS




