# Supplementary material for: iOri-Human: identify human origin of replication by incorporating dinucleotide physicochemical properties into pseudo nucleotide composition
Source: Oncotarget. 2016 Sep 12;7(43):69783–93. doi: 10.18632/oncotarget.11975 (PMC5342515; doi:10.18632/oncotarget.11975)
Supplement: Supplementary file 1 [file oncotarget-07-69783-s001.pdf]

iOri-Human: identify human origin of replication by incorporating dinucleotide physicochemical properties into pseudo nucleotide composition

Supplementary Materials

Supporting Information S1: The benchmark dataset used for studying human ORI (origin of replication). See Supporting_Information_S1

Supporting Information S2

Supplementary Table S1: The original values of the six physical structural properties (taken from Goni, J.R., et al.. Genome Biology, 2007; 8: R263)

Dinucleotide	Rise $P_1(R_iR_{i+1})$	Slide $P_2(R_iR_{i+1})$	Shift $P_3(R_iR_{i+1})$	Twist $P_4(R_iR_{i+1})$	Roll $P_5(R_iR_{i+1})$	Tilt $P_6(R_iR_{i+1})$
AA	7.65	2.26	1.69	0.026	0.020	0.038
AC	8.93	3.03	1.32	0.036	0.023	0.038
AG	7.08	2.03	1.46	0.031	0.019	0.037
AT	9.07	3.83	1.03	0.033	0.022	0.036
CA	6.38	1.78	1.07	0.016	0.017	0.025
CC	8.04	1.65	1.43	0.026	0.019	0.042
CG	6.23	2.00	1.08	0.014	0.016	0.026
CT	7.08	2.03	1.46	0.031	0.019	0.037
GA	8.56	1.93	1.32	0.025	0.020	0.038
GC	9.53	2.61	1.20	0.025	0.026	0.036
GG	8.04	1.65	1.43	0.026	0.019	0.042
GT	8.93	3.03	1.32	0.036	0.023	0.038
TA	6.23	1.20	0.72	0.017	0.016	0.018
TC	8.56	1.93	1.32	0.025	0.020	0.038
TG	6.38	1.78	1.07	0.016	0.017	0.025
TT	7.65	2.26	1.69	0.026	0.020	0.038

Supplementary Table S2: The normalized values obtained from Table S1 via the standard conversion of Eq.7

Dinucleotide	Rise $P_1(R_iR_{i+1})$	Slide $P_2(R_iR_{i+1})$	Shift $P_3(R_iR_{i+1})$	Twist $P_4(R_iR_{i+1})$	Roll $P_5(R_iR_{i+1})$	Tilt $P_6(R_iR_{i+1})$
AA	−0.11	0.11	1.59	0.06	0.09	0.50
AC	1.04	1.29	0.13	1.50	1.19	0.50
AG	−0.62	−0.24	0.68	0.78	−0.28	0.36
AT	1.17	2.51	−1.02	1.07	0.83	0.22
CA	−1.25	−0.62	−0.86	−1.38	−1.01	−1.36
CC	0.24	−0.82	0.56	0.06	−0.28	1.08
CG	−1.39	−0.29	−0.82	−1.66	−1.38	−1.22
CT	−0.62	−0.24	0.68	0.78	−0.28	0.36
GA	0.71	−0.39	0.13	−0.08	0.09	0.50
GC	1.59	0.65	−0.35	−0.08	2.30	0.22
GG	0.24	−0.82	0.56	0.06	−0.28	1.08
GT	1.04	1.29	0.13	1.50	1.19	0.50
TA	−1.39	−1.51	−2.24	−1.23	−1.38	−2.37
TC	0.71	−0.39	0.13	−0.08	0.09	0.50
TG	−1.25	−0.62	−0.86	−1.38	−1.01	−1.36
TT	−0.11	0.11	1.59	0.06	0.09	0.50
