# Supplementary material for: iOri-Human: identify human origin of replication by incorporating dinucleotide physicochemical properties into pseudo nucleotide composition
Source: Oncotarget. 2016 Sep 12;7(43):69783–93. doi: 10.18632/oncotarget.11975 (PMC5342515; doi:10.18632/oncotarget.11975)
Supplement: Supplementary file 2 [file oncotarget-07-69783-s002.docx]

**Supporting Information S1. The benchmark dataset used for studying human ORI (origin of replication).** It consists of a positive dataset and a negative dataset. The former contains 283 experiment-confirmed human ORI sequences; while the later 282 human non-ORI sequences. Each of these sequences is 300-bp long. None of the sequences included has $\geq75\%$ pairwise sequence identity with any other.

**I. List of the 283 human ORI in the positive subset**

>ORI-1

GTTAAACTTGTGGAGGGGGTGCGGGACGTGAGTTCTTCCCCATGCCAGGCGAATGGTGTGGCCTTGAGCTGGTCCAGGAGCCGGCTCGACGTGTCTGAGGGAGGCCCCGGAGGGGGCGGGGAGGTGGCCCACAGAACGCGGGTTCTGTAAAGAGACGTTGGGAAGATTCGATTCCGAGAAGAGGAAGAACCGGATTGAAAGAGAGCCAGGCCGCTGAGGGGGAGGGGGCTGCTAAGATGGCGTCGGCCTCCTCCGGGCCGTCGTCTTCGGTCGGTTTTTCATCCTTTGATCCCGCGGTCC

>ORI-2

GTGCTACTAGCAGTTTGAGAGAATCCCACATCTGGAGAGGAAGGAAATAAGAACAACACGCAGAAAGACGGAAAACAAATAGATCAATGACAGTACTCCTTTGCAAAAGGTGCCAAGAATCTAAATAAAATAGTCCCCGGGATAAACATGGCGCCCACCGCGGCAGGACATAATCACCAGAAAATAAAGATTCCCCGCCTCATTCCATTTTATAAATCACGGCGCCATCCCAAAAGAAATTTGGGGACCGAGGGAGGAGACTTCGTAAACTAGCTGCAGTGCACTAAGGCTTAGGATGTA

>ORI-3

ACAGTAAAAAGCATATTCTACATCTTGACTGGCTACACACAAACACACAGTACTTTTCATGATTCAGATTGTATGAAAGACTACTGATATTCACTATGTGATAGCTTTCTATTCTCTTTTATTACACCTCTTTTTTTTTTTTTTTTTTTTTTGAGACAGTGTCTTGCTCCGTTGCCCAAGCTGGAGTGCAGTGGCTTATAAAACATGGCTCACTGCAGCCTTGAACTCCTGGGCTCATGCAATCCTCCTGCCTCAGCCTCTGGAGTAGCTGGGACCACAGAAGTGCACCACCACACCCGG

>ORI-4

TTTTGGTAAATTTGCGTCCCCGGTTTCCATTCATTCAGTCAACAAATATTTATTAAGCATGTAGTATGTACCAGTCCCTGTTGTAGGCGTTTGGGGTGAAGCAAGGCACAGAACAAGAGATCCTTGCTCTGGTGAAGCTTATCGTAGGACGGGAGGCAAAATGATGAAAATGATTTTTAAAATTATGTTATTAACTAGAAGGTGATAAATATTACATAGAAAAAGATGTCAGGCTGCAAAATGATTGCGGAGGCCACAAGATAAAAGAGAATCCAAGATCAGGGTTCTGGTTCGGGTCAG

>ORI-5

GAAGGAGTCACCTATCTGAAAAGTGGTGCTTGTGGGTTCCAGGTGTCACTTTAAAGTGGACCTCAATCAACTGTGAGCCAGTTAACCCTGGAAATAGACACCCTGCTGGAGTGCTGAGGTGGTCCTGTGCAGTGAAGGGGAGGTGGTGGTGAGCCCATCAGTGTCACCACCTTTCTCCCGTCATGGCTCCAGAGCCGCCCAGGTTCCTGGGTTCTTTTCTAAGGTTTTTTTCCATGTTTGCTCTTTTTTCCACACCATTTAACATATTCCTGCTCCGTGTTTTAGGTCACACTCAGCCGG

>ORI-6

TGTGATCTGTGTGTTTGATGCTAATAAGAATTTTATATTAATAATCAATGATCGAATAATTACATATTAAATGAAAAGCCATTTATTTGTTTAAAATAATGATTAACATTGAATGTGTTCATGGTATTTTTGTTAAGTTTACAAAATCCATATGTGTTACAAATACTAGTATGTTTATATTTGACATGATTGGATTCTCAAGAGTTACTACATAATGCTCTAAAAGAAAGGGTGGTGCTGGGGTTGGATGGGGGGCCAAATTAAACAAATATTGTTGGTTCTGGGTCCGAGAATGTTTAT

>ORI-7

TAACTTCATATGGTGGAATAATATACAGTTGTGAAAATGAACTAGAGTTTGTATTGAATATGGATAAACATGAAAAAATATAAATAGGAAGTTGAGTTTTAAAAAAAGCAGCATTCATAATGATTTGCCAAATTTACAAACATTCAAAATAATATCCACTGCTATGGATACATAAATGGGTAGCAAAAATGTGAGCACATGCATGAGAGTTATGATCATGACCTTGCTTTTCTCTGCAGAAACAGAAGAGGAGGGAGCAGGAAGGGAGCAGAAAGGAGGAACTGAGAAAATCACCATCTG

>ORI-8

AAAGAAAGCTTGCAGGCTACTTTGAATGACAATGAGAAAGACGGTGCTGCCTGAGTGTGTTAAGGATCCACATGGTCTCCAAAATCCTCCAGGAGCATACAGTCTAGTCTGGGAGATGAGACACAAAAATAACCAGAACACAACAGCTTGCACTGACTCGAGGGCTGGATAAGAATATCTGGAACTCCCCCATCTATTTCAGAAGCTTGTCTCTTGGATGAAAATTAGACACTTAATGGGAAAGGGCTTTGAAAAGAGTGCAGTAACAAAGCCCCCTTTACAATTTACCCGGCACATTCA

>ORI-9

CTGACACCCTGGTGGGCACCTCCCTGGACACACCCCCGACCTCCGTGACAGGCACCTCAGAGGAGCAAGTGAGCTGGTGGGGCAGCGGGCAGACGGTCCTGGAGCAGGAAGCGGGCAGTGGGGGTGGCACCCGCCGCCTCCCGGGCAGCCCAAGGCAAGCACAGGCAACCGGGGCCGGGCCACGGCACCTGGGGGTGGAGCCGCTGGTGCGGGCATCTCGAGCTAATCTGGTGGGCGCAAGCTGGGGGTCAGAGGATAGCCTTTCCGTGGCCAGTGACCTGTACGGCAGCGCATTCAGCC

>ORI-10

CAAGTAATCCGTCCACCTCGGCTTCCCAAAGTGCTGAGATTATAGATGTGAGCCACCGCCTCGGGCCTTAGGATTCTTTTTATAATTTTTCCTTTAAAGATGTAGTTTCTCAAACTAGTTACTACCTAATGCATTAGGTAGTAACTGTTTGGTTTTCTAATTTGTTAATTAACTCTAGACATATGTAACCGCCACTCAGAACTCCTGATCAACATGGGCTAAACAGGGTTACCTGCTGAGTAAATACAATTCCAACCAAAAGCAAAGAAAGTGACATTTTACTTAGCACAGGCAATCTTA

>ORI-11

CCAAGCCTGCACCACCCCCCCAGGCCCTGCAAACCCTAGCGCTGCCCCTCACACCCTATGCTCAGATCATTCAGTCCCTCCAGCTGTCAGGCCACGCCCAGGGCCCCTCGCAGGGCCCTGCCGCGCCGCCTTCAGAGCCCAAGCCCCACGCTGCTGTCTTTGCCAGGGTGGCCTCCCCACCTCCGGGAGCCCCCGAGAAGCGCGTGCCCTCAGCCGGGGGTCCCCCGGTGCTAGCCGAGAAAGCCCGAGTTCCCACGGTGCCCCCCAGGCCAGGCAGCAGTCTCAGTAGCAGCATCGAAA

>ORI-12

CCGTGTACTGACCACGGCTCCGCCCTCCTCCCCGCCGCCCTTCCGCAGGGTGTTCTTCGAGATCGGCAGCACCACCACGACGCTGACCATTCGCCGGGCCACGCCTCAGGACAGCGGCAAGTACGAGGTGTACGTGGAGAACAGCCTGGGCATGGACCAGAGCTTCGCTCGCGTCGACGTGGCCTGAACGGCACCCCCGGACCTTCCGCCCTGGCGCCGAGCCCGGGGGTGGTGGGACCCACAGCCCTCCACCAGCTTGCTTAATAAAGCTGCTCTCTGACCCTCCGCGTCTGTCCTGCT

>ORI-13

ATGACCCCCATGTGCCCCTCTTGGTGCCCCCCTTCCCCAGCTGTGGGTGTGGAGCTGTGTTGCCAGATGAGCTGGTAATGATCGTAATCTGCTAATTGAGAGGAATCTAATTACCCGAAGAGAATGGGGGGTGCAGGGCATGGGTGGGAGAAAGGAGGGGGTCCTGGCAGCAGGCAGGGGTGGCAGGCAGCAGGGTGCTGGGGCCTTGTTATCAGCACCAGGCTCTGGGACAGAGCCCAGGGTTTCATAGCTTCATAGCCTGGCAGTTAGGAAGCTGCGCCTTCTCAGGAGCTGGACTGA

>ORI-14

TGGTCCGCACCAGGGCCAGGTGTCTGGGCTGGGGAGGGTGTCCTCTGGGTGCAGGGGGAGGGAGGCTGGGCATCTTTTGGAGAATGCTGTCTAGGAGTTCAGGGGGTCACAGCATTTTTGAATGGGGCCAGCCAGGCAAGGAATCTGGGCATCAGCTTGGGGGTTCCTGTGCTTTGAGAAAGCACCCTTGGAACCCCCCAGGTTGGATCCTGGGAGGTCTCTGGGTCTGCCTTTGGGGCACTTTGCCCAAACAAAATCTCCATGCCTCCTCCCAGGAACCTTGAGCCAGAAACCCACTGC

>ORI-15

TTCCTGAGCCCACTGCTGTCCCCGACCCTGAACCCCAAGGTGTCCTGGGATCGACTGAAGCGGGTATGGAGGCGTCCCAAGACCCCCCTGCGGACCTCCACTGGGGGCATCTCCACTTTGGGGCTTCAGGAGCTGAAGGAACAGGTGAGGACAAGCTCTAAATGCCTGCAGCATGCAGCCACACCTCCCCAGGACTGAATACATCCATTGTTTCTGAACCAGCCTGTGGAGGGGGCCCTGGAGCCTCTGCCCGAGGTGACAAGGAAAGGCTGGCGGTGTGAGCCCTGGGGGCACCACTTG

>ORI-16

GGAAGGCAGTGTTGGTGAAGTGGGGAGGAAAGGAACTGGTAGAAACAGGGCTGTTCAAGAGAGACAAACATGGTCCAGTGGCAGGAGAGAGGAGAAGCCTGGCAAGAAGGGGCGTAGAAAGGTGGGAACAGCTGACCAGGAGCAGCTGATCTGAGCCAAGCAGTTCCAGGGCTTTCCAGCCCTGGGTGTGCTCTACCCCTCCCCTCCCCCATCCCCACCTCCTACGTATCCCAGGACCCGTTGCCACCCCAAGTTCTGGAAAGCCTCCCATAGGTGTGGCTTAACCCATCAGCTCCCAGG

>ORI-17

AAATCCCTGGGGCCAGAGGGTGTTACAGAATCCAGAATGCTTTGGATTCCAGAAATGATGCGTTTCTCATCTATCACATACCTTCCTCAGCCCCCACGGGAGCCTCCCCCTTCATCGGTCACGTTAACATGTCTGTGGTATAACACATACTACATTCACCCTAAGCAGGATAGATTGAAAGATACAGAATCTTACAGATACAAGCATATATCTATAAGACACTCATATCTTATAGATATACAGCTATAGCTATATTCACCCTCAGCAGGATAATTAAAGCATAGAAATAGCTTCAGAGCA

>ORI-18

CACGGTGACGGCGGCCGCCGCGTAGGCCTCGCCGGCCGCGTTGCGGGCGCGGCACACGTAGACCCCCGCGTCGGTGGGCAGTGCGGCGGTCAGCAGCAGGCCGTGCTCCGCGCCGTCCGCCGGGAAGCTCAGGCGTTCCGAGGCCGCCAGCTGCTGCCCGCCCTTCTCCCACACCACTACAGGCGGCGGCTCCCCCAGGACCACGCACTTGAGCTCGGCCTCGGCGCCACTTACCACCCGCACAGGCCGCGGGAAGCGCAGGAAGCACGGGGGGCTCCCCTGATCCCCCGAGCTCGCCTT

>ORI-19

CATAGACCATCTGCTGGGACTGCCCTAGGCCCCTCCTTTAGGGCACAGAACGTGGCAATAATGTGTCCCATTTGGCAGCCTGTGTGACATTTCTTCTTGTATCTTATCAGGTGACTCAAATAATGATCATGCCTGTTGACACTTGGAGCTCTCAAAGGGAATTCTGAGCAGCAAAGGAAAGGTGACACAGAACTCAAGGGCACTGGCGCTGAGCGCTGCCCATGCAGCTGGCAATTAAGCCATTGCCGAAAACAACAATAAAACAGATTTTGGGCAAGTAGGGAATGGTGGATTGAATCC

>ORI-20

CCAGGCTGGTCTTGAACTGAGCTCAACTGATTCTCCAGCCTTGGCCTCCCAAAGTGCAGGGATTACAGGTGTGAGCCACCATGTCAGGCTCACACTGGTATTTTATTAAACATTCCCCTCCCCCGACATCCCCTCAAAACCCCAGAGATTCTAATATGGAGTCAGGTTTAAGAACCATTGTCCTAAACCAATATATTCTTTCCTATGGTTGATTTCCACTACAATTTCCACTGTGGTTGAAAGTTATTTCTTATTATTGCTTCTCCTTGGTATGAAAATGCAAATACACAGTATGCATAT

>ORI-21

ACCCGACCCCTCGTGCCCTGGCTGAGTCTGGGGGATCTTCTCTGGGCCTTGTCCTCCTCTTCTGTAAGTGGGGATCCTAATCCTTGCCTCAAGTCCTTCGTGGGGCTGTTGTGGGAATTCCTTTATTTGGAAAGAATTCACCAAGCTCCGACCACACATTCAGCACTGGGTCAGACACTTCGGGGAATAGGGAACAAGGTTAATTCTCAGCCTTCATCCTTAAAGAACTAACAGATGTGAAGGGACTTTCAAGAGTGGTTAGGGGCCGGGTGTGGTGGGTCACGCCTGTAATCCCAGTAC

>ORI-22

TGATTTAGGAGCCATCTGTAGATCATTCTGGCTAAATAATGGGATTCACTTTTAAGTGAAGGAGGCCTTGAATGCCACACTCAGGGGCCTAGCGCAGCACTTCCCAAGCCTTGCTGTGGTTAGGAACAACCTGGGGGCTGGGTGAAATGCAGGCTTGGATTCAGCAGGACTGGGGTAGGGCCTGAGAGTCTGCATTTCTAATCAGCTCCCAGGGAATGCTGGTGCTCAGTGGGTCAGTGGGCCCCTGAGTAGCGAGGGTCTAGAGTTTGACCTTGAGTTGGTAGAAGGTCACCTGCCACC

>ORI-23

CCAAGGGGTAACTCGAATAACAGTTGCCCTGCAGCTCCCTGAAGTTAGGACATGCTGAGCTTCTATCAGGGCTGCAGCTGAGATTACTTATTGCCTGGAGTGGACGATACTGCACTAAGCATTGCTCTCCTGGCATGTCTCAGGACGCCTGACTGCTGTTTGCTATTCAGTCCTCGAACAAAGCTTGCCAGTGCCCTTTGCACAGAGGCCTCGATCATTCAGGAAGGTGAGAAATCCAGAACCGATTACCTCTAAACAGCATTTTAAATGAGAATTTCAGGCCTGCTCAGTTTTTTCCAT

>ORI-24

GTGGCCATGGTAACACTCAGGAGACCAGCGCTGGGGAGCTGGAGAGGAACCCTGCCCACAGCCCCACAGCCAGGAGCTCAAGGGTGTTCTCTAGGGCAGAGGGCGCTTTGGTGGCCTCTTCTCAGAATGGTTGATGAAAAGTCAAAAGTCTTTTTTCTGCTACTTTTCACCCCTCAGGCTCCCAAACATTTCTCTACCTGTCCCTGACCCTGCCTCCCTTGCTGCATTAAGGCTTGGAAGCTGCTGTGCAAGTCCAGACGTTGTTTGACTCCTTTAGGCATCTAAAGAAACCAGAGGCTG

>ORI-25

AATGGGGTGAATTATTTTACTGACCTGTGGAATGTGATGGACACGCTGGGGCTTTTTTACTTCATAGCAGGAATTGTATTTCGGTAAGTAGTCTCATCACTTTTCCTAATTTTCTGTGTTTTGACTACAAAAGTGGAGAAAAGCTCTGCAGACATTTCAAACCCAAGGGAAGTCTATTACTCATTAGAGATTATTACACAAGACCATGGTGTCTGCTTTATAGAGATACACCTTGGTTGTCGTGAAGTTGAGTCCCCTCTCTTCCCCCTCCCACTGATGTGTAATTTAAAAGGGGTCCTG

>ORI-26

AGGATGTGGAGAAATAGTAACACTTTTACACTGTTGGTGGGAGTGTGAATTAGTTCAACCATTGTGGAAGACAGTGTGGCAATTCCTCAAGGATCTAGAACTAGAAATACCATTTGACCCAGCAATCCCATTACTGGGTATGTACACAAAGGATTATAAATCATTCTACTATAAAGACACATGCACAGGTATGTTTATTGTGGCACTGTTCACAATAGCAAAGACTTGGAACCAACCCAAATACCTGTCAATGATAGACTGGATAAAGAAAATGTGGCACATATACACCATGGAATACTA

>ORI-27

GTTCCTACTGAGATAGCCAGGGTTCTCCTGGTAATCAAAATATTATAAATGACCTATAAGAAATAACCCTTTTTCTCTAGGAGCTCATATTTTTATATTAAAAAGATATGCAGATTATATAAGCAAAATGGAATAAGTTAAAGTATCAACCACAAATTGCTGCAACAATCTTTGGTATCATTTATCATCATCATAATTATTATTATTACTATGAGTAGCTGAAGAATAACCACAGGATTCAGTGTTTAATATGCATTCTTAGGCCAGGTGCAGTGAATCACGCCTGCAATCCCAGCACTT

>ORI-28

TATCATTTATTTTTTAATAATGTTATATTTAACAACTGGCTTACAAAATCCCTCAAAATCAAACAGTTGGCTCTCAAGAACTAGCGCGAGCCACATTCAGCACCCCACAGGCTCCATTTATTCCTAAGAATAAACTTATTCCCACCCTGATTTCAGGAGCCTACAAATGCTCTAATTTGTTCAACCTAGAGATAAAGTGTCTTTTCAGTTGCTTGCAACCAAAAGAATCTTAGCTAATTTAAGAAGTCCCTCTTGGCTACAGTGGACAGGGAAGGCTTCGTCAAAGAGACAGAGCTAAAG

>ORI-29

AAGGGGTATGAGGCTTTTGTTTCCTCTCTCAGTGCTGAGGCACTGGGCATTACAGATCCACAAGTCAGTTTTGACCCTTGGCCAGACAGGAAGCCCCATGAGGAAAGAAGCTTCATTGGCCTGTTCATTACCATCTCCCCAGCACCTAGCTCAGTGCCTGAAGCCTAGCACCTGCTCATAGTATCTGTTGGATGAATGAAGTCTGCTGTATCTGCATACACATCAGCCATTTCTTGAAAATAGGATTGTTTTGAGATTTTTATGCTGGCCAAATGAAACCTCCCTCAGTATAGGAATATT

>ORI-30

AATACTCAAAAAGTATAAAAAATCAAAATCAAAAATCTCACCTGAGTTTGTCTTTTACCCCTACCCACAGGTCTCATTCATTCATTTGCTCACCTCTTCTACCACTAATTCACTATCCATTTGCCAAGTTTCATGCATGGCTCTGTGGAACAAGTAATATTGTACAGTTCACATAAACTAACCCAGCTTGATTAGCTTAGAAGAAGTAATAAAGGATATGTAAGAATGTCTTATCTACAATTCAAGGCAAATTGTAATTAAAAGTAATACAAAAGAGGATGTTTAGGGCAGTGAAACAAT

>ORI-31

GTCTTCATCCATATCTATTTTATTTATTCAAAAAAATTTTGAAGCAAGAGTGGCAAAATATTAGCACATATTAAAACTAGTTGGATAAATCAAATAAAATAAAAACTAGTTGGTATCAAACTCTCTACTACTGCCACAACTTTTTGTAATTTTATATAATTTTAAAATATTAAAAATAAAATATTGGCCAGGTACCATGGCTCACGCCTGTAATCCCACACTTTGTAAGGCTGAGGCGGGTGAATCACGAGGTCAAGAGATCAAGAGATCAAGACTATCCTGGCCAACATAGTGAAACAC

>ORI-32

CAGCCCACTCCCTCCCACCTGCGGCCGCCGGGCCGCCCTCCACCCACACCTCTGCCGCAGGCCGGACCCAGTGCGCCCGCCCGTCGGTCAGTCCAGGCCAGGCGCCCGGCGGGCCGCGCTCACGCAGTTGGCGCAGGAGGCCTTACGCTGGCGGCGCAGTGCCCGCCCCCTGCGCTCTCCCCGCCCCCTCCCTCCCTCGCAGGGGCCGAGCGAATGTAGCCCGCGAGAGAAAATGGCGGCGGCGGCGGGGAATCGCGCCTCGTCGTCGGGATTCCCGGGCGCCAGGGCTACGAGCCCTGA

>ORI-33

ACAGGGAATCATGACACTTTGTTTTTTTACGTGTAAATATTCGCAAAACGCCCAAACTGGTAACTTTGGGAACACGGGTTCACGCAGAAGAAGGGCTGCTGTCTGGGGAGGACTGAAACTCGGGTTCCCGAAGTCGGGCTGCTCTGGGGCCACGGAGAGAAGGGGAGAGAACGTCGAGGAAGAGGCAGAGCTCAAGTGGACGGAGGATGGAACACAGAGCGCGGCCGGGACAAAGGGGCAGGCCAGACAGCCGTGACAGAGAAACTAGGCGGTCCGCTCCTGGGACGCGGTCTCGGGGGC

>ORI-34

GGACCTTCCCCCTTCAGTGACCCGAGAAGGACCTACAGTGACACCTACTTTAGGTTTTGCGATCTCCTTGATCCTCTCGATTTCCTCATCAGACATGACATCGTAGTACCTGACGATGTGCGGGCTGTCCCACTCGTCCTCCTCTTTGAAGGGGGCAATGAGCAGCTGTGGGGCCCTGTTGCCATGGTGGTACCTACAGAAAAGCCTCTTCTGTCTACGGGGTGTCTGGAAAGCACAGAGTAACAGGCCCTGAGCTGAGTGAGTCCTAATCCTCGGTCCCAGTCTGGCTGTCTGCAGACA

>ORI-35

GGTGCCATGGCATCTGCATTGGCTGTTCAAGTACATCCCCGGAGGATAATGGTTGGGAATGGAGAGAAGACCTTGGAGAGAAAAGGGCCCAAAGTGAAGCGCTGCATATATTTCTTCCCTTTACACTGCACAGCCACGACCCCAGCCGCTCCAGCTGCTACTTTCTCAGGAGACCTGGGGAAACAAATCTCCTGGCAAAGGACGAGGGGAGGGGGCTGTGCAGACAGGGAGGTGGCCTGCGAGGAGCCTGCAAGCAGCAAGAGAAGGCCTGAATGTTCTGGGGAAGCAGGGACACCTTGC

>ORI-36

ACAAGCTCTACCATCCCGAGTGCTTCATGTGCAGTGACTGCGGCCTGAACCTCAAGCAGCGTGGTTACTTCTTTCTGGACGAGCGGCTCTACTGTGAGAGCCACGCCAAGGCGCGCGTGAAGCCGCCCGAGGGCTACGACGTGGTGGCGGTGTACCCCAATGCCAAGGTGGAACTCGTCTGAGCTGGGACCCTGCTCCCACGCCTGCTTCTTAAGGTCCCTGCTCGGCCGGTGTAAATATGTTTCACCCTGTCCCTCTAATAAAGCTCCTCTGCTCCACCTTGAACCTGTCACCTGGCCT

>ORI-37

GGTGCCACAGGAGTGTCTAACAGAGGAGGTCAGGGGCAGCATCCCTGAGAAGGTGAAGCATGAGCGAGAGTGGGAAGATGAGTCGAAAGTAGCCAGCTGAGGGGTAGAGAGGAGAAAGAACATCCAGGCAGGGAGAATAGCAAGTGCTAAAGCCGGGGCTCATGAAAAGGCATGGGAGCAGGACAAAGTCCGTGTGGTAGAGGTGCGGAGAGTGGTGTTAAGATGAAGGGGAGAGGCAGGCAGAGCCCTGGGCAGATGAGCAACCAGGGCTTAGTGGATCACAGTTAGGACTTTGGGCTT

>ORI-38

ACAAGAGCCAGGTTTTAGTTGGATTGTTAGTGTGAGTGATGAAGACCTCAGAATGGCTCTAGAAAGAAGCCCAGATCTCAGCTTTGCACTGTGATTGTCCCCCAGCAACTTTGCATTCCCCCTGTATGGGAATAGGGACCTGTGGATTCTGTTGCCATAGATCCAGAGATTGGACCTAGGAAAGTGAGACCAGGTGGTCTGACACCTACCTCCAGAGCGGACAACCCAGAGACAGAGCAGGCCCCTGCAGGTGTAGGGGCTGGCGGCGAGAAGACAGCTATGTTTACCCTTGTAGACTAG

>ORI-39

GAGCTTCATTTCTTATAAACACCGCCGTTGGGAGGAAAGAGAACTCAGCCCCCTGTCAGTCCCATGGGGCCCCGAGGCCGGGGGAGGCTCCTGGGCCAGGCCCAGCGGCCGCGACCTGGAGCGGGGCTTGATGGGATCAGCACTCTCCTCCCCCGCGCCAATGGGTGCTGCAGAACGGCCCACGCCCACTGCCGTTCGCCCGGGGCGCCCCTGGCAGGCTTCTCGGTCTTTGTGGGGGCTGCAGCACCTCCACTTCACCGTGATGTGGAGTCCTTCGCCCCCAGGTGACCCACGCTCCAC

>ORI-40

CAGAGCCTCATCTGAGGTAAAGGAGCAAAGTTGGGATTGGGGTCCAAAATTCACTTTAACTCCAAAGCCCACACACTTAACCACCCTGCCTATTTCTGTCCAAATGTCACCTGTCCTGAATGGAGTTTTTCCCCCTGTACAACTGTCATCAACCTGTTCGGGCCCTCTCACTGACAGGCAGGTCCCTACCTATATTTGAGGGGCAGCCCATTGCATTTCTGGACAGCTCTCGCCATTAGGGTGCACACACGCACCACCTCTGTGAACAGGGCTCTGGCTAGGCCACTCCTCAGCAGCTCT

>ORI-41

CAATCCTAGGTATATACCCAAGGGAACTAGGAACACATGTCCACACAAAAACCTCTACACAAATGTTCATAGCAACATTATTCATAATAGCAAAAAAGTAGAAGTGGCTGGGAGCAGTGGTTCACACCTGTAATCCCAGCACTTTGGGAGGCCGAGGCAGGAGGATCACTTGAGGCCAGGAGTTCAAGACCAGCCTGGGCAACAAGGCAAAACCCTGTCTCTACAAAAAACTAGCTGAGCATGGTGGCACATGCCTGTAGTCCCAGCTACTGGAGAGGCTGAGGTGGGAGGATGGCTCAA

>ORI-42

TTTAAATTTACTGTAGCAGGCAAGAAAAAACAAAAAACAGTAGGCAAAATTCAAAAGCTGTGTTAAGAATAGATGGTAAGTTGGTAACTTGAGAGACACGCTAGACATAGAATACATGAAACATATGGGTGTCAGGCTTAGATTTAAAAGGATAGAACACACAAGAGTATTACTTGGAGATAGAAAGGGATGGAAACCCCCAGTTATAAGGCAAGGATCATGCTAATTCCAGATGGACAACTGTCATCCTCAAATTGGTCCCCTTAGAGACTCCCACTTTGATCCCATCCTGGTCCCTCC

>ORI-43

GTTCCAATGATTCTCCTGCCTCAGCCTCCAGAGTAGCTAGGATTACAGGCGCGCACGACACCACACCCGGCTAATTTTTGTATTTTTAGCACAGATGGGGTTTCACCATGTTGGCCAGGCTGGTCTTGAACTCCTGACCTTGTGATCTGCCCACCTCGGCCTCTCAAAGTGCTGGGATTACAGGCGTGAGCTACCATGCCCGGCTTGTTTGTATTTTTATCTGCACTTTTAGCATTATAAACTTATGTAAGAAAATACACACAGGACAAAGAATGTCAATTCTTATAAACATAATAACTG

>ORI-44

CCTCCTTCCCACATCACAAACATTCTCAGAACCATCAGAAGACCCCAAAGCAAGGTCCTACAGTATGTGCATTTTATAAAGCAAACCCACCCTCTGGCTTCAGGCTGTGTGTGACCTGGTTTCTCTTTTTAATTTTATAACTAGGGTGGAAAGCTAACAGTGTAGTATGTCATGGTCAATATGTCAACCGCAAAGTGGGTCCAATGTTGCAGAAAGATTCCAATAGTCATTTAGTACATTTCCCTTGTTACTTGAGAGAATGGTAGTTCAATCACTAGGGTCTGGAGTCCCTAGTGTCAG

>ORI-45

AGGTTTCAGTTAGACGAAATGAATAGGTTCTAGAGATCTATCGTACAGCATGGTGACTATAGTCAGTAATGTGTGGTATACTTGAAGATTGCCAAGAGGGTAGATCTGACATGTTTTCACCACACACACACAAAACGATAATGTTGCGAGGTGATGGATATGTTAACCAGCTTGATCATAGTAATCATTTCACAATGGACACATAGATCAAAACATCACATCATATTCCCTAAATATGTGCAACTTTTATTTGTCAATAATACCTTAATAAAGCTGGAAAAAAATAGTTGTTGAGAGAAA

>ORI-46

TAGAGCTGGGTTGCTCGAAGTGTGGCTCCCCGACCAACCGTATCCCCGTCAACTGGGAAGTTGTTAGAAAAGCACATTCTGGGGTCCTAGCCCAGACCTGCTGAATCCGAAGCTCTGGGGGTGGAGCCCAGCAATCTCTGGTTTAACAAGACCCCTCCCCTCCCAGGTGATTTCCATGTGAGCTCTCGCTCGAGAACTGCTATTCTCTCCTCCAGAGCATCTTCCTGGTTATTCTCTGGGACGCAAAACCATGGTGGTATCTGATCTCATCACACTTCTGCCAGGAGGTGAAATCGAAGA

>ORI-47

GCTCACTGCAGCCTCAACCTCCTGGGCTCAAGTGATTCTCCCACCTTAGTCTCCTGAGTAGCTGGGACCACAGACATGCACCACCATGCTTATTGTTTTTTTTTTTTTAATTTTTGTAGAGATGAGGTCCCGATATGTTGCCTAGGCTGTCTCAAACTCCTGGGCTGAAGCCGTCCACCCGCCTGGGCCTTCCAGTGTATTGGGATTACAGGTGTGAACCATGGTGTCCAGCCCCTCTGTGCTTCTGTAGGACTCTGATTCTTTCCCTTCTACAGCATAGAGATATTGCTAACTGTACAC

>ORI-48

CAAGCACTTTACAAGTGTTAGTTATAATAACCCCATTTAATTGTTATAACAATCCTTTGAAGGTTTTTTTTTTTATTTCTTTTACAGATATAGGAAGCTAATGTACAGAGCGGTCATATAACTTGCCCAAGGTCATCCAGCTAACAGTTGACAGAGACGATACTTGAACTTCTGTGTGTCTGATGCTGAAACCCATGTGGGATAGTAGATTTTTTTCAATAAATATTGAAAGTTAAAGGATTTTTCTCATTAAAAGAATACCCCTCTGGGTTTGGTTGATATTAGTCTGTTGAGGTAGAC

>ORI-49

TATTTTTGTTTTTTGTAGAGATGAGGTCTTGCTGTGTTACCCAGGCTGATCTCGAACTCCAAACCTTAAGTGATCCTCCTGCCTTGCCTCTCAAAATGCTGGGATTACAGGCATGAGCAACCCTGCCTATAATTTATTCTGATATAGTTCAGCATTAGCAGATAAGAGATTCCACATCTACAGGAAGTTCCTTTGCATTTTTCTCCATATCCCTAAGATATGAGGAATGAAGTCCCCTGCTGACTTGAAGTCCCCTGCTGCCTGTACACTGATTTTCTGCTAGGAGGGCGTGTCTTCAGG

>ORI-50

TAAAGACCCTATTTCCAAGTGAAATCACCTTCAGAGGTATTAGGACTTCAACATATCTTTTTTTGGGGGACACAATTTAACCCAGTAAAGTAGGTCTGTTCTATGAATGGTGACAATCCAGAGTGATGCAGCCAGCATTAACTTGGGCAATTAGATTTTTGCTTTTTAAAATGAAAAAAATAAATTATGTTGTCAGCTAGGAAGTAGGAATAGGTTTTGCTTATGGTTATTCTTTTAGGATAACAAGGATAGAGAAGGAGGCACAGGGAGGCGGGAAAGATCTTTCCTATGTCAGGCTGT

>ORI-51

GGCAAATATAGCAAAATATGAACAACTGTAGAATGGAGGTGATGGGTATATGCATGTTTACTGTCTCTTCTTTCATCTTTTCTGTATGTTTGAGAATTTTTATAAGGGTATGTGGATTGGGAGTAAATGTGCTGTTCTGGCTGGCCTAAGTCACACTATTCCTGGAGTCGGAAGTGGAGTGGATTCTGCCTGTGATGGAGGTGGGGGACCCACACAGAGTGCAGAATTCTAGAATCTACAGTTCCATTTTAGAAACCTCATTCTGCTCCTTGGGAGTTGTGGGAGAATACTGAGATTCTC

>ORI-52

CTTCCAGGCTGCTGGGCACCTCCCCTGCCCATGGGTGAGTTTGCCACCCCACCCCAACACCCACTCTGTGCTCCCCTCTCCTTCCAGCATGCACCAGGGGAAAGATCATGGAGCTCAGAAAACCTAGTTCATATCCCAACCTTGCTTCTCAGTAGTTGTGTGGACTTGGGCAAGCTGCCGAGCCTCTCAGAGCCTGTTTCCCACTCTAAAATGTGGTCTCTAAAAGTCATAGACCTAGAAGCCAAACTGACCTCGTGATGTATTAGTGAGGACCCAAGGACAAAATGTCTGGGAGGCCAA

>ORI-53

ACAAATTTGAGTGTCCTTCACCAAGTTGCCCAACCTCTTATTACTCAACAACCTCAAGCTGCTTCAGTTTCCCCATCTACTAATGAATCTTCCGGTACCTTCTGCCAAGAGTCAGAGAACCAGAGACTCCCAGACCCCATTTCTAGCCAACCACTTTCTCCAACCAGACCTGCCTACACAGGGAGCCGGCTCCCTAATGTTCAATTTCTGGACCCCTGGGTCCTCCCCTCCCCTCCCCACTGGCCCCCACCCATATCCTCACAGGGGCGCCCAGCTCCAAGCCTGTGTTTGTGAGCTCAC

>ORI-54

CCCACCTGCACGGCACCCCTTGTGCTTCTGCCAGGTTGATGCCCCTTCTGGCCGCTCCCTTACCCCCTCCACTTCCTGGGGACTCTGTTCTGGATTAGAATTTTAAAGCGAATAAATTCTGGGGTTGAAATAAAAGCTGTCTACAGAGAGCCTGCTCACATTTCTCCTTCTCCCTCTCTTTTGCCTGCTTCGGGAAGTGGGAATGCTGGAAAGGCGTGGTCTCAGGTCCTCCACAGTCTGCATGCACGCATGTGCCCCTCGGCCCTCACAGGCAGTCTTTTTCTATCTGCTGCCCCTCCC

>ORI-55

ATATACTAAATGCCCTCATAAGTGCCCTTTGGAGTGAGTATGGAGGGTGCTCCAGATACGAGGCTTCCATGGGCCTAGAGGATGGACTCAGGAGGTTTGAGACTGCATTTGGATTGCGGCCAGAACTCAGGGCCCCATCCCAAGAAGTTTGAGGCCCAGCCAGACCACCATGAGAGCCTGCAGAGGACTGGGGTTCAGGGTACTGGGGGCCTCTTTTAGGAGTGCAGGCTGTTCCTGTGGACTCTGCCAGGGCTCAAAGCTGCGGACTGGGGGGTGGGCTGGATCAGAGTGAACAGGAGC

>ORI-56

CGCGTGCACACATACACATACACACACACACACACACACACGCGCACATCTCTGTGAAAAGATGAGGCACAGAGAAGAATCATTCCCAGTCATGACTGGATGGCACCAATAATAAAGGCTGACACGTCCAAAGCTCTAAGACTGTGCTCCTCACTTTGCTAAGCACGTTCGACACTTGCTTCATTTATTTATTTTTATTTGGCAGGTGAGAAGTAGGCATCATAATTATACCTATTACATAGAGTAAGAAACTGAGGCTGAGAGATTAACCTGTCAAGGTCCCTTAGCTAAGTGGTAAAG

>ORI-57

GGAGAACACCCCTACACATGCACTCATTCCTTTGTTTAACACATTGCCTTAGAGCACTCATCACCTGCCCTGCACTGTACTAGGCACTAAAGGGGGACCCGCGATGGGGCAGCAGCCCCCTCTGCAAAGAGCTGGAGCACTCGTTGGCAAAACAAAGCACTTACATGTGAAAGGTGAATAAATATCCAAGCCTCCTCCATCCCCTGCATCATCCCCAGACCCTATCAGAGGGGCAACACGCTGACCCTTGTCCCCACCTGCCCTGAAGGAGGCTGGCAGCTAGACCTTTCAGGTCTAGCA

>ORI-58

GGTGAAAGTTGCTGCACCTGCTTTAGGCGAATTATGGTAGCACTAGAAGTTGGTCTCCCCTCCCTCCGCCGCTTGCTCTCTCGCTCCCTCCTCCCTCGCTCTCCGCGCGCTCCCTCCTTCCCTCTCCACCCTCCTCCTCCCCCGCCCCCTCCCGCCCCGCCCGCGCGCCGCCCGCTCGCAGCCACTCGCAGCCCAGGCTCAGTCGCAGCCCCTCGGAGTCCGGAGCCCGCCGTCCCTGCGGACCGGACAGTGCGGCCGGCGGCCGGCGGCCGGGACGGAGCCCCCAGCCCGCGAGGAGGG

>ORI-59

ACAGCTCTTCTCAGTGACTTCTACTACCCTTACCCCAAGCCCTTATGGACCCATGACCCTCTCACCCCCAAGACCTTCCACCTTACCCTGATCTCCTTCTCCTTACTGCCCCAAACACTCCCTAATCCCTGGATCCCACCCAGCCTCCCAGGCCATTGCTGACTCAGCCCCTGTCTTGCAGGCTCTCCAAGTGGCCCGGCAGTTCCTGCTGCAGCAGGCCTCAGGCCTGAGCTCCCCAGGGAACAATGACAGCAAACAGTCTGCCTCTGCTGTGCAGGTGAGGAAGAGAGCACCCCGCTG

>ORI-60

TGTAGGTGACTGTCAGGAGAGGCTGCCCAGAGAGCAGAGACACTTTGGGTAAGGCAGGTGGAGATGTGAACTCCAGGCTCCTTAAACCCTCTTCCTTGCCTTCCTCCCTCTCTAACACTGCGGGGAGGAGGGGGAGCAGGGCAGGGAGCAGAAGTCCTGGCTCTCTCTACCATAGGACTCACAGTGTCACCTCTGTGAGCCCTGGTGACCTCAGCAGGAAGCCTGGGAAATGACCACAAGGCCTGTCATGCCCTGCTATCATTCTAGGCTTCCCAGCCTCAACACCACCCCGCAGCATGT

>ORI-61

AGCCCTAATGGGACAAAAACACCAATGAAGAAAGTAAAATCAAGGGCAAAGGGAGCACAAATCCCAAGCCCCACGGGCCCTGAGAGGGACAGACACCAAGAACATCAATAAGTTTGAAGTCTAAGACGACCAAAGACCTTTTAAGTCACTTTAGATCTCCCTCTGAGTCCTGTTACAACAAACACCAAAACCATCACCTTACCTTTGCAGAGAACTTTAACTCTACAAAGAGCTTTCTCAAAAGGACACTGTGATATAGTAGAGACAGCACTGGTTTAAGACTCAGGAAACCAATATATC

>ORI-62

CGCGGCGCGGCCTCGGTGAAGCCGCCTCGTCCCCGCCCCGCCGCGCCCCGACTCACATCGCCCCGCGGCCCGGGCGGCGCCGGGCCCCGCTCCCGGGTCCCGCTGCAGCAGCCGCCGCCCGCCCGGAGCTAAGGCCTCCCCGCCCCGCCCCGCCCCGCGGCCGCGGGGTCGGAGGTCACCGGCAGCACCAACGAGACGCTGCGGGCTGGCGGACCCGAGCGGGGGCGACCGGCACGCAGCCTAGAAGGTCCCTTTGGAGGACTTTGCAGTCGGGAGCAGTGAGCATGCGCAGACGCCAGA

>ORI-63

TGGAAGGTCTCCTGAATAGTCCATACTTGCCTCATTTTAGGTGTCTGAGTACCTTTCTCAGAAAAACATCATTTCCTATAGAAGTAGATGTTTAAAATATTGAAAAATATTATTAATAAAATAGTTTAAAACTCACTCCATATTACGCTAGATAAGTTTGTATAATGATCAGGTGACCTGATAGCCACATAAAAAGTAGTGCCTTAGTTTCGCACAAACTGAAAGGGGTATGGGGTGTATAAGTGTCATTTACCACAGTGAGAGCAGGAAAATTAATTCTTCAACATGTGAACATTGCTT

>ORI-64

GGGCATAACAATAGTGATGTTGTGTCTATTAATGGAGACATGGAAGAGGAAGTGCTTAGTTAATTGTACAGCTATAGAGGAACATGGTTAGCAATTACTATTATTGCACATTTGACTTTAAGAGTGAATGCTAGGCCAGACTCAGAGGCTCACACCTGTAATCCCAGGACTTTGGGAGGCCAAAGCAGGCAGATCATTTGAGGTAAGGAGTTTGACACCAGCCTGGCCAGCATGGTGAAACCCCATCTCTACTAAAAATACAAAAATTAGCTGGGCCTGGCAACCTATGCCTAGTCCCAA

>ORI-65

TAGCCTGGCATGGTGGTGCACACCTGTAGTCCCAACTATTCGGGAGGCTGTGGTGGGAGTATCACTTGAGTCCAACAGGTCGAATCTGCAGTGAGTGATCGCACCACTGCACTCCAGCCTAGGCGACAGAGGTCAAAAACAAAACAAAAGAAAACAAACAAAAAAAACATAGCAAACATCCAGAAGACTGTGTAGAGCAATGAATGTCTCTCAAGCCAGTGATCTTTTTCTCTTCATTTTTCACATCAGGTGGGTAGTGTGCCCACTTCGTAACAAGGTTTGAGGGAGGCACATTTGACA

>ORI-66

GGGCGATACACGGCGGCGCGAGGCCAGGCACCAGAGCAGGCCGGCCAGCTTGAGACTACCCCCGTCCGATTCTCGGTGGCCGCGCTCGCAGGCCCCGCCTCGCCGAACATGTGCGCTGGGACGCACGGGCCCCGTCGCCGCCCGCGGCCCCAAAAACCGAAATACCAGTGTGCAGATCTTGGCCCGCATTTACAAGACTATCTTGCCAGAAAAAAAGCGTCGCAGCAGGTCATCAAAAATTTTAAATGGCTAGAGACTTATCGAAAGCAGCGAGACAGGCGCGAAGGTGCCACCAGATTC

>ORI-67

CTACCATAAATAACCCATTAGGCCCTCGCAGTTACATCAGCTTTAAGAATACCATCTGCCATATGTCCTAGTAGAGTGCAACAAATGGATGCAAACATTACCCAAACATCAAGTGAAATATGAAAAGAATCTGTTATTCTGTGCTTTAATTCGATGTTGTTTAATGTGAAAAGACTTAGAACCAAAAGTAAATCGACCCACTACCTCAAAATTCCCTACAAGATTAATTAAACGAGACTTAATCCTTCATACTTGCACTTTTTCCGTCACAAACAGGAAACTCCGAAGAAAAGGAAATAG

>ORI-68

TTTTTTACGCTTGACTTGGTTATTGTTGCTTTTCTTTTCCCGTGATTCCTTCTGAGGATTAGCTCTGACTTCCCCACTATTGGCGGTCAAAGTGGCCCCGACTCGGGATGACAATTGACGGGGATCAAGGGATTGCCCATTCTGTGCCTGTAAGAACCGATTCGTGCCAGAGAAACTCATCAAGTGGAGGCGGAGAATAAAGACCGTTCGGGGGTAAATGTATTCATTGAGAAGGGTTCTCCCAAATTGCTTCTAAGTTTTTTTAATGAAAAGTAACGATTGCGGCAATCAGCGGCTTTT

>ORI-69

GTTGCACGTAGAAGGCTGCAGTTGGAGACGGGACAACTAGGTTCTGGGAAGCCATGGCAGAAATCACGCAGACTCTGATTCCCGCCGAGAACCAGCCTAAAGAACCACTCATGGCAAGACGGGGGCCCACCGGCCTGCTGGCTCCTCACCAAACTCTACTCCGCATTGACTGTGGGCTCTTGTTCTAGAGGCACCCGCATCCTCAGGATGAAATGTGAGTACAAGAGCAGAGACTGCCGGTGCTGGTGTTTGCAGGTATGTGTGTGTGTGTGTGTGTGCACGTGCGTGTCCACAGCAATT

>ORI-70

CCCAGCCCCCTTGAGGATCTTAAAATAAATGTATTCATTAAGGCTGAATATCAATAGTAGTATGCAGTATATAATGTACATGTAATGATGGATACTAAATACATATTGCAGTATTAAACCTGGAACATTCTTTTTTTAATTTTTTTGAGACAGAGTCTCGCTCTGTCACCCAGGCTGGAGTGCAATGGCACGATCTCGGCTCAAGCAATTCTCCTGCCTCAGCCTCATGAGTAGCTGGGATTACAGGCACATGCCACCACGCCCAGCTAAGTTTTGTATTTTTAGTAGAGATGGAGTTTC

>ORI-71

TGAACTTTATTGCTCATGGAATAGAAAAACCATTGAAATTTGTGGAGAGAAAGTGGCTGAATTAGATCTGAGTTCACAGTAACACTGGAATCATCTACCTGTGCCATTGCTTCCTCATTCATGTGCCCAGCTAGTTCTTTCTTTCCAGGTGGGAAGGAGAGAAACTTGAAGCCACTTTCTGTATTCCCAATCAGATGAAGTTAAAATTAGCACTGTTATTTCTGTTTGGCTTCCAGGCAGACTGCAGGCCATCACTGGCTAACTTGTTATTGACACTTGTAGCCTCCCTTGGTGGTACCG

>ORI-72

TATAAATACGAACGGATGGGGATAAATAATATTTAAAACTTAGATTATTCAATGCTTGCAAAAAAATATAAATAAATCTGACTGTTCACCAGCATACACACACGGAAAGACGTACACTTAGTCATCCTTGCACAGAGAGCCCCTGTTTCAAAGCGCCTTTGATTTTTTTTCTCACTCTTTACAAGAAGTGCAATTAAAAAAAAAATTTAAAATTAAAAAAAGTAATCGCTTCCCCTCGGGAGCCATAATGTAAGAACAAATTCACATTGAAAACTCGACTAGAAAATTTGTTCATATACC

>ORI-73

CTGGAAGTCCCAGATGCTCAGGCTCAGAGCAGGGCACTGGCAACTGGAGAAGGCAGGGGCAAAGGTAACCAAAATGCTAGGAAGCCTAAAGCCTGGGGCCTTTCCTCTACTGGAGCCCGCTTTCAGGAGGCCCATGTGGTTCCCATTAGGATGATAAGTCTGCATCCCCCTCCCCCAGGAGATCTTTGGCTGCTTTCATTGTACTAAGTCTGAGTAGGGTCTTCCCAGAGGTAAGGCAGCCACTCCATCTGCTGGAGCAAAAATTGGCTCTGATTATAGGCAAAGCATTGATTCTGTCTT

>ORI-74

GGACAATAATCCCCAGGTGCAAGAGCCGAAAGAGAGTTGAAGAGGCTAAATTGAGTGCAAGAAGCAAGCCCTATTGTCTCTCTGGAAGATGTGCCCAAATTCAACCTCCGTCCTTCAGACATCGCTCCCTCTTCCCATTCCCTCCTCTTTCTTAGCCTTCCCTCACCCCAAATCTGAGCGGGTTCGTCGGATAGGAGCCCCGAGTCCCCTGCCCACCTCTCCTTCCCTCTGCAACCCAGAAAAGTTAAGGCTGGGCTAGGGGAGAGGCAAGAGGTGGGGGCGGGGATGGGAAAGCGGCCT

>ORI-75

CAAGCTTGACAAGAGTTCAGCTCAAGTTGAACATACATACACACACTCTCACACACAAATTATGTGAGCCGTCAGAATCCAAGTGAATCCAGCTCAAGCTATCTACAAGGTTTTTACATGCAAGGTCAAGTATCTCAATCCAGAGGACTTTTGTTTTCTTAATGAAAAGCTTAGAAAACACATGAATCTTAGATTTTTAATGTTTTTTAAATGGAGTTTATTCTTAGCACATGGCTTTCTATGTAGCCACATCACAATTTGTACAGTTCCACATAAGTCTAAATGCACTCCCCTCTCCCC

>ORI-76

GGCGGCCGCCCGGGGCTGGCGCCGCCGCGGTAGCCATAGGGGTAGGCGGTGTCCGCGGCCCCATGCGCGGGGTACAGCGCGGCAGCAGGGTAGGCGGGCTCGCGGGCGGTCCGCGGCGCGTAGTAGGAGGCAGTGGGCTCTCGGCCGCCGCCCGCGTGAGGGAGCTGGGGCTGCTGCAGCGGCAGGTGCTGGGTCGGGGGCGCTGGGGGCTGCTGGTAGCCGGGGCCCCCGCCCGGGCCGCCGTCTGCGCCGCCCGAGCCGCTGTGCTGCGCGTACTCCTCGAAGGGAGGGAACTTGGGC

>ORI-77

TAATTCTACAAAGAAGGAGAGGAACAGGAAAGAAGAGAGGGAGAAGAAAGGCAAAGCGGAAGAAAAGAAAGCGCTTTAACCCCTTTCAATTAGCCTGGGGATTCAAAGACTAAAGTTAAATCCGGCCATAAAGTTTATTGCTTCAGACTCACAAGCGGCTGAGAACAGTCCCGCCGAAATAAAAAGAACATGCAGGCAAACAGGGTTCAGGGCCTGGTCCCGGGTGCGGGGGAGGGGGTCCTGAACACCCCCCCACACCAGGGTGGGGATCCTTGGTCCTCAGGGTCCAGTGGGCGCTAG

>ORI-78

CTCCAGCCATACTCACCTCCCCTTTCCCAGTCCCCTCTGGATAAGGCAGTCCACATTTTTCTTTGTCACCACGCATCTTTATTTTCGGTTACATAAAACACAGCTGGGCTGGGAAGTGTGCCTTCCCTGAACCCCAGGATGGAGCTGAGCAGGGTACAGGACAACACAGGAGATGAAGGGCATTGCGGAGGGCATTGGACCTCCCCACCCACTACAGTTAACTCAAGACAACATACCATGCTACAAAGTCACCCCATTAACACATCCTTTCCAAGTCAAGACACTGCCTTACAAATGAAC

>ORI-79

TGCGGAGAAAGACACGAGGCTCCTGAGCAGGGAAAGCCGAGGTTGCCACCGCAGGCCTGGCACGACCAGGGCCGTGATGCCCCGCCCGGCCCGACCCCCGCGCGCAGAGGTACCTGGAGACGATTTCAACTGAAGTAATGAAGGCAGTGTCGTGCTGTCGAGAGAAAGGTGGATCCCAACAACAGGAAACTACCTAAATCACCGACCAGTTCTGGTGCTGCCCGCGAAGGGCTGCCTCGCCCGCCGCCGCCGCCGCCTCCGCCGCTGCCGCCGCCGCCAAGGAGAGAACCCTGCCATCGC

>ORI-80

TAATTTTTGTCTCTCTCTCTCTGTCTCTCTTGCTCCCATAGAACCGTGCCCTCACAGCAATCCAGTGTTTGATTCTCCCTATAGAAAGTGAATCTGATCAGGGCCTTCTTCACTCTCCTCCTCCTTCTCTTCTGTTCTGCAGCTCATTCAAAAGAGCCCATAGAAACACAGAGAGACCCATGAGGAGTCTCTTGCCACTGGGACTGACTCTGCCTGGGACCCAGAGACCAGAAATGGTGAGTGGTTTTTCTTCAAAGGCTGCTGGGTCCTGAGGCTGGGATTGCCTGCACAGCGAGAGGG

>ORI-81

ATGGTGAAACAGAGGCCTAGAAAGCTTAAGTAACTTGCCCAAGGTCATCCAGCTACTAAGGGACAACACTAGGATTGGACTGAGGCATTGGATTTAGGGTCCATATTTTCTATTTTCTTTCTTTCTTTCCTTTTTTTTTTTTTTCTTTCTCAATACAGAGTTTCACTCTTGTTGCCTAGGCTGGAGTGCAATGGTGCGATCTTGGCTCACTGCAACCTCCGCCTCCCGGATTCAAGCAATTCTCCTGCCTCAGCCTCCCGAGTAGCTGGGATTACAGGCATGCGACACCATGCCTGGCTA

>ORI-82

CTCCCCGCCTCAGCCTCCCGAGTAGCTGGGATTACAGGTGCCCACTACCATGCCTGCCTAATTTTTGCATTTTTAATAGAGACAGGGTTTCACCATGTTGGCCAGGCTGGTCTTGAATGCCTGAGCTCAGGTGATCTGCCCGCCTCGGCCTCCCAAAGTGCTGGGATTACCGCCGTGAGCCACCATGCCTGGCCTTCTCCATTTTAGAGCCAGCAAGCCAATCATATTTCAAGGCCTCTGGTCCCCTTTTGTGAAAGAAATCTCTAGGTAGGGCAAGTTTTCCACCCTCCTCCAGCTCCT

>ORI-83

GGCGCCGAGCCCCAGGTAGCTGGGGCGGCCATGCTCGGCCCAGGACCCCCGGCCCCCTCAGTCGACAGCCTCTCCGGACAGGGGCAACCCAGTAGCTCGGACACCGAGTCGGATTTCTATGAAGAAATCGAGGTGAGCTGCACCCCGGACTGCGCCACCGGGAACGCCGAGTACCAGCACAGCAAAGGTAGCCACCGTGCCCCTCCGCTCCCCGGGCCTCCCACTGCGCCCACCCTTCACTTCGGCGCAGGCCAGGAGGAAGACACTCCCTTCCCCTAGGGCAGGATGGCTGGGGGGACC

>ORI-84

ACGAACTCAAACAAATTTACAAGAAAAAAACAAACAACCCCATCAAAAAGTGGGTGAAGGACATGAACAGACACTTATCAAAAGAAGACATTTATGCAGTCAAAAAACATATGAAAAAATGCTCACCATCACTGGCCATCAGAGAAATGCAAATCAAAACCACAATGAGATACCATCTCACACTAGTTAGAATGGCCATCATTAAAAAGTTAGGCAACAACAGGTGCTGGAGAGGATGTGGAGAAATAGGAACACTTTTACACTGTTGGTGGGACTGTAAACTAGTTCAACCATTGTGGA

>ORI-85

TCTCATTCAATCCTCACAACAGTCCTATTTTACAGATGAGAAAAGTGAGACTTCAAGGGATAACTCGCTCTAGATTCAATAAACTGACTCAAAAGCCTTTTAACATTCTGTACAATACCTTTTATGTTTGTTTATTTAACTGTCCAGTTGGGCCACTAAAGTACAGATAAAAGCTGGGGAATGTAAGCGAGACTGGTCACTTCCCTGGGGTGAGAGAAACGCTTTCAACACTATCCGTCCATCGTATCACGCCTAACGTATCCCATAGACATCCCTCACCCCTCCACCCACTCATGCCTG

>ORI-86

TATGGAAAATGTAAATATAGATTATGAGTAAAGTTATTAGTAAATAGGGAAAACAATAGTTTAGGAATCTGGGATTTTGAATTGTTATTTTCATTCCATGTTGTGAACTTCTCATAGACATATTCTATGTAAACATTATATCTTCAAATTTTCTCTCTATAAAAATTTTGATAATCCCTACTGTAGTGATTCCAGCGTCTTCTTTTTTAAAGTGATTATCATACTTTTCCAGTTTCGAAACAATAAAATAGTACCAGAATTTTGGTGGCCATTTTCTAATTGTGAGGAACTTAGGACATG

>ORI-87

ATAACCTCCAGATGTAGAGATAACTTTCCCAGGGCCACCCAATCAATACACAGCAAAGCCCCATACAAATCTGGGCTGTTTGGTTCTAGACTTTGAGCTCTTTTTCTACTTTTTGCCTAGATAAACACTTTGAGGGGAAAAAAAATCCAGATTTAAGAATTTTTGCTAACCTTCCTACGGGCAAGTCACCTTCAAGTCACCTGGGTACTTAGGTATCCAACTCAAAACTACTTTTAGTTTTGTTTCGTTTTGCCTTTTTTTTTTCTTTAACCGATAACCCAATTCTAGACGGAACATAAG

>ORI-88

GCACCCTACATCTGAATGCATCCCTAAGGCCTTACCGCACCCAGTCCAGGAGGCAGTCCTGGCAGCTGCCCTCCACCGAACTCCGCGCTTTTTCACACACGCTCGTGGAAGGAGAAAACCGCTCAAACAACTGGACTCGGCCCGTTTCCTTTCGGTAACCTCCCCACAGCCCAACACGCTGTCCCCAGACGCTGCCCGCTCCCACCCCGGTCCCTTCGTGATCCTCCCGCGCGCGGGCGGGGCGGGGGAGGAGCGCGGGGGCGGCCGGGAGGGGCGCGGCCGGGAGGGGGCGCCGCCGGG

>ORI-89

CCAACTTTATTGTATACACACCAAACAACAGAGTGTCAAAATACGTGAGGCAAAAATTGATAGAACTGAAAGGTGAAATGGACAAATCCACTATGGTGGTTGTGACTTCAACACCACTTTGTCGGTAATTGACAGATTAAGCAGGCAGAGATATCACTAAGGATTCGATGATCTGATCAGTACTGCCAAAGTAAACAAAGTGGAAGCTGCCAATATTTCAAGGCCTGGACCCAGAACTGGCACAGTGCTCCTTTCACCTACTCTTGATCAAGTGTTCAAAGAGACCAGATCCAAGCGTAG

>ORI-90

AACCAAGCACTGACTAGTAATATCCAAACTCTTTTCCTATTGCCATCAAAATGGAACTAAACCAGAGTGCAGTTTGTGTTTCAATATAATTTAAGCAGTAATGAAAATGGCCATATCAACAAACATATGAAAAAAAGCTCATCATCACTGGTCATTATATAAATGTAAATCAAAACCACTATGAGATATCATCTCATGCCAGTTAGAATGGCAATCATTAAAAAGTCAGGGAACAACAGATGCTGGTGAGGCTGTGGAGAAATAGGAACACTTTTACACTATTGGTGGAGTGTAAATTAG

>ORI-91

TCTGTATTTCTTCAAACACGGGGATGGGGACATTTTTTGGCTTTTCATTTACTTTTATACATTAAGCACTATAAATCTATACACTTAGAAGGGCCCAATTAACCGGTCAGTTCATTGTATATTGTTCATGCATAGAGCTAAACTTCTGGTTGGGAGATCCTCAGATGTCAGTATCTGAAGGCTTTTTCTTTGTTCCTCCTCATATGCTGATTACAGGCAGCTGACATCCTCAAAGCAGAGTGGAAAGGGGAGCTGATGGTCACATAGATCAGTAAACATGTTTTTCACACTTGATTTCCC

>ORI-92

TCAAGAAATGTATGACTCTATTATAATCAATGGAATCTACAAATATTTATTGAGCACATGCCACGCACTGTTCAAGGCAGTGGAGATACACCAGTAAAACAAGAAATTCCTACTCTTATAAAACTTCAGTTGTATTAGAAGGAGACTGGTGATAACAAGAGGTAGACTAGATAGATAGATAGGTTAAATGGTTTCAGAAACTGTTACATGATTTTAAAAAAAAAGCTGGATGAGGAGTTAAGGAATGCTAGAAGGGAGGGTGGGGAGGGGCAGGGGTTGATAGTATGGTTGGAAACTTAG

>ORI-93

GTCTTCACAGTTTGGGCTTGTCTGTGCCTGTCCTTTTCAGGAAGGCTTTCCACATAGACAAACCCAGGCCTAATAATGCTGTGGTTTTTGCAGACTCGAAGAGGTACTGCTTTGGTGGTCTTGGATAAGGTCTGGAAGAAGTGTCTGGATTGCTAGGCAAAGACTCTTATTCTTTTCTCCTACTTTTTCCCAAACATGGAGTCTCTTTCTCTGTGATGAGCCACCTGGAACTGAGGGTGTGGTGATGCAAGCACCCCTGTGGCCATCACCACTGGTATTGCTCTGGGTCAGACCTGAAGC

>ORI-94

AGAGCTTGAGTTTCTCAAGTGTTGGGATAGGGACTTGCTACAGCCTTCAATTGCAGGGAAGAAAAGGGAATCTGACACTAAATGTGGGGCAAAGGTGAAGAGGAGACTATGTAAAGGATTGAGGCCAGTAGTGAGGAGGGAGGGAGCCAGGAGAGGGTATATCAGTCTTTTCTAGAAGGCCCTTGTCGTTAGCCAGAGAGGCCAAAAATAACTCCCACACTTGGCCACTCTGGTGAATTTTGCTCTACCCTGCTATAAAAGAACTTCGTATTCTCATTTGACCCAGCCATCCCATTACTG

>ORI-95

ATGTGTGTGTGTGAACAGTAGTGAGAACTACTGGCTTCTCTTTTAGACAGGTGAGGTATGAAGTACCTAAAGGAATACTCGGATGTGAGTAGAGTAACATATATGCCTCAAGATCAGTAGCAAGATCTATGCAGGAGAAAAAAACTTGGAAGTCATTATCAAATACAGTGCTCCAAATCCAGTGCTCATTGCCCCATACCAGGAGGAAGCAATTTAAATAAACAGCACTTTTGTGCAGGAAGTGCCAAATTGTGCCATGTTGACAATTGCATTTTTTTAAAAAATGACTTTTAGAGTTAG

>ORI-96

GAACACAAAGGCTTCAAACTGAGATACAATAAATTTTTTGTCCAAAATTAAATCCCAAATCAAGAAAACAACCTTTAGATCTGTTGTTTTAGCAACTAATAATCTGCTGTGCACATTTCAGTTGGTATTTGGAAAACATTTAACTCTGTAGCTTTGTAGAAATGATATTCCCACTTGAACATAGAGGAAATTGTACTTATTGAGAGTTTAACTCAAGAGTTAGTGCACAACAAAAGTTCATTAGTGCCCATTTCATGTTCTGGAAAGATAAAATATCAGCATCCTATAGAGATGTCTTAT

>ORI-97

AGCGAAACTCTGCCACACACACACAAACACACACACACACACACACACGGTGTAGTTTAGGAAGTAAAAAAAAAAAAAAAAAAAAAATCAGATCTCCCCTCACACCTCAGATCTGAAGGCACAAACTCTAGGGCCAGGGCGTTCGCCTACCCAACTCCACATGCACTTGCAGGTCACCTAGCACTCAGGTACCTAGCACTCAGGTACATTGTGGCTCCTTACCTCTCACGACAGCAGCAACAACGTTGATTGGAAGTTTATCACTGTGTGTTACGGGCCATGGGCCATGTGTGTTAGAAT

>ORI-98

AAAATATAAAACATAGTTAATAACATAAGGCATAAAAGAATGATAAATTTATGTTAGCTCAACCATTTACTAAGGGTCAAGGATGTCCTCACTGTTTAAAAAAACTTAAACGCTAGTAGGAAAGACAGAAATACAGCATCTATTTATAATACCTTTTGATACATGTGAAAATTAAGAAGTTTGTTTCAGTACTTTAGAAATACAATGAGGGAATATCCGATCTAGCCTGGTCCAGGAAAAGGGAAGAAGACTGAGGCAGCAGGAAAGGCTATCAGGAAGTAAACCCTCAGGGAAGTGTGA

>ORI-99

TTTTGAAAGATCTTTAGTCTGCTAATCATGAATGGCCAACATAATTACAGGCATGCCAACATTTGTAACATTGTGACACTTTCCCTGCCATTCTTAGTTAAAACTGATCTTTTGTTCCAAAAATTTTTGCTACCAACAATAGCCTGTCCTTTATAGTTCTTTTATACTTTTGTGTCTTCTCTCTAACTAAATAATCAACTCTTTCAGCATTCCATCCATTTCCCTTTCTCCTCCCTCTTACTCCCAACCCACATTCCCCTCTCCATTTTAATTTTAACCTGTGCCCCTTCAAGTGTACTC

>ORI-100

TTACCCGAGTCCTGGGGACAGTCCCCGGGACTCTCCGCCAGGCGCCCAGACCGGCAGGTCCCGCAGGCGGCGCGCGGTGTGTTTGCACTTTCCAAAGTTCTTGAACCATCTCAAGAACTCCTTCTGCATCTTGGCGTCTGGCAGGGGTGTTCCGAGAGAGGTAGACCTCCCCTCCCCAAACTGCCACCATCACTTCCAACGCCCTCCACGCGCTGGAGCTCTGCCCGGGTGTGGAAACCTCGTCTTCCAACACGTAGCTGCCCTTCAGCCACCCGCCCGCAGCCTGGGAGTGCCCTGAGG

>ORI-101

CCTGTCTTTCCAGCCTCGGTGACACAGGTCTATTCTGCCTGAGACACTTACTATGACACCCTTGCTTGTTCCTGGGGCTTTGACACATTTCCAACGTCCCATTGTTCTTCCTCTCCAAATCAGCCAATTGCCCAAGCCCTGCTCAAATCTCCCACCTCATGAAGCCTTCTTGATGCCTCCCAGCACACCATGATCTAATTTCCTGAAGTAATTATGCTAATTGGGCATTTGAAGAATTGTTAACCGATTATCAACTAACTGCCCCTTAACATTGCATGTGTAGTTGTCTTCAAAGGCAGT

>ORI-102

AATGTTTATAACTTGTGCACCTTAAGTTTTAAATACTGAATAACAGGCCCATTCATGTTTCCTCATAGAAATACTGTCTATATGCATACAATGAAATGTTAAAATAATGTTCTTTTTACTAAATAGCTTTTTGACTTGGTAATGAACAATATGTTGTTTTTCCCTGAGAAGTAGTTCTAGGACTTTAGTTCTGAAGATTATGTTGTATTTTATACATTTTCAGCCTCCTGTTTTTCAGTTCCCAGTGATCTTACATTAAACATTTGTCTGTCTAAAACAATAGGTTAACTATAGCCAAAT

>ORI-103

TTTAAATTTTGGGAGGCTTTCTTCTTCTTTTACAAGTAATGTAAAATTGTAGTGAAGCTATTGGAAAAGAAAAGGATAGAAACATGTTAGTGCTTTGACACAGCGGGAGAGAATTTTGGAAGAGATATTCTACCAACTACAGATGGAATCTTCATCATCATGTAGACTTCAGATATTCTTTTAGAAAACTTTACATTTACTTATAATCTAAACCTTACTTGTTTAAACAAGTCATGAAATGTATAGCTTAATAATTGCCTTTAAGAAAATTGTTGCCCAAAACAGAAACCGTATTGAGTA

>ORI-104

ATATCAAGAGCAATCTTTCAATTATGGAGAGGGTCGTGATGTGTATTTTAATGAAAGCCAACTATCTTCTCTAATATTTGAAAAAACAATGTTTAAAAGGCTTCTATTTGTACACCTTTTCACATTTTAGCTGGCAGAGTTCAGGACTTGATGGAAACATAACGAAGGGCTCAGAATGTACTCATCGCAGACTGAGCCCATATGGTCACAGTGGGGAGAGCACGCTGCACTCAAGATGCTAGCAAGCCTGCAGCAGGCGGCCCAGCGAGACACGGTCCCCCGCCCGGCCACCAGGCGCCC

>ORI-105

GCAGCGCCTGGCGTGTGGTAAACGCTCAACTTATGCGGGTTGTAACTGCCTGAGGCGTTTCCTTCGGCGCGCCACTTGGCCTTCTCTTTCTAAATATTAGCATAATAAATATAAATGGACCCAGAACGCGAGGGGGCAGGGGCCACGTGATCGCTCCACGAAAGCCACTTGTAGGGACCTTTAGAGAGTTAGCAATAAGGCTGGAGAAGGAAAAGCCGGCGCGCCCGCGGCCCGCGCGATTTCCCGCCGAGCCCGCGTCCCCGGCGGCGGGCGGGCTCCGGGGGCGCCCGGGCCGCGGCG

>ORI-106

AATCTACTTTCTGTCTCTGTGGATTTGCTTAGTCTAGAAATTTTATATAAATGGAAGTATAAAACATTTGGCCTTTTGTGAATGGCTTCTTTCATTTAGCCTAATGTTTTCAAGGTCCATCCATTTTGTAACATGTATCAGTGCTCCGTTGCTATTACTATTATTTTATTATATGGATATACCATGTTTTATTTATCCCTTCTTCACTTGGTGAACATTTGGGTTGTTTCTACTTTTTGGGCTTATGAACGATACTTCTCTGAACATTTTTATACAAGTTTTTGCATGGACATGTGTTTT

>ORI-107

CTATTTATTTTTGCTTATGTTACCTGTGCTTTTGAGGTCTTATTCATAAAATATTTTCCCAGACCAATGTCCTGAAGCATTTCCCCTATGTTTTCTTCTAGTAGTTTTATAGTCTTGGACCTTACATTGAGGTTTTTGGAAGCAATCTACAGTTTTGCTGCAATCTGTATCAAAATACCAATGACATTCTTCATAGAAATAGAAAAACAATCCTAAAATCTGTGTGGAACCACAAAAGACCCCAAATAGCCAAAGCAATTCTAAGCAAAAAGTACAAAGCTGGAGGCATCATACTATAAG

>ORI-108

TTACAGACATTTCTTCCAAGATTATTGAATTAAAGTTTGGCTATTATGAACCACTTATGACTTTTGAAAATTCAAGAGTCATTCTCCCTAAGATGACCTTTTGTTTCAGCAGCAGCATTCCAATAACGGGTCCTTAAGGAGCCAGAAGAGGTGTGGTTTTCTTTGTTTTTTCTACTTTTCTTCAACCTTGTTTCAGTTTTTACTTTATTGTTTTTCTTCATCCTGCCCCATAATTTCATTCCACTGCTGTTTCAAATAGACATCGAGATTAGAGCACCAACACAATTTCAAACTCTGCTG

>ORI-109

AGATGCAAGCACGCTTTCTGCTTCTGCTGGGGTTTTTGCTGGCCTCGGTCCGTGGGGTCACTCAGAGGTTCCATGCAGTAGAAGTTTGGAGAGCTCATGCTATTGGCTCACTGTGGTTTTATGGGCCCTAGATGTGTAGATGCTTCCGGGTTGATGCCCAAGCAGTGGTCCCACCTGGATGGTTTTTGTCTTTCTGCTTTTCAGATTCTCTCCTGAGGCTGCATCTCGGCGGGGGCTGAGCACAGCAGAGATGAATGCAGTAGAGGCCATTCATAGAGCTGTGGAATTCAATCCTCATGT

>ORI-110

AACCTGGAATGTGGAAGGACGGGAAATAATACAAAGTCACACGCTTTAGGTTCTACAACTCCTGACTGGCTGAATTGGCCCGTCGATTTACCTTGAGGCAGTTTTGGCGGGGTGGGGGCTGGGATGGGGGAGGCGGTTGTAGTTTTCAAGGTGAATTTACACACACACACACACACACACACACACACACACACGTCTGTGCTAGAGCTGGAGACCAGGCTAGCACGTCTCTCAACAGGATAAGAAATTGGCTGATTTTGCTGTCGCTCAGCTGGATCCAAATAAACCAAACATCGGAGC

>ORI-111

TTCAGGGATTAGAGGGTTGGCTCTTTAAAAACCGTCAGAGACACAGGCAATCCTACACAAAATTCTCAGAAGGAAGGCGCCTACGCCTGGGAATGCCCAGATGCCCCTCAGAGAGTTGAAGATGGCGTTTCTCTGAGTCAGGTCAAAGTTAACACATTACCTTCGCTTCAAAGACTGCTTGGCTTCCTTTCGGTGGATTAGTCAAGATGTTTTGCTGACTGAGACTAGGAAATCTATAGGAGGGCGGGTTAGTTTACATTGTTCCTTGTCATTATCGCTAAAACACTCCAAAGCCTTCCT

>ORI-112

TGTAGGTCATGATTCTGGGTAACCTGGAAGATGACCTCATTCATATTCTGTATTCTATGTGAGACGTTAAGAAGGTAGAGGTGGCCAAGAAGGAAATTGTTGCTGCCTTTATGGAACAAATTATCTGAAACCCAGCTTTCTCGAGGGCTTCATTGAAGTACTCAACTGGGGCACTTAACCCAGTCTAAGGCTGGTCAAGGAAGGCTTGCTGGGGGAAGTGTCTTTTGTATTCACACCTAAAGGAGGTTATTCAATTAGAATTATCCAAAGAGGGTAGGGATGGGCTAGGAAAAATTTAAA

>ORI-113

ACATCTAGACTTGGAACTGGAGATGAGAACAGAATATTATCTTCCTCATTTTTGTGTTTTTGTTCAACTCTAATGTCTGCAAAGCACTTGCGTATGTAATGATGCTCAGTGTCATAGGAGCAGGCAGGTAAGTGTAAATTTGTCTGGATAGGAGAAAGCATGCACAACATATTTCACATAGTTTTCTGATTTCAGTTTGTTTTTGCAAATTATTCACTCAGTGAGATAGCTTAAAGACGTTATCACAGGGAAAGGCATGGAGATAGTTCTGTGTTGATAGAAAACTTGTAATGTACAGCC

>ORI-114

TGAAGGTAAAAAAAAAAAAAACCGAAAACACCAAACTGATATTAGAACTTAAAATATACCAATTACGGAACAAATCTAGTCAATTTGGTAATCCAACATAGTGTTAATAATTACTTGAAAGGTCTTTAAAAATGTCATCTCAGAAGGAACAGAATTGAACACGTACTGTCTGATCATGACCCACTGAGGTCTTGCGAGATAAATATTTAATAAGAAGGAATACTCTTTTCTCCCATGTAAATATTTTAGTCTCTCTAAATTAATGGTCTCCATGTGCTTTAAGCCACTACTTTCAGGTTA

>ORI-115

CCATCTCTACCTTACCTTGCCCCTCTTGCCCTCTGGGCCATTAGCAAGAAGTAGTGATGGGCTACTCAGCAACAGAGGCATGGAGGTTGGTTAGATGAATATTCAGTCTTCCATTAACTGAGAATTGACTTTAATGTAATCAGATCACTTCATTAAACTTTAAGGGCTGCCTTATATCTGGTGACTATGACTATTTTATTCCTGCAACTTTATTACTAGTGACATATTTGTGCTCATGGAGAAAAATAGTTCACGAAATCCCTTCCAGACAAGGATATTCAGCAGTTTAAGAGAGGGTAT

>ORI-116

GAGGCAGCTCCATAGGAAATGTTATCTCCATTTCACAGAGGCTGAGAGAGTTCAGAGTCCTCCAGAAGCTCACATTGCTATTTAGTAACAGGCATAGACAGGACTTCAGCCTGTGTGTTCTGCCTTCAAGTTCTGCCAGCCTTTCTTCAAAACCTTCTGTGAGTTTAAAACTCATCAACAATCAGCAAAGGCTTAAAGAGTTGTTGTATCTTAATATATCGTCGTCTTCTTCTTTTTTTTTTTTTTTTTTGAGACAGAGTCTTGCTCTGTCGCACAGGCTGGAGTGCAGTGGCACCGTCT

>ORI-117

ATATTCTCAAGGGTTTAGAAGGTGTATTAGCATCTTATTGTTTAGTCCTACTGCAGCCTTCTTGCAGCCAGAAGGGGAAACACTATTCACACACAGAGGGTGGCAGAGAGGGGACGAGAAACAGCTTGGGGTCATTGAGGTCATTGCTAAGACAGTGAACCAGCTCTGGGCCTCTTATTTCTAGACTTTCTGTTAAGTAAATGATAATGATCCTTGTGGTTGAAACCACTTTTAATCAGGAATGCTTTTACCTGATACCCAAAGTTACCCTGATATCCCTATAAACACAGCTTGATCAGT

>ORI-118

TGGATCACAGAATCTAGACTAAGCAAGGACGAGAACGCAAGACAGGGGCTGATAAACAGTGGGGAAAAAATGAAGCAACGAATTAGCTGTCAGTGAGCAAGGAATCTAGGATAGGAAGATGGTGATCAGAGCATGGAATGTCTGATGATGTGCTAGTTCAGGTCCTCTTAGAAGCAGACACCAAGACAGGGTTCAATGTGCAAGAGTTTTATTAAGGGAACTCCTGTGAAGGATAAACAGAAGGAAGCAGGAGCGGGGAAGGGAGACTTCATATCATAATACAGGTCTGACACCTGTGAA

>ORI-119

CCGGCTGCTGGCCCACGCCGCGCCCTCCCGCTACGCACGTGCGTTGGGGTCTCCCTGGCGGAGACCCCGTCCCCACAACCAAGTGCGCCCAACTTACTCAACTCTTAGGGCGGGCCGGCGGGCGGGCGGAAGGAAGGAAGGCAGGCCGGGCGGCCGCGGGGACAGCCTGGCGTGGGAGGCGGCTGCGGGCCCCACCTGGCGCAGCCTCCGCTGACCACGCGGGGGAACCCGGACTCGGGCCCGACGCGCTCCCAAAGAGTCCCCGCCGGCGCTGACGCGGAAGCGCCACAGCTCACCACG

>ORI-120

AAATAAAAATAAGTTGAAACTAGGCTCTCTAAAAGTGTTTTTTTAGGTCAAGGGAATGGGTTAGACAGCAGAATCACCAGGGGTGCTCATCGATTCTACGCTGAGGGTCTCTCCAGAGGATTTAATTTGACTGGGTGGCAAGTTCAGCAGGTATCCCCTGGCAGAGGTGACACCCAAAGTCTGATGGATACAGGTTTGTGCTTCTGAACTAATCTGGCTCTGTGCTATTTGAACTGAGTCACAAGGCACAAACTGAGGCTTTAAAAGACAGTATTCAAACCCTTGGGGAAATTAAAGGGC

>ORI-121

TGGCAGGCGCGTTAGAGACCTAGACCCGTTCCCAAGTAAGGGCCGCACGGGACCCAGGCCAGCAGCGGCAACTCGCGGAGCTTCAGCTCCCCTAGGAGCCAGAGCCGGGGGAGGCGGGGCCGAGAGCCGGGGGCGGGGCCCGGACGGGGGCGGGCCCGCGGTGACGTCGGGAGGGCAGCGACGCGCGGAGGCGGCGGCGGAGCCTCCTCCTGCTGCTGCTGCGCCCCATCCCCCCGCGGCCGGCCAGTTCCAGCCCGCACCCCGCGTCGGTGCCCGCGCCCCTCCCCGGGCCCCGCCATG

>ORI-122

AAATAGGACATTTACACACACAAGCAAGACTCAAAGACATAAAGACTTCTACAGTTGGAAGATTATAGACTTATCTCTAAATATCCACATCTGGTCAACCCCCATCCCCAAAAAAACCTTTTCTAAAAATGGATCAGTGCTAGAGTGATGTGGGAGAGGAGAAGAGAGTGTGATGGTGAAGTGTCCATCAAGTTTTAAATACATATTTCTATATTTATATAAGTAACATGACAAGTTGACAAAGTCCTTTCCAACAGGCAGACAGATTTGTCAGAGGTGTTTGAACCACAGTGACTCCAT

>ORI-123

TTCTTTTTCTTCCCAGTCTCAGATATGTCTTTATCAGCAGCATGAAAATAGACTAATACACAATGGCTCTTTTACCCTCTGATGTTTGTTGGGTTTGGCCAAAGGAGGGACCATTGGAGTGTAAGAGGAGAATGGGGTCAGGGTACTTATTCCCCCATCTCCCTCCCTCCTGCAACCTGTGTCCTCTCTTTCCACATTCCAGTGACCACATAGGCCCCACCGTGTCATTAGCCCTGAGACACTTCATCAGCTCTTGTTGGTTTCCCTACATTCTGACCACGTGTGTAGCATCTGTTTATT

>ORI-124

GGAAATGTGACAGTCTCTAAGAAATGATCATTTCAGTTGCCAGATCCTAGGAATCTATAGGCCCTTTAAAGAAACGTACATTTTAAGTAACACCTTACCTCCTTCCCAAGAATTTAGTTAATCACTACATTTTTCTTCTATCATTCCTATCATGTTATAAAATCAGTTTCTCAAAAAGCTGTTCTCATTATTCTCATGTGTTTCTCAATTTCTTGTTACAAACCCTAGGAGTCAGTTGCTAAAAATCATGATTCAAGCCACTTTAGCAGAGAAACAAGATCTAGACTTGGCCAAGCTTGG

>ORI-125

ACACACACACACACACACACATATTCCGGAGACCTTGGATCAAAGGCAGTGTTCACAGATTCATAGAAAAAACAACAATATAATAATAGCTACCTCTATTTAGTTCTACTTCTTGGCTAGTCTAGACTTTATTTAGTCTTCATTCCATTATCTGAGGACTAGAAATACCATTTGACCCAACCATCCCATTACTGGGTATATACCCAAATGACTATAAATCATGCTGCTATAAAGACACATGCACACGTATGTTTATTGCAGCATTATTCACAATAGCAAAGACTTGGAACCAACCCAAAT

>ORI-126

CACGACCAGGAGACCCAGCGCTCCTGCCGCTTCCGGAGATCTGACCCGAGACAACAGCCTGAAATAGGGAATGCGGACCCAGGCCAGTGTGCGTGGCCGGCCCGTGTGGCCCCCATGCTGCTTAGGAAGTCCCCGTCCAGGAGCACTGGAAGTTGCTGTGAGAGCCAACTAGGGTTGGCGGCCTGAGGGACAGTCACCCAGCCACAGAACGACGGCACGGAAGACATGAGGCAGCATGCTCACGAGAGAGGTGAGGGTCTCACCTCCAGGGGCGGGAAGGAGAGAAAGTGGGCAGACAAG

>ORI-127

GCCTGGGTGTGCTCCGGGCAGTCCCTTCATCTCTGGGCCTTGCTGACCTCTTGAAAGTGACCAGGTTGGGACGGACACGGTGGCTCCTGCCTATAATCCCAGCACTTTGGGAGGCCAAGGCGGGTGGATCATTTGAGGTCAGGAGTTCAAGACCAGCCTGGCCAACATGGTGAAACCCCTTCTCTACTAAAAATACAAAAATTAGCTGGGCGTGGTGGCATGTGCCTGTAGTCCCAGCTATTCAGGAGGCTGAGGCAGGAGAATCGCTTGAACCCAGGAGGCAAGGGTTGCAGTGAGCCA

>ORI-128

CTGAGGCTCGGAGAAGGGGTTGTGGTGGACGCATGAGTCCCTGGGCCTGGGACCCAGGTCGCTAAATGCCCCGCCAGGCGCCAGTCACTGCTGGACAAGGGCAGGGAGGTAGCACCCAGCCGGTGCCTCCTCCCCTCCTCAGCCAGGCAGGCGCCAGGGGAGCAGGGTAACCCAACCAGCCCAAAGGGTGGGCACCGGTGCCTCGCCCCAGGCAGCAGGGCCAAGGCCCGGCTCGGGCCGCACGGGCTCTTTCCCTCTTCCGGTCAGCAAGGGCTCAGGGGGGCAGAGGTGTGCCGCGGC

>ORI-129

TCTCGAACTCCTGACCTCGTGATCCACCCACCTCGGCCTCCCAAACTGCTAGGATTACAGGCGTGAGCCACCGCGCCCGGCCTCATCCCTATTTCATAGAGGGGTAAGCTCTAGAGAGCTGAAGTGGCAACACTTGATTCTATCTCAGGTCGGCCTGATCTCAATCCTGGTATTTAGTGTAAGACCCAGATAGGTGGCCAGAACTGAAGACCCCTTGACTCCAGCTCTCAGAGAGACACTGTCTAGCGGCGAGACCTGGCACTTTTGCAAACAACTCCAGAACAAGGGGGAGAGTGGCGA

>ORI-130

GTCCTAGCAGCCAGGAAACGTGTTTAGTGCATCAAGGTCCAAATTATCCTAACGAGCTTAAGGGCAAAGCCGGGGATCCATGAGGAAAGGGGATTGGAGGGGGTTAGGGCAGGCAATCCTATTAGAGGCAGAAAGGTCCCAGCTGGCCCCGCTGATCTCTCACTGGCCTGCCCCGCCTCACCCTGCCCTGCCCCGCCTGGCCGGCCTCCACCTGCCTTCATTTACCAAAATCTCAGGGCACACCTGTTCTGTGCCAGGCTGTGTGCTGGGGGCAAGCACAGTGACAGCCATGGTCCCCTG

>ORI-131

GGATGCCCTCACTCACCCAGTTCTCCGTCTTCCTGGCCCGACGCTCCAGCCCCTTCTGCAGGCGCTCCCCTACACCTCCTGAGGCCTGAAACTCATCCACCAGCTGCTTGGTGTGGGCCCACTCCTCCTCACTCACGATGGGCTGCAGCGCCTTCAGGTAGTGGTCCAGGGACTGCTGGAGAGGGGGCACGGGCAGCCGTGGCAGTGCATCCTGGTGTGCCTTGAAGCGGCTGGAAGCCTTCATCAAGGAGAAGGGCTTCAGGAAGCCCAGAGGCTTCACCTGCAGGTAGCAGAACATCC

>ORI-132

CAGCCCTTTCAGGGTCTCATGCCAAGACTGCCCTGATACCCGTGGGCATCCTAGGTGAGGGACCCCACCTTGCAGGGCCTCTACAGCCTCTGTAAATGCAGCCCTGGCGCCTTTAAGAGCCCAGGGCAGGCAGGAAAAGAATTTCAGTTTCAATCTGGCTTCTAAATTTGGAGTTTTGGGAAGGGAGGGATCAGATTTCAGCTGGAAGGGAAGGAGCTGACAGGAAGGCGCTGTGCAGAGCCTCCCCACCCCCGCCCATCCCCCCAGTTACTGACAGAGGAGCCATTTACAAAAGGCCGA

>ORI-133

GGCCGCGCTGGCCGGCATGCCCGGCGCCGCGTACGCTAGGTGCTGGTGCTGGTGGTGGGGCGGCTGCTGCTGTTGCTGCTGCTGCTGGCGCCGGTAGAGCTCGGCGTAGCGCTCGCTCAGGTAGAGCTGGCGCGCGTTGCGGAGCACGTAGGACACCAGGAGGTTCTTGTGCAGCTTGATGCCGCCGCGCTGGGTTCGGGAGCTGTGGATCTTGCGCAGGGAGATGCTGATCAGGCTCTGGGCGTCCAGGGCGCACTCCATGCTCCCGCGGAGACGGCGGCGGAGCAGCCGCCGCCGCTG

>ORI-134

TGTCCTACCTACATTTTATTGGTGATACAGACCTACAGGCTGTGGAACTTGCTCAGGTGATAACACGTAGAAGTAAAAATACACTGCCCTTCACTTCCCTGGTGCCTGTCTTAAAGGCACACAAAGACTTTATTTATGGCCGGGCGCGGTGGCTCACGCCTGTAATCCCAGCACTTCGGGAGGCCGAGGCGGACAGATCACGAGGTCAGGAGATCAAGAGCTTCCTGGCTAACACAGTGAAACCCTGTCTCTACTAAAAATCACAAAAAATTAGCTGGGCGTGGTGGCGGGTGCTTGTAG

>ORI-135

TCCCTGACTCTTGTGGGGGCTTCCGTAATGCCCAGTACTGAGTCCCCTACCCTGGGACCTGTGCTCTGCCTCTGGAATGGAAGCCACTCTGTAACCCCTAAAACCAGAGAAATTGGCAGCTGCTCCCTTGGCTGAGAACATCGCTGACTCCAAGTGCCCCAGTGCTCTCCACTCATCATTTCACAGACGACCCTGAGGGAGGCAGGGCCAGTGCGGCCTCATTCCACAGACGAGGAAACAGTCTCGGCCACTTGGCCAAGGTCCCAGAGCTTGTAAGGAGCAGATCTGGGATTTGAACCC

>ORI-136

TCCCCAGGGCCCGGCTTTTCCTGTGCCCTTCATTTTCCTCCCCCTGTTCTGGGACTTGTGGGTGTCCCCAGAGCAAATTCTTGGGGAGCCATGCCCAGCCGGTCTAGGGGGCACTCAGGGGCTTGCATTTTGTCAGGCTCCCTCGTGGTCTGGTGTGGGTACTCTGGGGACCACATTTGGAGAACCTGGGCCCCCCAGTTGTTAACTAGCAGACGAGGGCCTGTACTTAGGGGCCATTGTCCAGCGTTTAGTGCAGAGTGACTGGAGAAATGGGAGGACCAGACCCTGTGATGGGAGTGA

>ORI-137

AGGTAAGGAGGCTCTGGGAGGAGTGGCTTGCCACACTATGCAGATATAAAGAGGGATATTCACCAAGAAAAACATACCAATAACACCAAGGGCCCTCCTACACATTGTTCTACCACTATTTATTGAGCACTTACTACATGCCAGGCAATGAGCCAAGTGCTTTACAAGGCTGACCTATTTCAATCCAACAGCATACCGAAGAGGGAGGCATATTGATCCCCCTTTTACGGGAAACAGTGAGGTTGATGCATTTGCCCAAGATCACACAGCTCTTGGGGAGTGGAGCCTGACTTTGAATTT

>ORI-138

CCCCAGTTTTAGGAACCCCCGCGGCATCCTTGTATATATATATATGTATATACGTATATTTCTTCCTCAGCTCCTCCTTTGTGTCTGAGGCGCTGTGGGGACTGTCAGAGGGGCCTGGAAACCTCCCAAACTCTGGCAAAGATGCCCAAACAGAGCAAGTTTGGCCCTAGAGAGCAGGGAACCAATCCCGGCCCCATCCCACAGAGGGGGAAACCGAGACTCAGCCAGCTGAGGGGCTAGTCCAGGCCCCCTAGGAAGCCGAGGTGGCCTCAGCCTCCAGGCCAGGGCTTCCTAGGACTG

>ORI-139

ACACTGCCAATAAATTATATTTATTGATAGAATCATTAATAGCTAAAACCCTGGCCATAGCACAGATATTTTCTTGGAGTGAATATTAAGGAGCTTCCCTGAGTTCTCGGCCTCCCCACCACTGGCTCCCCTTCTCCTCAGAACATTCTGGAGCGGGCCAGTCACGGTAGCTCACGCCTGTAATCCCAGCACTTTGGGAGGCTGAGGCAGGTGGATAACGTGAGGTGAGGAGTTCGAGACTAGCCTGGCCAACATGGTGAAACCCTGTTTGTAAAACTACAAAATTAGCCGGGTGTGGTG

>ORI-140

CAATGCTATAACGCAGGCTTTAGTAACCCCATCTCCCAGAAGAGGACCACACTGAGGTCAGAGGGTGTAGCCACCAGTCCAAGGTCACATAGCAAGGGGCCAAGATTCTGACCCTAACCCTTGTGCTATGCCTTTACCACCCAACAAGACAGATCATGGTGCAGCCTTCAGATACCAAGATGCTGACGACAGGTCTTTACACAGAAGGATGTCGGTGGCATAGTGTTGGGTGGAAAAAGCAGTCGAGCACGTACCATTTTTGCAAAAATAAAAAGCACATTCAGTGTGCAGAGAAAAAAA

>ORI-141

CTCCTCATCCTCCTAAAGTGCTGGGATTACAGGCGTGAGCCACCACGCCCGGCCCAGGGTCAGTTATTACCCCCATTACTTAACACATAAAGAGGCCAAGTCTTGGAGAGAAGAACTCGCTGGCCCAAGGCTTCGCTGAGAAGGAAGGATTTGAACCCTAGCACTCTGGCGACAGTGGCCCCTGACTGACAGTTGTCCTGCCCCCAGCCCCAAACTTCTGATCATACAGATAATTCCACAGATGAAAGAGTGGGGCAACCAGCACCTGAATGGAAGGCACAGCGCCACGGTCCCATCTAG

>ORI-142

ATAGAGTGGATGGAGTGGAGTGAATGGAACGGATAGAGTGGATGGATGGAATGGATGGAGTGGAGTCAATGGAGTGGAGCTGGAAAAAAGTTGGAGAGGGAAGGGAAGGGAGGGAACAAGGGCAAATGGGCAAGTAGGTGACTTGGAAGATAAATTGTACAGAACGTGCTATCAAAATGGAGAGATTGAAGATGGGAAGAAGCAGGTATTGAGGATTCAGGGTAAGGAAGGAGGCAGGAAGAAAGTCTATGCTCTTATCGGGTTCTTATGCTAGTGGTTTTGGAGGAAACTGACATCTGC

>ORI-143

TCCTCCCCTACCCCTCCCATTTTGCTTGGTGAATTAGTCAAGGAAAACCTATTTTCCTTGCAAAAGCCTCAGTGGCTTTGTGCAGCCCTAAAGGAGAACCCTCTCCACAATGGCCTTTCCTGCACACCCCATCCCTGAAGTTGAGGCTGCTCTCAGGAGGCTTCCCTGCACCCTCCAGCAGCAGAAGTTCTTCCCCTCCTTCAGAAACCAGGATCCTGGCAGCTGCCCCAAGCTCCCGGCCCACCCACGCCTGAACACTTAAAGTGCAGCTTTTCTCTCACACTCAAGGGATGGGAGCCC

>ORI-144

GGGCGTCCTCGATGAGGCTCTGCCTGCGCCCAATGAACCGCGGGGACTGTGGGGACAAGGGGCACCCATGCCTCCTCCACCTGCTGAGACCCGGGGACCTCCACCCACAGCTGGTCCCACAGTCGGGCAGCGCTGATGGCACACAGAGGCAGGGGATGAGAGCACGTTTTTGAGCGCCTACTGTGTGCCTGCTGGGGCAGATGCTAGCCGAGGTGCCTGCGGGCATTGCACGCCCTTCCCGATGGGGGCAGCCCAGTCAGAAGCAGTGGTGGTGGGGGAGGAGTGGGGGCTGGGAAGGTG

>ORI-145

CTCCAGCAGCTTCCCCATCAATGCACGTCGCCCGGCGGCACACACAGAACAGGCCTTCCGTCAGGCCTGAGCCCCTTCCCTGGGCGGCACCAAGCAGGTGCCTCTCCTGGTGAGGGGAGTTGGGGCACTTGCCCCACCCCACCCCACCCACCCACTGGGCCACTCGGGCCCACCCTCCCGCCCTTCTCCCACCGACTCTGGAAACCCAAAGGGAGGAAAGGAAGGGAGAAACCAGACACACAAAAAAAGAAGTAGGGAAGGAGGGTGGGGACAGTGAGACACAGCAAGCCTGGGATGGGG

>ORI-146

CCTTCCCAGCTCAGCCCTGCACCCTCCCCCATAGGGGGCGCATTCGCACCACCACGGTCTCGCCCCGCCGCCACAAATCCCGACTTTTCCTAGCCACGCCCCTGCCCTAGTCCCGCCTCGCAAGCTTCGTCTCTCCAGTAGCCCCGCCCCAGAGCCGCTTAGCCCCGCCCCAGAGCCGCTTAGCCCCGCCCCGGTGCGGCCGCACTCCTATCAGAGGCCGCCACCCGCCCCGCCACCGCGCCTCCTCCGCGGGCCCGCGCCGCCTACCTGCCGGGTACCGCTCGTTGACCGGCCAGCTGT

>ORI-147

CCCTTCCCAAAGATGTATGTGGTCCCGGCTGCAGAGAGAAGGAAAGGGAGACAAGAAGAAATGACATCAGGAGGACGAGGTCAGCAGGTCCTCAGGGTTGTGACCAGAGGGATGGAACCCCAGGTCTAGATGTGGGACTTCAGCTGTGATACCCACAGCAGCCACCAGCCCTGCCACCCCAGGGAGAAGTTGGCAGGCGGGTGCCTGGGTGAGAGCCTGTGCACGCGCACAGACAGATGTAGCTGCCTCATCAAGAATGCTAGCTTTAATTACAGCAAAATAGAGAGGTAATAGAGTGAC

>ORI-148

CGGGCCCCCTCGGCCGCCCCCAGCAGCAACAGCAGCAACAGCGGCAACAGCGGCAGCAGAGGTGGCGGCGGCGGCGGTGGAGGCGGCGGCAGGGGCCCAGCCAGGGCCGGGGGGCGGCGCGGGCACCCCCCCGGCCCGCTCCCCCCGGGGGGCATTTCGGCCCGGGGGCGACCGCCTCACAGCCCCATGGCCGGGGGCGGGGACGCGGGGCTGCGCAGCCCCGCGAAGCCCCCCTCCGGCTCCGAGCTCCGGGAGCGGGGTAGGGATGAAGCGGAGGTAAACCAGGGCCCAAGCCTCGGT

>ORI-149

AATGAAGGAGCCAGAGAATGGAACTGCTGCGTCAGGGCCTATGAATGCGAAATCGCCTCCCTGACCCCACAGCCCACTTTCCTGCCCATCCCCCACTGTTCCTCTTCCTGCAGCCTCCTCACACTCACACACACACACACACACACACACACCCCTTGTCCATCCAGGCATCAGGGCATAGACAGACAGATGCAATGGCTCACAGAGACAGACTCAGACCCTTACAGAGGAGCCTGGGCAGGGAGGGAGGTAAGTATGAGCACTTGTCTCCTTTCCCTACCCCCAGCTTTGCCCATCAAG

>ORI-150

GAACCTACCCTCGGCAACTTTACAAGACTCCCTTGACAAGGGTGGCAGAGATTAAAAAACCCCACGCTTTAAACAAACATCTTTGGGGGTGGCTATGGCACCTTGAAAAACCCACAACCAGCTTGACATGAATGGCCAAAGCCCTTGCCCAAAACCATTTGCTCCCCTCTCCTTGGTAAGGGAAGGAGAACTGGAGAGAAGGGAAAGGAATCTAAATTGCAGCAGAAGACCGGGGAGAGCATCTCCTTGGACGGAGTCTGAAGAAAGGAAAAGATAAAGAAGTAACAAAGGAAAAAGAAA

>ORI-151

GACTGAGATCTAAACATACTGAACAGATTTAAAAAAAAAAACAAACTAAAATTCTCTCCCCAAAAGAGACCAAGAAAGATCAGACCCAACCATCATCTGAAGCATGCCAACCTCTCCCGGATGAAAACCCTGCCTTGCTGTAGAAGCAGAGGAGCTATCCTTGGTACGGTTCCCATTCTGGTTCCTCAAGACCAGAAGGGAGTCGCTGCACGTCATCAGGTGAGGCTTTCACGCCCTGCTGGCATTCACAGCTAGCACCCCGTCCAGCGCTGAACACCCGCCACCCCTGTTCCCGACACC

>ORI-152

TAGTCTAGGATACTGAACCCAGAGATGGACAGTATTTCTCTCAAGGTCACACAGCCAGGCAGGAGTGGAGAGGAGCCCAAGTCCAGGGCCCAGAGTCCCCTCTGGTACCCCAGTGGGGTAGGGGTGGGGGAAGGCTGGGGCCTGCTGGGGCCTGGAGCCCTGGCCTCACCATGGTAAATCTCCTGCAGGGAGCCCCCTCCGCAGAACTCCATGCAGATCCACAAGCGGTCATTCCTAGGGACAAAGAGCTGGGGTGGGCACAAAGCAGGTCACATGGGGGCTGCCTGGGGCCAGGAGGAT

>ORI-153

TCAGTTTCCCCCTGCCAGGAGGTAAACTCCCTGCCAGCTGGGCGGGTCTGTGGGTGGGACAGAGAGCTGTTCCACAGGTTTATCAGGCAGAAGCCCGCAGCGGGGGCGGGGTGGAAACCTCATTACAGAAATTCATTGTCTCTTTCCCAGGTCTGTGTGTGGAGCTGGGGAGGAGCAGCCCCGGGCCTCCCCGTGGGAGTTGAAGCCAGCACCATAGGGACCCACCCGCCAGAAGAGGGCCGAAGCCCAGGCCCACAGGCAGGAGGATCCAGGCCCCTGGACAGGCTGCCTAATCCTCTG

>ORI-154

GTTGTGTGTCTCAATGCATCCGAGAGGAAGGAGAAACAGAGCCCGTGTATCAGTGGGTGACATGCATGAGGGTCTGTGTGTGTCCTGCGTCAGGTGTGGCCTGTAAGCAAGGAAATCTGTAAGGGACTCTAGGCGGTGCACTGGGTACATGCGTCTGCGGTCGATACCTGAGCACAAGTTGGTATGTGTGTGCATGTGTCGGGGTCTGTGAGAGGGAGAGAGCGTGTGTGAGAATTGGAGTGAGGGTGAGGTGTGCAGGCGAGTGTGCGGGCCGCGGGGGTCCGGTGGTACGCGTCAGGG

>ORI-155

TCAGAACTGTGCTGACCATGGGCGGGGGGGACCTGGAAGGGCATATTTGGGGGCTCTGCAGGTTGTTACATGGGGACTCATTGTGAATTCTATGAATATACATTATACAGTGATGTTTTATACACACCACAGCGGGAAGAGCCCCACCAGGGCCACCTTGGGTAAGGCAGTGCGCGCCTGGGACCCGCAACTGTGTGTAGGACGCTGGCACCTGGGCTGTGCCCTAGGCCCAGGACAGTGTCCTGGCCAGGCCAGGCGCAGTTCTAGGCGGCCCGGGGAAGGCGAACCCAGGGGCTGCTC

>ORI-156

CCTCACGACAGTGTCCAGGAAGGTCACTTTGATCCTCCGAAGCACTGAGGAGTTGGGGAGGGGTCTCAGGTCAGGTAGAGCAGCAAAGGGGAGGCAGAATTTCCCATCTGGGCACCAGGGTGGCCGTGCCGTTGTCTGGAGCCCCTGTTCTGTGTGCCACTGAAGGGCAGGAGACAGGGCGGCCAGGAGGGGCCTGCTCACCAGTCTCAATGGTCTGGGCAAACATCTCCAGCCCCTCCAGGGGCTGTGGTGGCTCAGAGGGCTCCGGTAGCCCATCCCGCAGACACTCCTGGGCCAGCT

>ORI-157

ATGCAATTGTGTGTGAGAACGTCAGCACAGAAAGCAAGTCAGGCACTCAGCTGCATGCTGAATTAAAGCAGGTGTGAAAGCTTGAGAAGAAATATTAACATTTTGATAAATTTTTTTTTAAAAAAATCAAAATCAATGTTAAAAAAGTATTAACAAAATATCAAAATTTCAAATAGAGGCAGGATCAGTATTACTGATTTTTCCTTTGGCCTGTGGCTCTGCTATGGCTTGACCTGGTACTCTACTGATCCTAGCTTTATTTAAACTTTTGATCTGTCATTCATCATAGGTTTTTTGCAT

>ORI-158

ACCGCACCCAGCCTTGGGAATTTTTATAATTAAGAGAAAAACCATATCTGAGGAAATTATGTTGTGAATGTTAGCTATTATTGTTGCATACTATCTGGGACAGAAACCTAATGATAGGTGTACACACTGTCTTGGTGTCTGTGCATGCGTGTGTGTTTGTGTGTGTGTGTGTCTGCAAAGGGGAATTTCAGGTGATTTGTAACTTTTTTTCTTTTTTTAACAGTGTGTCATGACAGCCAGAGGCAGGCGATTCCAGTATTTTAAAGCAAACATAGAGATGAATATTATCTCTCCAGTTAA

>ORI-159

TTTGTCTTTCTACACAAATTGCCTGTTTCTGGAGTGGACTCGGCTTTCCACACCATGGGTCCGGGAGGACCGGCCTTCTTGGCAGGTGTGGGAGGAACAAGCCTGCGTGGTGCTGTCCCTCCCTCCCCACCAGGAGCAGAGCAGCAAGGGGACCCTTGAACATTTCCCCTCTGGGGAGGCCTCAAAGCATAGCACAGGCCCGAATGCATTGAGGGTTCAGGCAGGGATGCCAGCGAGCCAGGCTTCTGGGGCCAGGTGAAGGCAGAGGCGGCGACTTGAGCAAATGAGCCTCCTTTGAAG

>ORI-160

TGAAGTGGCTGAAAGTGGCTGTAAAATGCTAAATCATCTTTTTGAAGGAAGGTAATATAGATTCATTAACTTGTACAGAATATATCATATTGGGCCAGGTAGAATATCAATATTTCAAGCATATTTCTAACAATGAAAAGAAAAAGAAAAACATAAGACACTTGAAAACTGAAGCATTTTGCAATGTAATCTCGTGTCACTAGTACCATAGACTTACTTTATCTGAACACTGAAAGGAATGGCAAGATTGTGGAAACATGTTGAAGGTTTGCTTTTGAACCTGATGCTTGATGTTGACTA

>ORI-161

TTAACGTTCTAATACTTTCTTCCTGAATCTCTAAGGATTGATTTGGCACTTTAGCAATCACTGCTCTAGAATGTCAGGATAACCTTTTGGCAAAAGTAACAGCAGAATCAAAGGAATTAAAAGGAGGGTGCAGGATATTTTTGTAGAACTTAGGCAAGGAAGCATCACGAGGTGACAATGACAATAATAATGATAATGACAATGATGATAATGATGATAATTTCTTATTTTTGAGCAACTAACATGATCACTTAGCCTTAAAAGCAACCATGTGAGGTAGGACTATTATTCCTACTTTTC

>ORI-162

TCTCCTCTACACGCGCCCTCGCTGAGGCGCGGGTCCTACTGAAGATCCGGATAGTGATCATCCTTTCTGGCAGCATAAGTCCCGCCCCCTTTCCTTCCCTGGGCTTCTGTTGTCCAGGCTACCTTGGCGTTATCAAAGCTACTTGATCTTTACAGCCACTTCCCTCTGCACAGGCATCCAAATAGGAAATTCCGCTCTCAAGGAACCTTCTACCCATCCACCTGCCACCAGCACGAACCTGCTGTCTTGTTCTCTGGCCTCTCTCCCTCAGTCCCCTTTTGCGCCCTCATTTTCCTGTCT

>ORI-163

GTGGGGCTAATGTGCAGCCTCTTTCCATAGAAATGAGAGATGGATGGGCCAGCCCGGCTCACCCTTTGGAGAATCCCCCCTTCCCAGCCGGTTTCTGACCCCTTCATCTTGGGAGGGGGACAGACAGACGTCCAATTTTGATGCTTGACTCCCTTTCTCCAGGCCTTCAGCTGTTTTCTGTTATATAGATCTATATAAGTGTTTGCGTAGCTTGCATTTTTAATTCCCCTTTGCTGTATTATTACGCTTATGGCATCACATTTTCTTTTAATCTAAGCGGTAAATATTCTTTAGTGAACT

>ORI-164

GGAGCAGAGTGACCAAAAGAAGACCCCTGGGGAGCAGACGAGGTATGGGCAGAGGCATCACCCCCTCCACTCTCTCTGCTCTCAGCCCTCACCAGCCCACTGCCCGGGCATGCACCAGCCTCCTTCGCCCCCATCGCCTGGCCCCTGCCACCTGGGTTTCTCAGCCGCTGCCTTCACTCACCCCTCCCCACACCAGTCTGGAGTGATAGGCTGCGTCTGCACGCGGCTGGCTCTGCCTAGGTGCCCAGGAGTGACTGGCACAAGTGTGTGCTCTATGTGCTTAATGCCTCTAACGACCAA

>ORI-165

CAGGTCGGTGGCAGGGTGGGAGGAAGTGTGTCTTCGGGCAGCACTTTCTCATGGGACAGGTGTTCTTTCCGTAGACAGTCTATTTATTTATTTATTTTTTGAGACGGAGTCTTGCTAAGTTACCCAGGCTGGAGTGCTGTGGCGCGATCTCTGCTCATTTGAACCTCTGCCTCCTGGGCTCAAGTGATTCTCCTGCCTCAGCCTCCTGAGTAGCTGGGATTACAGGTGCCCACACCACGCCCAGATAATTTTTGTATTTGTAGTAGAGACAGGGTTTCACCATGTTGGCCAGGCTGGTCT

>ORI-166

CAGAAGCACCTGAGGGAGTCTGCCTCTTCCCTCCATATTCACTGTGCTGAAAGCTGCAGCAGGTGCCCAGTTCTGCAGGAAACAGGCTTTGAACATGGATTCCTGTGAGGAGGATGTGGGGGCGTTGGGGGCCACTGTGGGAAGGTGGGGAGATGTGGGGGTGTTGGGGGCCCCTGTGGGAAGGTGGGGAGGGTGTGGGAGTGCTGGGAGCCCCTGTGGAAAGGTGGGGAGATGTGGGGGTGTTGGGGGCCCCTGTGGGAAGGTGGGGAGGGTGTGGGAGTGCTGGGAGCCCCCGTGGAA

>ORI-167

GTCTCATGGCGAGCTGGGTATTTTCACTTTAAAGATGAACAAACCAGATTTTCAGTGACTTGGTGGATTGCAAAGAGTTCAGACGCCTCCATTCAGATCCTGCTGCTGCATGATGGCTGCGGGAGCTCGGGCTTCACCGCCACACCTCGAGTGTCTCAGTCTGTGAAATGGAGATGATAACAGATGCTGGCCTTACAGGATTGGGTTGCAGATTAAATGAGATAATGGACACAAGGTGCACAGCACAGAGTCTGGTGTGACCACCGCCCTGCCACCGCACCACCGGGAGGCACACACCAG

>ORI-168

AGAAATAAACAGTTCCTCAGGTTTAAATTGCATGCCATTCCGAGCAGCGTGATGAAGTCTCACGCCGTCCTGCTGTCTCCTTCCTGGGATGTGGCTTGTCCCTTTGTCCGGTGTGCCGATGCTGTGTGTACACTACCTGCCCCTCAGTCACTCTGTAGCTGTCTTGGTGATGAGGTTGACAGATCACAAGAAGAAGGGTGAATATGACACAATAATATATTTTAAGAGGAGGAGGGAGAGACCAAATTCATGTAACTTTGATTACGGTATATTATTATAGTTATTCTATGTTTTGTTGTT

>ORI-169

CTGGGACGGCTGCACAGATGAGAACACAGGCCAGAAGGGTTTGTTAGTGACTTCTCTGATGTCACAGGCTGGTCAGCTCAGCTGGGCCACATTCCAGCGGCCCCAGCCCTGTCCCACGTGCCCCAGCTTTCTCCTGGGAAGCTGCAGGGAAGGGTCCAGCTAGGGCGATCCCAGCTAGGGCGATCCCAGCTGGGTCCCTGTGGGGCTTCAGAGTGGACACAACTTCCCTGCCAGCTGGCTCGGATCCAGGGGGGCAGGTGGGACCCCGTTTTACATGTGAGCCCAAGGAAGTGAGGCCTG

>ORI-170

CAGCTGGAGGAGTGAGATCCTAGAGGAAGGAGGAACCTTTAAGCAGGGGCCACACCTGCCCCAAGACTCCTGGCCCCAGGTGTCCTCACACAACACCCAGGGGTCCTCAGGACCCCGGAGCTGACTGGCACCTGCTGCGGAAGAGGATGGCCTGGACCTGCCCTGTGGACGGAGGGATCGCTGCATCCCTTCCTCCACCATGTCAGTGTGACGGGCCGCGCGTGCCATCTGCAGCCTTCACTGGGTAAAAGTGAAGGTCCCTCACCTGGATCTTTTCCACCGTATCTTGAGATCAAGTGG

>ORI-171

GAGGGCTGGTGCCCAGGAATTCCCTCCTGTTTCGTGTCTTCAGGTGAGAACTGCCCTGGCCCTGTCTTCAGAGCTCAGCTCTGTGTGTCTGCTGCTGTAGAAAGAGATTCCACCTGGTGACTGCCGAGTGTCTGTGGTTCCCTGGCCCAGTGGTTCTGGGCCACAACTTGTGTAGACTCTTGCACTTGGAAACGCAGGCTGTTGGTTTAGAGCTGGCTGGCTGGGGGGCTGCGTTTCGGTATAGTCACCCCAGTCAGGGTATGTGCTTAGTTCTGCGGGATTAAGAATTTGATTTCCATC

>ORI-172

ATAGCAGAGTGAAAAGGAGGCACACGTTCATAGAAAGTGAATATAAAGCTTGCACAGAAATAGGCTGACCTGTTCAGTTTAAAGAAATGTAATGCTGACCACAAGGTCTCTTTCTCTTTCCATTTTTATGGCCAAAAATTCCAAGCCTGCTGGTAGGAATGCCAGTCCTTATCGATATACTGGAAAACAACTGATGTCAGTAAAAGTCTTCAAATATTTTTAGTTTTCGTCAGCAATGTTCACTTCTAAGAATTTAAAAGAAGAAAATGATAAAAATATTGTCACAAACATTCATGAACA

>ORI-173

AGCCAGGAAGGCGCTCAGAGAGGCCTGAGAGATTTCTAGCAAAAACCTGGTAAACTTTGAGGTGAAGGCTGTCAGAAACAGCTGCGAGGAGCATCCAGCCTAGGGCTCAGCAGGTGTGACTTAAAGGATCGCAGGTGACAGCCCTCAGTGGACTTCTGTCCTCACCATGTTTCCCACTGGCACAGGTGGGGCGGCCACGCCTGTGCCCGCGGCGCCTGTGCTGAGTCCCGACGCTGTGCGCCCCACTCCCGAGGCTGTGGTTGGGGGGTCGCGGTCGAGCTCTAGGGCCCCCGGCCGCAG

>ORI-174

CCCAGCTCTCTGTAAAGACCAATAGTTTACAGCATGGGGAGCACTCTGGCAGGTACCTGGAGCGCCTAGTGCTCCCTAATGCCAGGTGTAATGGTGACAATCGTTCTTTAGGCAAGTATTAAATGATGTTTATGGAAAGATGGCATCATCTGTGCTATAATTTAAGGCAGAATAAAGACTAGACAGAGCTAGAGAGAGACTTAGCTGAACAAATCAGCTCCTGAAATTGTGGGTGTTGGAAGGTCCAGGGGCAGCTGGGGACTGGGGAGAGTTGGGTTGCAGTCCAGGCTGTCTGGGAGC

>ORI-175

TGAGAGGCAGACTCTGGGTCCCTGGGCCTGCGAGCCTGAGACCGGATGACCACCAGCTCCTGGCAGGGTCCACACCCCGAGGCACAGGCAGGGGGACGCATAGCCCAGGTGTGGGACCTCCTCCTTCAGGCCGGGCGGTGCGGGTGCGGCTCAGAGCCCAGTGCCCTCTGCCTCCTGTTGCCTGGGCCGCCAGCGTGACCCACCTGGCATGGACGCCTGGGGACTGAGCCTTGTCCGGCCCCCTCCCAGCCCTGGCTGTCTCCCTCGGCAGGAGCTCGGCACCCCGCAGGTTTTCTTCCC

>ORI-176

ATGGTCTCAGGTAACTTGGGAGCTCGTCAGGGACAGAACCTGAGTTTGAACGAGCTCTCTGTGGGACTCCCACCCAGGGCAGCGGTTAGGAGCCCCCTCCTGGACTGCCAGTTTTCAAGTGACGTATTTAAGTATCCCCCTCCCGGTCCTGGAAAAGTAGAGAGGCAGCCGGGAGCCTGCCTTCTGTGTTCTCGGTGCAGGGGTATTCTGAGAACGGCCCCTGCTCACACGGGTTTAAAAGGAACTCAGTGACCACAGACGGATGAGAACAGCGGACGCAGCTGCCTCCCAGGCACTCAA

>ORI-177

AATCACCCAGAATTCTCTCTGAACACCAGTGCTTTCTTGCTATAATAATTTACGGTCTTTTTTTTTTTTTTTTTTTTTGAGACGGAGTCTCATTCTTTCGCCCAGGCCGGACTGCAGTGGCGCGATCTCAGCTCACTGCAAGCTCTGCCTCCCCGGGTTCACGCCATTCTCCTGCCTCAGCCTCCCAAGTAGCTGGGACTACAGGCACCTGCCACCACGCCCGGCTAATTTTTTATATTTTTAGTAGAGACGGGGTTTCACCCACGTTAGCCAGGATGGTCTCAATCTCCTGACCTCGTG

>ORI-178

CCTCAGATAGAAGCATCTTTTATAAAATGCTGTTGCAACATCTAACCCTCTTCCACTGTACTGCATGTGTGAGTGTGTGAGTGTGAGTGTGTGACTGTGTGTGTGTGCGAGCGAGTGGGAGTGTAAGTGAATGTGAGACTGTGTGAGTGTGTGAGTGTGTGCGCGAGTGGGAGTGTAAGTGAATGTGTGTGAGACTGTGAGTGTGAGAGTGTGTGTGTGTGAGAGTGAGTGTGTGTGTGCGTGTTCACATTTTGGGAATTTGTAATAGAGTATATATATTCATGTTTAGAGCTAGCCTAA

>ORI-179

TCCCGGGGGTGTGGGTGTCCCGGGGGTGTGGGTGTCCCGGGAGTGTGGGTGTCCCGGGGGAGTGGGTGTCCCGGGAGTGTGGGTGTTCCGGAGGCGAGGGTGTCCCAGGAGCGTGGGTGTCCCGGAGGCGAGGGTGTCCCGGGAGCGTGGGTGTCCCGGGGGCGTGGGTGTCCCGGGAGTGTGGGTGTCCCGGGGGAGTGGGTGTCCCGGGAGTGTGGGTGTCCCGGAGGCGAGGGTGTCCCAGGAGTGTGGGTGTCCCGGGGGCGTGGGTGTCCCGGGAGTGTGGGTGTTCCAGAGGCG

>ORI-180

ACTCTGTCGCCCAGGCTGGAGTGCAATGGCACCATCTCAGCTCACTGCAACCTCTGTTTCCTGGGTTCAATTGATTCTCCTGCCTTAGCCTCCTGAGTAGCTGGGATTACAAGCATGTGCCACCATACCTGGCTAATTTTTGTATTTTTAGTAGAGACAGGGTTTCGCCATGTTGGCCAGGCTGGTCGCGAACCCCTGACCTCAGGTGATCCGCCTGCCTTGGCCTCCCAAAGTGCTGGGATTACAAGCGTGAGTGACTGCACCCAGCCCACTGAATGGTTTTTATGACCCAGATCTAGC

>ORI-181

CGAGAAGTCACTCTGACAGTTGGGAACGGCTCTGACCAGAGAATAAAAAGATGGGAAGGCAATTACCATGGATCTCGTCTTTCCTGTTGCTGCAGTGGAAATGCAGGCGAGTGCCAACTGGCCAGCTCATTTGTATGAAGATGAACTAAAGATGAACAAGACTCCTCCCTGCCTCCCCTGGGGGCTTCACACAACAGGACAGACCACAACATCCAGGGAGCCCAGGCTGAAGAGACAGAACCTGACAGCCCAAGTGGCAGCTAGAAGCAGGGGCTGCAACCCCATCCAAACCCCAAGTTC

>ORI-182

GGCGCAGGCAGAGAAGCAGCCCTGGGACCCCCGCAGAGCTTCCTGCATGTGGCCCAGAAAACCCCTGAATCAGACCTCTCAGGAGTTGTGTTGAAAATCCACATTCCAAGGCCCCGCCCTCGACCTATGGACCAGAGTCTCTGAGCAGTGCAAACTGGGAATCTGCATTTTAACTAGCACTGCTAATAAGGATTCTTCTAGTGAAAGTGTGAGCAGGACCAGACTAATAAGGTTTTCTGGCTCCACGTCTTCCAGGAACCTGTGGGGCTTTCTCCAAGAGGCAGCCAGGCCTCTGGACAG

>ORI-183

AATGGACAAATAAAGAGGGAGTTAGGAGATAAACAGGCAAAAAAAAAAAAAAGGCCAGGAGTCGGCTGCAGGTGTGTGAGCGGGGATCAGTGTGTAGCTGTTTCTCACTCACACGTGGGATCTCAGTCTCCCCAGAAGCACCCCATGCAGGCCTTCCTCCTCCTCCTCCTGGAGACAGGAAGTCTCCCCTCTCCATAATTCAAGACTTCCTTCACCAGGCAAGCCTCCATCTTTCCTTTCTTTAAATCCACAAAAAATCTCGACATTCCACCCCAAGAAGTATGGCAGAGCAGTAAATCT

>ORI-184

AGGCCAGTTTGTGTCACCACACCGGCCCATCTGCCCATCTGTAGAATGGGCAGCCACACCGCCCGCCCGGGAGAGCAGGATGAGTCGGCACAAACAGCTGTGTCTGGGTCTGGCCCAGAGCCGGACGCACAGCAGGTACTCAGCCCCCGCCCCCACCCCTCACACACCTCACAGTGTTTCACTCCCTCTCGTGGCCACAGCTTTTTAAGAGGCTCCTTTTGAGCCACTTTTTTTCTTTTTCAAAGCTAATGGGCAACACATTCTCCCAAAGAGAAACTCAGCATTTTGGACGGCTCCAGC

>ORI-185

ACACTCCCAAAAAAACGCTGGCTTTGGAGGTCTATGCACAAAAAAAAAAAAAAAAAAAAAAAAAGTCTAAAAATAAGGCCCCTAAAATGCCTCTTCTTAGGCTCCAAAACAGGGCAATGCTAGATAAGCTCATTCTTGATGCCTTCATGGGTTTAAGCATTCCACAGGGGTCTGTCACCCGTCTCTCTTCTAGAGGGACTTTGCAGAGGCTCCTGCTCCGTTCCTCACACATGCGCACTCACCCAGCGAACACGCCCACCGCCGAGGAGCGTGGAGACACAAAAACGCCTGGAAAAGCCC

>ORI-186

ACGGCGCCACTCATCAGGGCAATGGCGGCGGGCGGAAGAGGGATCCCCGTGCGCCCCCAGCACCCGGGACGAATGCCACCCCGGCGAAGCGACCCGACCGGCGCGCACGGTTTCGGGGAGGGGACCACTTCCACATTCCCCATGCCCGAGAAGGTCACCCACCCGGGAGAGCGCCCAAGGGCCAGAGAGGAGGCGACTGGTGGAGGAGGGGCGGCCCCGTGCGCACAAAGGGGCCCGGGGCCGGCAGGCTCCCCGCCCTTGACCTTGGGTCTCCGCCTCCATCCATCTCCGCCGGCTGCC

>ORI-187

GCACTTGCAAATGTCTTCTTGAACTTAAACTGGGTTTTGCACCGGCTCCTGACACTTTCTTTCAGGGTCTGGTAGGTGGAAAGCGCACTTCTACCAGGAGGGGAAAAAAAATGCAAACAAATAAAAAAATAAAAGAAGAAAAAGCAAAGGAAAAAAAAAAGCAAAGAAAACTTGGGGACTTGTCTCGTACCCCCTCACCCCGCCCCCTCAAAAAACCCAAAGCAATGTAAAACTGCCCCGGTTCGGTTGTTTCTGGGTTTTCTGCGGGCTGCCTGTTTTTGTTTTTGTTTTTGTTTTTTC

>ORI-188

CTGGGATTACAGGCATGAGTCACCACGCCGAGCCTTATTTTTTTAGATATAGGATCTCTCTCTGTCACCCAGGCTGGAGTGCAGTGGCATAATCATGGCTTACTGCAGCCTCCAACTCCCGGGCTCAAGCAATCCTCTCACCTTGGCCTCCCAAAGCGCTGGGATTGCAGGCATGAGCCACTGCACTCAGCCTCAATAAAACTTGATTTGCGAAAATAGGTTGGAGGCCAGATTTGGCTTATGGGTTGTGGTTCACTGACCCTTCCTTGGTTTAGGGTAACGTTAGCTGCTGCCAAAAAT

>ORI-189

GATATCATTAGGGGGTACTGCCTAGCAGTGAAGACAGATAAGTGACCAAAAAACCACAAAAGGATGGTAGGCAAGGTCATGCAAATCCCTCTTCTTCCGGGCCACCTTCTCCAATCTCCAGGTCCCTGTTGATCCTCCTGCCCTGTTCCTGCCTTTCCCACGCTGGCCTCAGTCTGCCCCTCACAACTGCATGCGCTGTGGCCATTTGATTAGCATCTCTTCCCCTCCAGTCCCCACAGGCAGGCTCAGCAAGGCAGGGACCATGTCTTCTTTGACCCCCTGTGAGTCCCCAGTGCCTTG

>ORI-190

TTCTCAAAAGAAGACATTTATGCAGCCAAAAAACACATGAAAAAATGCTCACCATCACTGGCCATCAGAGAAATGCAAATCAAAACCACAATGACATACCATCTCACACCAGTTAGAGTGGCAATCATAAAAAAGTCAGGAAACAACAGGTGCTGGAGAGGATGTGGAGAAATAGGAACACTTTTACACTGTTGGTGGGACTGTAAACTAGTTCAACCATTGTGGAAGTCAGTGTGGCGATTCCTCAGGGACCTAGAACTAGAAATACCATTTGACCCAGCCATCCCATTACTGGGTATA

>ORI-191

ATATACACAGACGTAAACACACAACCTTAAACATAGTTCTTTAATTGGGTTTATTTATTTATTTAATTATTTTTTGAGACGGAGTTTCGCTCTTGTTGCCTGGGCTGGAGTGCAATGGCGTGATCTCGGCTCACCGTTCAAGCGATTCTCCTGCCTCAGCCTCCCGAGTAGCTGGGATTACAGGCATGCGCCACCACCCCGGCTAATTTTGTATTTTTAGTAGAGATGGGGTTTCTCCATGTTGGTTGGGCTGGTCTTGAACTCCTAACCTCAGGTGATCAGCCTGCCTTGGCCTCCCAA

>ORI-192

AAGCATACCATACTCCGGACCATTGGTTCTCACACCTGAGTCCACCTCAGAATCACCTGGAGGGCTTGTTAAAATACAGATTGCTGGGCCCCGCCCCTAAAGTTTCTAGTTCATAGGTCTGCTGTGGGGCCTGGGAATTGGCATTTCTAACAAGTTCCCAGGTGATGCTGCTGTTGCTGGTCTGGGGATTACTGGCATCTGATGGTCTTGTAGCTCTTGAAGCCAAGATTACAGGGATAAGGGAAAGAGAAGCAGATATTTGACCTCTGTCACAATCCAACTCTGTTAGCACAAACCCCA

>ORI-193

GGGCTACACAGTGCCAGGAGGGAGGAAATCAGAGTGGGCATGGAGGCGAAAGGCTGTGACTCTATCTGACACTCAGTTCCCTGCCCCGGGGACTTGTGAGTCGGAGTGCTTCTGGGTCTGTGAGAGGATGGCCACTGCACTTCCTGGCCTTCCCAGAAGCCACACGCCTCCCCTCTGTTGTTTCTTCGTGGTCCTCCACAGCCTCAGGAGGGGAGTCACAGATATGTCCTTCTGTAGGGCAGGTGCTTTATGCCTTTCCTTCTTTTTCAGCTACGGCATCCGGAATCGAGACGAGGTTTC

>ORI-194

AACCATGTTGGCCAGGCTGGTCTCGAACTCCTGACCTCAGGTGATCCACCCGCCTCGGCCTCCCAAAGTGCTGGGATTACCGCCACCGGCCTTGTTCGTGTAATTTTGTATTTTCTCATGATGAATACATCCCAAACTCATTTTATTTTATTTTACTTTATTTTTTAAAGGCTGGAGACTGATGAAATGTCAGCAGTAGTTGCTTTGGTGTGGTGAACAATGCATGATTTTAATTTTTTTTTTCCTTTTTCTACTTTTCAAATACTCTACAACGAGCAGATCTCGAATGCCCAGCAAAAG

>ORI-195

GAGGTCCTGTGGCCAGAGGAGACGGTGGAGGGGCTGGGGGCACCAGGCGTGCTGGAGGCGGAGGGCGGGAGATTTGGGGACCAGGCTGCACAGAACCCGTCGGAAGCAGGGCGATCAGCCGGGAGCTGCAGAGGCCTGGGGGGCCTCTAGCCCAGGGCAGCCTGGGAGGGGCAGCTGCCTGGGCACCCGGGCCCCGCGAGGAGGGGCTGGGGCCTGCTGCGGGGTCGCAGATGTGTCCCGGTGCTCGGAGAGGGCCGCAGGGCGCGTGGGCCGTGGCGGGAGGCCGCGCTGCTGGGAGCT

>ORI-196

AGCTGCAGAGGCCTGGGGGGCCTCTAGCCCAGGGCAGCCTGGGAGGGGCAGCTGCCTGGGCACCCGGGCCCCGCGAGGAGGGGCTGGGGCCTGCTGCGGGGTCGCAGATGTGTCCCGGTGCTCGGAGAGGGCCGCAGGGCGCGTGGGCCGTGGCGGGAGGCCGCGCTGCTGGGAGCTCACGGCCCCCGCCCCCCGTCCCAGGCTCTTCCTCAGCCACCCGCAGACCAAGACCTACTTCCCGCACTTCGACCTGCACCCGGGGTCCGCGCAGTTGCGCGCGCACGGCTCCAAGGTGGTGGC

>ORI-197

CAGCGACACGGGGGGAACAGCGACACGGGGGGAACAGCGACACGGGGGGAACAGCGACACGGGGGGAACAGCGACACGGGGGGAACAGCGACACGGGGGGAACAGCGACACGGGGGGAACAGCGACACGGGGGGAACAGCGACACGGGGGGAACAGCGACACGGGGGGAACAGCGACACGGGGGGAACAGCGACACGGGGGGAACAGCGACACGGGGGGAACAGCGACACGGGGGGAACAGCGACACGGGGGGAACAGCGACACGGGGGGAACAGCGACACGGGGGGAACAGCGACACGG

>ORI-198

CTCCCCTATGTGCTCCCCACCCTTGCACAGCGCGCTCAGCAGGGCTCTGAGAACTGCCAGACTCCAGGAAAACCCAGGCCTAGGCCACCAGCCCAGTGACCCCAGGGCCCAGTGGGAGGCAGGGCGCTCACCCTCCTCCGAGACGCAGAACTTCTGGGCCAACCACTGCACGACGTCGCTGCCTGCACGGGAGAGACAGAGGTGGAGGGAGGCCGAGGCGCGCACGCACCCCCGCCCCGGCGCGCCTCACCTGTCACCGCGTGGGGAATGACGGTGACCAGCAGGCGCTGGCTCCGCATC

>ORI-199

GGGCAGGGACTCAGAAACCCCTGCCCAGGGCACCAGGGAGAGGGGCTTCAGGAGGCCACCAGCCCGGGGCGCCATGGGAGGCGGCGCAGAGCCTGAGCCCAGGGATTTGGAGACAGCCGCCCCCGGCACCCGAGCACCATGGGGAGGAACTCCTAGACTGTGGGAAAGTGTTCAGAGACCCCAGTCCAGAGCAGGGTGGTGGAGGGCCCTAGAGGGTCAGTAGGCTTGGGGGGCCGCCGAGGGCCCTAGAGGGTCAGTAGGTTTGGGGGCCGCCGAGGGCCCTAGAGGGTCAGTAGGTTT

>ORI-200

GGGCGGCCCCCATCTCGGCGGCTGCGGCTCGGCGGCCCGGAGGCGGACGCGGGGCAGGCCGCGGGGGCGCCGCAGGGGCCCAGCCGCCAGCTCCCACCCGCCCGCTCCGCGTGGACGCGGCTCCGGGCCGACTCCGGCCGGCTCTGGCGGCGGCAGCGGCCACGATCGCCTCCCGAGCCAGAGCCCGAGCCAGAGCGCCGAAGCCCAGGCCCGAGCGCCCCCGCCGGCGGCGTCCGGAAGTGCGGGGGCGGGGCCGGGGCGCGGGGACGAGGTGAGAGCGCGGGGCGGGGCCTCAACACG

>ORI-201

TGGCCAGGGTTCCAGCTTGGGTGGCTGGTGGGTGTCAGAGGGACAGGGACTCAAGGGAGCCTGGGAGCCCCACACAATCCAGCCCGTGCCTGGGGCCAGCAGAGGGCTGGAGTTACAGACGGGCACTGGTGAGAGGGCGAGGGCGGCCAAGGACTGAAGGTGGAGGCGGAGCCCTAAGCAGGCCCGCGGGACACACAGTGGTCTCCCGGCAGCTCCCTTGGGCTCCCAGCACCCTGACGGGGGCACAGGACAGGAGCAGAGTTGCCAAGCGACCCAGAGGGCCCGCCCGCTCTCCATGCC

>ORI-202

CCGGTGAGCTCACGGCCCAGCCACTCCTCCCAGCTGGAGTCCCGGGCAGAGTCCCCTGGGCCCCTCCACTTCCCCGCGCCGGGCGCGCCGGACCTGGGCGCTCTGGGAGGAGGAGGGAGGGGAGGGGGCGCTCCAGGGGCCGATGTGGCCCAACCCCGATCGGGGGACCGAGGAGGAAGCGCGGGGTCCCGTCCGCCTCGACCCCTAGGACACGAAGCGCCCCAAAGGGCCACTGTGCACCCCCAACCCGCGGCGGGGACCCCCAACCCCGCGCCGGCGCCAGATGAGCGCACAGGTGCG

>ORI-203

CACTGTGCCCAGCACATGGGTGGTACACTAATTATGACTTCCCCCAGCTCTGAGGTAGAAATGACGCCTTTATGCAAGTTGTAAGGAGTTGAACAGTAAAGAGGAAGTTTTGCACACCCAGTTCCATAACGTTGTTGGATGTATGAGCCAGGCAGGGGTCCAGAGACCACCAGCCTGCAATCCGGAGAACCCAAGCTAGGCGCGAGCCGGTGCTGTGATCCCGCCAAAGGAGAAGCTGCCTTCCCGGGGAAGGGGCAGAGAGGGGCCGGCGGGCTGAGGCCAACCAGCGCAGAAAGGGCC

>ORI-204

GGGAGGAGGTGCAGGGGCCTCAGGGAGGAGGTGCTGGGACGTCAGGGAGGAGGTGCAGGGGCGTCAGGGAGGAGGTGCGGGGGCGTCAGGGAGGAGGTGCAGGGGCCTCAGGGAGGAGGTGCTGGGACGTCAGGGAGGAGGTGCTGGGGCCTCAGGGAGGAGGTGCTGGGGCGTCAGGGAGGAGGTGCTGGGGCCTCAGGGAGGAGGTGCTGGGGCCTCAGGGAGGAGGTGCAGGGGCGTCAGGGAGGAGGTGCAGGGGCGTCAGGGAGGAGGTGCTGGGGCCTCAGGGAGGAGGTGCAG

>ORI-205

CTTGGTCGAAGAAGATTTATTTAAATAAATGATGTTTATGCTGAGTGCTCAGGGTAAATAGGTGTTAATCAGGTAATAGAATTCTCATAACTTTGCAATATATGTTTGTATCTATCTTTTATTTTTATTTTCAGATGAGAAAACATAGAGAAATGATATAAGTTTTGTCATATAGTGGCAGAACTGGGACTCCTGAGTCCTGTCTCTCCAAAGCCTTTGAACTTGACCACCACACCATACCCATATGCTTCCTGCTACCTGGAGTGCCTAGTCTTCAGTTTTACTAAAAGATCATCTAGA

>ORI-206

CCCTCCCCACCCCCTTCTTCGCTGCTCTCCGCTCCCCGCCAATGGAGAGCGAGCTGATGACAAATAGCGGGCCGCGGAGTCCGCGGGACTCGCACCAGGAGTAATAAAACAGACCCAGAGATCAAGGAGCTGGGGAGGGGGCGGGGGAACAGGGAGGGAGAGCGTGTGAGCGTCTGCGAGTGTGTGGAGGCGGCTGCTGTGGCAGCGCAGGCGGCTCGGCTCCGGCCCGGAGCGCAGCGGAAGCCGCGAGGGATGCAGCGGCGGGGACCTTGGCCGGTGGAGGATGTGGAGGTGGAAGTG

>ORI-207

GAAAGACCAAAGTCCCATGGAGTTACTGTATCCGACCCGAAGTGCAGGATCTGTGAGTCATGTGCTGTGCATTCCACCATGTTCACAAGTGGGTCCTTGATGTCCAGTGATCTATAAGAAAATCATTTTTTGACCTCTTGGGCCACATTCAATAAAGAACAGTCAAATATGATTGAAATAGTCTCAATAAATTTTTTTATTTGGCAAATGGAAGATGGGGAAAGACACAGAAGTCACTGGTTGATATTGTTAGGCTTTGTGTTTCCAACCAAATCTCATCTTCCCCACTTGTTGAGAGAC

>ORI-208

CCAAGAGACAGAGATCCTAGCCCCCCCATTGAACTGAGGGGCCTGGTCCCCCAATCCGCTGCGACCCAGGTGTCTAAGGTTTCATCCGGCAAGGAAGGGGTGGGGGCGGAACAGACCAGGAAGGAGGGGGTAGGAAGGGAGCGGAAGAAAGGAGTGAGGTGAGACGGGGGCGGGGACCTGAGCTGGGGGGCTCTGCACTAAACAAACCCTAAACATGCGGGGTTTCTATGGTAACGGTCTTCCCTGGCCTACCCGGGAGACTCCTCTAGGGCTGTGTCGAGGAGGGCAGCCCCCAGGAGT

>ORI-209

GTTCGATCCCTAGAGGGAGGAGCCTGTCCAAACGGACGCTAACGGCCTACCTCCCCCTCAGGTGCTCTTACAGCCTGTTCCAAGTGTGGCTTAATCCGTCTCCACCACCAGATCTTTCTCCGTGGATTCCTCTGCTAAGACCGCTGGTGAGTAGGCAGCGGGCCTGGACTCCGGGGTCCGAGGGAGGAGGGGCTGGGGGCCTGGACTCCGGGGTCCGAGGGAGGAGGGGCTGGGGGCCTGGGCTCCTCGGTCCGAGGGAGGAGGGGCTGGGGGCCTGGGGGCCTGGGCTCCTGGGTCCGA

>ORI-210

GCCGCCGCCCGTGCCTCCGGCTGCCGGCGCCCCTGCCTTTGGCTCTTCCTCCCCACTCGCCCGCTCCCCCTGGCGGAGCCGGCGCGCCCGGGGTGCCGCTCCCTGCCTGGCGCGCTCCGCACCTGGAGGTGCCTTGCCCCTCTCCTGCCCACCTCGGAATTTCCCTGTGGCTCCTTTGATCCTTCGAGTCTCCAGCTCCTCTCCCTTCCACCTGTTTCCCCCAAGAAAGGCAGGATCCTGGTCCCTGCTACGTTTCTGGGGCCATGGCTGGTCTGGGCCCCGGCGTAGGCGATTCAGAGG

>ORI-211

AAATTAAGCCAAGGACAGGGTAGGAGGGTGGCCATTTTCCTCTGTCTAGCGATTCTCATCCTTTCCTTTCTTGGGTGCTGTGTCTCTTGGGAGCATTTCCTTATCGCTGTGTAAGGTCTAACTGCCTCTGGCTCTTTCTTTCTCCTTTCCACAGCGGGTGCGGATGGGCCCCTCTTCCTCTCCCATCCCCTCCCCTTCCCCTAGTCCCACCGACCCCAAGCGCTGCTTCTTCGGGGCGAGTCCAGGACGCCTGCACATCTCCGACTTCAGCTTCCTCATGGTTCTAGGAAAAGGCAGTTT

>ORI-212

CCGGCTCCTGGCTCCGCGGCGGCTGCAGGACCCGACTCCGCTCTGCGCCCGCCCGCTGCTGTAGGGACCCCGGCCCGGGACGCCCCCTCCCGCTTCCTGCAGCCGTCCAGACCCCAGGCTCCGCCCCAGGCCTGGCTAGAGCCCCAGTCCTCGTCAGGGGACCCCCAGGATTGTCCCTGCTGTCTCTCCTTCTCAGAGTCTCTCTCTGTCTCTCTCTGTCTCTCTGTCTCTCTCTCTCTGCCTCTGTCTCTGTCTCTTCTCCAAGCTCAAGGTCTCTGGGTCTCAGCATCTCTCAGCCCC

>ORI-213

GGAGGCTGCGCCAGCTTTAGGCCCCGCCCTCCTCCCAATGGCTCCGCCCACAGACTCCCTTATTTCAATGGCCGCGCCCTCTTTTCCCGACCTCTCCTTTTCATTGGTCCCTCTCACTCCCAAATGACTCCTCCCCTTCGTTGGCCCGCCCCTTTCCTCTGGCCCCTCCTCTCCAAGAAAATTAGCTCCTCCCTCGTTCTCCACCTGCTCTGAGCTGGGAGCAGCCAGAGGCGGTGCAAGCGCCCAGCTCCCCAGAGCTCCCCAACCTCGGACCTCACCGCAGGGGCGCTGGGCTGGAGA

>ORI-214

CTCCACTCCCCGAACCAGGTCGGACGCCTGGGTCCCTCACTGCTGGGGGAGGGGGAAGCAGCGAGAATTGGGAGCCAAAAGGTTTTTCTGGGGGGGGTGCCAACAGGGGGGGCTCGACGTCCCCTCCTCCGTGCATCGGAGAGTTGACCTATCATTAACAGAGGTGAGACAGATCTTTTTATTAATCGGAGGTGGGTGGAGGTAGAATTAATGTTCTGTGATCCCCCCCCCCGAATTCTGGAGAAAAGAGCTGGTCCCCTTCCTAAGGAGAAAGGAGAGTGACCCCCTCATATCCGGAAG

>ORI-215

AGGAGAGAGGGGAGACAGAGCCAGAAAGATAGATGAAGCCAGCAATAAAACTGGGTGGGGTGGGGGCGTGAGGAGAAGAAGGGAAGAGAAACTCAGAAGTAGATTCAGCCTGGCAGAAACGGGAGGAGGAGGAGGAAGGAAGGAATGTAGAGAGAGAGAGAAACCCAGATAGATGAAGCGAGGCAGAAAATGGGAGGGGGTGTGGAGAGAGGTAGTAAGTGATTTGCGGGATATGGAGAAAGAAATTCGCAGACGAGAGGAAGGAAGGGACGAGAAAGAGAAAGGGAGAGGATCCATGGA

>ORI-216

CAGGAGTCCAGGCCCCCAGCCCCTCCTCCTTCAGACCCAGGAGTTCAGGCCCCAGTCCCTCCTCCCTCAGACCCAGAAGTCCAGGCCCCAGTCCCTCCTCCCTCAGACTCAGGAGCCCAGGCCCAGCCCCTCCTCCCTCAGACCCAGGAGTCCAGACCCCAGCCCCTCCTCCCTCAGACCCAGGAGTTCAGGCCCCCAGCCCCTCCTCCCTCAGACTCAGGAGCCCAGGACCTAGGAGACCAGGTTCCTCTCCTCTCAGATCCAAGATTCTGGGTACCCAGGCCTTCTTCCTCTACCACT

>ORI-217

TCAGGAGGCTGAGGCAGGAGAGTCGCTTGAACCCCAGCTACTCGGGAGACTGAGGCAGAAGAATCAGGAGGCGGAGTTTGCAATAAGCTGAGATCGCACCACTGCACTCCAGCCTGGGTGACAGAGCAAGACTCCGTCTCAAAAAAAAAAAAAAAAAAAAAAACAAAACAAAACAAAAACAAAAAACTAAGGAACCATATTCACCCTACAGTGCTGCGAACACCAGAACGCGTTTCTCCCGTCTAGCTGTGATTCTATATCCGCCTTTTAATCATTTTCCAGCGTACGGTTCTGTGGCAT

>ORI-218

TGTAGTTTCCTGAGGCCACCCTAGCCATGTGGAATTGTAAGTCAATTAAATCTCTTTCTTTTTTTTTTGAGACTGAGCCCCCCTGTCATCCAGGCTGGTGTGCAGTGGTGCAATCTCAGCTCACTGCAACCTCCGCCTCCTGGGTTCAAGCGATTCTCCTGCCTCAGCCTACCGAGTAACTGGGACAACAGGCATGCGCCAATAGCCGGCTAATTTTGTATTTTTAGTAGAGGTGGCGTTCACCATGTTGACCAGGCTAGTCTCGAACTCCTAACCTCAAGTGATCCGCCCACCTCAGCC

>ORI-219

GTAAGGTTTCTGCACCTAAGGGAAGTGAAGAGGCAGCGGACTCAGAGCTCAGAAAGTAGGGTCACGAGGCTCAGGTCGGAGTGTCTGCTGGCCCTTAGTCAGCTCCTTTCCCACCTTTGAGAGCCCCCCTGCCAACTGCACTCTCTACAGCAAATGGAACGGTTCAAAGTTGTGGAACGAGAGACCAAAACCAAAGCTTACAGCAAAGAGGGCCTGGGCCTGGCCCAGAAGGTAGATCCTGCCCAGAAGGAGAAGGAAGAGGTTGGCCAGTGGCTCACGGTGAGTTGGGGTAGAGAAGAG

>ORI-220

CCCTGGTTCAAGCAATTCCCCTGCCTCAGCCTCCTGAGTAGCTGGGATTACAGGCGCCTGCCACTAAGCCCGGCTAATTTTTTTTGTATTTTTAGTAGAGACGGGGTTTTGCCATGTTAGCCAGGCTGGTCTCAAACTCCTGACCTCAGGTGATCCACCCGCCTTAGTCTCCCGAAGTGCTGGGATTACAGGCGTGAGCCACTGCACCCGGCCTACCTGCCTCTCCTTTTTTCCGAACCAGGAGTCTGAGCCCCTTCCTCATCTAGGACCCCGGAGTCTGAGTCCCCAGATCCTCAGACA

>ORI-221

CCGCGCACCCCAGCGCATCCCCGGCAGAGCCACAGGCGGTTGCGCCAGCCCCGAGTTCCAACGCGCCTCCGGGGCCGCCCCGCACCCGCCAGCCCGCAGAGACCCTGCCGCCGTGTAACCTCGCCTCGCCACTGGGCGCCGCCACCCTGGCCCACCTGAGCTGCTCGCCGGGCAGGAGGCGGCCGAGCAGTCCCAGCCCGCTTGCCGCCGCAGCTCCGGCCACGCCTCCCCCGCCCAGCGCGCCCCCGCGCCGCCTGCTCCTTCTGGGCGCCCGCCGGGCTGCGCAGATCAGGCCGGGGA

>ORI-222

AAACGACATCAGGATGTCATCTGTATTGTGCCAAGAATTGGGGGAATCAGAATATACATTCATATTGACTCATATTCACCCAGAAACAAACACTGGAAGGATTAATATGAGAAAGTTAAAATTGTGGCTCTCCCACGTTTTCTTCCCCGTCCACCACCTGAACAAGCTGCGGTCCTAGAAATCACCTCGACTGCTCCTCTTTCTCACCCTCTCCCCTTGTTCTACCTCCAGAATCTGTCCTCATGCAGACACTGCTTGCCAGCACCACGGCGCCAGGTTCACCCTCTCTCTCCTCGTCCA

>ORI-223

TCTGTGCCCGGTCCTGCCCAGGGTGGGGTGGGGGTGCAGCCTGCCAGGCGAGACCCAGCCCTCTGGAAGGGAAGCAGGGCTCCTGGCCACTCCCCAGCTCGGAGCCCTCCTGGAGCCCCGCCACCCGCCAGACCCTCATCCCTTCTGCTCCAGCTGGCAGCGCCTCTCCTGGTGGGCTGGAAAAGAGGGCAGGATAAAGATAATGGTGCCAGGAAGAGAGAGGAGGGTCAGGAGAGGGTGCTCCTGGGCGTCAGGAATGGGGAGTTTGGCCCCCTGGAGAAAACTGGGGAGCACCATTTA

>ORI-224

CATACTAAACATGTAGACTTTTTTTTTCTCGTCACTACTCCCTAAATAATACAGTATAACAACTATTTACATAGCACCTATATTGTATTAAATGTAAGTAATCTAGAGATTATTTAAAGTACATGGGGGAATATATATAGGTTATATGCAAATACTATGCCACTTTGTATCAAGGACTTGAGCATCCACGGATTTTGCTATCCGAGGGAGGTCCTGGAACCAAAGGACCAGGGATACTTGGGGACAATTGTACTAGGAGTCAAAGATTCTAGTTTAAATTCAGCTACTCTAACATTATCT

>ORI-225

ACAGCTCACCGCAGCCTTAACCTCCTGGGCTGAAGCAATTCTCTTGCCTCAGCCTCCAAAGTAGCTGGGATTACAGGCACGCACCACTATGCCTGGCTAACTTTTTAATTTTTTTGTAGAGACGGGGTCTCACTATGTTTCCCAGGCCGGTCTGGAACTCCTGAGCTCAAGCAGTCTTTCCACCTTGGCCTCCCAAAGTTCTAGGATTACAGGTTGAGCCACCGTGGCTGGCCAGTGAATGTTTGTTGAGTGATGCAATACTTAAACAAATGACCATACAGTGTGTTAAGGTTCTTAGAG

>ORI-226

TGGAGAGCAATTTAGGGTAATATTCAGAAAGCAGCGCTCAGGAGAAGCACTGGGACGCTGTTTAGGTAAAATCAACCCAAAAGACCCTGATTCCCAGAGTACTTTGAGGTCCAGAAGAACTGGGCTGGAAGCAGGTCCTGCTATTAATTGGCTGTGATCTTGGGCAAGTTATTTAACTTCTCCAGGTCACAGTTTGCTCTTTGGAAAACGGAGATAAGTCACACCTGCTTTCAGGGCGGACACCCTTACTTTACATCAGAATCATCCGATGCCATTCCTAGCGTTTCGGCTTCAGTAGGT

>ORI-227

GAGGTAGTACAGGAGAGAGTGACAGGCAGTTGCCCAAGCTGTTGAAAAATGGCATGGGAAAACTGTTCTGGAGGTTCCATATCGATATCTAGGTGACTGAAACAAGCAGGGCTGCTTTGCCCTGAGCTTCAAAGCTGGGGATTTTTTTTTTTTTTTTTTTTTTTTTTTTTTTTTTTTTTTTTTGAGGCTGGAGTGCCATGGCACAATCTTGGCTCACTGCAGCCTTGACCTCCTGGACTCGAGATCCTCCCACCTCAGCCTCTCAAGGAGCTGAGACCACAGGCACATGCCACCATGCCT

>ORI-228

GGGGGCTAAGCCGCTCTGAAACGGAAGAAGTTGGCCAGCAGCTGCGGACGCGCCGCTGTGGAACACGACCTGGGGCGCCTCCAAAACAGCTCAGCGCCCCAAGGAGCTGGAAATCGCGGGAGCCCGGCCTCGGGGGACGCAGCCTGAGCCCTGGGGGCGGGGTCGGGATTGGGGCGGAGCTACGGGCGATCGGCCAGGCCTGGGGGTCGGGGAAAGGAGTGGACCGGCAGTGTCAGCGTGTGCACAGCTGTAGAAGGGTGGGACTGGGCTGGTTACTGGACAGGCGGTTGTGGTCAGAAT

>ORI-229

AATCCCAGCCACTCAGGAGGCTGAGGCGGGAGAATCGCTTGAACCCGGGAGGCGGAGGTTGCAGTGGACCAAGATCGCGCCATTGCACTCCAGCCTGGGCAACAGAATGAGACTCCATCTCAAAAAAAAAAAAAAAAGAAAGAAAGAAAGAAAGAAAGAAAGAAAGAAAGAAAGAAAGAAAGAAAGAAAGAAAGAAAGAAAAAAACTGTTATAGACTGAGTGCCATTTTAGATGGGGTTTTCTGGGAAGTGCTGTGACATCATCGCTTGCTGTAAAAGAGGCCGGGCGCGGTGGCTGACG

>ORI-230

CAATTTATAGAAAGGAAATGAAACCAAATGCAGGTTTTCTAAAGGTACTCACACACTTAAGAACAACCACTAAGAAGGCAAAACAGTGAAAAAATAAATGTAAAAACAAAAGTCGTCTTCTACTTTAATTGAATATTGAGCTTTCAATTTAGAAGAAAATCATGAAGCAACCACAGGACCTCTTCGTACTTTCAGATGTGGTACTACCAGGGTCTAAAATCTATCTTCTCAGTTACAGTCTCACTTCGTATATATATATAAGGTCAAAGGACCAGCCAGGTGAGGGGGCTCATGCCTGTA

>ORI-231

ATACATGCAAATATATGGGGGGAAATTATGGGACTTGATGAATCAGAGGGTTGGTGTACAGAAAGGGAAAAGAAGTAAGAAAAAGTGACAAGATGGGAGATAATTGGGTGACTATTAGAAACCAATGTTAACTACAAGAATTCAAAGTCCAGGTGTAGCCCAGAACGAGGTTTCTTCCATTTGGTGCCTCCCCTGTCTTCTGGGGCCTCAGAGTCTGTCCTTGTCCCCTCTGTTGTCCACTTGTGACAGCAGACGGGGAGAGAGAGGTAGTGGAGGGTCACAAGGGGTCTCAAGGGCCAG

>ORI-232

ATGATTGTGAGGCCTCCCCAGCCATGTGGAACTGTAAGTCTAATAAACTTCTTTCTTTTGTAAATTGCCCAGTCTTGGCTATGTCTTTATCAGCAGTGTGAAAACGGACTAATACAACTCCTTTTTGCATTTTTGCCACAACAGTTTCCTTCAGTACAACAGCTACTTTTTAAGGAGTGCTTTCTGTCAGGGACCAGGCCAGGCACTAGGAATATTCACATGAAGAAGGCAAAGCCCAGGACCTTCCATCAGTTGGTGAGGCCTGTGATTGGCCACAGCTGCCTGCGTCTCATTCTGCCG

>ORI-233

GTTAGCTCTAATATTTCTCATTGAACTTGGTGGTATGTGCCTTCCCTGCATATAAGGCCATAGTGCTTTTTTGGGAGCGCTAGAATATCCATCCACTTGACAGTGACCACAAAATAGGCTGTTTCCAGCTGCAAGAACCTCCTCCTGGAGAAAAGCGTATGAAGGTGCTGGAGTGAAAAGTAACAAGGTTGTGTGTATTTGAAGGGGTTGGGTGTGGAACGGTAGAAAATTCTGATTCTGTGGTGAGATTATTCTAGTTTTGAAAACAAAATTAATGAAAGGTCCAAATTACATTTGCAG

>ORI-234

CTTAATTCAAAGACACTGGGTCATGTTGAAGTTGAAAAAATTCTTGTCTATTACCTTTTTTTCATTTGTGTTCCCTAGAATTCAATAAAAATATATGCTATCACTCCTGTCCCTGTCAACTGCTCTGCCTCAGTTATAATATAGCAGAGAGTTATTTAACCTAACCCAGACTAAAAATTATCACCACATCGAGAAATCACCTGGTAAAAGTATTTACTTTCTTTCCTATAAAGCATTCTCTCTCAGTGATTTTTGCATTAGTTTACTTTAGTCACAAAATGATGCAAACCAACAAGGAAA

>ORI-235

TGCAGGGCGCCCGAGCTCTGCAGCGGCGTTGCGGGCTGAACCCATCCGGCACAAACTGCGGGCCACTGGCCCCTCACACCTGGGAGTTTGCGGCGCTGGCCTGCAGCCCGGGGCCCACGTGGCGGAAGCTTTCCCGGGCGCGCGCTGCGCAGCCCCGCGGGGCCGGGGAGACACCGCTCGGGAGTCCTCCGCTCGGCTGCAGAATCTTTATCAGCTGCACTTTACCGCAGCCCTGGCTAGGACGCTAGGCGGTGGAGCGCCCTATCCAGGTGCGCCGCCGCACCATGGATCACCGCGCCC

>ORI-236

CTGGTGTCCAGCCGCCCGTCGTCGCCAGAGCCCGATGACCTTTTTCTGCCGGCCCGGAGTAAGGGCAGCAGCGGCAGCGCCTTCACTGGGGGCACCGTGTCCTCGTCCACCCCGAGTGACTGCCCGCCGGAGCTGAGCGCCGAGCTGCGCGGCGCTATGGGCTCTGCGGGCGCGCATCCTGGGGACAAGCTAGGAGGCAGTGGCTTCAAGTCATCCTCGTCCAGCACCTCGTCGTCTACGTCGTCGGCGGCTGCGTCGTCCACCAAGAAGGACAAGAAGCAAATGACAGAGCCGGAGCTG

>ORI-237

TCCCAGCACTGGGATTCAATAAGGGCTGTTGTGTCCCAGAAGCTTCTAAGTGATGTGGGTAGCTCCTTAGAGAATGCCAAAGTCATAGGACCTGGAGACTTCAAAATCACAAGAGCAGATGAGAAGACTTGTCTGTGAGGTTAAAAAGAAAGAAAAAAGAAAAGCCAACTCACCTGGGGGCAGGCAGGTGGGAAGGAGAAGCTACTCTGCAGAAATGGCAGAGCATTACTACCAGAAATAAAAATAACAATGGTGCTGATAAGAGCTCATTGTGTTATCGGGCACCAAGCAGGGTTGTAA

>ORI-238

GACAGGTTGCAGCAATGTCTCCTCACTCCCCCATCTCTAAGATTCCAGAAAGAAACCAAAACAAATGGAAAGTCAAAGCCTGCCCTGGAAGGCTCGTTTATTTGTCTGTTTGATTTTTAGAGACAGGATCTCGCTCTGTTGCCCAGGCTGGAGTGCAATGATGCAATCATAGATCACTGCAGCCTCAACCTCCTGGGCTCAAGTGATCCTCTTGCCTCAGCCTCCTGAGTAGTTGAGACCACAGGCACACCACCATGCCCAGCTAATCTTTTCATTTTTTGTAGAAACAAAGTCTCACTA

>ORI-239

TTCACTGGGCCCTTTCTAACTCCTTCCCCTTAAAACCCTTTTCAAAATGGGGTTGACTGGAGGCGGAGGTTGCAGTGAGCCGAGATCGCCCCACTGCACTCCAGCCTGGTGACTCCGTCTCAAAAAAAAGGGGAGGGGGGCGGGGGAGAGTTGAAAGCTTAATATGTACTTTGGGGGCTATTAAAGCAAACATTTCGACTAAAGGGGCGAATCCTCGAATTGTGCGATCAAGCACCCGAGAGGAGAGTTGGGGGGGGTCAGGAGGGGTGGGGGCTCCAGGGAAAGCCCGGGGGTCTGGGC

>ORI-240

AACTTACTGTTTTAAGTACCTTTAACCAGCCTATTGGCTAAAAACCGAAACGGTCCTACCCGCGACTACCACTGCTCCAGTGACAATGCTCTGCTGCTACTTATTTAACGGAGCTCAAGGTTTATAATTCTCGAAAGAATGTTGTTCCCCTTCCCTTTCCCCTCCCGCCTCTGTACTTGACTCTCGTAAGAATGAAATCAGAAAAGTGCAGAAAACACAGGCTCCTAGCAATGGTTCGGGGCAAACGGGAGGGGCGAAGAAGGGCTGGAGCCGCCCCGGGGCCCTCGCTCCGGCCTCGGG

>ORI-241

CTCTGCTGCTGACCGGTCTATGATGTCATCGTACTCTGCAGCTGACCGATCTATGATGTCATCTTATACTGCTGATCGTTCAATGATGTCTATGGCTGCTGATTCTTACACCGATTCTTACACTGACACATATACAGAGGCATATATGGTGCCACCTTTGCCTCCTGAAGAGCCCCCAACAATGCCACCGTTGCCACCTGAGGAGCCACCAATGACACCACCATTGCCTCCTGAGGAACCACCAGAGGGTCCAGCATTGCCCACTGAGCAGTCAGCATTAACAGCTGAAAATACTTGGCC

>ORI-242

TACTCAACCTGTATATGAATTGGGAAAGCAGTGTTTGGGTTTAGCAATGTAGCTAATCCCCTTTGTAATAAGTCTATGGAGAGAGAGGTTATGTAGAAAGGTTATGTTCATATTTTGAGAACATCTCCCATTTGTTAGGAAGGGAGAGGAGAGTTTGCTAGGAAAAAAGGGACCTATTTTATTTGGAGACAACCGTTTTCACCTTTTTGGTGGCTTAAAATCCTAACCTTTTTTGGAAGAGTCTTAACTGAAGCTTATTTTTTTAAGAGACAGTAAATCTGCAGAAATCACTTGGCAATA

>ORI-243

CCCTTCTTCCAATTGGCTGGCGGTTTCCCCACCCACTTATGGGCGGAGATGGATGGTGGATGGGGGGCGGGGGAGGCAGCGGAGGGAGATGAGGAGATAGGCGGGAGCGAGGGAGCGAGGGAGGGAGCGAAGGAGGTAGAGAAGAGTGGAGGCGCCAGGGGAGGGAGCGTAGCTTGGTTGCTCCGTAGTACGGCGGCTCGCGAGGAAGAATCCCGAGCGGGCTCCGGGACGGACAGAGAGGCGGGCGGGGATGGTGTGCGGGGCTGCGGCTCCTGCGTCCCTCCCAGCGGCGCGTGAGCG

>ORI-244

CCGTAGAGCCCTGTCCACGGCATTCAGGCCAACTCTCACACCCGCTGAGTGGCTGTTTCTCAGAGTGTTTACATCGGTTTCCTCAGCTCTGCAGACCCGGCCTCCTCTACTTCCTCCCTTGCCTTTCTCTCTCCAGCACACGCTCACAAAGAGGAAAAAGGCCACCGCAGTAAGCCAAGCTGTCTGTCCAGCATGGACCATGGGCCGACACCACGCCAGGCCTCCTCTTGAACCTATTCCCTACAGAGGCCTTGCCAGCAGCCACAGAGCCTTGAGCAGGAAGCGGGCGGGGGGGGGGGG

>ORI-245

CACCCCTCCCCCGTCCCTCCGGCCCCGCCTTCCACACCCCTGTCCAGCCCCCTGGTATGAACTTAGGTGCATGGGACACCCTGTGACGACGATCATTAGGACAAGTATGCACTTGCTTATTGGCCTGGGAGTCCCTTGGCTTCTCACAAGCTTGAAGCGCTGCGCCCCCATTGTCCCAGTGCTATGACAAGCCATGCAAAGGTAAACAATAACTAGTGGTCACAAGGCGGGACAGTCCACTTTCCCCAGAATATCTGCAAAAATGCATGCCAAAGGTAGTAAGCCATTTCCATTTTCCGA

>ORI-246

GGGCCCGCCGATAGGTACCGGGCGATAAGGAAGTACAGCTCTGCGGGAAGACAAGGAGTCAGGTCAAGCCCCGGCGGGGAGGGGAGGGGGACGGGGCCAGGGGAGCCGGGGGAGCCCGGGGAGCCGGGGAAGCCGAAGCAGCCGGGAGCGCCAGGGCGGGGGTGGCACGGCCTCGCGTCTTACCCGACTCGATGAGAGGCACCGGGCGTCGGGCGGACGACGGCTCCGCCATGGCCGGGCGCGGGGCGGGAGGCGGGAGCGAGCGAGCGAGCGGAGCGTGTAGGCCGCGCCGAGGCCTGA

>ORI-247

CCGGACGGGGTGGCTGCCGGGCGGAGACGCTCCTCACCTCCCAGATGGGGCGGCTGCCGGGCGGAGGGGCTCCTCACTTCTCAGACGGGGTGGTTGCCAGGCAGAGGGTCTCCTCACTTCTCAGACGGGGCGGCCGGGCAGAGACGCTCCTCACCTCCCAGACGGGGTCTCGGCCGGGCAGAGGCGCTCCTCACATCCCAGATGGGACGGCGGGGCAGAGGCGCTCCCCACATCTCAGACGATGGGCGGCCGGGCAGAGACGCTCCTCACTTCCTAGATGTGATGGCGGCTGGGAAGAGG

>ORI-248

CACCACCAGGCCTGGGCACTGGGGCTCTCACTCTGGTCTGACTTCTGGTGCTTTCTCATGCCACAGCTGTACTAGTTTCTGGAAAACCTTGGCACAGGGAGGCCACTCATCCATTTGACAAGCATAGGTAGCCACTGCACACCAGGTCCAAGGGCCACAGCAGTGAACAGCCAGCCAAGGGCCCTGCCATCCTGAAGCTTATATTCTAGTCAGAGACAAGCAAAGAAAAACCCCTGCCTGCAGGCCAGTAAAGTGGCTGCAGATCACGTAGGGTCCTGCAGGCCACAGTGAGAAGTAGCC

>ORI-249

CCTAAGGGCCAGTCACCTGCAACATGAGAACTCAGAGGGCTTTGTGGCTGATGACAACCTGGTCACTGCAATCCTGGCTGCCTTCCTCTACCTCTGCCCTGAGAGCAGGGAAAGGCATTGGCAAGTCACAGGTAGTCTCTAAGTCTTGGTTTGTACATCTGGAAAATGGGAAAGGAAATTCCTCCTGGCCACCCCCTTGAGGCTCAGAGCACAGCTGCAACCATGGCCCAGAACAACTGCTCAGAAACCCCACTTCCACCTAGGAGCGCGACGCAGCCTCAGACCTGGCTTCCAACACCT

>ORI-250

AACTTGCTAAGTTTCTCCCCGCTCCTGGGTGGGGGTGGGGTTTCTCACTTATGGACTCTCCAGCACTCAGACTTGCTTTTGGGGACGAGCTCTGATAAACCTGTGTACACTGAGGTGCAAAAGATGGACAATAGCTCAGAGCAACAGTCTACAGGTGACTTGCAAGGAACCTCTAAGTGTTAATAGCTGTTCCTTGGCAAAAAGAGTTCAATAGTTGAGTGAGTTTGTGAGATGTCATGTACCGTGTTCCCTCCCTGGGGATGACTGTACATGGCATATATTAAAGGCTCAGAAAAGTTG

>ORI-251

GGTGCTACTCTGCCTGTGGTGGGTGGGACATGTGGTATCTGGGGCATCATTGCCTGCGTCTGGGAGCTGGTTGGGGGCACTATCCATGGCTGCACAGCCTACAGCAGGGAGTGGGTTGAGCGCACAATCTGGGGCTGCAATGTTGGTGGTGGGGGACGGGTTAGGGTCACTATAGGGTGCTACACTATTGGCGGCAGGGAGGGGGGTGTTGAGGTTGCTATCGGGGGCTACACTGAGGCAGAAGTAGCGGGGTTTATTGGGAGTGCTACCAGGGACCAGAATGCCCGTGGAGAGGGGGTG

>ORI-252

TCTTGGTCGCGTTGGCCACCTTCCTCCGCGTTCCTGCCTCTGTGCCTCCTCTTCACCGTTTCTCTGGCAGGTCACTGCTGGCCTCTTCTGGAGGCCTAGATCAGCCGTTGCCTCCTGTGTGATCCCACAGCTGGTCTCCCAAGGAGAGCCAGCCCTCTCCTCCCTGTGACCTCTGGGCACTTGATTCTGGATCTCTTGTTTTACCCCATAACCCCACCATTCATGTTTGGCACACGAGTCTTTCACGTGGGTGTGCCGTGCTGGCACGATGGCTTTTTGCATTTTCATCTCCTGCCTGGA

>ORI-253

ATCACACACAGTTCCCAGTGAGAAACAGTCCTTACAAACGCGGTAGTTCTGCAAAGCAGCCTCGAATGCAGATTCCTCTGTGGACATCTTATCCTCTTGTGTTTTTTTGTATTATTATTATTACAAATTACATGGTGATGGACAGAAGCTCACAGCAAAAAGTAAGAGTAGGCAGCTTAAAAAATCAGGTTGAGTTGCTAAAAACCGGTAACTCTAGAAATCTATGCTTGAACTTGTCACTACCCTGCATCTTGTTTCTCAGGGACACTCTACTCATTGAAGGACCTGCCAGCCATAACC

>ORI-254

ATACTTATAAAAAGGCTGAATGATGGTGACCATTGACATCCTCAGTTAACTCTCCTCACCTCTTCGGTGGAGAGAGTGACATTGAAAACCCCAGCTGTGAAATGAGCAAAGGACGCAGATTTAAAGAAATACTCGGCGATTCCAATGTAGAGCATGGAGTTGCTGCGTTCTGGGAGCACAAAAGGAACTGGTGAGAAGGGGGGGTCGGTGAGGTTTTCCAGTGGGTAGAATACACCCTGTGGGAAAAGAGAGAGAAAGACCAGTGTATCAGGTGAAAACTCAAATTCTAAGAAGACTAAG

>ORI-255

AGTGGTGCGATCACAGCTCATTGAGGCCTCGACCTCCGGGCTCAATCGACCCTCCCGCCTCAGCCACCCAACTAGCTGGGACTACAGGCAGGCGCCACCACACCTAATTTTTTGTATTTTTTGTAGAGATGGGGGGGGGGGTTTCACCATGTTGCCCAGGCTGGTCTCGAACTCCTGGGCTCAAACGATCCACCCGCCTCGGCCTCCCAAAGTGCTGGGATTACAGGCGTGAGCCACCGCGCCCGGCCAACCTAAGGATTAACCTAAGGCTTCTCAGAGCAGATGCCGCTGGAACTGAGG

>ORI-256

AGGAAGTGGTTGGGGAAAGGGTGGTGAGAGGTGGCAGACACAGGAGGAGAGAAGAGGTTCCCAGGTCCGAGAGAAAACACCAGAGGGGAGAATCCAGAGAGGACTCAGAGGTGAAACTGTGCCTTTTGACAGTGACATTCCTTCCTTCAAAACACTTTTCCTAAGAAAATTAAGAAGGCAAGGACAAGGGACCCAGAATCCTCCCTAAAGCCAGACAATAATTAAATGAGAGCTAAGGGCCGAGGAGGGCAGATCACCTGAGGTCGGGAGTTCGAGACCAGCCTGACCAACATGGAGAAA

>ORI-257

ACTTCAGTTTAATGTTCTTGGGTAGGAGAAGCTTCTGGAGCTCACCATGGAGCCTAAACTCCATAATGAAGGAGGAGGCCATTCCTCTGTCCAGCTGGCAAGTTTTGATGTTTACCCTAGGGCCCCATGTGGAGCTGAAGGTGGCCCCATCAAATAATAACATAGCTGTTATTTTTGTTTGTTTTTTTGAGACAGATTCTCACTCTGTCACCCAAGCTGAAGTGCAGTGGCATGATCTCGGCTCATTGCAACCTCTGCCTCCCAAGTTCAAGTCATCCTCCCATCTTAGCCTCTGGAGTG

>ORI-258

TGGAACAGTTATCCGACACAATTTCATGTGTTGAGTGTTGATTAACTTTCCATTAAAAAACAAATTAAAATATAACTAGAACTGCTGATACTAGCCAGTCATTATTCTGACACAATCAGCTAATATACTAATTAATTATAGCCGAAAGCAAGGACGGTTGGTGTTTTCATAAGCCTGGGAAGTTTTCTCATGGGTTCCTGGGTGATTGCATTGTCGGATTTGGGTTGGCTTACTGATATGCCTAAAATAGCTCCTGGCTTCTTTGGTATATCGCTGTCCTTTGCCCCTCCTGAGAATCTT

>ORI-259

GTGCCCTGCCACAGGTGATTATAGGTGAAGGTCTTTTGACACCTATATGTCTAAGGGTCATGGAGTGACATCTTATGAAGTCAGCACATAGAAAGTCAAAAAACCCTTACATTCCTGTAAAATACAGTGCAGGCCAGGCGCGGTGACTCACGCCTGTAATCCCAGCATTTTGGGAGGCCAAGGGTGGCGGATCATGAGGTCAGGAGATCGAGACCATCCTGGCTAACACGGTGAAACCCCATCTCTACTAAAAATACAAAAAATTAACCAGGTGTGGTGGCGCACATCTGAAGTCCCAGC

>ORI-260

CTTTACCTCGAATAAATTCTCTTTAATTTCCCCTATGATTTCAATAAAAAGCCCTCAGCTATTTCCAGCTCTAGTGCCCAGCATCACCAATTACCTACCCCAGCCCAGAGGCCCCATCAGCATATCAACTTACAGCAGACTCCCCCTTTCTATAGCTCTCATGCCCCCTCACCTTCATACTATTAGTCTGTTAAAGTTGCTATCAGCCGGGCGTGGTAGCTCAAGCCTGTAATCCCAGCACTTTGGGAGGCCAAGGCAGGCGGATCACCTGAGGTTAGGAGTTTGAGACCAGCCTGGCCA

>ORI-261

TACGGTGAGCCGAGATCGCGCCACTGCACTCCAGCCTGGGCGACAGAGCGAGACTCCGTCTCAAGGGGGAAAAAAAAAAGTAAAACTGTCAAAAAACAGTAGCTGAAAGGAGGAAGCTGAGCAATCTACAAACATAACAGCCCCTAAATAAATGAGCTGGCTTTGAAAGTAAACAAAACAATGAACTGCTTTTATCAGTTTTGGGGGATTACCCTAATATCGATGAAGATGATGGTCTGGCTGGGTAACTTCCCATCTTGCCTTCCCCAAACTTGATGGCTATTTGTCAACTAGCTATCA

>ORI-262

CCAGGAGTTAGAGACCAGCCTGGCAAACATGGCGAAACCCTGTCTCTACTAAAAATACAGAAATTAGCCGGGTGTGGTGGCGCACCTGTAGTCCCAGCTTCTCAGGGGGCTGAGGCATGAGAATCGCTTGAACCCAGGAAGCAAAGGTTGCCGTGAGCTATGATCGTGCCACCACACTCCAGCCTGGGTGACAGAGCGAGACTCCGTCTCAAAAAAAAAAGGAAAAGAAAGAAATGGCAGAAGTATATGTCCACAATAATTCTAAGTTTTAAAAAACTTGTTCTTCACTGCAGATATTTT

>ORI-263

ATCTGCTTAGGGAGGGTCGCAATGGTGGAGATATGGTCCCCAGCTTGTATTAAGGAAACAAAATGTCTTCTTTATTGAAGAAAGGGACAGTATAGTTTTTAGTAAGAGTCCAAACAGAGCCTAGGCATGTGTACCACTCACCCCAAATAATCTAGGCTGTCCACCATTTTTTTTTTGAGACCGAATCTCGCTCTTTGGGAGACCGAGGCAGGCAGATCACCTGAGGTCAGGAGTTCGAGACCAGCCTGGCCAACATAGTGAAACCTCATCTCTACTAAAAATACAAAAATCAGCCGGGCG

>ORI-264

AAACCGTGGGAAAAACCATCCTTAAGGCTGAAAGAAGAAACACTGGAGCTGGGGGCGGAGACTACGAGGTGCTCACTGCGCATGTGCGCGCGCTAGGTCAGCTTCTCGGTTCCAGCCGGGCGGGGGGGTCGGGTGGGGGTGGGGGTAGGGGTTGCAAGGGCCTCATTTTTTCTTACTGCCCCCATCCGGTTCGGGGAGGTGGCGGGACCTTGGCGGCGCCCGGAGCCGGCGTGGCGCTCATCGAGGGACGCCCGGCCCAAGTGGTAAGTTGAAAAATGGCGGAGGGGGGAAGAAGGAGGG

>ORI-265

AGGTTGGGCACTCTGTTGCCATTGAATTTCCCCTCTGCTTTCTTCGCCCCCATGGGGGGCAGCCATGGAACGGGGGGCAGAGTCCAGGCGGGAGGATCTTTCTCGCCCCCGCGGCGCCCCCCGCCCTCCTTTCTCCCTTCTCCCCCCAAGTCGCTACCCCAACACTTCCAAGGAACTTCCAGCCCCCCTTCCTCCCTACCTCCTACTCCTAGAGTGGCTTTCTACCGCAGGGCTCTTCTTTTCGACTACCCTCGCTGAAGATTTTCTCTTCCTTCCCTCGCCCAACTCTCCCTCGGGGCC

>ORI-266

TCATTACTTGGGATTCCAAACACTACTTGATAATTCATTATAAAGAGGCTGCAATCAGATACTTGTTTCTGGATCTGCAAATACACAGTTTCTTCAGCTCAAAACAATACATGAGTTCTATGACTTTTGTTCATAGAACTTATAAGAGATTTAACTTACAAGTAAGAGCTCTTTGTAATATTTCAGCTCATTACATCTGGCTTTGATATCAGTTCTTAATAGCTTATTATAGTATTCTAAAATATTACTTAATGGGCATAAGTGCTAGAATTTTAATTGAACAACCTAACTTTTAGTATA

>ORI-267

CAAATGTTACAACCATAGACACATGCTCCAGCAGCCCTGCCCCCACCCCCACCAAATAACTACACACAAAAAAGGGTCCAGAAGGCTGCTGGGCTGGGACCAGGGTTTTTCAAGCCACCTTTTCCTGTCTCAGTTCAGAGACAGGGTGACCAAGGGCCCAGGAGGGGAAAGAGAAATGGGGACAAGACTTGAACACAGGCCTTTGCACAAGCCAGCTGAGGGCTGGGGACGACCCCATTCTCCTGCTGCCACACCCCTGTGGTGGGGGATGGAAGCGGAGGAGTCGGAGTGGGGCGGGCA

>ORI-268

ACATTGCTAGGGGAGGTCCCAACCTGCTGCTGAGATCCGGCACTGCACAGCCTCGTTCAGGCAGAGAAACTGATAAGGAGATGGTTGTCTGCTCACCCCAAAAGGTTAGCATGGATTCCTCGCCCTAGCCGGCTTTAGACGGAGGGAACAAAGGCTTTCCCAAGGCCCTGCCAGTCCCCACCCCTGCCCATCTTACCACCGTAGTCTCCCTCAATCATCAAGTCCCATTGCACAGAAGGGGAAGCTGAGGTGCCAAGAGCTGGCGTGGCCTGTTCAAGGTCACACTACTGGGCAGGGGCC

>ORI-269

GAAGTTTGCAGTGGGTCAAGATCGTGCCACTGCACTCCAGCCTGGGCGACAAGGGGGAAACTCCATCTCAAAAAAAATAAAAGCTTCAGAAAGATTCAAGAAGTACTGTATTCATGAAATAAGAATAAAGGGTTGGCCGGGCACAGTGGCACACCTGTAATCCCAGCACTTTGGGAGGCCGAGGCAGGCCAATTGCTTGAGCCCAGGAGTTTGAGACCAGCCTGGGCAACATGGTGAAACTCCCTTGCTACAAAATATAGAAAAATTAGCCTGGCGTTGTAGTGCAGGCCTGTAGTCCCA

>ORI-270

ACCGACCATACAGCCCCCTGGGCTCTGACAGACTTGTCCACTCCCTCCAAGATGGCCAGATGGGATTTTCCTCGGCCTGCTTTCCCCCATGCCCAAATTCAGAAGCTGCCCACCTGGGGCCAGGAAAGGCCATGGTAGAAGTAGTATTTCATCTGGTAGTTCTCGGGCAGGCAGAGGAGGTTGCAGTGCTGCATGTTCATTAGGTCCTCTGGCTGCAGGGAAGGAGGGCAGTGGAACGCGCTCAGTCTAAGGCGGCGGTGACCACGGGTGAGAAGCCTGCTCCCCGTCGGTCTGCTCGGC

>ORI-271

CAAGGGAACGGGGAGAAGATCAGAGAAAAGCACTGAAGCCCGGCACAGCAAAGGGGAGGGGCAAGCCGATCAGTGGGGAGGGAGGGGGAGGAGGGGAGAAGGGGGAGGGGGACGGGGAGAGGGGAGAAGGACGGGTAAGGGAGGAGGGGAGAGAGAGAGGGAAGGGTAGAGGGTGAGGGAGGAGATGGAGGGAGGGAGGAGAGGGAGGGAGCAGATGGAGGGAGGGAGGAAAGAGGAGGGAGAGGGAGGAGATGGAGGGAGGGAAAGAGCCGAAGACCCAGCGGGCTTTGCACTCACCCG

>ORI-272

CTCTAGCGCCACCCGTTGGAGGACGTCCCTTGAGAATGGACCATGCCACCTCACCCTGGGCCATCCTCTCCTGTGGTTCAGGCCAGTCTACACCCAGTAGTTCAAGGGACTCCCCCCTCCTAGCCATGCCAGAGCTGGAGCAGACAGGTGCCACTTTCCTGTCCTGTGAAGCGTCCTTGCCAGTTTTGGATTGGGGCTGGTGTGGGGCCAGACTTGAGCCACCACGGGTGGGATCGCACAGGCCATAGCCACTGGTCATATCCATCTTCTCTAAAGAATCCAACTGTCTCTCCTGCCTCA

>ORI-273

ACGGCTCCTGATGGGAACTATAACCACCTCTTTATGATGAGTTGTGGGACCCAGAACACAATGTCTAGTCCCTTTTGTGGATAATAATCCCCACCTACTTGTTCCTGCTAGATGGGGAAAGGAGCCGGTGGACCTACTCGCTCGCGGGGGGGTAGCGGGGGGCAGAAATTCATGGAATGGGCGAGAAGGAGGGGGAGGCAAAGCCCAGAGATGGGGGTGAGAGCTCAGGAGCCCTTGTGGCGCACTCTCCCGACGGGAGGCGAAGTGCGCACGCGCGCCGGTGGGGGGGCCGAGTAACGG

>ORI-274

GTTGAGGGCGCCGTCTGAGCAGGAGGGACGGACGGGTGCCCAGGGTTTGAGGAAAGAGGGGTGTGGGAAGGACGCATGCTAGAACTTCAGAGCAGTTCAGCAGGTGCAGAATGGGAGTTATCATGGGGACTGTGGGAGAAGGGGCGGTGGGGGCAATGCCCAGGGATGTTCTGATTCATGTTCTCTGTGCTGCAGGTGAGCGTGGCTCTGGGCTGTGCCTCCCGTGGACGGACCATTGCTTTTGCTAGCACCTTTGCTGCCTTTCTGACTCGAGCATTTGATCACATCCGGATAGGAGGC

>ORI-275

GGAGATAACACATTTTATTCTTCCATTCAGTTGATGAATGTGGATTAGTTCCACTTTTGTCTCTTGTGAATAGCGTTCTGACAATTCACATATTTTGTGAAGATGTAGGTTTCACAAAATATGTGAAAGATTTTGTGTAGATGTAGATTTTCCCTTCTTATGGACATGTATACTAGGAGTGGAATTGAATTGCTGTGTCGTAGGATAATTCTGTGTACCCTTTCAAGAGTGTGGTGGGGGGTGGTGTTTAAATACCGTTTTGTGAGGAGTAGATGCAGTGACACTGAAGGTGGCTGTAAA

>ORI-276

TTGGTTATATCCTGGCTGGCAGTGCGCATCCAACTCAGAGCCAACCTGGCATAGGGCCCAGGGCCCAACCAAGGAACCTGGCCTGGTCTCATCCTCACCATGGCCCCCATGGAGTAGAGCAGCCCCAGAACCAGCTCCCCTGTGCCCAAAGCAGCTGGTGCCCTGTGGACTCGAGCCAGCAAGGGACACGCTGTCCCCCTCCCAGTGGGCACACTGGCTGAGAAGTGTCCTTCCAGGTGGCCACTCGCCCTGAGTCCACACAAGTTCCTGGTGTCAGGGCCCTTCTCTCTCAGGCATGCA

>ORI-277

GAAGAGAGCTACTTCACCACCAGGACTTATGGGGAGCCCGAGTCTGCCGGCCCGTCCAGGGCTGTCCGCCAGTCAGTGACTTCATTCCCAGATGCTGACGCTTTCCATCACCAGGTGAGCTGGCTGGCAGGCGTCCTGTACTTGGGTACAACCTAGGGGATCGCGGCTGTGTTTGGATAAATCCAGGGGGGCACTGGGTACAAATGGTGGCTCTTGGGCCTCCGGGGAGACTCTGTGTGACTAGAGCACCCTGGTCTGGGATCTAGGCTCAGACTCTTCCTGAGAGTCCTGGGGGCAAAA

>ORI-278

CCAGCCAGGGGCCATGGGGGACATTCTGTGCTCCTTCTCCCAGCATAAGAACTGTACTCTGACCTCATATCAGGGTTACTTGGGTCTGAGTCTCTTCTGGGTCAACCATCCCCCCCAAAAACAACAACAACAACAAAAGCCACCTCATACTCTTAGGGAGCAGAGACGCTGGCTTTGGGGTAGAAAGGCCTGGGGCCTGAATGTCAGGGGCTGGGGTGTGGGCCATTTTCTTGGTGAGCCTTTATGGGTATGGCTTGCCAGAGGAGAGCTGCCAGAGAGGTGTGGGGGGCTGGGCCAGGC

>ORI-279

AGGCAGCGCGAGGGGCTGGGGAATTCAGCCTCCGGGGTCCACAGGAGCCGGCGGCGGGGGCTGGGGCGGGAGTCCACGCAGGACCAGGGGCGGTCTCCGGCGGGGCGGGGCGGGCGGTGGTCACGCTCCGGGCCAGCTGGCGCGCGGCGGGGCGGGGCATCCGTGCGTCTCCTGGTGGCTGACGTCACGGCGCGGGCGTCAGCTGACTGTTCGGCCGCCACCGCCGCTGCCGCTGCCGCTGTCGCTGTCGCCGCCGCCGCCGCCCGCCGCCGCCGCCGCCGCCGCCGCCGCTGCCATGGC

>ORI-280

CCTTGAGCCCAGGAAGTCGAGGCTGCAGTGAGCCATGATCCAATGCCGCTACACTGCAGCCTGGGTGATAGAGCGCGACCTCAACTTGGGGGGAAAAATCTGTCTATCTATCTATCTCTCCATATATGTGTTTAATAAAAATGGGCAAATGACTTGAATAGACATTTCTTCAGAGATCATATACAAAAGGCCAGCAAGCACATGAAAAGATGCTGAACACCATTAGTCATCAGGGAAATGAAAAAGAAAACCTTAACGGGGCGCGGTGGCTCACACCTGTAGTCCCAGCACTTTGGGAGG

>ORI-281

TGCTGCCGGGGGCCCGTTCCCCTCCTGCCTCATGCTGGGTCCCCGGTGGGTCTGAGTGGCCTGAAGGCCTGTAGGGGAGCAGGGGGAGGAGGCATCCAAGCCATGGCTTCCTCATTGGTTTTGAAAATGAGAAGAGGACCTTTGCTTTACTACCCCCGCATTCAAAACCAGCCAGAGGACAAGAGCCTCTGGCTGGGTTGGGAAACCACCCAGCGCGGGCCATGCTGCATTCGCAGAGCAAGGCTGCCACCCTGCGGCCTGGCCGGGCCTTTGGGGAAGCAGAGCGGAAAGGCGGTGTTT

>ORI-282

GGAAGGCATCGCCCTGGAAAAGCTCTTCCCGCAGGATCCCGCACACCTGGGTCCGGCTCAGGGCCACCTGCTCTGCCATGACGCTGTCTGGTGGAAGAAAGGCTCGTTAACAAGGCAGAAGAACAGGAGAGCATTGAGAAGTTAGCCCCTTTCTTGAGAGTTCCTCTGGGGTCTCACACCAGGGTGACACCTGATTGCCCAAATCACTCCTTGTGAACGGCTGGGCATTGGGGAGTGGTTGATGGGTTAGAAACTGCTGGCTCTGGGCTCCAGCCACTCTCTCCTAGGCAGCCTTCATTC

>ORI-283

ATTTGTGTCACTCCTAAAGCAGTGGATCAGATGTCAATGTTTATTATCATTCACTAATTACATTTATGAATAATTTTTAAAAGTAAAGGAAAAGACTGGCTGTTTTAACTAACATAATAGAAACTGTTGCTGGATTTTCTGTTGAAGGTATATTTTGAAGTGAGTTTTATTAAGTATAACTCGCATGTAAAATTCAAAAGAAAAATTAAATACATAAATAAAATGTGTCTTCCTCCACTAAAAGAGTAGGACACATCGCAGAAGCCGATAAATATTTGTTCAATGTTTACATGAACAAAC

**II. List of 282 human non-ORI sequences in the negative subset**

>non-ORI-1

GAAAGATGGGTGGGGGGGTGACTGTAAGTGTTGTGGGGGAGTATTGCATAATAGAAAAATGGACTGGAAGAATGCTTACGCTGTATCAGGAGATTGGAATTTGGAGAATGGAGCCAGGTTAATGTAGAGAGCTCAGCGTATTAATAGTTAAAAACGAATGAAGATTGAGGGAGGGCTGAGGAGGCTCACAAGAAAAGCAGTAGATTGTGTGTAGGGGAGAAAGTGTGTGTATGTGTGTCGGGTGGGGGGGCGGGAGGTGAGTCAGAAACTGAGCGCTTCCATCAGAAGTGTCTGGTGTTT

>non-ORI-2

AAATAAATAAATAAAGGAAAACATTTTTTGTAATGAAAAAAGAATAATTAGCTTCAGAAGCTATATCTAAATTTTGGTGAGAAAAATGAGACTTCCAAAGAAAATATTCCCCAATTTACTTGCCAAGAGTTTCCAAGGAAGTGTTGGGAGGCTCCCCCACTTTCCACTCAAGGCTTAGACCCAGTCAAGAGGGAGACTTCATGACACAGGCCAGGTGGTGAGGCCCAGTGGCACCAACAACTGTCATTGGAACAAAGAACAAAATGGCACTCACTCTTTCTCAAGAGACACTCGGCCAAG

>non-ORI-3

GCTTGGGTTTTCAGGAGCCGCCTTTTCTTATTGATCATTTCTCTTTTGTCATCATAATCTCTTGGCCTCAGACTGAGTTAAGGGAGCCATAGTAGGAGTGCAGAAAATGAAGTGGAGGAAAAAAAAATGTGATATTTAAAATATAGTTTTTCCTACTTTCTTTTTCTCTCTCAAATTAATTTAGATGTAAATAACATTTTAACAGGGCCCAACATCAGGCTCCTAGGCTCTGGCTGCTGGTCTCACTTGACACCAGAATAAGATTTTGGTGTGCTAGTAATTTTGCCAAATGGAGGCCTA

>non-ORI-4

GGTGCCAGGCGAGGGGCACCTGGGGCGAAGGCTGTTCCCAGCGCATGAAAGCGTTTCAGCGCCAGATGTCTCTGGAGGTCAGACGCCAGCCCTAGGGGCAGAATTTCTCTTGGAGACAGCGGTGGCGCGGGGAAGGAGTTAGTTCCAGCTGAGTCGGGAACTGCTGCTGCCTGAGGTGCAGACTCGCCAGGGGAGGGCATCTGCAGGTGCTGCTCGCCTGAGGGTCTTTCCTGCGTCCTGCGGTCTCAGCTGGCCGTTGTCAGCACCCTGGTGGCTTTGAGATGACTCTGAGGTGTGGGA

>non-ORI-5

GGGGAGGGCAGGCCATACAGCCTGTTGCTCCTGTGGTGGCCTGGTCTGGGGAGGAAGCTGGTGCTGATGTCAGATCTTCCAGCTCTGACTCACCGTGAGCTTGCTCAGGGTGGAGCCCCACCCAAGAGATACCATTTGGGAGGCCGCCATCTCCAGGGAGTTCATTTTCCATGGGGTAATAACCTAAAGAAAAGGCTTTCACAGTTTTAGAGCCAAGGGCAAGCAAGAGCTGGTGGAGCATCTGCTGTTGCTGTGTGTCATTCCACAGATTAGTGCAGTGGATATGTTATGCCTTCGTTT

>non-ORI-6

CAGGCTTTAAGTTGCTTGGGCAGGATGCAGCAAATTGTCAGTGTAAGAATAGCCATGCCTGGATACACAAAATGCTTAAACTTTAAGTGTGGTTTTGCATCCAAATTCCACTTACGTTTACTTTCCAAGGTTTGTAGGCCAATGTACCTAGATCTAATATGCCATTTAATTAAATTTATCTCAAACCTACACCCACCTCTAATATGATCATCAATTGCAAAGATAGATCTTTTATCCATTGGTAAAAGACACATATGTTTGTGTTCTGCAGCCATATCCAAAGAGGGAAAAGCAGAGACT

>non-ORI-7

AGGTGTAGGCTAAGTATTAGCATAATGTGTGCATCAGAGTGGCCACTAGGTGGCAGATGTGACAGTCAATGATTATTCTTTCGGAGAAATCAGTTCAGATGGTTAGCTACAAGATAATGGACCACAGTTATAGTTTTTCTTTCTTATTTTCTTCTTTGTTTACTTTCTTTTTTTATATTGAGATTGGATCTCACTCTGTCAGTCACATTGGAGTGCAGTGGCTCAATCATGGCTCACTGCAGCCTCGATCTCCCAGGTTCCTGAGTAGCTGGGACCATAGGTGTGCACCACCATGCTTGG

>non-ORI-8

AGCGCACATAGCAATTGGAAACTGAAAGCTTATCAGACCCTTTCTGGAAATCAGCCCACTGTTTATAAACTTGAGGCCCCACCCTCGACAGTACCGGGGAGGAAGAGGGCCTGCACTAGTCCAGAGGGAAACTGAGGCTCAGGGCTAGCTCGCCCATAGACATACATGGCAGGCAGGCTTTGGCCAGGATCCCTCCGCCTGCCAGGCGTCTCCCTGCCCTCCCTTCCTGCCTAGAGACCCCCACCCTCAAGCCTGGCTGGTCTTTGCCTGAGACCCAAACCTCTTCGACTTCAAGAGAAT

>non-ORI-9

TGGTTAGAGCAGGGCTAGCGGTGGGCAGGGGAGAGGGCCAGGGCTGGGCCACGGCCAGGGGGAGGCTCCCTTGGCTTATCTTCTTGGCCTTACCCGGTGTTCCTGTGCCCTGGACTTGCCTTTCCCTTCCTGCCTTTCTTTACCAGGCCCCAGCCAGAGCCTGGAGTTGCTATGGCAACTTCGGAGGAACCCATTTCCTTAGTGATGTCTATGGTACAGAGACTGGCCTGGAGCTCAGAGCTGCCGGCAAGAGGCCTCCTCTGCTGTCCTCAATTTCTCTCCAGGCCTCACCCACTTCCC

>non-ORI-10

AGCCTGGGGGAGGGAGGAGGGGCTCCCTGGGGGCGTGGGAGGGTCCTGGAAGGCCTTAGGAGGGCGGAGCCCGGGCAGAGGCGTGGTTAGGAGGAGGAAAGGGGCTGCAGAGGACTGACTAGCTGAGGGGTGCAGGGCTTTCTGTGGGAGATAAGGGAGGAGCTGACTCTGGGTCCTGGTGAGAGATGCGCTGCCCAGAGTAGGAGATGAGGCCCTGGCCCCAAGGTAGAGATGAGGCCAGGCCCAGGCTGAAGGTGAGACCCCACTCTGCAGGACGAGGTGCTGCTGACCGAGAGCAGC

>non-ORI-11

CTGAACAAATACTCACGGGCCTGAGTCTTCACAGCCCCAGGGGAAAGCCGAAGCCGGCTCCGCTGGGGCTTCTCTCGGCCGCGGAAGGACAAGGGGTTATCGCCACCAAACCTCTCTGCCAGCGTCCAGGAGGAGTTGGGTCACCAGTACGTGCGCAGTGAGTCAGGTAATAAGAGGCCTGCTGGGTGAGGACCCTCCTCCCCTCCTGCCCTCCCCTACCCCCATCAGGGAGCAGTCATGGCTGGTGAGAGGTGGGCCACCTTGACAAACCTAGTGGAAGGGGTCTGCTCAGACAACTAT

>non-ORI-12

GGGTTTGGTTGGGGTGTGAGATCTGAGCTTGATTTCTCTGCTGGGATTTGAGATGTGTGGGATTTGTGAGGTGTTGGGTCCAGGCAGAGACTATATTGAGCTTTGGGTTTACATCTTGGGAGAGGGCTAAGTGTTGCTATTTGGGTTTTGGGGCTTGGTTGGGGTAGGAGATCTGAGCTTGATTTCTCTGTTGGAATTTGAAGTTTAGGTAGCAGTCACCATTATGCTCAAAGCGGTGATCCTGATTGGAGGCCCTCAAAAGGGTGAGGTGCCAGGGGAATGGGGGGAGGAGGTTGGGCT

>non-ORI-13

CAGCCATTATCGGGGGAGCAGATTGCAGCCCCCTCCCCCTCTCTGGGAAGCAGGCACAGCTGGGGCCCCACCACATCTGCTGCATCCGCACGGAGCACAGCTGGGTGTGCCCAGAGCTGCACTCCCCAAAACACAAAGGGGGTGGAAAGGGGTGGGTGGGGGCCAAGGAGAGCCCAGACCCCTGAGACAGACACACTGACACCTCTGCCAACAGACACATACTCTTTGAGGCGAAGCTACACCCACTGACACACGCTCCCTCGTGTGCAGAGACGGGAGAACTGGCAAACATGCAATTAC

>non-ORI-14

GGGTGTGCATGCAGCTAATTGCTGCTCCACTTCCTGGCAGCCTGGAGCTCCCCTCACAGCACCCCCCGCATGCCCCTTATACCCAGTTACCACCCGATTTCTTTCCTTATAGAGGTTGAAGGCATCCTGCCTACCCTCAGGAGGAAAGAGGATGTGATTTGTCTCATGATGGAGGCCAGTATGTTGTCGGGGAAGTGTGACCCCAAGATCTCAATAGATGGTGGGTGAATTTAGAGATATGTATGCACTCCAGGGTAGCAACGAGTGCTGCGGAGAGTTTGGGGGGCAGTTTCCCGCTTT

>non-ORI-15

CCAGTTTATTAAAGGCAGGGAGCTTCGGCAGGGTCCAAAAGGGAGAAGTTGGGAGATGCCCCCTCCTCAGCTCCCTCCTTCCCCAACAACTCTTCTACAGCCCAGCCCCCAGGGCCCAGAGGCAGGGCTGGCGTCAGGCAGACTGTACTGCATAAATACGTGGAGGCCACAGCCCGAATCAGCAGCGTCAGGGGGCAGGGAAACTGGGTTTGGGATGAGAAGTGGGTGGCTCTGGGGGCACGTCCCCCAGTGCTTGTCCAGAGCCCAGGGACCCACAGAGCCAGAGAGGGGACCAGTGGG

>non-ORI-16

CATCAGTTTCCTGATAGGTATTACAGTGACAAGACCTACCTCCTAGAGTTGTCCTTGGAGTAAGGGATTAAATATAATGCAAATCTTAGCACAGTGCCAGGCACATCCGCTCAAGGAATATTAGCTGTTTCTGTCTGGGGCATTATTATCGTTGGTGTTACACTGAAGTGGGCTTGGCTGGGGCTGGGATCTGACCTTCTCCCAGGTAACATCAGCCAAAGCTTGGGAAAGCTCGCACTCGAACGTAGCTGTGTCGCCCTCGGTCACCTCTAGGTCGTGTGGCCCCCGCACGATGGTCAC

>non-ORI-17

CAGCTGCCTGAGGAGTGGGGAAGGTGATGAGGGCAGTGGGGTGAGGACAGGGTGCAGTGATGGGACCGGGTGGCTGCCCAGCCCCGGGACCTCAGCTTGGTGTCAGACTTGATTCGCTCTGGCCTAGAGCAGAGGTGGTGTTTGTGTGAACTTCCCTGTGAAGAGACTGGTGTTTGTGGGGAGCCTGAGCATGGGCTGGAGTTTGTGTGGCTGTGGCCATTCTGTCTGGCCATGGCTGGGTGGGAGAGGGTGAAGATGATTACGACAGGGCAGAATATGTGCAGAAGGCCTCTTAGGCAT

>non-ORI-18

CGCTTGAGGCGAAATCCTTCCCAGCCCCTCCCCCACATCCCTGGCGGGAGTGGGAGATAAGGCTCATGGCCACAGACATCTGCGTCAGAGATAGGAGGTCTCAATGCCACGGGCAGGGGCAACTCGGACTGTGGGGCGTGGAAAGGACTGGGGAAGACTGGATGAGAAGGGTAGAAGAGGGTGGGTGTGGGATGGGGAGGGGAGAGTGGAAAGGCCCTGGGCAGACCCTGGCAGAAGGGGCACGGGGCAGGGTGTGAGTTCCCCACTAGCAGGGCCAGGTGAGCTATGGTGCTGCACCTA

>non-ORI-19

GGTTATCATAAGAGAGACCATAGCGGTCCTTGCGCTCTAGGGCCACCAGGGAGCTCATGCAGCTTTTCTAGGCTTGGGAGAGAGAAGGTGGGTTGGGGGAGCTTAGGAAGGACACACTCTCAGGAAGTGGTGTCTCTTCCAGGGACATCCAGCCTAGACTATAAGGTCCGGAGCAGCTGGTCAGGATATCAGGCTCTTGAGATAGGTGGATTTGTTCTACGAATGTGGCCCACACAGCAAACAATATTTTAAACTTAATTTTTAAAATTATTTTTACTTCAATAGGTTTTGGGGAACAGG

>non-ORI-20

AATTCAATTAGGTGACGTTTAAACATCTATAAGAGGTGTGTGGTCCTATGAGATAAGAAGCACATGAAAAGGATGCAGGATTCTGTAGAGATAGATGGACAAATATGTAGTTATAAGCCTTAATGGTCACATAAATACACAATCAGGTATAGAACCTGGATAGGGAGTTATGGACTACACTCTGGCTTGACCTTTAGTAACTGATCACTTGTAATCAAGGGACTAGTCTACTCAGCCACCTCCATCTTTAAGTTAGAAAGTTAAAAACTGGAAATAGGGCTCTACAGGTCCATAGAGTCA

>non-ORI-21

GCTCCTGGTGCTGTCACTCTCAGTGGCTGCGGGCAGGCCGGTGTTCCAGGAATGTTCTGCGTACCCGGCGGGTCTTCTCCAAGAGCCACCTAAGGGGAGAGTTGAGGGCATTCCCCAAACTGGCGCCCACACTGCCGCTGACCCACAAGCCAGAGGCAAATTCCCGCACCTCGCCCTTCTCACAGCTTGTGCAGTCCGCCTAAAGCAGAAGCTCCCCAGGCGTCCCAGCTTTAAGACAGTGTAAAGAAAAACAGGTGCCCACTGGTCTAGTCACATCCCCTATAAAACGGTTGGAGTCTC

>non-ORI-22

TCTAAGCTTTAACCTAATCAGCACTGTGGCTTACCCTCCCCACACCCAACCCTCTGAGTCCGAATTGTTAACCCGAATCCTCCTGCTAACCTCCAAAGCTCAATCAGAGGCTGGATATAAGGCCAAACTACACCACATAGGCATAACTCTGAAGCCCCGTGGACTCAATGCTCAACCATAGTCTACACATCTGATTCCAATTCCTAAGCCCAAACTCTAAACCTAGCCCTGAGATTTAAAGAGAACCCCTGTCCCTAACCAAGGCACTAGCTCACACTCTGCCTTTGTGTGAATTTGAAG

>non-ORI-23

AGGTTAGATCCCTTCTCAGTGGCTGAGGCTCTTCTGCCTGAGCCCAGATGGAAGCCCATCAAAGAGAGGGGCTCCCTCTGTCCAATGCAGCTCAATCCAGTGAGCAGTTATTGTCACCTGCTGCCATCAGGAGCTCTGCTGGGTTTGTCTTGCCTTCAGAAAATTCTGCTATTCAGTAACAAATTGTGTTGTGATGGCATTTTCTATGCCTTTTGTAAGAATGGACTCACGCACAGGCTATTTTTGGATTCAGGGGTTGGCTGGGGGTGGAAGCAGGACAGTTTCGGTCATGTGCAGCTC

>non-ORI-24

TTGCTCAGGGAGGACACAGGGCAGGGACATGGGGTGGGAGTAGACAGGGGCAGGTGCGCAGGAGAACTGAGGAAGGACAACGTGCTGTGAGTAATTCCCAGGGGCTGCGGGAGGAAGGGTGGGGGTCTGGCTGACCCCTACCTATTCTGTCTGCTTTCATTGTGGGTGATGCTGGCTATGGGGGGATGCTTACTAAGTATCTCTTCTTGTTATTTTTAAGAACGATCTTTGAGTGGGCCACTTTTAACTCAGCAATCCTGGGTATGAAAGCAACAGTATGGGAAGCACCCTGTGCTTAGG

>non-ORI-25

TCAATGAGCACATTAGCAGGTGGTTAGATAATCAAATGATCTGGTGCAAATGAGGTAAGTTTCATTAGGGGAAAGCTGGCAGGGTGAAGTCCCAAGTGGTTATGGGGGTGGGCTGGGGGTTGGCATCGTTATAGAAGCCTTGTGAGCCAGGGAAGTTAGCAATAGCACGGTTAGGTTCCTGCTGTAGTGTGTGAGGATGGCGGGCTAAAGAGACAGGAACCTCCGAGAGCTCAGTTTGGTGAGATGTCTTTGCTTTTCATGTCTTTTCATGTCCTTGACTCTACTTGAGGCCATGAAAGC

>non-ORI-26

GTCCAGTAGCACAGAAGATTGGTTAATAGGATGGTGTGTCCCAGCTTTTTACTGCACTTGTACTTGCTTCGAATATTTAGGACCCCTATATAAAATTATCTTCTCCAAAGTCATAATTTCCAAGAAGATCTTATACCCACACTATTTTCATAAGTAGTCAACTGCAAGATCTTTAGAACCATGAGGGGATCTCAATACACAACAATTTAAGATTAACCATTTTGTTGAATGTAAGATTATATCCCCTCCAAAGAATGAAATTAAAATAGAGAAAAATAAACAGCACACGTGGAATCCTGA

>non-ORI-27

CCTGAAGTTGAGGCTGACTAAAGTGTCGTCAGGATTTAAAGGTGTGACATGTTAGGAACTGAAAGTTTGTGTTAGAACACTGCAGGTGAGAAGCTTCTATCAGTAAAGAAGACGCTATTTTAGCAAGCTTTGAACATCACACTTGAGTGATGGAGACTTGGCACTCAAACAGCTAATAAGAAGTCATGTAGATTGTTTAAGAAATGGAGGGCATGTGGAGATTGGCACTTGAGGGCTAATCATGGGTAATTTGCCCACCTCTTTTGAGTCTCAGTTTCCTCATCCACGTGGGCAGTGGTG

>non-ORI-28

TCTTAGGTCTTGTTTAATGTACGGTTTTGGGGGGTGGGGTAGGAGATGGACCTTCAGCCCTGCATCCAAAGTTGATTAAAATCCTGAAATTTTTCATAATTGGACCTCATTAATAAATTCCTCAGGGTTCCAGGAGGCTCTGTAGAGGTAAAACAAGGGTTTGGAGAGATGAGAGCAACAGGAGGTTGGAAGATCCCAGACCACAGAGTTAATTCCACTTAATCAGCCTGGAGAAACTCCACATTTACAACACATCCGAGGACTCAATGAGGCCTCCCACCCTCTGCAGCACGGCAGGGA

>non-ORI-29

CATACCTCATGCCAAGATGAACTGAGATCGAAAGTGACAAGACCTACCTGAACCACCTCTAACCAGATACTCTCAGCAGGGGCTCAACTCTCGTTCCAGTCTTGCTTGTTACATTTTGCTGTTGCCATTAAAGGGTGAAAGGATGATCCAGGGAGCAAGCAGATTCCCTTGTTACTATTTAGCTTATAACCAAATCTCTAACTCTTTAATTATTTTCTTGATGCATTTATTACAAGAGAGCTTTAGTTCAAAGGTAGCAGAGCTTGAAAGTTAGATTTGTACAAAAATTTAAAAAGCAGC

>non-ORI-30

GTGCTGTAGATCCGTTGTGTCTTCAAGCCAGAGATGGAGATGTCTGTGTTCAGTTGGTCACCTCATCCCGCACCTCCCTCTTCCTTCCCCTCACAGCCCTATCTCAAATCACTTCCCAGCAATGACTTGCCCTTCTCCCTCCAGGGCTGTCCCTGCATTCCCCACACCCTCCCGGCTGGCACCTCTGCCCAGGCTGTGGTTTGGCCCCGGAGGTCTGGACTGGACAGGGACAGCCTGAGCTGAACCTCAAGGAACAGGAAAGTTCAGTGTTTGCCTGAGGCACAACGAGTCATTCAGCAG

>non-ORI-31

ATTCCAATTGATTTCTTTCTGGTAGGAAAAGTTCTCCACATGTATACTTCTCATAGTTCCTTTCAGCCTCTAAAACATTATCTTAATTTTAGATAATACCTGCAAAAATTGGATTCAACTTGTGTATTGAGAGCTAAAGAATTTTCATGTGACTTTATACATATAGTGTGTGGCTTCTTGTAGCCAGACCCTTGGGAGGCCCTAAACATTGAAAAGATGATGGAGTCCTACCATCCCACCAGTGGTAATTCAACATTTTATTATGATGCAAAATTTCCATGCATGCTGAAATCAATAGAG

>non-ORI-32

TAGCAGGTCCCAGCGGCCGTGACAGGCAGAGCCCCTCGGCCCGCCCGGGACTCGAGTCCCTAGGCAGATACCGCGGGCTGGGCCCGGACGCAGGCGGCGCGCCCCCAGCGCCGGCCGCAGGGCTCCAGGAGGACGGCTCGGTAACGCCTGTCAAAGCCGTGCGGCGGGAGGCTGCACACTCGCGGAGGCTTGCAGTCTTTTAGGAAGTGCGGTTTGTCTAATTGCAGCCCCCAAGGGGCTTTGAGTGTTCCTGGGAATGCGAACTGCACTGCTAAGTCACTTGAGTGACTTCTGTTTGGA

>non-ORI-33

GTGAGAGTGCGTGGCTTTTTCTCCACCAGGGGTTAATTAGGTGCTTGAACTTCCTCAGAAAAGCCAAGCCAGGGTTTATTTTCATAGTTCATACAAGTACTTCACGTGGTTAAAGAAGCCCATCCCGGCTGTGAGAATAGCGTGCAGTAGCTCGCGCGTTAAGAACGGGCAGAACGCCACTCAAAAGCACGTCGGGATGGTGGGTTTCGGATTGGGGTGAGTCCCGCCGGCGTTATTAAGGGCGCACCAGTGATCAGCTCAACACCGTGCTCACCAAAGAGGCCGACCGGGCCTCCCTGA

>non-ORI-34

ATGCCATGAAAAACTGGGGGATATACTGAGAAAAGTACCTACAGAATAAAGAACACCTTCCCCAAATCTGCCTGCCCAGAGCCAGAGTCCAGGTCCACAGCTTTGGCACCCTAGCCCCCTCACACATCTCACCAGTTTGACACCCTCCCCACGACAGAGGCTCTCGTAAACATCCCTCTCAGGCAGGTAGTCCACAGGCCTCTCATAGATGCCTTCTGGGGTTGCTAGCTCAGCTTCTGTCTGATTTGTTAACGTTTTTTCTCTCTCTTCCTCCAATAACTGCTCAAAGTACCGCAGATT

>non-ORI-35

GGACCCAGATCCCCTGTGTATCCGAGGCTTGAAGGCGGAGGGTTGGCCTGGGCGCCCCGCATGCTGTAGAGGCGGCTTCCAGGCAGAGGCAGGCAGCCCCAGCCGCCCTCCTCCCACGCTGGGCCTGTCTGCCCGATCCCGCGAAGAATCTCCCAAGAGTTCACGCACCCGCCTCCCCGCTGGCCTCATCTGGGGGAGCCACTGTGGACCCGGGAAGGACCCGCGGAGATGGGCCAGGGGTCTAGACAGCTCTACAGAGGCCCAGAAACTCTTCAGACTCACCCCCTGCCTGAAGATCTG

>non-ORI-36

GTTTGCCTTGGGCCTTTCATGCCCCGGCTGCACACATCGTCTGTGAATTGTGGCTGTTCACTCCCGAGGATGTGTCCTAGACTCCGGGTCGCGTGGATCTACCCTCTAGTTTACTTGCTCGGGAGAAGAAACTGACTCGTTTTATTTAGTGCCTATTTAGCGAGCCCAGAGTAACGTACATTTGTGCTGTTTTCAATTTTGTGCTATCGCAAATCACAAAAAAACTGTTATCAATTCACATTCATCATCAGCACGAGACCCATTTCCTTGTGCTCCTGCCAGCTCAGGATATTATGTTTC

>non-ORI-37

TGACACAATGATTACCTGAGGAAAGCATTTAAGTTCCAAAAGGTCTATTCCCAGGGAAACTGGAAACAAAATTTGTGAGTGTGTTTAGGACCACTTTAGTCTACAGAAGGGCTAATATAGTGTTTTTCAAACCTTTCTAAACATTTTGGCCACAGAACTTTCATTAAAGTAGGATAATTTAAGTCTAGTAAATGAAATACACCCTAAGTGGCTAAAAGTATGGCTGTTCTGCCTGCAGCCCCTGCCTTCAATTCCCAATGCCCTGCCTCAAGCCTGTCTGTGCCCCCTTGGAAGGCCCAG

>non-ORI-38

GAAATATAATTTTAGGTCAGGTGCAGTGGCTCATGCCTATAATCCCAACACTTTGGGAGGCTGAGGTGGGCAGATTACTTGAGGTCAGGAGTTAAAGACCAGCCTGGGCAACATGGTGAAACCCTGTCTCCACTAAAAATACAAAAATTAGCTGGGCATGGTGGTGGGCACCTCTAATTCCAGCTACTCAGGAGGCTGAGGCACAAGAATTGCATGAACCTGGGAGGCAGAGGTTGCAGTGAGCTGAGATCGTGCCATTGCACTCCAGCCTGGGGGCGACAAGAGCAATACTCCATCTCA

>non-ORI-39

GGAGTTTCCGGGAACCGAACCACGCTGGGAGCGCTGAGGTCTGCGCAGCGGCGGGGGCCGGGGGACGGGCGGGCGTCCAGTGTTACCGGCCAGTGGCCAGCTGGAAGTTCCAGCGGGAGCCGGGGAAAACCGGCCCCGGAAAAGCCCCACCTGAATGCACCTGCCCAGGCCTCTCCGGATGGTGTTCATGCTGAGGGTGGGGGTGTGAAGGATGGACCTGCCTGCAGGGTGGCCTTTAGGGAATGAGGGAGGAGTTCTACAAGCTAAGGGGTTTGAGGGTGTGCACGCGGGGAAAGAGGG

>non-ORI-40

AGTGGCTGCTTCCGCAAAGGACGAGAGAGTCTTGGAAGCCAGGGGAGGTGGGTGGGAGTCAGGGTCTCGGCATTCTTCTCCATCCAGAGCCTAGGGTTTAAGGCTGGAAGGATATTTACGGAGGGTAATGACTTTGTAAACAAGACCTAGAAATCTATCTCCCAGTAGCATCATCTTTGACCCATGTCCTCTCTGCCCAGCCAAAGTATTGCCCTGCCTATCCAGAAAGATGGAAACCCTTCCAGTGAAATTCCACGTGACTCCCTCAAGAACAAAGACTGGAATTGAGGCTGATGTGGG

>non-ORI-41

TCAAGTCTCACCCTGACCACAGCCAGTTTCACCCTGCCCACATAAAATACCAGCATGCCAAGGGCAATAAATAGCTATGAGAGTCATGGAGGCTCCTCAGGCCAACCGGAGAAGACACATACTGTAAATTTTAATTTCCTTTCTTCTTTGATTGAGGCTCATTTGCATTCAATGGCTAATGTGAGTTGTAGTCTGCCACAGGATATGGGTAGTGGGTTACAGTTTGTATATGAGGACAGGATGGTAAAGGAAAGTAGGAAGATTTAGAGCACGCCATGGAAAGGACACATGCAGACTCTA

>non-ORI-42

TCTCACTGTACGCTTTAAATTGGATCAATAAGCAGGGTGACTTAAGTATCTTAAATTGGTCTGTGAGTCTTTCTGTTCATGATCTCCAGACAGCTTACCTGACAGGGTACTTGGAAGCTACATTGCATTAAGATAATTTCCCACATGACAAAACAATGATTTGCCTCTAGGGCTGGGCACATTGCTATCTGAAAAAACTGGAGTCTGTTAGAAAAGAAAGAGAATGGCTGTTGGGTAGGTGCCTGAAGGCACAAGAGTTCACTTAGTCTGGCCTTCTGACTCCAGGTCAGGCAGGCTGAG

>non-ORI-43

TGACCTCGGCTCACTGCAGCCTCCGCCTCCAGGGTTCAAGCGATTCTCCTGCCTCAGCCTCCCAAGTAGCTGGGACTACAGGCGTGTGGCACCATGCCTGGCTAATTTTTGTATATTTAGTAGAGATGGGGTTTCACCATGTTGGCCAAGATGGTCTCAATCTCTTGACCTCGTGATCTGCCTGCCTCAGCCTCCCAAAGTGCTGAGATTACAGGCGTGAGCCACCGAACCCGGCCGAAAACAAAAGATCATTTAAAAAGTAAATACAGCCGCTGCACATGGCTATAAGAGTTCTACAAT

>non-ORI-44

TAGGGAGGGAATGACTTCAAGACCCAACGTGATGAGAATTCCAAAGGAGGGGGAGGGGGGGTCAAAGTCTCCAGGGCTTCCAAGAGCAGAAAAATCTGCTAGACCAGCCTTGGAGGCAGCAGTGGGCGCCCATGGAATCTGTTCCATTACATTCATTTTGCATGATTAGCAGCAAACCAGGCTAAAAGAATTAACAGAGCCTTCAAAACAGATGGTTCCTGGGTAACAGGATGTCCTCTTGTCTTGTTTACTCAAGAACAAACTGCGTCTTTGAAAACACTAAAGTCATAACACAAGATC

>non-ORI-45

AGCTATTTGCATCCTCCGTTTGGCCTCCCTTGGGAATCTTCAACTCCTCGAAGGCAGGGATTGGATCTTGTGCACTCCCTTCCCCCACAGACATAGACTCTCTCCATATACTTAGTAGATGCTCGGTGAATATCTGTTGGTGTTTATTCAGTTGAATACTAGGATTTCTGGATAACACTTTGGAGACATACATATTACAAAAGGAGAAGAGTTTTGATTGCAGGGAAGGGAAAGATAATTTGGGCGTATAGAGATCCCACACTTAGGTGGTTCTTCTAATGGAGAATGTTGAATGCTTTG

>non-ORI-46

CGTGAGTTCAGCCTGTGTGTTATATCCTTCCCTATATTCTTTGGTGACCATATTTCAGTTGTGTTTTTCCCTCTTCCAAGTATGAGTGACATTTGTGAGGTTGTATTTTCCTTCTTGTGTTAAAATATTAGGTTCACTGTCTTTATTGCCCGTGCCCTGGTGCCTGAGGCCTGTGAGTCAGCTGTGCTCAGTCTAGCAAGCATCCATGGAGGACCCGCCGTGCGCCATGCATCGTGCTGGTGCCAGCTGTCCCATTTCATGAAACTCCATTTTAATAAAAGATACTGACAGATAGAAACC

>non-ORI-47

TTGGCAAGGGACAGTGGCGAGTGCCACCTTCACAGAGTCATTGTTGAAAACAAGGGAGAGAGAAGAGGCTTGGGTAGAACCAGGGAACCATTCCCCTTTCAGGGTAAGAGGAGGAAAAGTGAGAGGCAAGTGCAGAACAGTCAGAGATAAAGGGGAAAGGCACTGAGAGGATGGTGCCTTGCAGTCCCGGGTTCATAAGACAGGTCTTCCTGCTGTAGAGGTGAAGGATAAGAGGGCTGTTGGTGGCCTGTGGGTGGGATTGGGTCTGGGGTCGGGCCTCGATTTCAGCCTGCCATCTTT

>non-ORI-48

ATGGCAAGTGGTAGTTGTCATCGTCTTATTTGAGCCTAGTTGGTCAGTAGAGGCATCAGAAAAGCCATGTGAGGAGTTTCACTTAGCCTCTTCTCTGAGGTCCTGGTGTGTGTACTCCACCCAGAAGCTGAGATGAAATTGTCAGGGAACAAACATGGACTTGTTCACAGAATCCTAGAATAAAAGCTAGATTAAGCAACCCTTCCATATCTACTCTGCCAGTTCTTTTTCTTGCCTTCCTCCTCCAAAGAAAGGTTCAGTAGCGGTAATGTAAGAACATAAAAGGAAGTAGTACTTTCA

>non-ORI-49

GGAAAGACCACTTTCTCTTGATTGCTCACTGCTGGTGCCTGAACCAGAATCAGAAGGGTGGTGGTGGCAGCAGTGGGGGTTGGTGGTGGGTGGAAGCAAGGGAAAGCTTGTGTGTTTGTGCATGTGCTGGTGCCTAAGAAAGGGAAATGATTCTTTTGAGTCTGATTCTTGGCTGAGTTAGTATTTGGGGGTGGGGGTGTGGCAAGCAAATCACCATGAATACCCTGCAGATTGCTATGGCCTGTGGTAAGGGAACAATGACTGGATTATACTTTTCAGCTTGCCTCTGCAATTTTATTA

>non-ORI-50

GGCTGGCCTGCCCTCTGAGTTTAGGTCCAGTGCTATATACTTTTGTGCTTACTATTCCTGCTGCAAACAAGGTGGGAGAGTAGGGGGAGGGGGGGCGGGATGAGATTCCTTTACCGTCACTTATTATAGCTGCCTCTCAGAAAAGGAGAGTTAGTTAGGTGAAAATAAGTGGGATGGGATTATGTTTAGTAAGTAAAGTAAATTTTTTTTTTACATAATAAGTAATGTTTAACAGGTTAAAAAAAAATCATTTAGGGACTGAAAATCTGAAGACCACAATGTAAAATAAAGCAAATGTGT

>non-ORI-51

CCAAAATACAGTAGTTCCTTCTAGAAGCTTGAAGAAATTTGGCAGATCCTACCCTCTGATGGTCTATTGTGGTCTAGAGAGTCAGTGTTATCCTGATTTGAGGTTGAATTTTTCTTAAGGTCAAACAACAAAGCTTCCTGAATTATAACTATGGCAACAGTGTTTATGTAACACACAAATCAAAGTCACGGGGTAGCGGAGGAGAAGCACCCACAACAATCAGTGTTAATAATCCCAGAACAGTTAGCCTGTGCCTAATTCACCAGAACCAAAAACAAGTTAAAATAAATGGCCTACTCC

>non-ORI-52

TGAGTGTGTGCGAGGGGATGGGGGAAGGCCCAGCATGTGGAAAAGTCATTTTCATGCTTGAGTTGGGAGCAGACAGAGATCAGGGGAGACGAGGGAGTCCTCGCTTTCGGAGGGGGTGTAAAGTAAACTAACCAGGGAGGAGCCTCCCACAGCCACTCTGGTCTGAGACAGCGCCTGCCCAGAATCCCTCTCCACAGCTCTTCTAACAACTGAGGATCCCTGGGGCCAGCGTGAGTGGGAGTGGAGGAAGGAGTCCAGGAAATCCCTGGAGATCCGCAGCCTGGGATGGCCCCTGTTGAG

>non-ORI-53

TCGAAGGCTCCTGGGTCCTGGCCAACTCTTCTTGGGCTTCCTAGAAGTTTCTTCTTTTAACAACCAGTGATTTAGGCATAACCAGCCTTGCTAGATACAAGACAGTTCAGGTCACTGATGGGGTTTTCCGAGTCCCTTTTGCAAACCGGCTTCCTCACCTTCCCTGCTGCCCCAGTCCAGAACCGAATAGGGCCTCACACAAGTTTTGTGGATTCCCAAAAGGGCTTCACTTCACTCTGTCACTGGGCACAATGGGTCAGTGAATGCTGTGGCCGAGCTGCCCTTTCTTCCTGGCTGGTT

>non-ORI-54

CACAGTGTCCACATTGGGAAAAGATTAATGACGAAAATTAATCGCCTGACCTTTCTTTCCAGGAGCTGGAGGTGAGGCGGGCCATTAAAGCCTCTTATGTTGTAATTAACGTTGGAGGGGAGAAGAGAGCTGCCTTCAGAATGGATGTGATCATTTAATTAGGCTTAGGCGTAGGTGCCACAACAGGTCCGGAGAAGTCATAATTTAACTACAGCTTTAAATTACACAGGCAGAGTGGGGATGGCAGAGTTTGACGGCAGGAGAGCCTGCCTCGGAGCTCGGGGAAACAATTAGATAAAC

>non-ORI-55

TTTCTTTCTTCATTAATGGCCTTTTTTCCCTATCCGAGAGCCTGGGCGCGGGAGGGAGCGAAGACGGCCGGGGGCAGGGCAGGCCCTCCGCAAGTGACACTTAATTCCCGGTCTGGACTGGGCCGGCGCTTGAAAGCAACTAAATTAGCAAAACAGAAGTCAACTGTCGATTGCTTCATTGGAGGATTAAGTGTGATAAATGGGGGGTTTGTTCGCTCTCCGGTCCAGATGTTGTGTCTAAGTAAGGGTTTTTTCAAAAAAAAGTTTTTTTACATTCTTGGCCCAGGGCCCATATATATT

>non-ORI-56

AACACACACGTACACACACATCTCATAATCACTGTAACTGTTGAGCATAGAAGCTGCTGAAATTCTAAGCTATCACCACCAGCACTGGGCCCAAGGCCAGGAGGGATGCAGACCTTTGGCAACCTCAGGGTCCACTCTGGTATGACCGTATTTAACAAATGCCTATGTAGCATTCCTTCTGTCCTGAGGCTTTAGCTAGCTTCACTAATTTCACCATTAAAGCAACCTCCAAAGTGGACACTGTATCGTTTCCTTGATGCTGGTTCTTTCCCTCCCCTCACAGCAGAGTATACACCCCAC

>non-ORI-57

TCAAAATCCATGATTCTACAAATCTGTTTCAGTCAGAACGTCAGCATCTGCTGAGAGCCCCCATGAGGAAGGGACAAAGGCTCCTTTGGTGCCATGAGAGGTGGCAGGCAGAGCAGGGGCAGGGGTGAGGTGTGCCACGTGGGCACTTCTCAGGAGCCAAGCAGGCTGTGCTGTCAAAAGTGGGGGCCACGGAGGAGGCCACTGTATGGGGAGCTGGGAGCTGCCGCAACATTGTCCAGCAGACACGGGCAGGTGAGAGGGCTCTGTCTGCTGGGCCTTCAGCTTGAAAACACAACAAAA

>non-ORI-58

TGAAAATGGGGGCAAGAGAGCGGGGGATCGCGGTCCTACCCGCCGTGTCGTCTCTGGTGAAGAGGGGACTTGTCTTGCTGAGGTCGCACCTCGGAAGTGCCAGGGCCAGTCAGCCCCTTTTCCATCGCTCGGTCAGCTCGGGGATAGAGCGGAAAACTTCTTGGGGACTTGAGAGCGTCTGGAAATGGCCCCTGTGATTTGTGCGCCGGAGGTGGAGGGTGGGGAAACGATCTGGCTGACACCTCTACTCACCCGCTGTGGAGAGAGCTCCCGCTTCACCACTTAAGGGGGGAAGAGAGG

>non-ORI-59

CTGGGTGGAGATTCCAGTGCTCTGACAAAGTCTGAGTTTTATGTAAAACAAGTTTATGTTTGGAAAAAGTTCAAGGTGCCCAACAGATGTGCAGCCTTAACAGTAACCCTCTTAAAATTCTTGACCCTAAGGAGACAACCCTCAAAGGGGAGGAGCTGGGCAAAGATCAGGGCGTGGCCTGGGGTGGAGGAGGGGAAGACACAATCTGGAAAGTCAATAAAGAGAGGAAGAGAAAGATCTCATCAGAATGAGATCTAAAAGGTTCTCATACCCATGCTGGTCAGGATTGGGCCTCTTTAA

>non-ORI-60

TTAGGACTGCAAAGTAACCCAACCCTTTCCATGCAAAGTAACCCAACTCTGATAATTTGCCAGGCTGGTAACTGGTGAATGTAGGGCTTGGATCTTCTGCTTCTCACTCAGGTCTTTCTGTCTCATTACACCCTGTGGGAGAGTCAGGTCCCCCAGTTCAGGGCCCAGGGCCATAGCAAGGTGCCTCTGGGGGCCAGGGTCCGGAGCTAGGCAGGCCCCTGGTTTCCTGGAAATTAGGACCCTGAGCCTCCCTTTTCATGGCAACTTGGTCTTAGCCTTCAAGAGCCATATCTGAGCTGG

>non-ORI-61

AGGTATGGATCAGGGGCAGGAGTGAGAGGGGGGTGTGGGGGCTGAAAACGTATGAGGACGTTTGGGGAAACATGAGCAAGAATTTTGGATGTGGGCATTTTGGATAGTGCAAGGAGAATGGGGATATGAGGGATCCTAGGAATTTCCGGGCAGTGGAAGCATTTGGGGGCATCCAAGAGAAATGGTGGGTCCGTGGGCATAGCAGGAGATTTGTTGAGTGAATGAGGGGGTTCTGAGGGCTCCAGGAGATGCGATGATGTGTCCAGGGCGGAGAGGGTAGAGGTGGGTTGGGGGAGCCAG

>non-ORI-62

ACCTCCCCAAGCCCTAAGTCCTCTCGGGCTCTGCCCTTCAGGGCAGCGGGTGTTGGGGATGTCCAGCTCCTGAGCCCTCTTGCACGGAGGCTCTGCACGGAGGCAAGACAAGGGGTTCAGCTCTGTCCCTTATCTGAGGTGGGAGGGGGAAGGGAATTATGGAAGGAATCCAGCTGGCCTGTTAGTAAGGGTGTTCCAGTGCTAGGTGGAGGTGGAGTTTGGCTGCCTTTGGGGAGATTGGAAGGGTGAGGAATAGAGGCCTGATTTCTGAGTGTTGTTTCTGTGTTGGACAATTCTATG

>non-ORI-63

GCACGGAAATTCTGTACTACAGAAGATTAAGATAAAATATAATTTGTGAAATAATCTTAAAACTTTTGGTTGGCCTCTAGGGGACAATGAGGTACTTTGATAATGAGAGCAAAATTTTTAAAGGAGGGCAAAGATACCAGAACATGTATCTTGTTTTATTGTCTCGGGTGAAGTATCTACTTGGTGTCAGCAGCTTGAACTGAAGATGTTGGTTTGATCGTCTTCTTCTAAAGATCATTGTTCTGGAAGATACTTAAGAATCCTCAGTTTATGATTCCTCAGTGAATTCCTTTCCATTGA

>non-ORI-64

ACCTGGCCCACTGTATATAAATTTAACTGATCCAAAGATCAGCGAGAATTCCTTTCAGCTACCCAAAGTGTGAATATCCCACTGGGCTGATAGATGATAGATGCACCTGAAAGCAATAACTGTGATATATGTTGTATGGCAGACAAACCTGAGAGCAGTAACTTAAGAAATGAAAGTTGCCATGTAGCACTTGCTGCCAGGTGGGTGCTAAATGAAGAAGCTATATAAACGGCATGCTTTTTGCAAATGGTTGTTGTTCTCCTGTCCAGTCCACTGTCACTGGACTGTCCCTGTCTATAA

>non-ORI-65

TAGAGAGCGTGGGCGGGGGGGCTAGCCTCGTGCGGGCTCCTTAAGTAGCGGCTGCGTGGCTTCCCTGGCACGCTACTCTTACGACGTCACGGTCAGGTGGTGCAGAGCTGGACGGCAACGACGTCGGACGCGCCCCTTCTTGGAACAATGTCCCACCACGGAGGAGCTCCCAAGGCCTCTACGTGGGTCGTTGCTAGTCGGCGAAGCTCGACAGTGTCCCGAGCGCCAGAGAGGAGGCCGGCGGAGGAGTTGAATCGAACAGGTCCTGAGGGATATAGTGTCGGCAGAGGTGGTCGCTGG

>non-ORI-66

GTATTGAGATTAGGGTGTACCTTCGCCTCTCAATCAGCCTCCCGTCCTCAGCCTTGCCATCTCGCTAGTCCGGGACAAATCCCTAGAGCGTCTTCCTCTGCGGGTCTCAGCCCAGCCCGGGGTTGGCTCCTCCTCCGCCCCGGCTTCCGCGCCCCTCCCGTGTGGCAAGGAGTACCAGGCCCGGGGACCCCGAGGGGCTTGGGGCGAAGGGTCGGGACTGGGGGCCTCCTTAACGGCTCACGGACTTGCGAGAGGTTCGGCTCGATGGCCGTGAAAGCGACGAATCCGCTCCTGTGCTGG

>non-ORI-67

CGGCCCGAGTGGCGTAGCTTGGACACTGCCGCCGCCGCCTCTCCTCCCCGCCCCCACGCCACTCTCACGGACACGCCGCCGCCACCTCTGCCGCTGCCGCCGCCGTCGCCAGCTCTCGCGAGAGGAGATAGTTCCCGCCCCGCTCAGTTTTCCCTCCCCCTGCGTGTCCCCAGCGAGCTCGCTAAGGGAGACGTGGCGAGCGCAACGGTGGAACTCGGCGAGGTCTCGCCCTTAGCCCCGCCCTAACGTCAAAGCCCTCCCCCAACCCCTCCCCTAGGGTCCCGCCCCTAACCGCTCCGC

>non-ORI-68

GGCCGAAATCAGCAGCAGCAGCCTCAGGCAGCGGCATTCAGCCTGACTTTTTATACGTTTATTTTTAAAAGTGTATCTTAGCAGGGGGGTGGGAGGTATGTTTCTTTGCTGGGACTTCTTTAAGGCTCTCTGAAGTAAATAGGGGTGGGGGGGGGCGCAGAGAGACAAGGGTGGAGAAGAGGAGAAGAAAAAGAAAGACTCTCCCTCTCCTTCCCCCCTCCTCAAGTTTAAGAATAAATCCAGCAGGCAAGTCCGCAGCCCTGAAGGTTTAACTTTTTTTCTTTGCAGCTTGTCAATGCG

>non-ORI-69

GTAGAAGAAAGGACTGAGAGCCTCAGAGATCTCTGGCCAATCCCTGGAGAAGTGGATCCCAACATGGCCTGCAGACTAAACCGTGGGCCCTGTGAGAGCTAAGCCACACTCGTGTGTTGCCACCGGATGAAAGGTGGAGGAGGCAGAAACCTAAACAAGTGAATTCAATTACATCTGAAAAAGATTAGGCTAAATTAGGATTCATTCCTTGGGCCAGTTCCAGGAAATATTTGTGAAAAGTTGGTTGAAAGAATACTACCATCTAAACCCTAATTTTGCTATTACGTAAAAGCCCCGATT

>non-ORI-70

GATGCTTGGTAAATCACCCATGATATGTTGCTTTCTGAGTATAAATTATTTGAGTGATCTGAGAGGATGGTAAGGTAGGCAGAGCCAGATATTGATTTTCTAAAAGGTCCATAATAAAGAAATTGTACTTCATTCTGCAGACAGTAGGGAGGCACTGCAAATTTAGAAACTGTGAAGGATCGTAGTCAGATAGGTGACATCTTTGGAAAGATGCATCTGACAGCAGTATGGTGTGGAGGGGAAACAGCCAGAGCTAGAGTTGGGAGATGACTGCATCAGCCTCTGAAAGACAAGAGGGAG

>non-ORI-71

AGGGGGAGAGAGAGAGAGAAGGGGAGGGAGTGAGGGAGGGAGGGAGGGAGGAAGGAAGGAAGGAAGGAAATTTTCTTCATAAATAATTTTTCTTTCTCTCAATAAATGAAAGTTCTGAAACTCTGGAGGTTTTTGTTTCAGCAATGGCAGTGCAGTGCTCTTGAAAGAAGTCTTCAGCAGTACTGCGAAGAATGTGCTAAAAGCCATGTTTCAGATACATAAATGGGGAAGGAATTCCGTAACTGAGGCAGCTTTTAAGACATAGCTGCAGCTCTCATATTGCTGTATGTATAGTTAAGG

>non-ORI-72

CGAGGACAGAGAGAGGAAGAGCGGCGTCAGGGGCTGCCGCGGCCCCGCCCAGCCCCTGACCCAGCCCGGCCCCTCCTTCCACCAGGCCCCAAAGGTTCCTGCATCCGTCAGGTCCCAGAGAGAAGTAGGAACTAGGTGCTATCCTCTCTCCTGGTGGGTAAAACAGTGATACTCCCTCATTTAGCCAAGGAGCAAATCACAGCCTTCTCGGGGGAAAGGACAGAGAAGTAGAACCCCCGGTTTGCCTTCCTGGTCTGGATTTTCTGTTTAGAGCTATTAACTTTCTGACTCATTTACTGG

>non-ORI-73

AGGGCTCCGCGCAAATCCATCTTACTCTCAATAGCTAAGTGACATGAAAGCCATAAAAGAAAAAGTGGTCAGCAATATTTAGCAGCACGACTTGGCCCCGGGCGCAGGGAGCCGTGCTATAAAAAACCGCTGGAATTTACTGGCAGCTACAAATATTTGCTTAACTTGCGTCTGGAGTTGGGGGATTTTCCGGGGAGAAGGAGAATGAGTGAGGGCTGCAAGCTGATTCTCAGGAGCCGGGATCCAAAAGGAGAAAGGCTTGATAGGCTAGAAAGGAAAAAGGCTGGGATCTTTCTTTTC

>non-ORI-74

AGTGAGTTTCCCAGACTTTGCCCAGTTTACTTCACCTGCAACTCCCAAACAGATCTTTGGGTGGGGGTGTGGGGAGCTGGGATAAGGCTTTTCTCTAGAGACCCTCAAGAGCCAACTGGGCTCTCTTTGGTTAAATTTTCAGGCAGAAACTGCTGACATCCTAGCAGCCAGGAGAAAAATGTCCTGGGAGAGACAGGTACTTGTTCCAGCAGTGAAATGGGATGCTCTGGGCTCAGGTACAAACAAGGGTGTTTAGAAACGCGGATCTCCAGGGCTCATGTGCCCATTTCAGAGTGCAGA

>non-ORI-75

CAGCTCCAAGACCTGCTGCCGGGTGTAGGCGGTTCGAGAGCGCTTAGGCTCCCCTCCGTTATAACTGGGGTTAACTGAAAACCCAGAACCCCGAAATAGAAGGCCAAGGAGGAGGGAGCGGAAGAGAGGGAAAGGAGGAGGAGAGAGAAGGTGGGGTGGGGAAGAGCAATTGGACATCATCATTATATAATAACCTGACACCAAGTTCACGCAAGATACATAAAACGGCAAAGAAAATGTTTCACAGGCTATTGACAACGGGAAACACCCGAGAGCCATAAAGAATGGTGCTGGGCATTG

>non-ORI-76

TAAGAGAAATCGACCCAAGGATGTAAATCGAGGCCATTCCATTATAACTGGATGGACACTTTTCATTTTTTCCTTCTTTCAGAGACAATCTGTTTCGTGTTTTCCTAAGAAAAATTGGAACCTTCGTAATAGCATCTAATTTGACGGGGGTTGTCGATGTGAGAGCTAAATATGCCCGCATTTACTAGGTGCGATTGTGAGAGAGAAGGTGGCCCAAGGATGGGAATGGATAGAAGCAACACCTCCACAGAACCGAGCTTTGAAAACAATAACTTCCTATTTCAGAACTATCCCCAAACA

>non-ORI-77

GGAACTGGGGCTTATAAAGTTTACTGTTTTATAACTTTTAAAAGGAAAGACGGCATCAGTGTAAGCAGTCGGTAAATGTGCAAATCTCTAGTTGCGCTTTAGCTGCTCTGAGGAGTTTCCCAATCGAGCTAGGATGGGGTAAGTACCTTCAATTTGTAGCAAATTAATTGTAGCAAAAGAAGCCAACTGGGTCCCGGGTGAAGAGTGGGGAAGGGGTGCTGGGATGGGTTAAGGGCAGAGGGTTTGGGGTCCACAGACAGACATAGCAGCGTCTTCAGCAAGTGGAGGCCTAGGACAGCC

>non-ORI-78

GTCCTCGGAGGCAGAGGGAATCCAAGGCGACCCAGTCTCTGCGGCCGCTCAGTCCACAAAAGTTGGGAGCTGGAGTAGGTGATGGGGGTGGGTAGAGTGCAGGTTGGGGACTGGGTTGCTTTTTTGTTTTTGTTTTTGTTTTTTACATTTTCTTTTATTTTTCCCATTTTTGTAAGTAAAACCAGTGAGTCTCTTAAAGACGCTTTTCCGACTGTCCGGTGCAGAGAGGGCCCCGGATCGGCCCCTCATTCCTCCTCGTCTTCCTCTTCTTCATCATCGTCCTCCTCGTCGGCCTTGTCC

>non-ORI-79

TATACTAGCAAGCCATAAAACTGAACAAAAGCCGACAAACCTTACGGCACCAGGGCTATAGGGCCCAGACAAATTACGACCGTCTAGGTAATATTTAAATAGATGCCTAAAGTAATTGCAGGGGCTCGGGTTAGTCCTCCTGTTGTAAAAGTGGTGAACGGGATAAAGTGGAAGGTGAAGATAAGAAGTCAAAAAAGAGGTAAAATTAAAAAGCTTCATTCCACAGCTTTTATTCTATAAGAACATAAACATCGTCTTTTTCCACGCACAGCAGCAATACAATATTAATTTATTCTGATT

>non-ORI-80

TCAGGTTGGGGGTTCATCTCAAAGGGAGGCAGGACGTTGAGGAGACTTTGTCCCTGGGAGAGGAAGCAGGCCGAGCCTTTGAGGGCTGGACAGTAAAGATTGCAATTTGAATTTCCAACTGTGTTTCAAGAATGAGGGGATGCAGGAGGGAAATAGGATATGAGGGGCAGGGTTATGCAGCCTGGCGGGCTGGGAATCATGGATTGTATTTAATCTTTTCAGATGCCAGGGTTTTGTGTTCTAGGAGCAAAGCAGAAGGAGGAAGAAGTCTGCTAGACCTCCCCATTCTTAGTTTGGGGC

>non-ORI-81

GGGTGGGCAGCAAGGAGGCTGAGATAATGACCTGGACAGGTGGAGAGGGGCCGTCCCAGGTGGAGATGCAAGGAGGATGCTGGGAACAAACTCTGGCTCTTGGGCTAGAGGGGAAGGGAGGGTAAAGGAGGCATTGTCTGGCACCTACCAGGTGCTGTGCTTTGCAGGGATGATCTCATCGAACCCTCACAATATCCCCATGTGGTAGGTACTTGTGTTACCCAAATTCTACAGATGAGGAAACAGGCAGAGAGGGTCAGTGACTTGGGCAAGGTCCCGTGGCTGGGTGGCAGAGAGCCT

>non-ORI-82

GGGGGGAGTTTGGGGGAAAGTGTTCAGCGTTTCCTCTCAAACGACAGGGCGGGGTGGGGTGGGGTTGGGAGGAAGGAGCTCGCAGGAGAACTGCTTGATACTTCCCGCCCCACCGCGGTTAATCACTGTCTAGAGAAGCCGAGGTTAAGTGGCTCTGACCTATCACCCATCCCGCTTAGGGTCCAGCTCAGAGAGCGGGCGCATAGGGCCTGGTGGAACAGGCTGCCCTCTGGGGATGGGGGATTGGGGCCGCTGCTCCGGGCAGAGCCGCGGGATCCGAGCCAGCAAGGATGCTGGGAC

>non-ORI-83

GAGGAGAGGGGAGCCCACCAGCAGACACTCCTCCACAGAACTGTAGGAGTGGGTGGAAAGAGCCTGGGGGCGGGGGGGAGAAAGACCACCCCCTGGTCTTGGCAGCCAACGCCTTGTTGAATACCTGCACCTACCCCTTACTATCTTATCACCGATTTCACCCAGCCTCCTTCCCATAACCCTCAGAACAACCTGGACTCCACTCACATATACTAAGGTAATAAATAAGATACTCAACATGCATTCTCACTCCAGCCCTGCAATACACGGAACCTAGTACACATTCCCACACCTGTTCTG

>non-ORI-84

AACTTATTTAATATTCACAACCCAATGAGTTGGACATGATTATTATCGCCATTTAATAGATGAGGCAACCAAGACTGGAAGAAGTTAAACAACTTGTCCAATGTCCCAAAACTGATAAATGGCAGAGCTGGATTTCACAGAAAAGTTAACAGACAAGCGAATGTGAAGAATGATACCGTTTTACAGAAGTTAAATTGTTCCTTGTAATGTGAATATGGTATTGACTTTAACTGTAGATCTTCTGGCTTTAACATACATAAATTAAAATGAGCGGGATAGTGACTAATTTCTCCCACCACT

>non-ORI-85

GGCTGTTGTCAACCATCCTGTCTGGGGACCAGCGCTGTGTGGTCCTCCTGGAGGGTCCTAGCAAACTGAGTAGGCGGGGTTTTGCCGCGCTTCTTCGAAAAGTGGTCATAGAGCTACAGGGAGGAAAGGAATCATTCTTAGCAAAATCTTGATTTGATGACCTGATCAGTTTGAACTGAACTTTTTAATACGTAGATCCCTGTTACCGAGTGATGTTCTTGATATAGATGTAAATTTTCCCATGCTTAAATTTGCTGCGACTAAAGCCTGCACCCAGAGGGGAGGAAAACGCTGTTAAAT

>non-ORI-86

TCTTTTAGTTGATATCATATCCTGTCACTGTCAGTAATACTTCCATGAACTGTGTGCTTAAAATTGTCTAGCCTTGTTTATAGGGTTTTTGCAAGAAATAACCTCTGTAGTTGGCTGCATGTGTCTATAGTGCCAGCTACTTGAGAGGCTGAAGCAGGAGGATCGCTTGAGCCTAGGAATTCAAAGCTAGCCTGGGCAACATAGGGAGACCCCCACTCTTTAAAGAGAGGAGAGAGAGAGAGAGAGAGAGAGAGAGAGAGAGAGAGAGAGAGAGAGACTCCTTGGTTGTCCATTGAGGAG

>non-ORI-87

CTTTGGAGTGTGCAATTATGATTGATACCAACCCAAGAAACACATTTGGGGCGGGGGAGGGGTTGGGGGGGACTTGACTGAAATCTCCGAGCAGCCAGTTGCAGAGAGCATTCTACATAATGATCGTAATAGGGCTAGTGATGAACGCCTCAATCCAGGAAACTCTCAGGCGCACATAATAATGAGTTTCCATTCTCATCCTACCTGTTTGAGATAAATGGTATTTGAAGAGGAAGTGGCTAATGTTTAAAGTAGATATATAGAAGCATCTTTAAGTATCAGGTTGAGCCATTGGTGGAA

>non-ORI-88

GTTGTAGGCGGGGATCGAGGGCACAGTGCTAGGGACCCGGGGGAGGGCAAGGACGTAAAGGAAAGAGACCCCTCTTCCTCAGGATCTGGGGGTTCATTTGTAGATTCTCTCAAGCCTACAATATGGGTTCTCCCCTCCTCCGCCACTAGTGCTCGCCCTTTCGCGCGCGTGGGGGACGGGCGAGGGGACCCCTCGGCCCCCACCGCGCAAGGGGCTGCGCGGCTGCTTGGGCCACTTGTTATTTTTTGCGCACAGGCTTGGATATTGGGCGTTTAGTCAGTAATTTAGCTTTGGAAATTG

>non-ORI-89

CAGGTGCCTTACTACTCGTAACATAAATGTTAGGCTTAGAGTAATTCAAGATAGCTGAAATGCTGGAAATGTGAAAGAATTAACAAGGTGGGGTTTGGGTGAAATTAGGTGCTGTTAGAGCTCAAAGCTAACAGGTTGCGGTGTCAGTGAGTTTTAGTCAAATGCTCCCAGTCTATTTAGCACTGGCTCATACAAAGGAAGTATACTGTACTACTAGTAGGAATTGTAACAATGGAGAGATGGCAGGTACCTTAAAAATCTAGGATTGCAATTTGCATAGAGTAATTCAGTGCAAGATTG

>non-ORI-90

TATTGTTCTGTGCGGTGTCAGTTTCTAGGACTTATTTTCTTTATACTTGTTGTTTGTTACTATTCCTTTTGTTACTTTGGCATGAATTTAATTATTGACACTGGATCATTAATGTGGCCAGGGCATTTGGGATGATTGGTCAGGAGACCCAGGGATCTGTAAGAAAATGTCATCCTGGGAAAGAGGAGATATAACAAGATATTGAAGATTGTGTCCAGTTCAGGGATATTAGTAAATTCTGAGAAAGATTTGAGTATCTTCAGCGAGGGTGAGTTTGAGGTTGATAGGACTACGTACAGA

>non-ORI-91

ACGTTTTACCACATGCCCTGCTTTTGTTGATTATTTAATATTTGTGACTTTAACTATGAACATTGACTACTAAGGGTCTTTGTCATGTAGACATTTGTGATTTTCCTGGGGCACAAATTTAACTTGCATATGCTGATGAGTGGGAGCTGATTAAATAGCGTAGTAATGCAGGCAGGAAACTCGTGACTGCTGCCATCTTAGTGTGGTGGTGGTTTATAATCCCTACCATTTAACTCTCAGTGAAATAACAAATTTATGAAAACCAACCTGAAAAGAAAGCCATACACTGGTTGATGAGAT

>non-ORI-92

TGTAGTAACTTTGGAAACCCGAATTATCCTGTTTTTTCTTTAGGCTAGGTCCTTGTCTTAAATCTCAAGGAGCCAGAATCACTGAAATGATAGCCTTCAGTAAAACTACCTGTACGAAATTGTGATAGGCATAGCTATTTAAGGTTAATGCTGTGATGAATAATATAACCAGAAGTTATGTTTGGAACAGTGAGAATTCTTCTGTTCCTATTGGCTACTGGCCAATACTTAGATATAGTGATATCTAGATCAGGGATTTTTCCATTTGCAGGGATTGGGGTGAGAGAGTTCATTCTTCTC

>non-ORI-93

TTTGGTGCAATTTAAAAAGCCTCCTCAGATGTATACAGCTTTGGAACATATCAATCTATTTATTTTTTGAAAACAAGTTTTACTAAGACATATTCCTTTATTTAGTTTTTTTTAATTTTTATTTTTTGTTAGTCAACAATCTTCACTATGTGTAGAACATTATACTGGAAACTTGGGAAGTTACAAAATATATAGAATATATATCCTATAATGAGATTTGGAACCCTTTGTTCATCTCCATATTCCTTGGAGGAAGTAAACTTTATAAACTATCAGAATCTTTATAAAAATAAATCTCCA

>non-ORI-94

CTGTAGACTGGCTAGCCCAAAGACCAAAAGTAGCACAGATGACCCCATTTGACAGAAAGGCCAAATTTTATCTTCATTGTCTTTTGTCAAAGGTAAGAATAGAGACTCCTCACACTACGTGGTTCGATACACTTTCTTCAATAAAATAATGAGCCTGGGTAAATTAACTATCTACTCATTCTTAGTGTTTATAAATTACAACATAACTAAACTCTGACTTCATCCAGGAGTGAGTAGATAACTTTAAACTTAAACTGAATATTTTATTTTAGAATGTAAAGTTTTCTCAAGATATGTGTC

>non-ORI-95

GCTTTGGAATGTCAGTCAAAAGGGTGGGAAAATATAATCGGGGCTGGGGCTAATGCAAAGCACTTGGTGAACCTGCCTTGGTTAATGGACAAAGCCAAAATTTAAGCAGGGAAATTGAGACTGGAGCTGCCTAGAGGGAAAGCAAAAAGCCTAGTGGTGGGACAAGGAGAGTGCAAAACTGAGGCACGGAGCCAAGAATGTGGATACTAGGAGGGGAGTCTGAACATCAGCAGATTAGGACCAATGAAGAGTTGTAGGAATTGAACCCCAACAATGATGTAGATGTCAGCTGTCACTTGT

>non-ORI-96

ATTTTATAGCTTTAAGAGAGAATCTTACAGATCAAGAAGTCCCAAGCCTGTATTTTTCCAGATATAATCATTTAAACAATCCTAACCTTTGTGAGGAAAGTATAGGAGGCCAGGCAAGGTAGACAGAGCTTGAGGCCTTGGCAGTTGCTTCCTTTTGATCTCTAGGGGAAGGGGGTGGGGGTTAATATGTATTCATTAATTAATACCAACATTGTATTGAAAAAATTGCAATTAGTCAATGATAATGGGTTCTTCTGAACATTTTACATGACAAACAAATATTCTTAAGTTTAATTTCAA

>non-ORI-97

ATCAGCAAATACGTAATGTACAAGTTCCTGACGGTGTTCCTGGCCATTCCCCTGGCCTTCATTGCGGGAATTCTCTTTGCCACCCTCAGCTGTCTGCACATCTGGTGAGACGGGGCACACCGGGTGGACCGGCTTTCTGAAACATGGGCATATTCTCCGCCACCTGCCCCCTACTCTCCTCTTATCCCAGGCCGGCGTCAGGAGGAGGAACGCGCATCAGTTCCCAAGCAGTAGGAAGAACTGGAAGGCCTTGAAAGGCAATGCGCTTCCTTTAGAATAACAGTTTGGGCTTGGAGTTTC

>non-ORI-98

ACTGTTCTGTTCATTTCCTAGCTGTTCTAATTAAGAAAACTATTAAGATGAGCAACCACATTTAGAAATGTTTATTGACAGGTCTTTTCAAATAATGCTTTTCTAATTAATAGCCAAAGATTTCATATCTAACTTTGTAACCAGAATTATACAGTAAGTTGACACCACTTAGATTTAAAGGCAGACAGTTTTGCTTTAGTACAATAGTATACATTTTATAATGATGAACTTATAATGATTAAGGGACATTTCTATAAAAATACTACAATAGTTTTATGCACAACTTCCCATTAAAAATGA

>non-ORI-99

TTGCCTCAGGTTTAAAATAATCTGCCCAAGCACCCCAGCGCGGGAGAAACGTTCTCACTCGCTCTCTGCTCGCTGCGGGCGCTCCCCGCCCTCTGCTGCCAGAACCTTGGGGATGTGCCTAGACCCGGCGCAGCACACGTCCGGGCCAACCGCGAGCAGAACAAACCTTTGGCGGGCGGCCAGGAGGCTCCCTCCCAGCCACCGCCCCCCTCCAGCGCCTTTTTTTCCCCCCATACAATACAAGATCTTCCTTCCTCAGTTCCCTTAAAGCACAGCCCAGGGAAACCTCCTCACAGTTTT

>non-ORI-100

AAGCCCAACAACAAGGCCATGGCAGACGAGCTGAGCGAGAAGCAAGTGTACGACGCGCACACCAAGGAGATCGACCTGGTCAACCGCGACCCTAAACACCTCAACGATGACGTGGTCAAGGTAAGCCAAGGCGACCAACAGGGAAGGGCTGGGACAGCTCTCCTCTGGCAGTTAGCCCGTGCATCCTTCTTTAGCATTGCCGTGTACGCACACCCCACCCCGCCCCCTACACGCGCACACACACACACACACAGAGTTTTGTGGGTTTGATGTGTGGGAGCTCCCGCAGTCGGCAGAAAC

>non-ORI-101

TCTCAGTTTTCATAAGTATTATGTCTCTTCTGAGCTATTTCATCTATTTTTGGCAGTCTGAATTTTTAAAACCCATTTAAATTTTTTTCCTTACCTTTTTATTTGCATGTGGATCAACCATCGCTTTATTGGCTGAGATATGAACATATTGTTGAAAGGTAATTTGAGAGAAATATGAAGAACTGAGGAGGAAAAAAAAAAAAAAGAAAAGAACCAACAACCTCAACTGCCTACTCCAAAATGTTGGTCATTTTATGTTAAGGGAAGAATTCCAGGGTATGGCCATGGAGTGTACAAGTA

>non-ORI-102

AGGACCGGTTCATCAACTTCTTTGTAGGCAATACCATAAATTCTTCTTATTTCCCAGATCATCCATTGCATTCGATATCAGTGAGAAGGCTAAAGGAAACGAAAGATGGTTTTATGTTTTTGACGGACCAGTCCTACATTGATGTTTTACCTGAGTTCAGAGATTCTTACCCCATTAAGTATGTCCATGCCTTTGAAAGCAACAATTTTATTTACTTCTTGACGGTCCAAAGGGAAACTCTAGATGCTCAGACTTTTCACACAAGAATAATCAGGTTCTGTTCCATAAACTCTGGATTGC

>non-ORI-103

TGTGAACATGTAGATGTTTTGTGTGTATTTTTTTAAATGAAAACTCAAAATAAGACAAGTAATTTGTTGATAAATATTTTTAAAGATAACTCAGCATGTTTGTAAAGCAGGATACATTTTACTAAAAGGTTCATTGGTTCCAATCACAGCTCATAGGTAGAGCAAAGAAAGGGTGGATGGATTGAAAAGATTAGCCTCTGTCTCGGTGGCAGGTTCCCACCTCGCAAGCAATTGGAAACAAAACTTTTGGGGAGTTTTATTTTGCATTAGGGTGTGTTTTATGTTAAGCAAAACATACTT

>non-ORI-104

CTTTCCCCATGGAGGCATCTCTCCCTCCTTCTCTCTTTCCTTTCGTGGCTCTAGCAGTAAGCTTGCAGCTCCCATGTTCCCCAGGAGGCAGCGGAGTTGCTCACAAAAAGACGACCCCCTCCCCATAGTTGTTTTCTGCCCACGTTGGCAGCAGCCCCCCCCCGGGGGTCGATTTGGTGGCACCTCACTTTCCTGCTAGGACTTCTTCCGAAGAGGGGAAAATGTGCAACTCCTTTTACTGTATATTAGATGCTGTGACAGCAATGGAACCAGATGAACGACTTAAGTTCTCAGATATTT

>non-ORI-105

CTCTGGTTGGGTAGTGGGGAGAGGGGTTCCCGAAGGCACTCTTGGGAGTGTATGGATGACGTGTCAGGTCACCAAATGGATTGTCGAAGGGACCGGAGGGAAGAGGGGAGAGCTGGAATTTCTGAGGGTTTCGGTTGCAGTTTTCAGTGATCTTTGCTCGTCTCCCTTCCCTGTAAAGCTACATATGCTCAGCAAGTGCCGCGTGCCTCCCATCCTTGGTCGGTGGTAAATGCAGGAGGTAATTAACAGGATCTTCACAAACTTGGAGCCAGCCCACTGAGTCGTTAAAAAAACACCCGC

>non-ORI-106

ACATTTCATTCTGCATCCAGTGGATATTTGTTGACATGGTGTTGGCTGAGCTGGGATATAGCCTATGGAGAAATTTCATGAAGCTGGCAAAGCTCCTGTTATTTTCTATTTCATGCTTACATATTAGTGTTTTGTTAGGTTGTTTTTCTTTTTCTTTTTTCTTTTAGATCTACACGGAAAGATTTGGCAGGTCTGATAATTTAGGCCTCTGGAAACCTCAGTTATGCTATAAAGAGTAAAGGGCAACATCATATGTTATTGTAGGGTTCACAGTTTAGGATGCTTGGTCTTATTTTGGTA

>non-ORI-107

GTCTGGTTTGACAAGGCTAGAATTTAATCCTGCAGAAGACAGGAGAGGCCGGAGGAATCTGGCCTTGAGCTGAGAGAGGGCCTTAAAAGTTACGCTTAGGAGCTGGGAATTGTTTACCTGGAATTGCAGAATTTGAAAGGTTCTTGGTCCTTTCTTTAGTATCCCTATCCCATGACTGGCTTCTCTGTATATAAATAGTTCTGGTGACAGAGACTTCTCTAGACCATCTATTCCATTTTCAGACAGCTCTAAGTATAAGAAAATTCTGAGCATTGAGTCAAAACGTACAGGTTCTACCTA

>non-ORI-108

GTGGAGTGCCACTGCAGTGGGCTGGCGAGGTGACCTCTGACACTATAGTGGAGCACATTCTGTCCTGGAGGGAGACATTTGTGAGGTTTTCTGAACATTGGTGTAAAAGAGTTAGAATTTGGTTTTTCTTTCTTAAGCAACAAATGGGAATCCTAGTGCAGAAACTTTTGTCTCCTGGGTGGGAAAACTTCTTATTTTAAACTATAACCACTTTATTTCATTTGCTTCCGGGTAATCTCTTAACTACAAGAAACTGAACCCTATGTAGACCCACCATAACCCACAGATAATCTTTCAAGG

>non-ORI-109

CTCAGGTATGACAAGCCATAACTTCCTTGGCAAAATGTCTGATGAAGACTTAGTGGTGAAAAATGACAAAGGAAGGCTGTTTGACTTGTGTTGTAGCACTATAGCAAAAGGGTGGGGGGGTATGAGTGAGAGAGAGAGAGATGGTTTATGGAGTTAACTACTTTATTTTGTTGATATTCAGAGTGACAGAGATAAGTGATCTAATATTCCTGGGATTTTACTCAAGTCTAGGTCATTTTTTTCACTTAGCAGGAAATTGAAGAGTGGTTCACTATGCCACATTTGCATAAGGATGTATAT

>non-ORI-110

AATACAGGAACCTTTAGGGAAGCTCCAGGCCAAAGAGCATCCGTGGGCTCTCGGAGCGTGCGTTCCGGATTGCCGAGGCCATCCGCTGGCAAACAAGTCCCTGAGCGTGCAATGTGAGGAAACGAGGAAGGGGGCCAGGGGATAAGGAGATTACTATGCATGGGATACAAGAATCCAGAGATGATTCTCAACTCATTGCTTCGCTTCTCTCCCTGAAGCTGGGATGAAGAGCTAGCACTTGTAAATTTGGAGCTGCCGCGTCTATGCAGGGCAAGGTTAGGGAGGGGCCAGGCCTCTGCG

>non-ORI-111

TAAGAAGAGATGGAAGAATGAACTGAAGCTGATTGAATAGAGAGCCACATCTACTTGCAACTGAAAAGTTAGAATCTCAAGACTCAAGTACGCTACTATGCACTTGTTTTATTTCATTTTTCTAAGAAACTAAAAATACTTGTTAATAAGTACCTAAGTATGGTTTATTGGTTTTCCCCCTTCATGCCTTGGACACTTGATTGTCTTCTTGGCACATACAGGTGCCATGCCTGCATATAGTAAGTGCTCAGAAAACATTTCTTGACTGAATTCAGCCAACAAAAATTTTGGGGTAGGTAG

>non-ORI-112

ATATGAGAGATCATAGAAATTCAATGTGGTATGAAAATCTGCTTGGGACTTCAGATATTGTCTCCAGTGATTGAATAAAAATAGGAGCTCACCTACTATGATGAGGTTTCTGTGTGTGTTAAAAGAAGGTTTTCATTACTTTTGAAAAGGTTATGTATCCTTGTTTTATGTTAAAACTTTGAGCTTTGTTAAATATGCAGAGTTCTCTTTCTTAGCATGGACTACAGAGGTGCAACTACCTCCTACCTGACTTCACATCTACTCCCAAATGCCTAGTGAAGGCTTAATAATTTCAAAAAG

>non-ORI-113

TACAGTGTAAGTTGAAGAAGGTAAAATCTGGATGTAGTTTCTAAACTCTGCTTGGCAGTTTTCATATTTAAGCCACTAGAAGAAAAAAATTGGGAGGGAAGCTGAGAAGAATTTACTGAAAGAAAAAAATACTTGGGAGGGAAATTGGCAAGAAGTATGAAAAAGCTTGGGAGGGAAGTAAGCAAATAAATGAGTTAATGACTGTTCTGGAAAATAAACTCTATCATGCAGATATCACATGACTGATTAAATTTGAATTTGACCTCCTGCTTTCCAGGTCTGGTAAAAACTAACCTGTAA

>non-ORI-114

GGAGGGTGGGTGCCAACATCCCCTGTTGGGGGCACTCCAGGCCTTGAGGGAGCACCTGCTTGAGGGCTTGTAAATCTTGACAGTGCTTGGGTCACAGTATTCCGAGCTAGTTGTTTGGCCACTAGGTTGTCACGACTTGTAGGCGAGACATCTCTTGACGGAGGACTTTGGGTAGTATTTCCATTTTGGTCTGGGTCGTTAGCATTGCCCTGAAATCTAAATCTAGCTGCTTGGATTCGTGGGTTTAGACCTGGATGCAAAGAGGTGTTGGCACATGGTGAATGTAAACTGTGCATAGGT

>non-ORI-115

TATTTGGGGCTCAGTAAGATCAGTTAAGTTGCACAAGTCATATAGATACTAAATGTGGAGCCAGGAAATGCAGCAAATTCAGAAGTGGAATATGACATTCTAGATTTAAGAGTTTGTCAAGGAAACTTTGGAAATCTACTGTTAAAACTCAAACTAAAAATTAAACTCCTTCACTCAAAAATGTGACTACATAAGATGCAAAAGCATTTATCAATAAAACCAAATGTAGGAGAAGATGATTCCCTCTCATCCACTGCAAATTCCTACCTGAGGTGACAAAATCATTTCCTCCACAAAATA

>non-ORI-116

GGCAGGAGAATTGCTTGAACTCAGAAGGTGGAGGTTGCAGTGAGCCAAGACGGCGCCATTGCACTCCAGCCTGGGCCACAGAGTAGTGAGACTCTGTCTTGGGGTGGGGTCGGGGGAGGGGGCAGGGAGGAAGAAAAAAAAAAAACATGATGAGGCTTAATGGTTTATGTCTAAGGGAGGGGAAGAGGCAATTTATTCAAGTTTTCAGTGCTTTTAAATAGCCACATATTTCAATCTTTATCATATTGTTTTTGCCCTAACCCTATTAGGATAACTTGCTTACATTAAGCAAAATTCTGA

>non-ORI-117

AGATTGTAGATGAGAAGACTCTTGGTGAGTTATGGGCCACAGCAGAAATGTAAAGGATCATTCCTCTTGGTGTGCAGTGAGGATAATCAGACAAGGCACCATCTGGATTCCCAGAACATGGTAATTGATAATGACAAGGTCAAAGATGGAAAGGCAGAATGCCTGGGGATACTGAGAAGACAGAGAAGCAAATAGGGGCCGCTGGCCTCAAAGACAGTGGAGCAAATTGTGTTCACCGATCCAAATTATTTTCTAGCACACCATCGCTGCCTTTTTCATCCCATTTATTATATAACCATG

>non-ORI-118

CCTCTAGAGGGCTTCGCGGGAGAGTTCTGGCTCAAACCAGGGACGGCGAGGGCAAGGGGTGAAAGGGGTTTGAGGCGAGGCAGAGGACTCAGGGGTCTGCGGACGGCTGGTCGGCCCGTGGAGACGGCCGCGGCGAGGCGCCTGAGGTACGGCGGTGCGGAGCGAGGGCGAGGTCCCGCCGGCGGCCAGGCGGCGGCGCTCACGGAGCCGCGGGCTTCCCCAGGCCCCGGGCAGCGTGCTGGAGCTCTCGGGCGGCGCCGCCGCGGAGCTTGGCGCGGCAGCTGGCAGCGGCCTGACGCG

>non-ORI-119

CAGTGCATTGCTAAATGGCAAAAGTTGAACATTTTTTTCCTGCCAACAATTTTCGAAGCATGCCTCCCGGAACTTGGGGATTCTTTTATCAGGCCCAAGGGAGGGAAAGCATCTATTGGTAGCAGATGGGTATTTATCCTGGCAGCTCCGCTTCTCAAACTTGAAAGTGGAGTTGGGTCACGTGGGCTCACGCACTTTCCCTCCTCTTCTGCGCCCCGATCCTGCTTGGAAAATCCTCGAAGATCCCAGTCATCTGTCAAATTCTGCCAACCAAGGGCCGCTTCTTGGGGGCGAGAGAGC

>non-ORI-120

TCCTAATATGCCAGACCTAGAGGATACAACTCAAAGCTTAAAAGACGTAGTTTTATCTCAGTGAAAGGAGGTACTACTTTATAAGAACATTTAGCTAAAAACAGGAAAATGCTCATGCAGGGTTTAACTAGCAAATAATAAAATATAATTAAAGTAAGGTAGGGTGTTTGAGAAAAAAGAAAATCCTAAACATTTTGAGGCTGACATCATGGAAGTCATCTAAGCTTTTTACTATGTATCCATTGGCACTACTTCCAAGGCTGAGTTGGACAGATCATCTGTGTTGCCAAATACTGCCGT

>non-ORI-121

CACTTTTCTGGAGCTGACCCTGACACCAGATACAGCTATTTGAGAAGTGGGTCAGGGTATCTCTACGTGGATACCGGAGGCAGGGTGGATGTGACCTAGCCCCGCCCTGTTGACCGCCAGTTCTGGGGTTCTGTTCCCCTGGGCCTTGATCATTGCCTTCTGGTTTTGTGTCCCTGCTGAAATCTAAGTAGTTTTAGTGAGTTCCTTTCTTTCAGGAGTTTTCTCATCTTTTTTTTCCAATTATCTGCAGGCTTCAATGTAGAAACAGTGGAATATAAGAACATCTGTTTCACAGTCTGG

>non-ORI-122

AAGCCAGACACCTAGGCGTGGTTATTGGCTACACATTGAGCTGGCTGTTTTACCAAGGTTATCAGTCTTTCAGAGATCAATAATAAAATGATATGAAGAGAACGAGGATACCTATTTTCAAAAATATGCTTCCAAAGATGTCTGTTGCCGGACATGCTGGCAGAGAATAGCTGCATGATGCAAAATTCCATTACTTTATCATATCCATCTATGTTTTAGGTATATTTCTTTACCAGGACATCATAGCCTTAAAAGTCACTCAAGGTTTCTATTGCAGCTAAGGTCAGAATCCTAAAGTTG

>non-ORI-123

TAAAATAAAACAAAACTAACCACAGACAGATTAAAGTCCAAGAAACACAGTAGACAAATTTCCTTTCATGGAAGCTACTCTGCAAGGAAATGCCGATCCTAGCATCATGTTTTAAGTTTTAGCACACTATTTGTTCTCCTCCTGGCTTAGTTTGGTTCTATTATTGTTTTACCAAGAATGATACACTTTCTTTTTAAATATGTGATTTTGAAAAGCTTTCCCTACAGATGCAATTGTTTCAAAATTGCATGTTGGGGCTACATTTCTTTGTTCCTGTGGGAATACATCTTTACTTATATA

>non-ORI-124

AGGATGAGTTAATTGAGTGAGGGGTGGGAGAGAGATGGGGTTGTAGTGGCTTGCAACCTAATCTAGTGTTTGGAGCCAGATCATGAAGAGACAGGTATACCAGGCTAATGGGTTTGGATTTTATCATGCAGGCAGTGGGCATTTAGGAAAGTTTCATAGCAGAGGGTGTGCCACGGTCATAGTTATGGTTTAGAAATCTTGCTCCATACTGGTTCTGCAGGTAGGGGATTATATGGCTTGCCTTGCGGCAATTGTCAGAGTTGCCGTTTTTACATTCGTTGGGGTGATTATTTGATTAAT

>non-ORI-125

AACTCTGATGATTCATGATGACTGAAGGGAAAGTTACTTTGATGGTTACCTTCTTGAGGGCCAGAAAAATGTCTTTTTTTGCCCACATTAGTGGTGACACAGGCTAGGCATTTAACCAATATTTGCTAATGAAAAGACAATATATAACATTGAAAACAGAAACATACAGAAGTCAATTTATATTTGGTTTAAAAATACATGGTTTTCCTCAGCAATTTATGTTCTTAGGAAATTATATCTTCTTTAGGATTACCATAGCCAGTTTGCCTTTATTTACTTATTTTTTAAAAATTTTAACAC

>non-ORI-126

GATGGATGCATGGGCAGACAGAGTGACAGGCGTCCAACATTTGCAAAAAGCTGACTCACAGGCCCCAACGCCTGGCATCCCCCCCTAAACCAGAGGCCCTGCACAGTAGAGGCTCTGGCGGGGAGACCAGCTGCTTGCAAAATGCCCCAAGGACGTGGGCTGGCCATGGCACCGTGGGTCCACGGGCAAGATGGGCAGACCGGAAGCAGCACAGCGCGTGGGACAGGTATACTCACATCTCCCTCACACTGAGCCAAGTTACCCAAAGCAGGACGGAAAGGAACAAAAACAAAGAGTTAG

>non-ORI-127

GGTCTGGGGTTATCAGGGGGCTCCATGGGCAGCTGGGAGCTGCTAGGGAGAGGGTGCCAGAGGAGGGAGAGGAAGAACCCAGGGTCGGGGTTCAGAAGGACCTGATTCAAGTCTTAGTTCTGCCACTGTCAAGATGTGTGACTTTGGGCAAGGGGCTCTGCCTCTCTGAGCATTGGTTTCCTGAGCTGAAAAAAGGGAATAATAGCACCTCCCCCATAGGTTATGAGGATGAAAGAGGAAAGATGTGGGTTAGACATAAAGGCTGGGTCGGCTACAGTGCTGCTGCCCCACGTAAGGCAC

>non-ORI-128

GCGAGAGGGCAGTGGGGCGGAGCTACATGGGTGGGGGCGAGGCCTTGGCGCGGCCAGAGCGGGGGTTGGTCTGGGGAGCAGGTTGTGCTGGGCAGGGCTCGCTGCTGGGCAGTGCTCGCTGCTGCCCCCATGCGCCTGCCGCCGCGGATTGGGCCGGCCTCTTTTTTTCCTTTTACTAAAATTATTAAAACTGGAACACATGTGCTCTTAGATGATTGCAGGTACACGCCATAATTACATATTATTTGCCATCTTACCAATGCTTTTCAAAAGTTTTAATTGATAAATATATATTTACGG

>non-ORI-129

CATGGTGGGACTATTGAGGGGTCCGGCATGGGGTGAATAGGAGACTGGGCAGAGTGGCTCAAGGGAGTCGGGGACGTTGTTGGGGTCCAGACGGAATGAGTGAGTGCACAGGCCAGAGTCATTAGTGGGGTGGTCTAGCGCAGGGGAGGAGGCGCGTAGGATGATATGCTTAGGCACCCAGGCTGAGGGTGCTGTAAGTGCTGTGGTGACGGTGGAATGAGGAGGTGTCCGCCTAAGATTGTAGGGCACAAGAGATGGCTGCCCAAGTGGGGCCAGGCCCTACACAGCAGTAGAAGTGTC

>non-ORI-130

ACTACGGGTGGGGTGGGGACAGCTGTAGGTCACTGAACACCAGATGTCTCCACATCTGACCCCCATCCTGTTTTGCAGGAACAGACAACGCAAGCTGGGGACGAAACTGCAGTGACCAGTGGGTGTGGCTGGGTCCAGCCTCCAGATCTGGCCCGCACGATGCCTTGCAGGATGGACAGGATGACAGACAGGGACGAAGCAGAGACCTCTACAGACCCAAGTCACCAAGTGGAGCCTTTTTTTTTCTTATTTTAATTTTAATGAACAAGGTGGACCAAAGGGCTGAACCAGCCCCTCAAC

>non-ORI-131

GCCCGTCCGCGGACACGCAGTCCGCTCCGCCCCACACACCGGGCAAAGTCCGCGCCGCCGCCGCCGCGGCTGGGGTCGGTGGGTCCTTGCTAGAGCCTTCGGGCCAAGGTCGCTGAGTTACAGCCGCCAGCCGGTAGAGGCAGCCCCGCGCCCACCCTCTGGGCCGAGCGGGCTGCGGGAAGGCACCCGGGGAGGAGGACTCGCGAGGCGGGGCCTGGGCCGGTAGCGGGCCCCGGGCGGGCAACGGTGCCCGGGAGGTTGGCTGTGGGGCGGGGACGGGGCATCGATGGGGCGGAGTCT

>non-ORI-132

ATGGCAGGCAGAGGGCTGTCGGATACCTGGGTTTGGTCATACCCACCTTGGTCCTCTTGCAAATTGACCCTTTCCTCATCTCTTCTAGCTCTGCTGGTGGCAGGTGCAGACCACGTGTCCTGTGGGGGGGCCTGGGGGAGAGGGGGTGGGCCTGGAGGGGTGGGGCCTAGCTGCTGCTGCTATTTATCTGAACCCAAGTGAATCCCTGTGCTGGAGAGGCGCTCTGAGGCTCCTGGGGAGTCGGTGGGAACGACACCAGAAGCTCAGGTGGAGATCTTATCTTCCTGAGGCTGCTGGATT

>non-ORI-133

GCAGAGAATGTAGGGCCTGACTCCTTGACTTTTTGGTCGAGGGGCCTCTGGGAATGCTTGCGGAAGGGAAGTCCTCCCCATTTGTCTGACAGGAAAAACCAAAACATCGTGAATTTTGACCTTCTCTGATGGAGAGAGTTCCGAGGCCAGGCATGACCCAGGGAGTGTGTGGACCTCGCGACCAGCTGAGGCTCAAAAGCGTCCTGGGAGCCGAAACGCTGAGTGCCCTAATCACCAGCTCTGTAACCTTTCCTTTCGTGCCTAACTAATAATAATGACAATAGGGACTATTATTATGGG

>non-ORI-134

TGTGCAGCTTCATAAAAACCCTTGGTCTTTTTTGAGGTGGGCTGGGAAGGGGAGGCACCACAGCTGTCGTCAGGAAAACCTTGCTGAAGGAGGCAATGGAACAGCTGCACTATTTGGTGCCCGGGGGAGCGGTGTGGCTTGGGGGGCACATAGTAGGAATTCACAAACGTTTATAGAATGGGTGGTTTGCATGCCAGGCTTCTGCTGGGATGCATAAAGGTTCAGGGCAGCCAGTGTGTGCATGTGTGTGAGAATGTATGTGCCCACGTGTCAGGAGAAGGTACCCTCAGATCTGCACCC

>non-ORI-135

CACCAGCTGCCCCTTTGTTCAGAACAAGGCAGATCTGAACTGGGTGGGACACGCGGCTTTGATTTAGTTTTTCTTCCGGGGGAGGGGGCGGGCTGGGGTGGGAAGGGATGGGACTTGGGTGACTCCTGTCGTCTAGCCTGAGATCTTCCAGTCTGGAAGCAGCTGCTGTCATTCATTCATTCAACAAACACATCGACCTGCGCCAGGCATGGGCCATGGAGCAGGAAACGAAGATAGTCCCTGTCCTCTTGAAGTTTATGGCCTGGAGGGGGAGATGACATCTAACAGATGAAGACGTCA

>non-ORI-136

AGCATCTTGGGCTGACTGTGGAGGGCCTTTGCTGGGCTTGGCAGGTGGGCATCAGGATGGGTAACTGCAGAGAAGAGGGAAGGGGGTGGTCTGAGGGCAGGGGGTGGCCTGAGGGTGGCCGGAGGAGTTCAGGAAGAGCACTGGAGCACGAGTCTCCTTTAGGGGACTCACCTTCTCTTTCTGTAAAGGGAAGGGACAGGGCTGGGTATCCTGAGGGGCACTGGTGGTCTTATGGGGTGGGGACATGGTGGGAAGGGGCCTGGCCCGCTCTGGTCTCCAGCCTGTGTCCCAAACCCACCT

>non-ORI-137

TTCGGCCCCGTTACACGCGGGAAGCCGGGGATGGTGGTTTTTCCCCCATCGCACCACCGCTTTCTTTTCTTGGGCTTCCAGGGCTTATAACTTAATTGGTACGGTGTCATTTAAGTTTCGCATTAGCCCATGAAGTGGGTCCCGGGTTCACACCCATTGTGCAGATGGGGAAACTGAGGTTTGCGGGAGTGAAGTCCCTTGGGGAGGGTTGCAGAGAGAGTGGGTGGGGATTGGGTCCCTCCAGAGCCCTGTCTTTGATCCCCACGGCCCTCTGCTGTCTCTCTGGCTCTCAGCGGTGGG

>non-ORI-138

GCTGGGGTTGAGCGCTCAGGGTGTGGAAAGAGCTGAGTTCTAATCCCTGCTCGGCCTCTTCCTGGCCGTGTGGCCCCCAAGCCCGGAAGCCCCTGCACAGCGCCGAGCCTCGGGGTCCCCTCTGCGCAGTGGGGCTGTGGCAGCACCTGCCTGCGGGGCTGACAGTCCTTCCTGGTGGCCTGGTAGTCCCTGTCCACCCCTCCACCTCACGGATGGGAAACGGGAGCTGCTGAGAGGGGAAGTGGCCGGTGGGCACAGAAGGGATGGAGCGGGGAGGGGCACCAGCCATTTCACAAGCTC

>non-ORI-139

TCCTGGTGCCCGTGGCAGGAAACGCAGCCATGTGAGCGTGAATCCTTTTGGGGGAAGCCCTTGGCGAGGAACAGAATGCAGTGATGGCAGCAGATTCTATTAAAAGCAATTCTCATTACGGTACCATTAATTATTAATAGGATTGGGCAAATAAAAGATGGGAAGACCCAGCTGGGCCTGACTCAAACTTTGCCTCCGAAGCAAATGGCTTTTGGTTCCCATCCAGGAGCCCCAGCCATGAACGTGCCTGTTGGAAAAGTAATGGCAACCCAGAGGCTGCCATGGTGGGACGGGCACCAC

>non-ORI-140

ACACCCATTGCCCAGATGGGGAAACCGAGGGCTGCGTGTGTTCGTGGTGGGGGCGGGGAAGAGGTGGATGGAGAGGGCAAAACGGCATTGTTTGTGTCAACTGCAGAACAGAATCTTGTATTCAAGAGGCAGTCTCGTATTTGAAAGGCACTCACCTGTTCTGGGCACTGGGAGATGAGTGGGTGGGGACAGAGAGGGGGCAGCCGAGGCTCCCTCAGGAGCTCACAGTGGGTGCAGGGGGTGGAAAAGGAATGCTCCTGGGCCTATCGGGTCAGTAAACCCAGACTCTACATTTGCTAG

>non-ORI-141

AGAATGTTCTGGTCTTTATCTGGTTTAGTTTTAAGTGCTTCCAACGGCAGCAGGGCTGTTCCACCACAGTCCCCAGGAAATTGGTCCGTGTAGGACGGAGCCTTCCCGAGAATGAAGTCATCTGTTCCTTTTTTATTTTTTGTTGCCTTTCTCCTTCAACGCGAGTCACCGGGCCTCTGCAGTCCCTCCCCGCGCCAACGCGCCCCGCCGCGGGGCCCGGACGGGGTGGCGGGGGAAGGGGCGAGGAGCAGGGCCCCGGGGTGCGCGGCCGCCCGACGCCCCCTGCTCTCGCCCTGCCCC

>non-ORI-142

GGGTATTGGGTGCATAGAAAATGGCGGCCACAGCTAAAGAAGATAGGGAAGAGGAGGGTGGGGGAGTGATCTAATGATCGGGAAGGTGGGTGGATGAAGCTGAGGGGTTATACGGATGGAGACTGGGTGTATGGGACAGAGAAATGGATGTAGAAACTTGTGCTGGGTGGGGAGGAGGGCTGGACGGATATAAAATGTACGTTGGATGAGTGGGACAGGGAGAGAGCTGAATTTATGAATACAATGCTTCTTGATGTACAATGGGGCTACAGTCCAACAAACCCATCATAAGTTTAAAGT

>non-ORI-143

GGGGGGGGAATAAGGGGGACAAAGCAGGGTTCAAGAATCAGCAGAAAAATTGGATTTTAGGTGCTCCCGGTACAGTTATGTACTGTGCAGTTATGTCCTTCCATCTTCCATGAGCAAGGTACTCAGCTGGGGGGTTTTTCACTTCACTGGTCACGTCTGATCTGACCTCAGACACTCCCTGTGTGGTACTAAGCTCTAATGACACTGCTGCAGGGGGCACTTGCAGGCATTTCTGGATTGGGCGATTGGACAGGTTCGCAGCCTTTCTAATTAAAGCACAGACTCAAAGCTGACAGCAGC

>non-ORI-144

GTACCTGGAAATGACACTGCTACAACTCACACCACATTTCAATCAAGGTCCATAAATAAAAACCCATTTTAAATGTGCCAGGGAGCCCAAGGTTCTGAGTGCCCAAGGAGGCACCGAAGACCCCTCCTGTGGGCTGAAAAGCTCCCGATTATCCAGCCTGGCCCACACAGTCCCCTGTACACAGGGCTTCCGAGTGCAGGTCACAGGGAACACAGACTCCATGGTGAATGAATGAATGAATGAATGAATGAATGAGGGAAATAAGGGAGGAACAGGCCAATGGGAATCACCCCAGAGCCC

>non-ORI-145

AAGCCCGAGGCCTCGATGGGCTCCTCGCAGTCGCTGTCGTCCCGCTCGGCCTGGCACGGGCCCCCAGAGGGCGGGCGGCGCGCGGCGGGCGGGGGCAGCGTGTCTTGGGTGGCGCCCACTCCCGTGAGGAAGGGGTAGAAGGTAGGGGGCGGGGGCACGAAGGGGGATCGGGTGGCCACGGGAGGGGGGTCTAAGGAGTCCTCCGTGATAATGGGCAATATTAACTCTCCTCCTAGAACAAGAGAGAGAAGAGAAAAGAGAGGGCGTCAGCGAGGGCCAGGGCGCAGGCGGCCGGCACAG

>non-ORI-146

GAAGCTCATAGCTCCCCATTTCATCAAGAAAGAGACTGAGGCTCAGCATAGTTAAATAACATCTCAAAATCTTGCAGCTCATGAGAAGGAAGCCCCAGATTTGAACCCCAGCTAGTCTGACTTCAAAGCCAGTGTTCTCTCCCTTTGCCCACCAGTCTAGGAAGACCCTGACTCAAGGAGGTGGGGGTGTGGGATGATCCAAGAGGCTGCTGAGGCCTACCTTCAACACCAAAGGCATCTAACCAAGCCAACGAAGGCCCATTAGGTACTAAGCCTGAGCCCCTGTTTCACTTCCCTGCA

>non-ORI-147

TTCCCGGAAAGGAGCCTTGTCTGGGAGGCCTGGAGGACTAGTACTCCATGGCCTGCAGAGAAGGGAAGGGTAGAGAACAATCTTGCATTCTTTAGAACATAAATGGAGCAGATGAGATTCATAAAATGCTTAATTAAATAATTAGCCACTAATGACTCCTCACAAAGCACTTCTTGTGAGGAGAGGGGTTCAAGGGAGGACTTAAAGAAGGAGAGAAACAGATGAGGCCTAAGGGCAAAAGCCTAGACAGGGAGAAAATGTCAGTCTGGGGGTGGGAGAACTCTGGAGTCAGGAACTAGG

>non-ORI-148

GCCACCTCTGCACCACCCCTCGCAACGCACGTGCACACTGAGATGTGTATCGGCTTGGGTATGGAAACATCCACGTGCCCATATGGGCACAGTGTGCGGGTAGGCACACAGGCTCAGACACATACATGCATGCATGTGCTCACAGACACTGGCACACCAACTTACTCTCAGGGGAGCATATACACATGCTCACAGGGCATACATCCGTGCACACACACGTTTGATCACAGGACATGAATGGCCAACATACACACAGACACCCCTGCTCCCCAAGCTCACCCTCCAAAATAGGGTCCACAG

>non-ORI-149

TGCTGCAGGCTCAGGAGGGAAGAGCTGGGTTGGGAGAAGCCACCACCGGAGGCTGAAAGCAGGATCAATATGCATTTCCTCTGGAAGTCACTGTCCTTCCGGCTGGCAGCAGCACCAGGCCAGGAGGCTGCAAGGCGGAGGAGTGCAGCCCCCACCCTGAGGCACAAGCCCAGCAGGGCCCTCGGACTGCCCAGGGCCCTCCCTCCCTTCCCCGACAGCCAGCAACTCTGTGCTGCCCCTCTCCCTCCCACGATTCCTGCAGTGAGGGCCCTGTTACTGGAAGCGTCCAAGCAGGGGCTG

>non-ORI-150

CCACTCACCCAAGCCTAGGTGAAGACCCACAGCCAGCAGACAGCCATTACCTTTTCCTTGATGCAGGCGATAGACCCCAGAGCCCACAGGCGTACTTCCCAGGTACTGATCTTGATGGAAGGAAAGAGCAGGGACTTAGCAGGACATTGGAACTGGCCAGGGAAGCCACCCCTGTGATAACCTCAGTTGCTATCCTTACGCGAGTTCTGAAAACCATGCCTGTAAAAGGCAGGTCTGTGCTTACCCAACACAGAATCACTCCCTGAACCAGAATGGAGTCTTGCTCCTTCCAGAGCCACA

>non-ORI-151

GTTCGGTGGGCTGGGAGGTGTCGCAGCGCGCACTTCATTTGTTACTGCGTGAACTGAGCTCCGAGTCCAGAAAGGAGGGGATGGGGTGGGGCGAACTCTATGACGGAGCGACGGCGAGCTCTCTCATTGGCTGCGGTGGTGGACTCAATCAATCTCGCCTGCGTTGTAGTGTCGCGGGATTGGCTGTCGCTGGTTCCAGCCTTTTTCTACTCCCCTCCCCCAGGGGGCAGTTTCTCCGGGATTAAGCGAGAGTCGCGGCGGAAAAGGCCCAGCCGGAACGAGATTTACGGCGGGGGAGTC

>non-ORI-152

GTCACCCACTCCTAACCCTCTGCAGATTTCCTCCGGGATGCTCCGAGATGGGCTGGACCTCTGGGAGGTTCCCAGAGGGTCGGAAGGGAGGTCCTGCTCTGATCCGGGGCCAGTTTCGTCAGGAAGAGGGCGGGGCTCAGGATGCTCATAGGCTGGGGGCGGAGTTTTGTGTCCCAGACTCGGGATACGAAGGAGAGGAAACTAGGATTTCCAAATTCTGGAGCAGGACTGAAGTTATTTGGGGCAGGGAGCTTGGATTCGCAAGATATGGAATTCTGAAGTGCGGAAATATACTCCTAG

>non-ORI-153

GTGTGTGTGTTGGTGGGGGGAAGGTCTGCAAGTGTGCGATACCTACAGATCTGTGTGTTTTGCAGTGCCAATGCGGTCGGGGTGGTCTGCAGCGCTCCCTGGGTCTCTCCATGAGTTGTGTGTCTCAATGCATCCGAGAGGAAGGAGAAACAGAGCCCGTGTATCAGTGGGTGACATGCATGAGGGTCTGTGTGTGTCCTGCGTCAGGTGTGGCCTGTAAGCAAGGAAATCTGTAAGGGACTCTAGGCGGTGCACTGGGTACATGCGTCTGCGGTCGATACCTGAGCACAAGTTGGTATG

>non-ORI-154

GAGAGGGGCTAGGGACGCCCGCGGCCCCGGAGCCGGATGGGAATTGTAGTTCGCTCTCTCCATGGTTTGCCCGCGCTTGGCACCCATCGCCCCCCCGCCCCCGCCCAAAAAAAAGTTTCTGAATCTTACTTTGATTGTATCATTAGAGTTGATGTGGGAGACAGGAAGTTGGGGTAGGGATGGTCACGCCCATTTGGCAGATGGACACACTGAGGCTTGGGAAGGAGGAAGGCGAATTGAGGGGCTGAGGACAAGGTGGCTCTTAAGTTAGACCCTGGGGTGGAGGTGGAGCTGGGCTGG

>non-ORI-155

GAGTCTGAGGCCTGACAGCCCCAAGAGGGTTAGAGTCCAGGGCCCTACCATCCAGCCCATGTATCCTGAACACCTACTATGCTGGTGGGTGGGGGAAGCAGCCAGGGAGGATGAGTAAGTCATGGACCTTGCCCTCAAGGAGTCTGAAACTAGCACAGGATTAGATAAGCACACAGGGCTCCTTAACTCTGTAGGGAATGAGATAAGGGTGGCGGGAGAGGCCCACGAAGCCGGGGTGTGGGCAGAGAGGGGAGCAGCGCCTGCAGAGTCATGGGATGGTCTGCAGGGAGGGGTTGCTGA

>non-ORI-156

ATGGCAGAGTCCAGAATACCCATGTTCACCCTGGATGAGCAGAGTTCAAAGGGACAGTGTCCAAGAGAGGGGCTGACCTAATGCCAGATGGGACCCCCCCCCCCCACCACCAACCCCAAACACCTAGTGTAGCTGTGCCTCTCTGTGAGGCACAAGGCTGAGCCCCTGGCTAAAATGTCCATGCTGGAGTTGTACCCCTGCCAACCTGGAAGCTCCTGGAGGGCTGTACCCCACCTGCCTCCCCCGTCACCCCAGGTGCCACCCAGAAAGGGCCTCAGTAAACTGGTTGGATCCAAGAAT

>non-ORI-157

TGGTGGGGGTGGGGGAAACTCATTTCTTTCCATCCCCTGAAGCTTCTTTTCACTTTATATCCTGGCTTGATGCAGATGTAATATATTCTGTCGGTGAAAAATTTGGGCTGAAAAATATTGTGGCCTTTGAAAATTGAGGAGGGAATTGGGTGGGGGTTTTCCAAATTAATGGCAATGTGCAGATGAATCCACTGGGCACAGTGGGGTAGAAGTAGGGAGGGTGGTAGAAGTGTATTTCTAGACCGATGACTGCATATAAAGCAATGCTTGAGTGAAGAAAACAGTAGAGTAGGTAGAAAT

>non-ORI-158

AGCTCCAGAGTCAATCATATAACTCACTTTGCAGCATGCAGCTCAGCTTTTGAGGGGTGGTATCTCTGCTGCTGTCTAGAAATTCTGCATCAAGGTAACTGAAAAGAAAAACAGTTACCTACCAATGAAATGTTGTCCCCTACAGCCTTCCATTAACATGTTCTGTTTAGTACTGACATACGAAACCAGGAAGCGCCTTAATGAACTTGCCGAGCCTTTTGTGCTATGACATTGCTCCCTTTGAGCCCAGCCTCTTGAGAAACAGACACCATTCTGTGCAAGGCGGATGCTTGAGGTTGA

>non-ORI-159

AGGAGGATGCTCAGGGCTGCCTGTGGGCTCCTGAGTGGAGAGACTTGAGGTTGACCAGTCCATTTCCACTAACTTTGTAGGATGCCTACCTTAGAGGACAAAGGAGGCTGGTGGCCTTCTCTGAGCAGGGCATTATGGTGGCCCCAAAAGGATCACAGGGCCCTTTCTGCCAGCCCCCAGGCCTGTACTTTGTAAGAACTGCAAATTGTGTCCTCATTGTCTGTGTGTATGTGTTTCAGTGTGTGTGTCTTTCTTCTTATCTCTTCATCATCTGGGTCACCTGCCCACCTCTGGACAAAG

>non-ORI-160

GTGCAAACCTTTACAAATGAGGCAAAAACACAGCGGAATAAACTCCAGTCTAGGATTCTGTAGATTCTGGGCAAGGCATTTAATGTTTCTCGCTGCATGCTCTTACGTAAAATGTGTACAGTTGCAGCCCTGGAGATTCCGCATTGGCTCTGACAGTGTGTCTGCCCCTACAGAACTCATGTGAGTCGAGAGACTGATAAGTAAACAGATTATTATAATACAGTCTCAGAATGCAGTGGCAGTAGTGTGTAAAAGACGCAATGGTAAGAGTAGAGTGGACTCAGCTGGGGTTACCCAAGG

>non-ORI-161

TTCTAGCTACAAAACCATGGAAAAGATAATGAAGTAATGAACCTTGTTGCCTCTGTTTCCTCTTCTGTAAAATGAGAATAATAATATCTCTCCATCTGATAGAAATGTTATGCTGATTAAATGAGAAATGGTGTGAAGTGCATATCTGTGTTTGGCCCATAGAAATAACTCAATATTTGTTAACTATAATTAGCTAACTGTCATTATAACTGGTATTGTTCTCTGAGCTAGGAACCAAGCAGGTAAGGATTAGTGCAAATGGAGGTGACCTTATAGCAGAGGAGCATAAGAGCTCAAAAG

>non-ORI-162

CTGGCCGCGGCGGCGGTGGTGGCAGCGGTGGTGGCGGCTGCGGCGCAGGCAGGGCTGGGCGCTTGGGGAGCTCCGCGCGCGGGGCACTTCCCCGTGACCGGCGGCCGCGCAGCTCAGGGAACTCCGCTCCGTGAGGCTTCCGGGAGAGGGAAAGCTGTTCTCCTTTGATGCTCTTTGGTTTTACAGTGGAAAACTACAATTAAAAAAACATAGCCGTGAAAATAAATGAGATTAGAACCTGCTGGCTTCACTTCCTGGAGGCTTTTTGTTGTGTCTTGAAACTAGGATCTTTGCGGGTCA

>non-ORI-163

TAGGTGCTGGGGGACAGGCCTAAAGGCAGTTGTACTTCCGAGAGCTTTTCCCGGCAGATTCCCAGATCTGCAGGTGAATGAGGTGAAGGAGCCCAAAGGGGCCGTCTGGATGGCAGCTCCTGACCTTCTCCATCTGTGCTGCTGCCATGTCCTCTCTAATGCTCCAGCCGGGGGTGCTGCAGATGCTGGGGGACAGAAAACAGAGCCAATCAAATGCCTGGCTTTGCAAACTGGAAAAAAAAGTCCATTGCTTTTGCAATTTGAAGGGCTCACAGGAGCAGAAAATTAGTGGTGAAGAGT

>non-ORI-164

ATGGGACAGTGGCAGTCCCAGCAGTGTGTGGGGTAAACCTACTAGATGGTGGAGAAATGGGGGCGGGGGGGGGCAGGTGGCCCATGTTCCGGTGTGGGAAGGCATAGCGTGCGGGCACTGGCCTCCCCAGGTGAGACAGTGGCCCGCAGCGGCATCCTGTGCATACCTGCACCTACGGTGTTGTGCAGTGAGTAAAAGGCTCCGGCATGAGTTCGGTCCTGTCGGGAAGTTGGTTGGGAGTTTTAGGGACGTGGCAATAACTGCGTCCCAAGCAGCTCCTCCATTCAGGGCCCGTGACGG

>non-ORI-165

TTGTGGTTTCGCTGCTGGCTGGGGAACCCCCTCCGGCTCCTCACTTCCCCACCAAGCGCTGGTCACAGAAACGCCCTTCTCTGGTCACTGCTCTCTGGCCTGGCTTGGTGGTGGCTTTGGTGGCAGCCCCGTCACCCCTGGGATCCGTGTCCTCCTGTTTCTTTCGTTTCTCCTGAAGGTTTTCCAAGTGCCTGTTTGCTTCTGGGAATTTCCTCTCTGCCTTCCTCCGACTTCCTCATAGCCGTTCTTTTATTCCGGCCTCCCAAGATTGCGTGGAGCCCGGCCACTGCCAGGGCGGCC

>non-ORI-166

GTTGAGTAGGGGGGAAAGCGCCAAGAAGTGTTGAGTGCGGGGGAAAGTGCCCAGCGGTGTTGAGTGCAAGGGAAAGCGCCCAGCAGCATTGAGCACGGCAGATTTCTTCTCTGCCCTCTTCAGTCCATCTCCTTGGAGTTCTGCAAAACAGAATTTAATTTCTGTTGTTCTGTCCTGACCAAAACCAGGCTCCAGCTTGGAGGCTGACCTCCTCGTACACAAAGAAGTGTGTACTGTTGCTCACGGGGGACACACTGAGGTCAAGACGGGTGGACAAGCCCAAAAGGCAGGTTCATCTGA

>non-ORI-167

TGGAGGCCCCTTGTTCCATGTCTTCTGAAACTGCCGTGGCCCTGAGATGCGGGCCAGTGATGATGTGGTCTCCTCAGGGCCCAGCAGTGCTGGGCTGGGCATCCTGATGCCACTGCAAGAATTAACTCCAACTCGAGGTCCTTGTGGACATGGGCCACCCCGGTGCCTGGCACGTTAACACATGCAACGTGCCCATAGGGGAAGAAGACAGTTCCCATCCGGGAGGGGTGAAGGACTCCCCGGGCATCTGCCCTAGATGGACACCTCCTTCTGTCACATGTGTGCTCAGTGGTTGGCGGG

>non-ORI-168

ATCCCTTGGGCACCTCGTAGGACCACCCCGGGAGCTCTGCCCACCCCTTCCTGCCAGAGCGATGGGGGGCCAGTGTCCAGGACAGCGCCTAGGGACAGGGCTGGAGGGAAGGGTAGCCTGGGGTCCGGCTGGGGTCGGGCCCACACCTGGTGGCTGGGGACAGTGCTGGAGGGGAGGGTAGCCTGGGGTCGGGCCCACATCTGCATCTTTCATCTGCCTGCTTGTCCCGGGCCAGGGTCCCCTTGGCCACAGGTCCCCAAACAGACAGCGGGAAGGCCGTGGAGCGCGGGCTGGGCGGAC

>non-ORI-169

ACTGGGCGACAGGATGCAGTGTGAACAGTGGCGTGGAAAGCGACATCCCTGGTGGAGCCACCGATACCGTTTATTTTTTATTTTATTTTTTTTGAGACGGAGTTTCACTGTTGTGCCTAGGCTGGAGTGCGGTGGTGCAATCTCAGCTCACTGCAACCTCCGCCTCCTGGGTTCAAGTGATTCTCGTCCCTCAGCCTCGTGAGTAACTGGGATTACAGGTGCCGGCCACCACGCCTGGCTAATTTTTGTATTTTTAGTAGAGACGGGGTTTCGGCATGTTGGCCAGGCTGGTCTTGAAAT

>non-ORI-170

CAGCTGCTGTGAGGAGCCCAGACATGTGGCTGGTCAGAGCACCCACTTAGCAAGCCGTGGCTCTTAATAATGTCACATCTGACTTCTTCCTCCCCTAACTCGCAAAACCTCTCTGATCCCTGAGTCCTGGAGGCCCAGGGCTGGCTTTACCAAGAAAGCCCTTTAGGCAGCTTCCCTCCAACCCTGGACTCCAGGGGATTGGAAGATAATCTGCTTAGAGTTCTGAGCGGTGAAGCCCTTCTGGCTGTCCTTGCCTCGTCTGCTCTGTGGAAAGAGAAGCGCACGGCGTGGACTGCGGCC

>non-ORI-171

TCTGCAGCTCCGCCCGGCACCGTCCATGCAGGGTGTAGACCCAGACACTCTCCTGCCTGGGAGAGCGGCCCAGCCGCTAGTCCTGTGCGCCCACCAGGTTCTTCTCTCCCAGCGTGGCCGCCATGTTGGCCGTGATCCCCGGATCCTGCCTGCTGCAGGCTCCTCTGGGAATGGCCCGCCCACCATGCTCCTCTGAAACCCTCCCTCACTTCCAGAGACCTGGGCCCCGGGCCTCACCCAAGCCCCTGCTGTGGTCACCCGGGCTGCAGGCGTCCTCTCAGGTGTCTCAGCCGTGGGCCC

>non-ORI-172

GTCCCGGGCCGTGAGGGGAGGGGAAGATCCTAGGCCGTGAAGGGAGGGTCCTGGACTATGAAGGGAGGCTCCTGGGCTGTGAGGGGAGAGTCCCGGGCCGTGAGAGGAGGGTCCTGGGCTGTGAGGGGAGGGTCCCGGGCCATGAAGGGAGGGTCCCGGGTGAAGAGCAAGCTCATGACCCTCATTTACTGGGAATCCATTGCTCTGGCCCCGTCATGAGTGGGGTTCGCAGACTGGGCCAATCCCTACAGCGTATGTGTGCTCTGGTGTCTCGGGGCAGCACAGACGTTGTTACTGCTC

>non-ORI-173

CCACAGAGGGAGAGGCTCCCATCCACTCTGAGGCCTTTCCAGAGTTCTCCTTTCACTGGTCTCCAAAGAGATTCTGTAGAACCAAGTGAAACTGCTGATACTCAATACGTTTCTCATGTTAAACAAAAACAAAAACAAAGCAGCCATTTCTTACAGATTTATTCAATACTTTCTATCTGGTTATTATGCCTCTTATTCTGTAATTATTTTTTATTATGAATTATTTATTTCCAGATCTGTCTGGCCTATAAGGCCTGTAGGCACTATAAGGGGGAGTACTGCGTCACCTTCATCTTTTTA

>non-ORI-174

CAGCTCGTCCCCGAGTCCATGGTGGTGACGCTCAGGAGGCGCTGGTTCCAGTGGGGAGGTTGGGGAAAGCACTTCCTGATGCCTTTGGGTGTGGAAAGTGGTGGGGGCAGCCAGGCTGGTTCAGTGCAAGGCCTGAGGTGAGGTTCAGGTGCGGCCCACGGGCTGTGGCAGGGACGCAGCACTCAGCAGGTGCCTCAGGGTTTGTTGGAGGTGAGGGGGTAGCAGAGCCCCTCACACAGGTCTAGGTTGCAGGTTCATGGCAGCTGGGTAGGGACGGGAAGCTCCAGTAAGATTCACCAT

>non-ORI-175

CTGGCCACTGCTGTCCCCACAATGGGGCCACCCGTGGTGCAAGGCGTGACAAGCTGCCCTCTCTAGGTAAGCAGGACTTGGGAGGCCCCTGGCCAAGCCTGTGGACCCGGCTGGGCGGCCTCTGTGGTCTCAGGTTTGGGTGTGTTTGGTCTGGTCAGGGCTCAGGGGCTGCTGGTCCACACTGGCCCCATCCTGACAATTGGAGCTTTGGGGCAAGGTCCCTGGAGAAGGGGTCACGTGGGGAGGAAACAGCCTGGGTTTTGTTGATGCTTTTCTAAGAATGGAGTACTCGTTTTCAAG

>non-ORI-176

TGACAGCGAGCTTTGGCTGGCCTAGGCTTCCACCTAAAGTGGCTTTGGGAAAACTAGCACTTAGCAACAGTCCAGGCCAGCCGAGCCCTCAAGCAGGTTTCCAAACAGGCACAGGCTCCAGCTGCCCGACCCCACCAGGGTGCCCAGCTCATGGTGCTGCGGGCGCCACAAAGCAAGCCCGTGGCAGAGACTTGCCTCCAAGCGCTCGGCCACCGAGGAGGACGCAGCGCTTACATAATGTGCCGGGATCCGGGGCTATTTTTAGCCACCATATGTGCTGTCTTAAGAGCTGAGCTCTCC

>non-ORI-177

GGGGACCAGGATCTGCAGGCTGTCTCCTCCGCACCAGCTGCTGTGGCAAATGCGTTTCAGCGCACCCTGCGCGTTCACCAACCGCCGATTACAGGGGAGGAGCTGGGCTTCGGGGCGACGAGGGAAGCTGCCGATGGGGTGGGGCGGCTCTGGGGGCGGCCTGGGCCTTTCTGTCTCTGAAGCCCGAGCTCTCTCCCTACTCCGGTGGTCCCAGGGCCACGGCGGTGGGCGGTGCCGGTCATGCAGAATATTGCAGGGGGAGGCAGCCCGTGAGGCTTCCACGACGCTGAGAGCGTCTAT

>non-ORI-178

GCCTTTCTCTCGGCCCCACGTGGCCCAGTAGCGCTCACCTTCCGTCCCTTCTTCCGCGCTCAGTAACCAATTTAGGCCGCTCCTGCAGAACTCGGGCTCCTGCCCACCGGCCCACAGCGTCCACCTGAGGCCTCGTCCTCCCAGCAAAGGTCGTCCCTCCGGAACGCGCCTCCTGCGGCCTCTCCAGAGCCCCTCCCGCGCGTCCTCTCAGCCCCGCTCGCCTCCTCCCGGGGCCTCCCTCTCCCGCCTGCCCCCAGGCCCGTCTCCCCTCGCGGGCTGAGGCAGGTTCGGGCAGCACGG

>non-ORI-179

AGTTTTTTGTGTCCCTGAAAGAAGATATCCAGGCTAACCAGGGGTGTGTGTAGCAGTAAGGGGAGAAGAGATGGGTTGGTTCACCAGTTTGATGCCAGATGAGTCTTCTCCTTTCTGTTGCTGTATGGTTAGAGCATTATATTCAGGTTCAACAGAGAGGAGGGTGAGAAACTTTGACTCTCTGGCCTCTTCTCTCACCAGAGCTCTAACACTGGTACTCAACTGGGGGAGATTTTGTCCTCCAGGTAGGGTTGCCAGATAAAATACAGGATGCACAGCTAAATTAGCATTTTAGGTAAA

>non-ORI-180

TGGGCTGCAGTCTTGGCCAGGCTACGCCAAGCTGGACCATGCCAAGCTGGTCTGTCTTGAACAGTCAGGATGAGTCATCATGAACAAGATGCCTTAATTATCTGTGTGCCTCAGTTTACTCTTCTGAAAAGATAAAATAAAACTCAGTTCCTAAGGGTATTTAAGAGGAAAAGAAATGAACTGATAGATGTGAATAGTTAACTGTGAGTCTCACAACCTAGAGGGTAGTTCACGCGGGTTCATCCCCTCCTGAGGCCATCCCCATGTGATGGGGGTAGGGGTGTTAGAGGAAGGATGCTG

>non-ORI-181

AGACATAGCACAAGCAGTATACTGGAATGGCTTTGTAGTGTAGAGGGAGTTATCTCTTTATTTATTAATCATTCTTTACCTTAGCATCTTCTAAGACATACTATGGAAGCTACAATAAGATTACATATACAATAAAGCTATCATAGTGGTAAACAAGAATAATATAGATGGGAGAAAGGATTTCTTAAAAAAAAAATCTCAAACTAATACTGTGGCTATGATTAACCATTAAATCGAACACTGAGCTCCCTAGCAGTCTCAGCAAAAAAGGAAAATGCAACAAGTTATGTGGCTCCAATC

>non-ORI-182

GGGAGGAGGGCCAGGGGCTGGTGGGGAGCCCCAGCTGTACCACCGGCTTGGGGCGGGGGGTGGCGCTGCGGCTCCCGCGGCTGGAGCCCAGGTGCCCGCCTGGCCGAGTCTGCTGTCGGCCCAGGTTAACCAACTGCAGTGCTAGAGTAACTCCAGCCAGCTTGTCACCGAGCACAAAAGTCATCAGCCAGTGAATGTTCTTTTCTGCACCACAATTAAGACGAATCAATACGACGTGCCACAAATGGTATTTTACTCTCAATAAGAACTCGAGCTGAGTTTATTCAGAATATTTTAGCG

>non-ORI-183

TGTATCAGGATGGGCTGTTCCTACAGGCCCGGGACACCTTGGGTCCGCCTCCAGCCTTGGGTCCGCCTCCAGGTGTCTGGGCTCTGTTAGCCTCCTGTCTGGCAGACCACCAGCTGGGGGCTCTCTCCAGCCCCAAGCACTGAGCGTGCTGCACCCAAAACGCAATGCCCTTCAGAAGGCTTTCACACCCCCCTACCACACCCCAAGAAAACCCTGGCGCTGCTCACCCCCTCCTAACGTCTCGGCCTGGCTTGGGAGGCCTCCCGCCATGTGACAGCAAGGACACAGGGCCAGGGCCGG

>non-ORI-184

TTTTTGGTTGTTGGCAGGTACAAGACAGCTCATAGGAGAAGGGGGAAAAAATTAAAAAAATTAAAAAAAAGCTCTGGGCAGCAGGCAGCCAATCAAAACGCCAGAGCCTGTAATGAGGGCGATTGATGGCTGTTTGGCTTTTACTGCAGGGTAATGAACTAGAATCCAGTCTGAGGAGGGGGAAAAATATGAATATTCATGAGACTGTGCTCCTGCCTTTGATGATTAAAGAAATTTTTAATATTCAAATAAGCGCTTGCCAAGTGATTAACAAAGAACAAGCACGCAGAAATATGGAAA

>non-ORI-185

GCTATGGACCCCAACCATTGAGCCCTTGGAGGGAGGGGCCTGAAGACCCCTCGGGCAGGGCGGGGGAGGCTGTCTTGCGGGCGGCGGAGAGGATGGGTATGATTTGCTCGGGATAGGCACCGTCGCGGGGAGGGGAGGAGGCCAAGCTTGAGGAGGGGCTGCTGGAGATGGAAGGAAGTGAGAGGAGACTTGCAAGTTGCAAACGTCGGGGAAACCGGAAAATTGCAGTAGCGGGGAGGCAGGTGGCGCCCAGAGGTTCGAAAGCAGAATTGGGAGGACTTCGATGTCCACCTCGCAGAC

>non-ORI-186

GGCGTGGACTGTGGAAAAGGGTTGGGGTCGACATGGGAGTTGGGGGGACAGTAAGAATCGCTTCGTTTCCCTGGAAAAGTCGCTTGGTAGTTTTTACTTTTGTTTTATGGTCGCGGGCGGCGAGGGGAGGGGAGTAGATGTTGGAGTTCAACTTCCAAAAAGTGTCTCCATATAAATCCTGGTATTACAGACTCTGGGAGGGTGAGGATGACAAAATAAACGACACTAAGACAACAGAATCAAGAGCACGTGAGGGGGTGAGAAGTGTTTGAGACCTTGTTTGAGTCCCACTTGGAAAAG

>non-ORI-187

GGGTTGCAGGCACATAGTTTTCTTGGTTGGGTGGTGGGGACTTTCACAGAGATCAGAAAGCTGAGAGGAGCAGATGTGGGGAGTGGGAATGTTTTTTGGACATGCTGAATTGTAGGTGTCTGAGGAGGATGAGTTTTTTGGACGTGCCAAATGGAAGGTGTCAATCGGACATCCAGGTGATGGGAGAAAGCTGAGAAACCACATGCCCACTGGCACAGTACTGCCCCAGCTGAGACCCCAGGAGCCATTCTGCAGACCAGGCCAGCAGGTCGTGCACCCACTGGCCAACCTTTGCTCTGT

>non-ORI-188

ACCAATCGATCAATGCTTATTCAAAAGATATATATACTTCCAACTCCTCCATCTTATTAAAGAAACTAGAAATAAACAGAACGGTAAAAAGTTATTATTTTATAAGCAGGCTAGAGTGTATCAAAAAAGCAGGCTAGAATTATGTATTATCACCAGTTTCATAATATATTGTTTTAGTTTTAATTTCAGCAATTTTGGGTTTCAATAATTCAGGATAACTACGTTGGACTCTGTTTAAATGAGTCAATCTAGTATGATCAGTCATTTACAAGAGTAGTTGTAATCAGTAGTAGTAATCAG

>non-ORI-189

AAATAAAATAAAATGTCAATACTCCAGTGAGAAAGTAACCAATATTGAGTAAATACTCCACTTTAGGGTGACTTAAATGAACAAGGGTGTAAATTCCTTAGATAGAACTAACTCAAAATAATCTGTTGGGGAGAAGGAAGAGCAAATAAGATTTCTGGCCCAGTCAATTTGAGTGGTTTTCTTTGTTTTGTTTTTGTTTTTTGTTTTTTAAACCTCCAGATCACTATCATAGTAAACAGTGATTCCTTTATCCTTGGGATAGCTCAATATATATAATTACAGTACTTAATTTTTCCCCTC

>non-ORI-190

TTCAAAATTTATTATCTCTTTCTCTCCTTACATGTTTATTTCCCAGGCCTACCCTGGTGATTAGAACAGCTGAAGGGCCTTTCTTGTTAGGCTGTCCATGCCCTAAGGATGGGTTCCTGTTTATCCTTGCCACGCAGCTGAGCTTACTGCATGTTTATATCTCCCAAGGACTGTTCTCTGCTCAGAAATGCCCTGTCAAGGGTGTGGCATCACGCAGTTTCATCCAAGTTGTTTCAGGAATTGCTGACACTGCTGGGTGCAGTTCTATCCCCTAAAGCCTAGGGTGTGGCCCTTTAACTT

>non-ORI-191

AACTCAGGGCCATTGCTCAAAGTAAATGGAAAGAGTAACTAAACAGAATCCATTCAATTTTAGAAACTGCCTCTAGCAGGCCTGACACAGTGAACACCATTATGTGAAACTATTATCCTGCTAGAGTCTGGTCATTTTACATTCCACTGTGAGCGATGGAAAAGTCCCATTAGATCATCTAATGCCCGCCTCCAGCCTCATAAAATGAGGATCGTTGCAAGATCTTCTCAGAGTGGATCCTCAGGCTAATACCAGCCTCTGTCCTTCCCAGGCCCACTATTTCCTAAACTATAACCCAGG

>non-ORI-192

CATGTGGGGAGGGTATGACAGTACATCCCTAGGTCCCTGTCCCAGCGTCGGCTACCAGCCACTCTAAGAAAGAGCAAGGTGCACGTGCCTGGCTGAAGGATTTCACTGGGAAGTGAAAGAGCCCGTGACTGTATCTGACACTCAGTTCCTGCCCGGGTGAGTCAGCCACAAGGGGCCTGCAGGACCTCCCTCCACAGCTTCCAGCCAGAAACAAAGAAGGATAGGGAAGCCCTGGTCAAGTTAATGGCAGCTAAAACGCTCCCAGTCCATTTATTGGCCACATGAGGTGGTCGTCAAGAA

>non-ORI-193

CCACGTGCCACGCTCCGGGCTCGCGAGACTTCGGCGACACCGTCCGGAGGAGGTGCTAAGGCGGCGGCCGGGAGAACGGCGCAGGCGCAGGCGCAGGCGCAGGCAGCGGAATGCGCAGGCGCGGGGCCTTCTGGAAGCCACCGAGTTCCGCGGTGCTGTCTGGAGCCGTACGGTTCGCGTTTGGGGCACAGGGGCCCCTCTTGCTGGTGCGGATGGGAAAGGCGCGGGGTCGAGTCAGCGTCCCTCTCGATGTTGGTTTCTTCGTTTTACAAATAACACTTGGGCGGGGCGCGGTGGCTC

>non-ORI-194

GCAGTGCTGGCGCCCACCCGCCGCGGCTCTCCCTGGGACCTCCGTGGTCTTCCTTCTTTATTTCTCCCGAATGTGTACTATTTCCTGATTTCAGAACGATCAGGACGAAGAGGGGAGGGATGGGCGTCTGCGCTCACTCATTCCTTCTTCCATTCCTCAATGAAACATTTACTGGGCATAAGACAGCCTAGGCATGTTTCTAGGCTATGGATACCGCAGCTGAAATAAAGAAAGCCCTCTGCCCCGTGGGGCTGACAATCTAGTGGGGGATACAGACGTGATGAAGACAGTCAGATCACA

>non-ORI-195

GGGGCGCCTTGGGGAGGTTCTAGCCCCTGAGCACCGGAGCTGCGGCCCGGGTGGAGCGGAGCAGTCCCGGGCCGGCCCGCGGCGTCTCCTGGGGTCCTTGAGTCGGACGGGCGTTTGTGCGTCTCCCGGCTTCCCATATCGCACAAAGATTGTCACTTCACTAAGCGTATTGGAAGCGTGTCGGGGCTCAGGGAACTTTTCCACAAAGCCTGACGTCCGAATCCCGGGACTCTGGCAGCTACGGGGGTCCCTGAGGCCGGTCCCTCCCCGACTCCTAAGAGAGTAGGGGGTTTCCTGCCC

>non-ORI-196

ATCCCAAGTGACTCCTGTACAGGGCGGCCCCTTCCACCCTCCTTTCCTTGGAGACAGTTGGCTGGGCTAGACCTGTGTCACGGGCTGGCAGAGCAGCCCACCTACCCATCTGTCCTCCTGGAGGGGACAGCCTGTGGCAGGAGAGCCCAGAGCAAGGCCACCAGCTACACTGTGATACCGGTGGCAGAAAGACCCAGACCACAGCGCCCGTGTGCGCCCACTGAGCCAGCCAGCTCAAGGGTGGCATGTGTACCCCTGCAGAAACAGAGCGGATGAGGATGGCTATGTGTTAACAATGTG

>non-ORI-197

TAGAGGAGACCCGTAGGGAGAGTGAACTGGGGTGGGAAACGTGGTTTAGGGCCCACTGTAACCCCGGTCCTATGTGTGGGGCCCTGAGGACATACAGGGACTAGAGGCCTGGCTGTGGGGCCGGGGCCAGGGCCAGCGGTATGACTGCCCGAGGCCTTGTGTCCTGCGATGCTTGTGGCCAAGACTGCATACATTGCTGTGACGGTGGCTGGTTAGGGGCAGGGTGCATGAGAGATTGGGTCAGCTTTCTACATGGGCCGGATGCCGGCTGTCAGTGGAGCACGTGGCTGCTGCAGAGTG

>non-ORI-198

CAGGCTCCGGGGCCCGTCGTCATGGATCTCACAGGTAACTCGCAGGATGCTGCACCCTGAACACAGGTAGGGCCCTCCCAGGCACGCTTCTGCACCCCTGAGTTCCGAGCCCTGGGCCATCACAGTCGCACACCTCATGGGTACCACACCCTACGTGGGGGCTCCTGGAGCAGGTCTCAGCCTGTAGGGGAGTTCCGACCCTAGCTGAGGTTTCCCCAATAGGGGACCTGGCTGTTCTGAAGGACCAGGTGTTTGTCCTGAAGGAAGGTGTTGATTACAGAGTGAAGATCTCCTTCAAGG

>non-ORI-199

TGGGCTCCACCCTCCTGAGTCCCAAGGCAGCCTGGACTCACAGCGCCAGGGAACACGTTAGGCCCCAATATATGGTGGCTCTCACCCGGACAGGAGCCACAACCTCCAAAGAGCCCACCGGCTAGGACCCTCTGCCTTGATTTCTCCCAGGCACCATCTAGTATCAGAGAAGGGTTCTCCTTCCCTCCGCTGCCCAGGCCAGCCCGAGAGCAAGGCCATTGGAGTGTCCAGACCCCTGCACCCCTTCCCCTGGGCTGTCTCCACAGCGGCCCTGCCAAGGGGTCCAGTCCGATCCTGCCA

>non-ORI-200

CTTACCTTTTTCTCAGTCAAGGACTTTTGTGGCCTCTGACCAGAAAGTTGTCAGGGCCCTGGCCACACCCAGACCCCACCTCAGTCTGGCCTGGCCTCCCCTGCGGGAGGACTAAACAGCCATGGGTTCCATCGAGGTGACTGGCAGAGGAGGTAACCTGCTGAGGCCAAATGCAGCTTCTCCCACTGGCCAGCTGGTAGCCTGGTCCCAAACCCACAACCCTGAGTCAACACAAGTCAGGACACACTGATAAGCACGTGCCACCATGGCCGACAGGGGGACCGAGGCCAGCGCCTACCA

>non-ORI-201

GGGGTGGCGGTTCACACGGGTGCGTGGCATCAACTCCCGGCGCTTTGCACCTGGATGGATTCCCACCATGTAAGATTTGAGCCGAAAAAAGGAAGCGATAAAGCCTAGAAAACTCGGGGAAGCTTTTTCCGTCTTAGGGCCAAGTCGGCTTTTCCAAGCGTGGCACAGAGTCCAGAAGTTCCGCCGCGTCGTGGGTGGGAGGGGGGTGGGGGCGTCTGCAAAGCCAAACTGCATAAGCGAAGTCCAAAAGACGAGCCCCCGACTGGGAGGTGGGGGACACAGCTTGCAGCTCACACCGCA

>non-ORI-202

AGACTCGGGGAGGAGACCCGCAGACCGCCGAGCTTCTGCCGCGGGAACCGCGCCCCCGGCCCCGCAATGCGCCTGCGCCTCGCGCGCCGACTTCCAGTCCCAGCAGGCCGCGGGCGGGGGCGGGGCCTCCGGGGAGAATTCGGGGGAGGGGCGGGGCCTCCGGGAAGCAATCAGGGCAGGGGCGGGGCGTCCGGGGAGTGGGGCGGGGCTCCCGGTTCCAGGCGAGTTCGCAGCTGCGCGCCGGGTCCTGGAGGCCGAGGCCGCTCCCGCCCGTTGTCCCCGCAGTCCCCGACGGGAGCG

>non-ORI-203

CACTTCAGCCAAAGTTCACTTCAGGTGTTAGCTCCATCGTGGCTGCAGCAGCCTGTGTTGTCTGTCAGCCCTGTCTGCGTCGAGGAGTGACCAGCAGGATGTGCTGGCCTAGTTTGCTGGGAAGCACAAGGAGGCTTTCCTGTCACAGCCACAGGCCTTGTTTCAGGAATAGAATGAGATAGAGAGGGATTGTTCCAAATAGGCCATTGCTGTGTCTCACACACCCTCACGCTTTGGAAGAATAAGGGAAACGCACGTGAAAAATTCCTAATTAATTGAAAATTGTTTTAGTTTTTATAA

>non-ORI-204

AAGGAAAGAAAGAAAGAAGAAAGGAAAGAAGGCAGGATGGAAGGAAGGAAGGGAGACAGGGAGGGAGAGAGGGAAGGAAGCAGGGAGGGAAGGAAGCAGGGAGGGAGGGGGGTCAATTTCAAATAAGGTTGAAACCAACTGATGCTTGTATCACCCAGAGTGCATAAAAGCCATGTGAATATAGACTCTTTTCTGTATATAACACTGACTTGAAAGTCACTTACAGATTATTAAGCATAAAACTGAGTAGCTAAACATTGGTTAACCCAACCAGGTGGGGAATATCTGACCTTCTAATTT

>non-ORI-205

CCGAGCACCGCCCCGGCAGGTGGAGGGGCTGCGGGAAATAAAATAACTTGGGCAGGAACTTGATTGGTAGCAGGTTCATCTTTGGTCCGCGGTAGGGAATCTAATGGAGATGAAGAATGGTGGGGAAGGGGGGGCGGTGTCTCCTTGGGAGCCTAACGTACGCTTCTTTATTTTGTCATTCTTTGGAGATGGGTAATCTTTCCTGGCAGGGGCAGACACAGCAAACTAAGGACGCAGGCCAAGCTCATCCTAGCTATTGTTGTCTGAGGCGCTCGCCGGGAGCCACCTGCGGGCGGGGGA

>non-ORI-206

ATTTATTGACCCCTAAGTCATTCACCAGGGCTCCTAGCCTCTGCCCCTAGGGAAGCAGGCTGTGATACTGACTTGGGCCCAGAAAGACATGCTCTTTAGTACTGACTTGTCTCCAACATACAGGCTTTTAGTGAAGTGCTTCCAGTTAGTTTAATTTGAGAATAACACGGTCAAAAATTCTTCCTATCTAGGTTTCTTGGTATATAGACTCTCATTTATTCATCGGTTGCTGTTCTTGACCATCTCTGTTTTCCTTAATTTAACCCTCGCTACTTTGAGGACATTTGAAACTTTAGGCAT

>non-ORI-207

TGGAGGCTGGAATCTGGTACGTGGAGGTCCTTTAGGAGGGGAAGCGCTTAGGGTTAGGTGTCCTGGGTTTGGGAATGAGAATGGAAGGAATTCAGAATTACTGACCCCTCCCCATCCAGGGGACAGACAGAATCGGAACTTTGGGCTCCTGAAAGGCCGAGGGAGGGGGGTGTTAAGATCCAGCCCCGTGCTTCCGAAGTGCAGAAGGAGCTGGGAAAGGAGGAGAAGACAGGGCTGCGTCCCGCACCTCTGGGTGCCGAGAGCCGAAGCTGGGGTTCCGGGGCCCGATTCTCAGCGTGA

>non-ORI-208

TGGCGAAACCCCATCTCTACTGAAAATACAAAAATTAGCCGAGCATGGTGGCACACACCTGTAATCCCAGCTACTCAGGAGGCTGAGGCAGGAGAATCGCTTGAATCCGGGAGGCGGAGGTTGCCGTGAGTCAAGATCGCACCATTGCACTGCAGCCTGGGTGACAGAGCGACTCCATGTAAAAAAAACAACAACCAAAAAACAGGATTGTGAGGCCTAGAGAGAGTTCAGTGGAAGGGCCTGGGGTTCCTACTGGACAGTTGGAAATCTGGGGAGCTGAGTTTCCCAGAAGCTGGGGGA

>non-ORI-209

TCTGTGTCTCTCTGTAAGTCTCTGCGTCTCTGTTTCTGACTCTGAGCCCATCTCTTGGGTTTCTGTCTCCTGCTTCTCTCTCTGGCCTCCGATTTTCTCTCTGTTGGACTCTCTGTGTTGAGATCCCTCTCTTTCTGGTTTTCTCAGTGTCCGAGTTCCGCTCTCTCTTTCCAATTTTCTGTCTGCTGGGGTCTCCCGCTGGACTAATCCATGCCTCCGTCTGTGTCTCTATGATTTTCATCTATAGTCTGCAGCTTTGTGGTTCATCGACGATGCCACGAATTTGTGACCTTCGAGTGT

>non-ORI-210

GCACAGTGGAGGAGGGTGCCTTCCTAGTGGGCCTGCCCAGAATTGGGCTCCGAGTGACGGGGTCATCACTTTTGGATTCTGACTGAAGGACACATCAGAAACAGGACATTATTTCCTTAGGATTGCGACTTAGGGGCAGAGAGTCAGAACCTGCAAGATTTTAAGAGGGCGTGACTTTACTTCCAGGGGCTCCGAATGAGAGTGGCCAGCCACCTGGATTAAAATATATGTATGAGCAACTTTGATTCCTTTTTTTTTTTTTGAGAAGGAGTTAGCTCTTGTCCCCCAGGCTGGAGTGCA

>non-ORI-211

CCCACAGGGCTGCGAGGGGACCAGGCAGCTCGCGGTCAAACCCCTGAGCCGCCAGGCGGGGGATGGGAGGAAAAGCTGGCCAGAGCGTTCCCTGACTCCGGATCTCGGAGGCCTCCAAGAGGTTGGGGGGGAAGGAGGGGGGATCTGGAGGCTGGGAGTGCGGGGTGGAGCGGGGAGGAGTGAGGGTTGTCTCAGACTCCAGAGTCTTTATTCAGGTGGAGCAGAGTTGCAAGGTTAGAAGAATTCTGGTGAAGGAAAGTGGAGTTTTATGTCTTGATTAGTTTGTAGGGGAGAATGTTG

>non-ORI-212

GGGGGAGGGGGGCAGGGGAGGGACGGGGGCTTTTAGTTTGCATCTTAGGTGGGAGGGGGGAGGGGGGACCCGCCGCAGTTAACCTGACTTTACGCAGCGATTTTTAACGAGGCTGGGGGGAGGGGGGCACTGGGGTGGGGACAGGGTGGGGTGGGGGGCCTGGCTCTGTTATTTACCGTGTATCATATGTAAATATCGACAGAAACTTCAATAAACTTTATTTCAAACACGTCTCCGCCTGCCCGGAGGGAAGGGATTGGATAGAGGGGGCTTAGTCTGGGTCTCTGCTTCTAGGGTTGC

>non-ORI-213

AGAGGAGAACCTGGGAGCTATAGCGGTGGAGCAAGAGGAAGTGAGTCAAGGGAATTCTATATGATGAGAGAGAAGTTGGGGAGGATGCTTCCTGGTACATTGAAGAGAGAGTGGGCTTCAAAGTGAGTTAGGGGGATCTCAAGTTGGGGGAAACAGTGTGCTGAAGATCCCGTGTGATGATGAAGGGCTACGAGGAGGGGATTTGGAGGCAGTGGGAGCTGCTGTGAGTGCAGAGGGGCGGGTGGGTGGTACAGTGTGCCCCCTGCTATGGGAGTACAGGGCAGAGATTGGAATGGTCCT

>non-ORI-214

CAGGACCTGGACCATCCAGGGCCGTGTCTGGACCATTCAGGGCAGGGCCTGGACCACCTGGGGGTGGAATTTAGACCATCTGAGGGGGTGCTTGAGCCTGCCTGGGCCAGGACCACGCATGATGCAGTCTGGACCATTGGCGGCCCTACACCACCTGGGGGATGGAGTTTCGACCAGCCGGGGTTGGATTAGACCAGCAGAGGTGAGGCCTGGGCCAGCTAAGAGTGGGCCGGAGCCCTCTGGGTTGGAGCTTAGACCAGCAGGGAAGCGCCTAGAGCATCGGGCGGGGAGTTGTGTTCA

>non-ORI-215

ATCTGCCCCAGCCCTCGGGGAGGCCCCGTAGCCTCCCGCCTCTGGACTCCACTCTGTCTTCCGGCCACCGGCCCCTTTTCCAGGCTCAGTAGAGACCTCGGATCTTTTCTGAATGGCAGGGGAGACCCCTATCCCCTTTTCCTGAATTCCAGCGAGGCCCAGTCCTCTTCTGAGCCCCAGCCAGGGCTCTGTTCTTCTCCTTCTGTGGATGCCGGGGTCCTACTGCCTCTCTTGAGACCCGGATTGAATTCCGACCTCCCCTTGGGAGCTTCCCGGGACCCCAGGTCTTCCTTTTCCGAA

>non-ORI-216

TCCTTTCTCCCTCTGTAAATACGTTTTTCTCTGTGGCTGTATGTGGGTTGCTTGGGGGTGGGATGGGAAGAGGCTCTTTGCAAACGAGGGTCCCCAGAGAAGACTGGCGGGGACCTGATGGGGTAGCTGGGGTGTGGGGTTGGGGGATGAGGTCAGGGGGTCTTGGGTGGGAGTTGGGGGCCCCTTCATTTCCCAGGTCTGGATCGATTCACTTGCCGGGAGAGACTTTTTACAACTCATCTGCAGCTCCGGGTGCGGTTGGGGGAGATAGCGAAGGGTCTGGCCTCGCTGTGATCTGAT

>non-ORI-217

CGTGGTAGCACATGCTTGTAATCCCAGCTGCTCGGGAGGCTGAGGCAGGAGAATCACTTGAACCTGGGAGGCAGAGGTTGCAGTGAACCATGACTGCACCATAGCACTCCAGGCTGGGCAACAGAGCAAGACTGTCTCAAAAAAAAAAAAAGTTATGAGACTTGCTTTACATGTCACCCAAGGGCACAGGTAAAGAATTAGACCTAGGAGTTGGGTTGATAGGGCAATGGGAAAAAAGAAAAAAATTGTTTACTGAATCAAGGGAATAATCACACCTACATCTTTGCAACTCACGTGCTT

>non-ORI-218

CTTTCGGGGAGATGATGGGTCCTTGAACAGAGCAGAGATTTGGAACCAAGGCTAAGATGTTAAATCCTAAAGGGGCCTTGAGGGGAGGGCAGGAGCGAGGCTTAGGAATCTGGGCTCTCTCAGGGATAAATGGGTAGGGTTGGGGGCCTAGTGATGACAGATATCACAATTCTAAACAGCAAGCTCCTCACAAATGGGGGTTATCATTGTTACTGCTGGAGCAGGTCGGAGGGTATCTGTATGCCAGAGGCAGTCACAGTGGTGGGCGGGCTCAGTTGAGAAATCTGGGCTGTCAGGTGA

>non-ORI-219

CTGGCCCAGGACAGTCTCCCAGCACTGAAGAAGGAAGAAATATATCAGAAAGAACTCGGGACCCGGGCACCTGGAGGCCCACGCGTCCGAGTCTCCACATCGCAAGCCTATGAGACCCTGTCAATACTTTCTCTGGGGGTCCTCGTTTTTCAAACTTTCATACCCTTGGGAGAGTGTTCCAGCACCCCAAGCTCCCCTCTCCGCCCAAACCAAGAGTCTGGACCCACCCAGCTCCATCTTTCCTTCAGGGACCCAAGAGTCCCACGCACACCCATGCCGTTCTCACCGGAAGTTGTTTGT

>non-ORI-220

GCGGGAACTCCCCAACTGGGGTGCGCTGGCGCTCGGAGGGGGCGGGGCCACAGGCCGCGAGGCTGCCGGGAGCCGATGACGCCCGAACGCCGAACCTATTGCGTCCGGGAGGAGGCGGGGCTACGGATTCGGCCGAGCCGAGAACACCCGAACGTCAAATTGCTGGCGTTCGGGAAGGGGGCGGGGCTGCGGATTCGGTGGAGCCGAGGACGCCCGAACGCCGAACTTCCTGTGCTCGGGAGGGGGCAGGGTTTTGTACTGTGGGAGTCTGAGAGCGAGGAGGTCCGAAAGCCGAATCAC

>non-ORI-221

CGGGCGGATCACCTGAGGTCAGGAGTTTGAGACCAGCCTGACCAACAGGATGAAACCTCATCTCCACTAAAAATAAAAAAAATAGCTTGGCATGGTGGCGCACACCTGTAATCCCAGCTACTCAGGAGGCCGAGGCAGGAGAATCACTTGAGCCTGGGAGGCGGAGGTTGCAGTGAGCTGAGATCGTGCCACTGCACTCCGGGCTGCCCCACACAGCGAAACTCTGTCTCAAAGAAAAAAAAAAAAAAAGGATGATGTTGTTTTACTTTTATTGACATGAAAGATGTCAAGGGTATTGTT

>non-ORI-222

AGCTAGGCGGGGCCTTGGGTTGTGGGCGGGGACGCAGGGCGGGGCCAGGGCGATGGGCGGGCCTGAAGAGTTCGTGGGAAAACGACCCCTCCTCGCCCCGCAGAAGCCTCTGCTGTCCTTGGAGGAGACTCTGAATGTGACAGAAGGAAATTTCCACCAGTTGGTGGCACTGCTACACTGCCGCAGCCTGCACAAGGTGAAGCCCCTCCCCTCCCAATTTCTTCTCCCACGGGGGGCCTCTGTCTCGACCCACAGAACTACCTCCTCCTTCTCCTTGGACCCCTGCCATTGCGCCTGAGG

>non-ORI-223

TCCAATGTCAACAAAACTTTTCTCTGTAATTTTGGCTCCGGTTGGTCTTAGGCCAATACTTTTAAATCAGTTCTTATTAGAAGAAAGGAACAATCAGCTGTTCTTTCCTTCGCCAATCCTACAACAATTCTTGTCCAAATCCTTAATTGAAACCACTGAAGTGTAACAGAAAGGGTAATCCCAAGTCTGCCATCAAATAGCTGTGAACTGGTTACTCAACAATCTGGGCCTCCATTTTCCCTTATGAAAAATGGGAATAATAAAAGCTATCTCACAGGAGTGTTCCCAGGAACATTCCTA

>non-ORI-224

AGGCTATAGCATCATGTGGTATTCAGGGAGTGCAGACGGGCTGGGGGTGCCTAAGGGATTTGGGGGAGGCTGCAGTTGGGAGTGGTTGGGGTTGGGGCTGGGGCTGGGGTGTGCTGCATCGTAGCTCTGTCCACATCCATGGGCTGTTCTGGTGACAGTGCTATGCTCTTCTCTTTAGGGTGACCGAGCTCTCTGCTCTGTTGACCCAGTCTCAGAAGCAAAATGAAGATTATGAAAAGATGATAAAGGCTCTGAGAGAGACAGTGGAGATCCTGGTACATGATCCTTTGCTCTGGAAAG

>non-ORI-225

CTCACCATGGGTGGGACTGGAGAGACCCAGGGTTCTCATGTGGGCTGCCAGATTCACACCTCCACCCTTAGGTTCTGGACTGGACCCCAGGACAACCTCCTTCTCCTCATCCCGTTCTGCCCCAAGACAGGCCCAGTATCCCCTTCCCCTCCAAAACCCACATCCCCTTGTCCCTAGGGTCCCAGTACCTTAGCCTGATTTTCCTGCTTCCAGATCTGAGTATTGATGCCATGGATGTGGCCGGAGAACAGCAGCTGGATGTGGAACACAACCTGTTCAAGCAACGACTAGATAAAGATG

>non-ORI-226

AGTCAGGAACTTGGCTCTTTGAGCTCTAGGCCTCTTGTCCTTCTAGGCCACTATTTTGCTCCTCTCTGGCCAGATTAGTTCACTCTCGAGGAGGGGCCCAGAAGGAGGCTTGTTAGCTGGGGCCAGACTGTGTTGCTGAGGCTGAATGTTCCAGTCTTCCCTCCTGCAACATATCTTGCTCTCATAGTTCTCATTGTGAAGCTCTGAATCTTTCTTCTCTCTCCTCCCTCCCCCATGCTGCAGTACATGCTGTGGAGAGTAAGTGGCCCTGCCCCCATGGAGGCCAAACCCCTACTTCTG

>non-ORI-227

CGGCCACCCCCGGATCCATGTCCTCCTCCCCACCTCACCCCGGCTCACTTCTCGATGTCCGAACAGTGCCCTGGGGCCGGGGGTAGCATTGGAGGGGGCAGGTCCCCGCGCGCCCTCACGGACAAGGTGACCTTAATGAAACCCTTGGTCCCGGCGCGGGTGTCTCGGGGATCATGCAGCGGAACCCATCTTTGGTAGAACTGGCCATCTAAGGGCGAAGGGGCGAGCCAGGGGTAAGGGGCGACCGGCGGGACGCACAGGGTGGCCGTGAGGCTATCGGAGTGACGGACAATGGCCAGG

>non-ORI-228

ATCCGTTTGGGGGAGGGACCAACTCTTTTTTTTTTTTTTTTGCAACGGAGTTTCGCTCCTGTTGCCCATGCCATGCAATGGCATGATCTCGGCTCACCGCAACCTCCGCCTCCCGGGTTCAAACGATTCTCCCGCCTCAGCCTCCCGAGTAGCTGGGATTACAGGCGTGCGCCACCATGCCCGGCCAATTTTTGTGTTTTTAGTAGAGACGGGGTTTCTCCGTGTTAATCAGGCTGGCCTCGAACTCCCGACCTCAGGTGATCCGCCCGCCTCGGCCTCCCAAATCGCTGGGATTACAGG

>non-ORI-229

TTTTTAAGTGCCTCTGATATTTTCTAAATTCTAATATTATAAATATTTTTTCGATCAAACAATATACACCTTGTAGTATTCAATAAAACTGATATATTTGAAAATAAACTTATGTCAATGAATTTCGACTAAAAACTGGGGTGCAGGGTTTAAAACTGTCCTGACAACTCATTTTGATGCATGGTTAATTCTACCCTATGTCTCCACTTCCTTAAATAGAAGACTACAAACTAACAGGCTATAAGTACTTGATGAATTGAATTCTCAGTAAGTCCTTTTAAATGTAAAAATTTGGAAAAT

>non-ORI-230

AAGGAAGGGAGGGAGGGAGGGAAGGAGGGAGGGAGGGAGGGAGGGAAGGAAGGAGGAAGGGTGGGGAGGGAGGGTTCCCTTTTCCTCCAGGCTCAGCAGTGACATGTCTGGACTGAGGGCAGCCACGGGGCTATGTACTGGTCTCTGAGCTGTCTACCTTACATCAAGGTGCTCAGAGCTGAGAGGGCTGGCCGCGAGTGTAATTTGATTAGAGCTGGATTGTTCAGCATTGTACCAATGGAAGCTCTATGTTAATTAGTGTTCTTTAAGATTTACAGTACATGGCAGGGTACAGTGGCT

>non-ORI-231

TAAACAGCCACCGGGTTCTAAATCTGATTTTGCTTAAATCCATTTCTCTAAGAAATATTATAGCATTTACTCCTTTTCATTGTATAAGACATGTTGAAAAACAGCCATTCATTTCTAAATCCTTCTTTTCAGTCACTTTTAATATTCTTTTAAAAAGAGAGAAAGGAAAAGAAAAGACTTCTTGGGACTAAAACTTATCATAGTTGGATGAAACCCAAAGGTGAATATTTATTTCAGCAAAATTCATTCGGGGCCTTGAAAGTCAAGTGTGTGAATATAAAATACACCTTTTCAGCCTGG

>non-ORI-232

GTCAGGTTGGAGCCAGAAAATGAACTTGGCTGATTACCCAGTAAGTCTGAACAAGCTGACTTTGACTTCCCTTTCACACCAAAATCCTCTTCTTCCTCGAAGGTTACCCATCCTTTCGGGTTGCTAATTTTTAATGTAGACTGGCCCTGTAGATCAAAACTGTCCTTAAAGCCATCAAGTGAAGATGAAGCCCTGCTTTTGTTATGACCAAGAGGCTGCAGAGAAGGGAAAGGACTGATAGTAACGGGCTCTTCTTTGGAGAACCATGAAGTTGCCTCTGATTCTTCAGACTTGGCTCTA

>non-ORI-233

GTTAAGTGTCAGAGCAAATCTTTTGAATGGCAGTGGAGTTTACTTAGAGGCCCTTTCTTCAAGAAGACACTCTATCCATTTTCTGAAGCCCATTCCAGGCTGCCTCAGGAATAAATTTGCCCCACATCTCACACACACAAAAAAACGTCTTTTTCCTCCACATCTTGACTATGAGCCTTTGTCTCAAGGTGTGTGAACCAGAACAAAAATCCACATCAAGAGCCCCTAAGGTTTCAGAGCCTCTGGATTTAGCTTTAATACAAATTAAACACTTTGAAGATTACACAAATACTTTTTTTT

>non-ORI-234

TAGGATGAAGCCCTAGAAGCCTCAGGCAATTGTGATCCGGTGGGCTGGATACTGAAGCCCACCCCTGCCTTGACCTCAATTTTCAGTATCTTCATCTGTAAAATGGGAACAACCTGCCTTCCTCCTAGCCCTAAAGGGGCTGCTGTCAAGATTGGCTGAGATAGCTGTTTGCAAGCTGAGCTCAATGAAAGTTCATTGTGTCCCCCTCAGTCCTATCCCAATATCGTCTCACTGCAAAGGTGGGGGGCAGCTTAACTTCAAGGGCACTTCAAGGATAGCCAGGTGGCTGTCAGCCCAGCT

>non-ORI-235

CACTTCGGGGGGGGAGGGGGTGTTATGGGAGGGGGACACATTGGGGCCTTGCTCCTCTTCCTCCTTTCTTGGCGGGTGGGAGACTCCGGGTAGCCGCACTGCAGAAGCAACAGCCCGACCGCGCCCTCCAGGGTCGTCCCTGGCCCAAGGCCAGGGGCCACAAGTTAGTTGGAAGCCGGCGTTCGGTATCAGAAGCGCTGATGGTCATATCCAATCTCAATATCTGGGTCAATCCACACCCTCTTAGAACTGTGGCCGTTCCTCCCTGTCTCTCGTTGATTTGGGAGAATATGGTTTTCT

>non-ORI-236

GTGGTGGTGGTGGTGGTCGTGGTGCTGGTTTTGTTCCATTTGCTTGCTTCCGGTGGCTTCACTCACATGCTTCCTCTGCAGTCCTGGTTCCCCACAGCCATGGGCAGAAGGTACAGCCTATATATGAGTCTCTATCAGTGCACAGGTGTCCAGTGTAGGAAAAAATAAAATGGAACATTACAGCACCCCGTTTAATAGACAACTTTTGAATTCACTTATGACCATGGTCTCCATGGTTCTAGGGGCTGTGAGGGCATCAGAGATGCCTTTTGTATTTTATATTCACACAGACTCACAGTA

>non-ORI-237

GACAACCATCGAGGAGAGGGTAGTCGTGTTGATTAAACCCCAGAGAGCCAAGAGAGCAAAGCCACATTTTGCCGGAACACAGCACCTCTTACAAAGACACCTCCATTCTTTTGGTTTCCTCCACAAATATATCAAGTGATTTTTGAGGAACTAGCTGCCACTGGCTTTCTTCCTAAGTTTTGACGAAGAACAATTTATGTCTTCCCAGAGCTCACGCTTCCGCCTCATGCGCAAAGCCTTAAAATGTCCTTCTGCATAATTTGCTAACCCCAGCACCTGTGCCTCATCTCAGCTTAATTC

>non-ORI-238

CCTGGCGAGACTGCGCTTAGTCCCCGCCTGGAGTGCCTCCCGAAGGCCTGGCTGGAAGCTAGACTCAGTCCCTTTGTAGTAGCAGCGGTGGAGCAGGGACCAGCGTCTTGGACGGGTGACCCAGACTCAATTCCGACTTTGGCCGAGGGCATGTTTGACACTGGGTGTGAAATCTAGACAGTTCCCGCGGTCATAGAGATGGGGCAGCAGCATTCAGGCAGGGCTCAGAACTGTCCGGGTCCCATCAGTGTTGGGGCGGAAGAGGAAGAGTGTCAACTGAGCCAGGCATTTATTTATTTT

>non-ORI-239

ACGTGCGGACGGAGCTCGAGACCCGGGGGACCAGGGTTTCCCTTCGCCTATGGCCGATCAGTCTTGTCTGTCTGCGAGCTGCCCAGAGTAGGGAAGGTGCTCCGGGAGGGTTCTCGGGAACGGAGGGTCTTTAGAGATGCCTTTTTCTAATCCCCTCAATTCAGAAATGGAGCCCCAGAAAGTTGAAGTTAGTAAACCAGGGCTACCCAGCGAGTGCTGGTTACCCGAGGTGATCCTCACAATACAACTAGTCTTCTGATTTCGCTCGGCGCTCTCCCGTCTATTCACGTAGATTTTTGT

>non-ORI-240

GGAAATTGGTGAAGAGAAAATTTTGCCCACCAGTGAGACTAAACAGCGCACAGTATTGGATACCTACCCTGGTGTTAGTGAAGCTGATGCAGGAGAAACTCTATCTTCTACTGGTCCTTTTGCTCTGGAACCTGATGCAACAGGAACTAGTAAGGGTATTGAATTTACCACAGCATCTACTCTCAGTTTAGTTAATAAATATGATGTTGATTTATCTTTAACTACTCAAGATACTGAACATGACATGGTAATTTCCACCAGTCCTAGTGGTGGTAGTGAAGCTGACATTGAAGGGCCTTT

>non-ORI-241

TGGTCCATGATAGTGGCCCTGATCATCGCAAACATTTTCTCTTTAGGGTAAATATGAATTTCTGCATTAATTATATATATTTATTAATGTGATGACTTAGAGGGTGAGGGGTAAACATGATCACAGGATTAAGATTCTGTTCATGGGTGGGGGGTTTGTATTTGTAGTTAAGTGGTGGGGTTTTTAAACACTGAAGGGTGCTTTAATACGACTTGTCAATGTCCTATTTTTTAAAAAATTGGAAAAGGGTAAAATATAGTATACAATTGTTATTTTCTAGTTGAATGTGGGGAATTAATT

>non-ORI-242

GTTGGAGGCTATAGAGATTTTTATAGCCTCTTCAATATGAAAGTTTCCTCTAGAACTTTGCCATCATCAGTGCTATTCTGAGGGCAGCAGCAACCTGGAAATTTAGATACATGCAGGCTCCGGCCAGATGCGTTTCTTTGTGCTTGTCTGTTGGCATTCTTCTCTTGCTGCTCCTCTTGATCAAAACGTACAGATAATAAAGGCAAGGCCAATGAAGAGGGTAGATAATAACAGCAAATCCCATACAAACAAATGCCTCTAGAAATTCTCAGTTCTGTCAGCGGGGAGGCAGTTTTGCTA

>non-ORI-243

CGACGCAAATAGCCAAGACCCTTCCATTCGTGTAAGACCCCATCTTCTTCCACCGCTAAAGAACTCTTGGCTTAGTAGAACATAAATGACTTAAATTGTTTTTGGTGTATTTACATGAGGCCCGGCACTCTAAAATTTCCAAAGGATATGGATTAGTGGATGGGATGGGGGGATGAAAGGACATCAAGTTTGACTCCATCCATACTTGGGGAACAACTGCAAAGTTTCCTGGGGGTCTATGTAGGCTGGAAAAGACATGGTCCCAAAGTTACACAGCCTTCATTTCTCAGAGATTCAGTC

>non-ORI-244

ATTGGTGCAAGATGAGTGAGAATTCAGGTGCACATCTTTGAATGACCACCTGCATATCATCAGCCCTTGGATAGCATCTCTACTTTCTTGGGAGATTGGAGGTAAGGATACAAAACACCACAGAGACTCACCGAGTCTCTAAGCAAAAAAGAGTCAACAGCAGCTGAATGTTGGTGGGCAGGGACACCCTGTGACGTTCAGGTGAAGTTCAGGACCCAGCCCTGTTGAGTTTGAGCAAGGTCAACATTTTCCTAACATCTTCTGGGTATTTCCACAGTTGAGAATACCAAATGATGTGGG

>non-ORI-245

CTGCCGAGACCCAGCCACATCCCCCACAAGCCAGCAGCCCGGCAGAAAATTACATAAAATGCAGCGCTTCGCCGAGGGGGTGCGAGTCGGGGGAGCAGCGCGCAGCTGCGGCTGACGGCTCGGCTGGATCCATCCAAGCATGGACAGGAAAACGGGACTGTGGGGAGAGGGTGGTGCAGACGGGCCGCAGACCCTCCTTAGGAAGAGAAGCCGGAAACTAGGGAGTCATGTGCAGTTAGAATCCTCTTGGAAGACTAGTAGGATGTGAGGTTGGAGAGGTGGACGACGACCAGAAAATTC

>non-ORI-246

GACGAGGGAGAGGGAGAGGGACAGGGAGAGGGAGAGGGAGAGGGAGGTGTCCATCTTTATTTCAAGGGCTTTCAAAACAGCCTGTTTGTAACCTTCATTATTAGCCACTGAGAGCTCCATTAAAGCAGCCCATTAGCTTTCTGGATCAGAGCACGGTGGGACCTTGCCTCACCTTGGCAGACATTCTTAAGTGTCATTTCCCCGAAACACTTAGTTCCTGTTCTGCTAGACCTGGCTTTTTGAAGTCTTGAAATGATAGTCATCAACAAAATTACAAAGGTCGGGCCGGGTGCAGTGGCT

>non-ORI-247

GTTCCTGTGGTGAGTTAGGCCATGTGGGGCCACTTACCCTGGCTGGAATGGTTTGAATGTGGGTTTGAAAGGGTTTTGACCCCTGTCCGGTGATGGAAGGGGCAAGTCCACTCTCACCAGGAGAGGAGGGCATGGAAAAGGCTGTAGAGCAGTGGCCTCTCACTGACCACTTCTCACTGATGTCAAAGCTCAGCAAGGTCATGAGCTTAGAGCAGGTGGGGGTGGAGGGTTGGGTAAGGCAAGGCTAGGAACAGCTGCTCAGGCAGTGGAGGCGTGAGGCGGACTGTGGTGACACGGGGT

>non-ORI-248

TAGGGGGCGCGTGCGTGTATGGGTGTGTAGGTGCAAGGGGTGTGTGTAGGTGAGGGGGTGTGTGTAGGTAGGGGGTGTGTGCATGAGGGTGTGTGTAGGTGCAGGGGTGTGTGTGTGTAGGGGTATGCGTGTATACACTTACAGTCCTGCTCCTAGTGGATTCTGGTTCAAGCCTTAGCAAGGCGGAGCAGGCAGTGAGGGATATGGCACTGTCACCTGCAGTCTGCAGCCCGCAGCCAGCTCTGCCGGAGTATATGTAGCTTTGCAGCTTGTGTGTTCAGCCAAGGACCCTGAGAACTG

>non-ORI-249

GGTAGACATATGTCCCACCATCTCCACAGCCAGCTCTTTGCTCCTCCCTGCCTTGTGTGTGGAAGACGCTTCTTCTTACCACAGAGGAGTCATCTCCTTTGAGCCATCTTCTGGCTCAAAAGGGTCACACCAGACTCTTTCCCCTCAAGTAGGGAGAAACATTCGCCACTTCTATGACAGGTGGAGCTGTGGGAACATGCTGAATAGCAAAATACTGTAACAGGACAAGGGAGGGAAGGAAGGAGAGGCAGGCAAACCACTGTGGGGATGTTCTCAGAAGGCCAGCAGAAGTAGAGGGTG

>non-ORI-250

AGACCACCTGGGACCCCCGCACATAGAGTAGCGGATACCACGGGGTGACCAGGCCCTGTGGGTGGAGGCGTCAGGAATGGGAACCGGCACTTGGGTGGGGAGGGCTGGCTGGGGTTGAGTTTCTGCTGCTCCTGCTTCCCAGGAAATGCAGCCAGGGTGGGCCCAGCAGTTCCTATGGAGTAGGAAGCCTGGACGCTGTGGTGTCTCCAGTCCCCACCCCAGGGCCCAGTCACTGGCCAGCTTGGGCCAAAAGGAGAGGCTGGACTTTAGAGGGTGGGTGTGAGTGTCTTCACTGAAACT

>non-ORI-251

AGGGGGAGGCGCTGGCGCTGTGACGGCCGCGGGGAGGGCCACCTTCCCAGCACACGCACACATACGCCCCCGGCCCTCAGCCGCTCCGGCTGCCGCTGCCCGCCGACAGCTGACCTCCAGGCACCGCAGCGCCCGGGAACAGCCATCTGGGAACCGCGACCGCGCACTGACACACGCAGTGGCCGCCGCCGCCGCCGCTCCGCCAAGCGCTGCCTGTAGCACCTGCACGGTGGTTTGGAGGGGAAGGTCTGGGCAGGCGAACGTTCCACGAGGGCGTCCCAGAGGCATCTGCAGCCTCCG

>non-ORI-252

TTTGGCAGAGTAAGCACCTATAGAGCATCATCCAGGACAGCAGGACATGAGGGTTGCAAACTCCTTAAATACCCATCTGACTCCTTCCTCTTACTGATGGGGAAACTGAGGGAACAGACCTTGCCTGAGGTCCCGCTGCTCTCAGTGGCAGAGCTGATGTCAGAATCTGGGTCTTGGGCCCCCACCCCAAGGCTTCCTCTGCCTTCCAAGGCACTGCACGTGGGACACAGCTGTGGCTGCCCCAGCTGGGCTAGCAGACAGGCTGTGCACCCCCTTATGGCAACATGGGCCTGGGGGGTG

>non-ORI-253

TTTAAGGCATTTTTGTAGCATTTCTAGCACTGCTCTCATTAGCTATAGGGCGTTATTACAAAATATTGTCTTGTCCAACACTGAACTGAGTTGAAAGACTCTGGTGAGTGAAATATGAGCCAATATGCATTTTAAAAGACAATGATATTAGCTTTGGGGCAAAATGAAACATAACCACAATGATTGTTTTTCCATGATCAGTGAACACTAATGCCACGTGCTTATGGCCCTGACAGAGGTCAGCAGCCCTCAGTTAAATCCACTAACCTGAACACTTAGGAATGGAAGTGCCAACGTGTA

>non-ORI-254

TTTAGATTCTGTTGTGTTCATGCGCCGAGCACCGCGGTGAGCGCTTGGACTTCGCCGCCGAAAAGTTTTAGAAAGGACAGCGTGTAGGCGTGTTTCAGACCATTTCATTTTCTTGGGTGAAATGTTGCTTTTTTGCTAAAACTGAGGCAAACGTTGATCTTGAGAGCTTGCAGTTTGGGGGCAGTAAAGTTGAGAAAGGATCATATTCTGTTATTGGTTTGTGGGACTGCAGGTGAAGCTGCTTTGTTTCCCTAAAAGATACTTTTTGGGGACAATAATTTGGGAGTAAAGCGGCAAGGT

>non-ORI-255

CCATCAGCTGAGCACCTAGTGATGGACATCTCCATGAATGCCATTTGGATCAATAACATTGGTGTTTCCTCCCACTGTCTTGGAACTAACTCAAGCTCCAAAAGTCTGGCGCAACTCTAGGAGAAGGGGAGGGAACCCCAAACTGACTTTTGTTCATGCTGGTGCTTAAAATAGAAAATGAACCTTAGAAAAATCCACTTACATTTCTTGCATCTGAGTCCCCCTGGGAAGGTGGATGGAGAACCTGACTCATAAGGTCGCTTATTTAGTGTTGGCAGTTTCCATAAATATTTACACCTG

>non-ORI-256

GTGGAGAGAGGTGAAGGATTCTTGGCTGTGGAGTCAGACTGCTGGAGTCTGACTGACAAGGCCTGTCAAACGCCTTCCACTGCGGATCTGACTTGAAATGGGAGCTTTGGTCTCAGTTTCTGCATGGAGGCCTGGTATGGCCTCACCCCGTGGCGGCCTCATCTTAAACCTTTCTGTTTGTGCACGCAAGCATGCATGTATCCATGTGGGTTCAACAGGTTTAAGAATGAGGCTCTCTGTAGCTATTTCAGATCCAATCACGTCAAGCCCAGACAGAGATTTAAGGCGACTAGCTTGTGT

>non-ORI-257

AGAGCCCTAGATGATTGGGCGGGGGGTGGGTACTGGGGAGTGGAGAGGGGAAGTCCTGAGCTGACCAAGCCTCCAAGAGCCATTCCGGAACTAGAGATCTTAAATACTACATGGTAATTATCCCCTCTGCCTCTGCCCATGAGGGTGTCAGGCACAGGGCCGCCTGGCTGCCTGCAGAGAAGAGCTGTTTCATTCAGAAACTTCAGAGCTCCTGCAATGCCTGGATTAGGATGTGGGGATGGTGAGTTTCGCAACCAGGGTCAGATTTCATGAAGACTTGGTTTCGTGACTTGAGGTGTT

>non-ORI-258

AGAAAGGGACCTTGTTTGGGGAGGCGGCTTTGAGCAGTGACTTTATGACATAAACGATTAGGCTCTTCCTGGCCCCGCCTGTCTTGGGATATCTACTCAGACCCTGGAACTTTGAAATCGCAGGCTGGGTCCAATGAGCGCAGAAAAGGGACAGGACTCAGGCAGATAGGATTTCTTTCGGGTTTGGTAAACAGCTGGCCTCTAGGGGTAGGGACAGGGGCAGTGGGTGGGAGGACTTGGGGGAGGGATGGGTGAGCTCGTGGGAACTGAGAAACTGTGTCTAGGGCTTCCTTAGTTATA

>non-ORI-259

GAGACCTACCTGGCCAACATGGTGAAACCCCATCTCTACTAAAAATACAAAAAAAATTAGCCAGGCGTGGTGGCACGCGCCTGTAATCCCAGCTACTCAGGAGGCTGAGACAGGAGAATCACTAGAACCCAGGAAGCAGAGGTTGCAGTGAGCCAAGATCGTGCCACTGCACTCAGTCTGGGCAACAGAGCAAGATGCCATCTCAAACAAAAAATGGACTGGATAGGCTAAGAGAAATTTCCCAAAAGCAACCAAGCATCATCTTCATGAATTCATGTTACTCGTACAAGACAATGACTT

>non-ORI-260

CATCTAAAAGCGACCCAGAGAAGGAAATGAGATGTTATTAATATTTGTTCTTATATCTGAGGTTTGCTTTGCTGCTTAAACGACTCCTTCCAGGGGAAGGGGAGGAGGGAGCGCTCTAACGTGGGGGCGAGAAGACTAGGGGTATGGCAAATTACTGTTTTCGGTGTTACGGGGGAAAGTTTCACGACTAAAGGAAACGCGTTATCCTAGGTACTGGAGAAGCTTGGAGAGCTGCCAGAATTAGTTGCAGTCCCAGGATAAAATGGGGTTTAGGGAACATTTAATGACGAAAGTTGAATC

>non-ORI-261

TGGGCTCGCGGTGCCTGGTTCCTCTTGGAGCCGCTTCTTCCGCCGCCGCCGCCGCCGCCGCCGCCGCCGCCGCCACTCGGTTCATCCTCCTGCACCAGATGGCCAATCGAGTGATCAGGCTGTACACAGTGGTAGTCAGTCTCAGGACCTCAACTGAAAGCACAGCTAGAAGGGCGACTTTCACAGGGTCGGTTAACAAAAAGATTCTAGAGCCATTATCGCACTCCATGGGTCTTTTTGTTGCTGGTGCTTATGGCCTTCCCATCATGTGTTCCCTGTCGATGTGCACTTTCTGGTATG

>non-ORI-262

ACCTGTTGATGTAGGGTGACATTCTCATCACATTCACAGATGAGGAAACTGAGATACAGAGAAGTGAAGTGACACAGCTAGTAAGTGCCAGAACCAAGATCTGGCCCCAAGCAGGCTGGCTTTATAGACTGGCTCCTATCTACTACAGAGAACTGTCTCCTTTGTAGAGCAAGGCTAGACATGCAGCCTTTATACAGCCTCCACCCTGCCAGTGTTATCACGATTCCGGAGTTCATTGAGGCCTCAGCTGGACTAAAGCAACATGCTCCAGTGCAACATCTGGACCCTTGCAGACAAATA

>non-ORI-263

GTGTGTCTGGAGAGGGCATATGAAACTGAAAGAATAAGGGCCAAAGAGCTTCCGGAAGAGGAAATTAGCTGGTGATTCTTTCCAATCTGCTTTCCCAGTCACCTTTCCGGCATGTTCTGTAAAACAAAATGTAATTTGACTTTGTTTCATTATTTTAACTCCATAACACTTAAATTGTTTTCTTTTCGGGGAGGGGAGTGTTTATTCTCAAACTAAGCAGTAGCCCTCTCCACCTTCGAAAAAGGAACAACGTCAATCCAACCTTTGTTAAAAAACTCCAACCTATCAGATTTTAATAAT

>non-ORI-264

CCTGTGCGTTTTCACTCCACACTTTTATTCTTGCTCGGGGTCGACTTTCTTTTTTCTTCTCCCCTGGATCCGGAGATCTGCGGACGGCGATTTTGGGACGAAAAGGGTCGGATGGGCCGTGGGAGAGAGAAGGTGTGGGAAACAGCATCAAGTAGTTCCGCAAAACTTTGAGCCCCGTTCCCATTTTCTTTTGGCCTAGAATTGTGAACGTATTGTTGATTTCGGCCTTTTTTTCCCCCCGTCGCGAGAATGTCTTGTTTTGCCTTTAGTTTTTTATAAACCGCTGTACTTTCGACATTT

>non-ORI-265

ATTTTTTCATCTAAAAGTATTTTAAGATATTTTTAAAATCCAAGAGCTTCTCTATACTTTTCAGAAATATCCAGATGCAGTGAACTGCCAGAAGGTAACCAGTCTCAAACATGCTTATCCCATTATCAACCCTGAAAGTTTGCTTGTCCTTTAAGATAAAAATGTAATGTTGTGATATTCCTTCCAGTAATGCCACTGTATTTTGTCTCCAAATAAAAGAAGCTTATTGTAGTATGTTTGCAGAAAAATTCTAAACAAAAATTATACAGCTTATTAGAGTGTGGGAATAGGGATCTAAAT

>non-ORI-266

CTTGTTGCCCCATCAGCTCCCCTGTGCATGGCATGCACATCCCCGTATGTGTCTGCATGTGCAGGTGTCCGTGGGCATGTGTGTGGTTGTGGATCTGCAAGCAGGGAGTGAGCTGTATGTGAGCTGGTATTTCTGTGGTGTGTCTTCTCTGGGAGGGGTTGCACTCATGAGTGGGTGTGTTAGTGTGTGTGCCTGAGGGAATCAACACACAAGTCTGCTAGCTACGTGTGTCTTCTTGTATGTCTTTATGTATCTGTGGGGATGAATCCCTGTATCTATTATCTCTGTGTGCACACACAT

>non-ORI-267

TTTCACCCCTTCCTTGCCTGGGCCCAGCGAGGAGCAGGAGGACCCCAACTTGGGAAGGGGGCGTGAAGCTCCCATGAAAAAGCAGCGCAGTCCTGGGCCAGACCCACCGCTCTCTGGCCTCAGTTTCCCCACTTATAAAATGGGAATGCTGAACTAAGTGACCTCCCAGAGCACTGCCAACCCAGACAATCCAACCAGGGGGGCGTGGTAGGGTGAGGGTGGGCGTGGAGGCTATGGCAGTGGGAGGAGGGAGTCCTGGGCCGGGACCAGGGAGGCCCTGGGGAAAGGAAGGGAAACAGG

>non-ORI-268

TGACGCCATCGGAAGCCCTGGTGATGCCGGACTCTTCGTAAAGTTCCAAGTCGGCCCGAAAGGGGCCCAATGTAATCCCAGATCATTGACAATGCCCATCAGGATAAACTGCTGCAGGAAAAACTAGCTCGACGGAGAAGGATAAAGAAGCCCCATTCCATCTTACTGCCAAGTTTGTGTCACGAAATTTAAAAACACAGAGAAACCAGCCTAGAACAATGTATGGGCAGCTACACGGCTGCCTGCTGCACGGTTTGAACAGTTAGTGTCTTGCCCTCCTTGGCCATTGCGGGGGTGCAC

>non-ORI-269

GCGGGGGAGGGGGCGGGGGCGGGGCGGCTCAGCAGGGCGCCCAGCATCTCCTCGCACATGGCTAGGCACCGCGGCACGTGGAAGCAGCCTGCGGGTAGAGGCGGGCAGGTAGTGAGGGCCGGGGGCAGCCGAGAGGCGCAACCCCTGCGGCCCCGCCCTCTCCCCCACTCACCTTTGAAAGGACCTCCCTTGATGGAGAGGGCCACCTTGCCCTCTCGGCTCAGGTAGCCAAACCATTCCCCGTACTCGGGATCGCGAAACTGGAATAACAGAGACAGTGACAGCTAGCGCCTGCAGGAC

>non-ORI-270

TTAGTGCAGCCGCCGCTCCCGAAACAGCCTCGACACACCTAACGAATGGAGGCGGGCCGGAGGCCGGTGGAACCAGCTCGAGCTCTCGAGGGCCGTTTGGGGGCTCGCGCCGTACGGCTTGTGAAGTCTCGCGCCTCTCCCCTTAGTCCGCCTCTGCTGCTCAGGCTGCGCCTGCCGCCGTGGGAGCCGCCATCTTGAGACTAGCTCCCCGTTCCCCCCTATTCTCTTCCTCCTAGGTCAGTTCTTCCACTGCACACCAAACTCAAGGCGGTGTCCGATCCTCACTTCCCGCCTTATGAC

>non-ORI-271

CTGCCACCACACTCCCCGGCTTTCGGCCCCGTTTCTTGGGAATGGCCTGAGGGTCGGCCTCAGCTTTTCGCTTCCTGCCGGGGCGTTTGATCACCATGACCTGGGTGGATGTGGTGGCCCCACCCCCCTCAGCCTTGCCCCCTGGCGAAGTTTGAAAAGGCATCTTGACAAGGAGCTTCCCAGGACTTTTCTCCAGGACCCTTTTCACCTGCACACCCTCTGACGTGGCCGCCTTGGGTCTCGTGGTGCCGCTCCCTTTGGGGCGTCCCCGGCCTCTGCCAGTTCCTGGAGCTTTGGGAG

>non-ORI-272

CTTGGGACGCCTGTTTGCGCTGCTCTGAGGGGCGATTGACCCTCTGTCCACAGATGCACCGAGTGCGCTGCAATAAGCGTTTCATTGCACCCACATTTTCTCAATATCACTATCTTCGGCAGAAGCAGCAAAGTTGCCCACCCTGGAGCCTGACGTTGCCCCAGCTGAGGGGCCTTGGTTTCAGAGGCCTACTCAATGCATTATTTCATCCGAGCCTGCCCAGTGCTCACACTCTCATAGAGGCGGGGAATAAACTGACAACAAATCGCAACCCTGGCTTTCGCAGAACTCTGGCGGGGG

>non-ORI-273

AGAGCAAAACACACGAGTCTCCGGGATTCCTCACTTGGGAAACGTCAGAGAGTCATTTTCGTGAATGACAGAAATGACATGGGAAAGATGAGCCAGCTGGCGTCCTGTGGCAGGCAGATGGGCTTACTAGATGTGGCTGCACACACTTGCTTCTAGAATGCCAGAGATGCACGCTGGAAAGCCATCTCAGGGACTCCCTGGGAGAATGAGGCTGGACGAGATGGCTCTAGATGGAGCTAGAGTCACGGCTGTCTGGGTGAAGCTGGAGGCACAGGTGGTGGCCAGAGAGAGGAGGCGCGG

>non-ORI-274

GCTTCTGGGCCAGGCAGCCCTGTGCAACCCGAGCTGCTCCCCTGTCCCCGCTGTCCCACCGCCCTCCCTGGACGCCTGCTACCCACCATCAAACACCGCCTCGTGCCGGCCCCGCGCACCCTCCCGCCCCGTCGGCCCCACGCTTCAGGCGCTACCTGGAGGGCATGGCTCCTACCCGTGGGCACTCGCCTTCACCCTTGGAGCCCGGCCTTCCCCGGGCAGCCGGGAGAGGTTCTACCACTTCACGAGGCCTCCTCGCCCTGCCAGGCCGAGGTCAGGGTTAGAGAAGCTGGGTCGTTA

>non-ORI-275

CTCCAGCGCCCACAGGGACTGCAGGGCTCGTGTCTAGGCCCAATCACAAGGATCCTGCGTGTCTGAGTCTGGGGGAGCCAAGCGCACCCCAGGTGGAAAGGCCGAGGCCCAAGGCCACCTTCTCCAAGGAGCCACCCACAGTCACAGCCCCTGGTGTCTGTCCCAAGCCGGGATTGTTTCCTGGGGTGGGGGCAACAGGTCGTTGCAGGTGTTGGCTGGGCGCCTACGTCAGGCAGGGCCCCGGGACGGGACTGCAGGGCTCCGAGCCCTGGGATGAACCTGACTGCCATGGATCAGGCG

>non-ORI-276

ATCTTGTTGGGGGGAGGGGATTAACCAAAGGCCACCCTGACTTTGTTTTTGTGGACACACAATAAAAGCCCCGTTTATTTGTAATGCGTTGGCTCTTCCTGGAGGAGAGGGTTGGGCTCCCATGGCAAGGGCCTCTGCGTCTTGGGGCTCCAGGATTGCAATCCGGCTTTGTTGGGTCCGCATTTTTGCTTTAGTCTGGGGATAGGAATCAAATGTTACCCAGAGATGTTTGTGTTTTGTTTGGGAGTTTTATTCCCTAACTCATTCCCCAAAGCACGTGTAACTGCTTATACATATAAT

>non-ORI-277

CTGAGCACACTCAAGTCCGAAGATGTCCCATACACAGCGGCCCTCACAGCGGTCCGCCCTTCCAGGGTATGTGCCCTTCCAGCAGGGGCTCTGGGGCGTGCAGGGAGAGGCAGTGTGGTGAGTCTGCTTGGAGGTGGGGAGTGTATGCCACAGGTAGGCTGCCCAGGAGGCCCAGCATAGGGGAAGCATGAGGCACAGAACCCCTGTGGTGTGACTATTTGGAGTGGCTGACTCTGGGGGCAGGGCTAGGATGAGCACTCAAGTCCTGCCTCCCCATTCCAGGGGCAGCTTTCCATCTCG

>non-ORI-278

TGATCCTTTTGGGGAGACTCACTCATCAGGACTCGGGGCTCCAGGGGAGAGCAGGCTCCTGCCTGGGGAACTGGGGAAGCCTGGCCAGAGGAGCCTTGAAACACCAAGTCCTCATGCCCTGGCCGCACTGTCTGAGCCGGGGTGCCCAGCAAGCAGAAGGTGTATTTCTCTGCCAGCCACCTTCTTGGGAGACCACTGGTCAGCGAGGGCTTGACATGGTACCAAGTGGGTTGGGGCTGGGGCCAAAGAGAGGCCTCCTGGGAAGCTGTTGATGACATTCTGGGGCCTGGAGGGAGGAAA

>non-ORI-279

CGTGCGGGGCCAAACAGCTCGGGGCCCGGGGCTCCGGCAGCCCGGGGGTCCGGCTCGGCCTGTGGGGGTCAGGCCCTATCCCTGCCCGTCGGCGCCGGCCTGCCCGACTCCTTGTGCCCCGCATGGTGGGGACGCCAACCCAAGACCAACACCCTGCCCAAAGAGGACCCTTTGTCGCAGGGGAGCGGCCCCTCGGAGGATCAACGCTGTACTTCCCTCCCGCTAAGGGCTCCGCGAGTCCGCCGAGGGGTGCCCTGATGGGGCCCGGGCAAACCGGACCAGCAGCACGGTGGGGGGTGT

>non-ORI-280

TGGTAGAGAGGGCAGAACCAGGCTGGGGGAGGCCCTGACACCACCCACCTTGAAGAAGGGCTCACTCTGTTTGCGGATGTCAGCCACTGTGAGGCGGGAACGGGCATAGCCCACGATGAAGGTGTTTTCGGGCAGAAGGCCATCCCGGAACAGCCACCTGAGGGCAGGGCACAGCTGTAACCAGTGCGGGCAGGGCAGGACCAGGCCTGTCCCTGGCGGGAGGTCACAGGGGCAGTGGTGGGACACACTTACCAGATGGTGGGGTAGATCTTCTTCTTGGCCAGGTCACCCTGTGGCAGA

>non-ORI-281

CTTCCGCCGGCAGCGTGGCCACGCCTCCTCTGGGCTGTCCCTCGGCTCCTGGGCGGGGCCTTCTGCCGCGCCACTCCCCCGGGAACCCTCGCCTGTTCGCGGGTCAAGCCGGCCCTATTGGGCAGTCTCTTCTTGATTCCTCCCCCGGAGAGGGCGGGGCGGCCGAGCGCCCCGAGGCTAGACGCCGCCGTCCGAGAGACGAGGGGGCGTGTAGGCGGTGACGCCCTGCAAAGTGGCCGGCGTGCTTATCATTACCGAGCTTCCGCGGGCCTGCAGAGCCTGGCGGACTCAGACTTCTCT

>non-ORI-282

TCACGCGCGTGTCTGGGGCCTAACCACCCTGGGGTGTCGTCTCCAGTGGAGCTCACCCAGCCTTCCGGCGCCGCCCCCGGCGCAGAAGCCCGAAGACATTCTGTCCCCGTTCTCGGAAGCGGTCTGGCTTCTTTGTCCCTTTGGACGTTCATCTCAAATGCCTCGTGGCGTCATTCTGTGTACAAGGCACCTCCCCAACTGCTTTATTGTTGGTTTTGAAAAACTAGCACATATGGGATGGTACTTAGGAATAGCCTTCTGTTTAGTCGGCATAGTCAAAGATTCCGTGTAATTCGCAGT
